# Dehydropolymerization of H_3_B·NMeH_2_ Mediated by Cationic Iridium(III) Precatalysts Bearing κ^3^-^*i*^Pr-PN^*R*^P Pincer Ligands (*R* = H, Me): An Unexpected Inner-Sphere Mechanism

**DOI:** 10.1021/acscatal.2c03778

**Published:** 2022-10-12

**Authors:** Claire N. Brodie, Lia Sotorrios, Timothy M. Boyd, Stuart A. Macgregor, Andrew S. Weller

**Affiliations:** † Department of Chemistry, University of York, York YO10 5DD, U.K.; ‡ Institute of Chemical Sciences, Heriot-Watt University, Edinburgh EH14 4AS, U.K.; § Chemistry Research Laboratories, University of Oxford, Oxford OX1 3TA, U.K.

**Keywords:** dehydropolymerization, iridium, mechanism, amine-borane, catalyst, metal−ligand cooperativity, polymer, kinetics

## Abstract

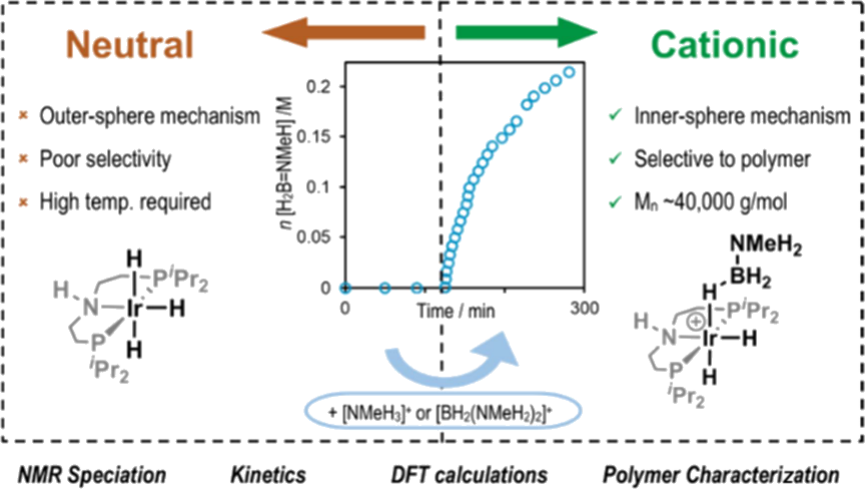

The dehydropolymerization of H_3_B·NMeH_2_ to form *N*-methylpolyaminoborane using neutral and cationic catalysts based on the {Ir(^*i*^Pr-PN^H^P)} fragment [^*i*^Pr-PN^H^P = κ^3^-(CH_2_CH_2_P^*i*^Pr_2_)_2_NH] is reported. Neutral Ir(^*i*^Pr-PN^H^P)H_3_ or Ir(^*i*^Pr-PN^H^P)H_2_Cl precatalysts show no, or poor and unselective, activity respectively at 298 K in 1,2-F_2_C_6_H_4_ solution. In contrast, addition of [NMeH_3_][BAr^F^_4_] (Ar^F^ = 3,5-(CF_3_)_2_C_6_H_3_) to Ir(^*i*^Pr-PN^H^P)H_3_ immediately starts catalysis, suggesting that a cationic catalytic manifold operates. Consistent with this, independently synthesized cationic precatalysts are active (tested between 0.5 and 2.0 mol % loading) producing poly(*N*-methylaminoborane) with *M*_n_ ∼ 40,000 g/mol, *Đ* ∼1.5, i.e., dihydrogen/dihydride, [Ir(^*i*^Pr-PN^H^P)(H)_2_(H_2_)][BAr^F^_4_]; σ-amine-borane [Ir(^*i*^Pr-PN^H^P)(H)_2_(H_3_B·NMe_3_)][BAr^F^_4_]; and [Ir(^*i*^Pr-PN^H^P)(H)_2_(NMeH_2_)][BAr^F^_4_]. Density functional theory (DFT) calculations probe hydride exchange processes in two of these complexes and also show that the barrier to amine-borane dehydrogenation is lower (22.5 kcal/mol) for the cationic system compared with the neutral system (24.3 kcal/mol). The calculations show that the dehydrogenation proceeds via an inner-sphere process without metal–ligand cooperativity, and this is supported experimentally by N–Me substituted [Ir(^*i*^Pr-PN^Me^P)(H)_2_(H_3_B·NMe_3_)][BAr^F^_4_] being an active catalyst. Key to the lower barrier calculated for the cationic system is the outer-sphere coordination of an additional H_3_B·NMeH_2_ with the N–H group of the ligand. Experimentally, kinetic studies indicate a complex reaction manifold that shows pronounced deceleratory temporal profiles. As supported by speciation and DFT studies, a key observation is that deprotonation of [Ir(^*i*^Pr-N^H^P)(H)_2_(H_2_)][BAr^F^_4_], formed upon amine-borane dehydrogenation, by the slow in situ formation of NMeH_2_ (via B–N bond cleavage), results in the formation of essentially inactive Ir(^*i*^Pr-PN^H^P)H_3_, with a coproduct of [NMeH_3_]^+^/[H_2_B(NMeH_2_)_2_]^+^. While reprotonation of Ir(^*i*^Pr-PN^H^P)H_3_ results in a return to the cationic cycle, it is proposed, supported by doping experiments, that reprotonation is attenuated by entrainment of the [NMeH_3_]^+^/[H_2_B(NMeH_2_)_2_]^+^/catalyst in insoluble polyaminoborane. The role of [NMeH_3_]^+^/[H_2_B(NMeH_2_)]^+^ as chain control agents is also noted.

## 1 Introduction

**Scheme 1:**
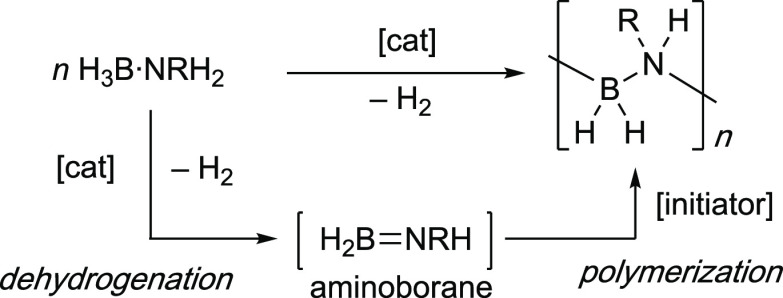
Amine-Borane Dehydropolymerization

The dehydropolymerization of primary amine-boranes,^1−5^ H_3_B·NRH_2_ (R = alkyl), allows for the atom-efficient formation of main-group polymers with B–N main chain units, polyaminoboranes, (H_2_BNRH)_*n*_,^6−8^ with H_2_ as the only byproduct. Dehydropolymerization is proposed to occur through a two-step process, in a cascade-like^9^ polymerization, Scheme 1. Dehydrogenation of the amine-borane premonomer first forms a transient^10,11^ aminoborane monomer that then undergoes polymer chain propagation.^10,12−14^ Since the initial report^1^ by Manners in 2008 that Ir(POCOP)H_2_ [POCOP = κ^3^-(OP^*t*^Bu_2_)_2_C_6_H_3_] can act as an efficient amine-borane dehydropolymerization catalyst to give high-molecular-weight polyaminoborane, the use of various transition metal catalysts based upon Ir,^15^ Ru,^16^ Fe,^17−19^ Co,^20−23^ Rh,^24−29^ Zr,^30^ and Ti^31,32^ has been demonstrated. Polyaminoboranes are isosteric with simple polyolefins, and in addition to the fundamental interest that surrounds such main-group polymeric materials,^8,33^ they have potential piezoelectric applications^34^ or as polymeric precursors to BN-containing materials.^35−39^

**Scheme 2:**
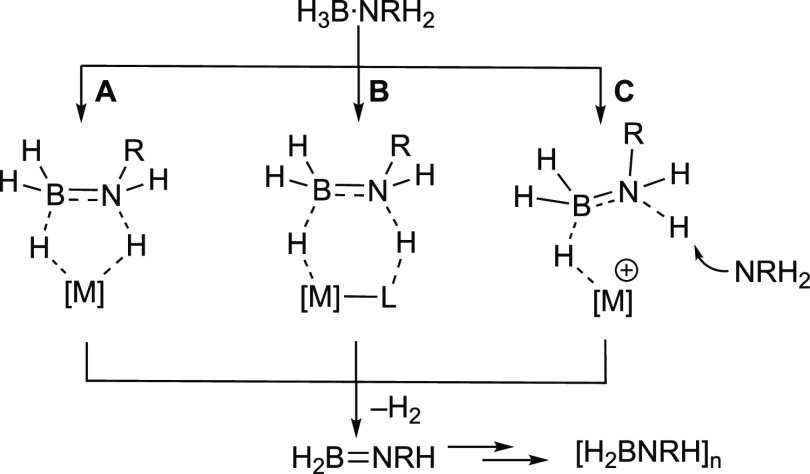
Pathways for Amine-Borane Dehydrogenation: (A) Inner Sphere, (B) MLC, and (C) Hydride Transfer

While the precise details of chain growth processes remain to be resolved, due to the transient and highly reactive nature of aminoborane monomers, the mechanism is likely a rapid head-to-tail chain growth polymerization initiated by the catalyst or other Lewis-bases: as first proposed by Manners^5^ and Baker,^40^ and supported by computational studies,^12,41^ polymer growth kinetics,^5,27^ and analogous phosphinoborane polymerizations.^14^ In contrast, the understanding of the initial catalytic dehydrogenation of amine-boranes to give aminoboranes is better developed, as reaction progress can be conveniently followed by H_2_ evolution as a proxy for aminoborane generation, catalyst speciation, and isotope labeling studies. There are three accepted pathways for dehydrogenation, Scheme 2: (A) stepwise or concerted inner-sphere BH/NH activation;^42−45^ (B) metal–ligand cooperative (MLC) processes;^16,19,46,47^ and (C) amine-promoted hydride transfer at a cationic metal center, followed by reprotonation to release H_2_.^48−50^

Group 8 catalysts with the ^*i*^Pr-PN^H^P ligand [^*i*^Pr-PN^H^P = {κ^3^-(CH_2_CH_2_P^*i*^Pr_2_)_2_NH] have been reported by Schneider and Beweries to be particularity effective for the dehydrogenation of amine-boranes, operating via MLC mechanisms (i.e., B, Scheme 2). Examples include Ru(^*i*^Pr-PN^H^P)(H)_2_PMe_3_,^16,51^ Fe(^*i*^Pr-PNP)H(CO),^18,19^ and Fe(^*i*^Pr-PN^H^P)(H)(HBH_3_)(CO).^17^ We also have recently reported the use of this ligand with group 9 systems for the dehydropolymerization of H_3_B·NMeH_2_ to form *N*-methyl polyaminoborane. While the precatalyst Co(^*i*^Pr-PN^H^P)Cl_2_^20^ promotes rapid and selective dehydropolymerization, mechanistic studies were frustrated because of the paramagnetic nature of the species present, although the NMe variant, Co(^*i*^Pr-PN^Me^P)Cl_2_, was inactive suggesting a cooperative ligand mechanism. In contrast, by using diamagnetic [Rh(^*i*^Pr-PN^H^P)(NBD)]Cl as a cationic precatalyst, the generation of an active neutral dehydrogenation catalyst, Rh(^*i*^Pr-PN^H^P)H_3_, was revealed,^52^ and dehydrogenation operates via a cooperative ligand mechanism. It was proposed that Rh(^*i*^Pr-PN^H^P)H_3_ forms from an NMeH_2_-promoted hydride transfer (C, Scheme 2)^48,49^ from H_3_B·NMeH_2_ to in situ generated Rh(^*i*^Pr-PN^H^P)H_2_Cl. The eventual boronium coproduct of this, [H_2_B(NMeH_2_)_2_]Cl, was also shown to act as a chain-transfer agent that modifies the degree of polymerization in the resulting polyaminoboranes. This system can also be used at low catalyst loadings (0.01 mol %) to selectively produce (H_2_BNMeH)_*n*_ in a controlled polymerization, on a scale (10 g) that allows for the study of the resulting materials and processing properties.

**Scheme 3:**
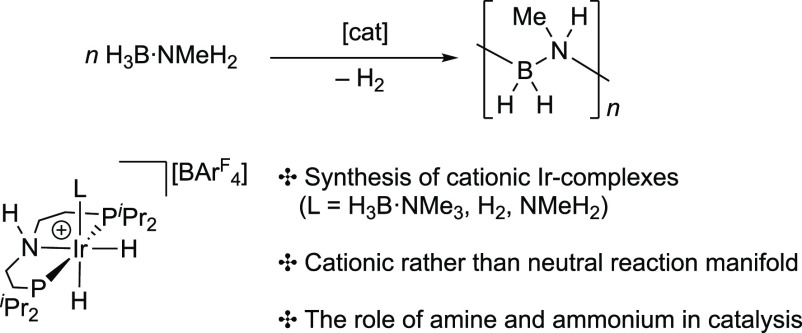
This Work

Given that Rh(^*i*^Pr-PN^H^P)H_3_ is an active catalyst and that the iridium congener Ir(^*i*^Pr-PN^H^P)H_3_, reported by Abdur-Rashid,^53^ promotes a variety of outer-sphere hydrogen transfer processes,^54−56^ were we interested to explore whether neutral Ir(^*i*^Pr-PN^H^P) complexes also promote rapid and selective amine-borane dehydropolymerization. Here, we report that, surprisingly, under the conditions used they are poor to inactive catalysts for the dehydropolymerization of H_3_B·NMeH_2_. Instead cationic species, such as the σ-amine-borane complex [Ir(^*i*^Pr-PN^H^P)H_2_(H_3_B·NMe_3_)][BAr^F^_4_], are competent catalysts at room temperature, selectively producing (H_2_BNMeH)_*n*_, Scheme 3. While the resulting kinetics are complex and suggested to be modified by polymer precipitation/coproduct or catalyst entrainment, an inner-sphere mechanism is proposed for dehydrogenation that proceeds via an asynchronous, concerted N–H/B–H activation rather than a cooperative pathway involving the ^*i*^Pr-PN^H^P ligand, in which NMeH_2_ and ammonium, [NMeH_3_]^+^ (or its operational equivalent boronium [H_2_B(NMeH_2_)_2_]^+^), play a role in shuttling between observed resting states. These studies are supported and informed by independent synthesis and speciation experiments, as well as density functional theory (DFT) calculations. The NMe variant, [Ir(^*i*^Pr-PN^Me^P)H_2_(H_3_B·NMe_3_)][BAr^F^_4_], is also a competent dehydrogenation catalyst, supporting an inner-sphere rather than an MLC mechanism. While it is well established that inner-sphere mechanisms can still operate with ligands that can support MLC processes,^57−59^ for amine-borane dehydrogenation/dehydropolymerization this is an unexpected observation.^3,4,16−19,46,51,52,60−62^ These studies again demonstrate that the mechanism of amine-borane dehydropolymerization is highly catalyst-dependent.^2^

## 2 Results and Discussion

### 2.1 Trialing a Neutral Ir(^*i*^Pr-PN^H^P) Precatalyst

**Scheme 4:**
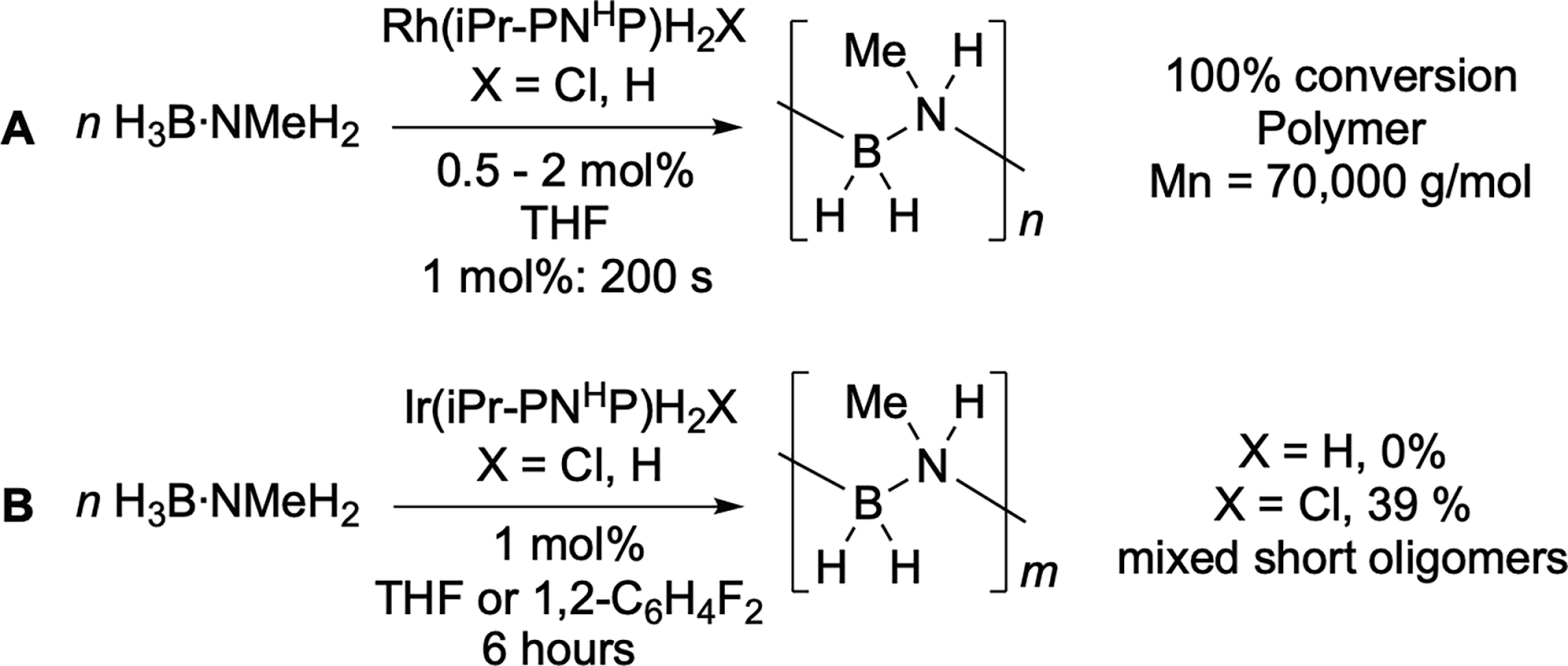
Difference in Reactivity between Neutral Rh (A) and Ir (B) Systems in H_3_B·NMeH_2_ Dehydropolymerization

In our previous study,^52^ we reported that the neutral complex *cis*-Rh(^*i*^Pr-PN^H^P)H_2_Cl was an excellent precatalyst for H_3_B·NMeH_2_ dehydropolymerization at room temperature, forming the active catalyst Rh(^*i*^Pr-PN^H^P)H_3_ in situ. We thus first looked to the analogous iridium system *cis*-Ir(^*i*^Pr-PN^H^P)H_2_Cl^53,63^ (**1-H_2_Cl**) as a precatalyst for dehydropolymerization, in THF solvent at room temperature. However, this neutral iridium complex was a poor precatalyst at 1 mol % loading, returning unreacted H_3_B·NMeH_2_ (61%) alongside a mixture containing short-chain oligomers and the cyclic triborazane (NMeHBH_2_)_3_ after 6 h (Scheme 4). In contrast, *cis*-Rh(^*i*^Pr-PN^H^P)H_2_Cl promotes 100% conversion in ∼200 s with excellent selectivity and pseudo-zero-order kinetics for H_2_ evolution.^52^

To discount whether this unexpectedly unselective and low activity of **1-H_2_Cl** resulted from the inability to form the putative active species Ir(^*i*^Pr-PN^H^P)H_3_, **1-H_3_**, this complex was prepared independently^53^ and deployed in catalysis. At 298 K, **1-H_3_** showed no activity at 1 mol %, being returned unchanged after 2 h. This poor, to no, activity at room temperature for both systems shows that a neutral Ir(^*i*^Pr-PN^H^P)H_3_ catalytic system is not operational for iridium. We discount an inhibitory solvent effect, as changing from THF to 1,2-F_2_C_6_H_4_ solvent did not change the outcome of the reaction. No borohydride^19^ or amido-borane deactivation products are observed.^17,64^ Heating H_3_B·NMeH_2_/**1-H_3_** (1 mol %) to 60 °C (30 min) in 1,2-F_2_C_6_H_4_ solvent did result in conversion, but this produced an ill-defined bimodal polymer/oligomer mixture as measured by gel permeation chromatography (GPC) and ^11^B NMR spectroscopy. This result is also consistent with an early report by Fagnou and co-workers on the poor performance of *trans*-Ir(^*i*^Pr-PN^H^P)H_2_Cl/K^t^OBu for H_3_B·NMeH_2_ dehydrogenation at 50 °C.^62^ Under the same conditions but without catalyst present, H_3_B·NMeH_2_ is unchanged.

### 2.2 Catalysis by Cationic Species

**Figure 1:**
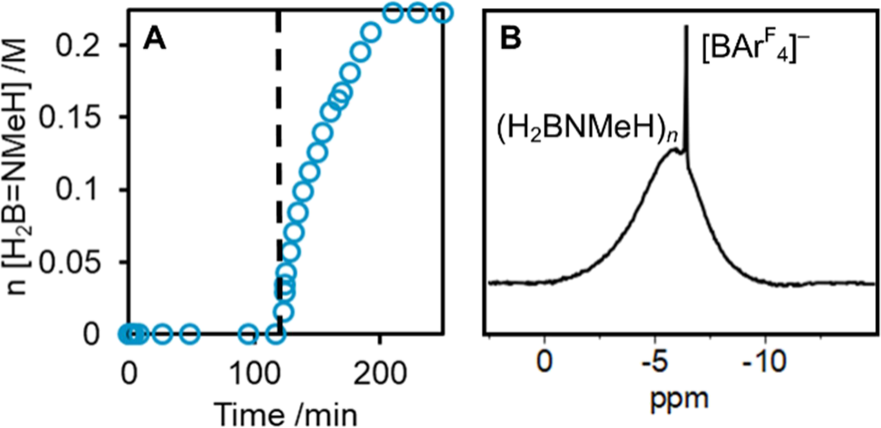
(A) Dehydropolymerization of H_3_B·NMeH_2_ (0.223 M in 1,2-F_2_C_6_H_4_) at 1 mol % **1-H_3_**. H_2_B=NMeH equivalents formed from H_2_ evolution (eudiometer). Dashed line indicated addition of 2 equiv. [NMeH_3_][BAr^F^_4_]. (B) Baseline-corrected ^11^B NMR spectrum of the reaction mixture postcatalysis.

Given this poor and unselective reactivity of the neutral systems, we turned our attention to the investigation of cationic iridium catalysts for H_3_B·NMeH_2_ dehydropolymerization to see if this opened up a productive mechanistic pathway. While **1-H_3_** is inactive at room temperature (1 mol %, 1,2-F_2_C_6_H_4_, 0.223 M H_3_B·NMeH_2_), addition of two equivalents of [NMeH_3_][BAr^F^_4_] after 110 min of inactivity immediately starts productive turnover, as measured by H_2_ evolution (eudiometrically), Figure 1A. Catalysis proceeds to completion with one equivalent of H_2_ released, at an initial rate of 2.3(3) × 10^–4^ M/s. Polyaminoborane (H_2_BNMeH)_*n*_ is produced selectively (92%) and can be isolated in moderate yield (44%) as a white solid by precipitation into hexanes. ^11^B NMR spectroscopy of the postcatalysis mixture shows the characteristic broad signal at δ −5.90, Figure 1B,^5^ alongside a sharper signal for [BAr^F^_4_]^−^ at slightly higher field. GPC analysis (THF, 0.1 w/w% [NBu_4_]Br, relative to polystyrene standards) shows a monomodal distribution *M*_n_ = 18,700 g/mol (*Đ* = 1.3). Very similar results are obtained when boronium [H_2_B(NMeH_2_)_2_][BAr^F^_4_] is used instead, reflecting that that these two proton sources are interchangeable in this system. A control experiment shows that addition of [NMeH_3_][BAr^F^_4_] to H_3_B·NMeH_2_ did not result in reaction.^65^

As discussed later (Section 2.9), addition of [NMeH_3_][BAr^F^_4_] to **1-H_3_** results in the clean formation of the cationic amine adduct [Ir(^*i*^Pr-PN^H^P)(NMeH_2_)(H)_2_][BAr^F^_4_], **[1-NMeH_2_][BAr^F^_4_]**. This supports ammonium (or boronium) in promoting the movement from an inactive neutral system to an active cationic manifold. As it is likely that cationic amine-borane σ-complexes are intermediates in catalysis, as demonstrated previously,^3,4,43,66^ the synthesis of suitable precursors is described next.

### 2.3 Synthesis of [Ir(^*i*^Pr-PN^H^P)(COD)][BAr^F^_4_]

**Scheme 5:**
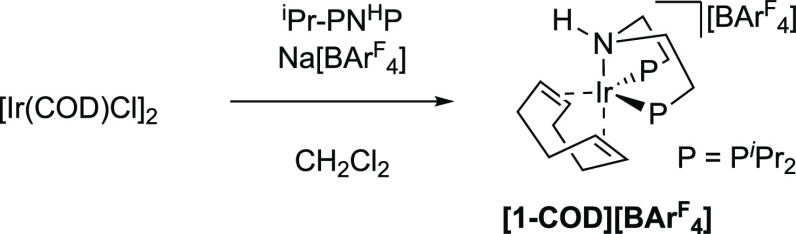
Synthesis of [1-COD][BAr^F^_4_]

An entry point to cationic σ-amine-borane complexes is labile dihydrogen complexes.^67,68^ A suitable precursor to such a complex is the conveniently synthesized [Ir(^*i*^Pr-PN^H^P)(η^2^,η^2^-COD)][BAr^F^_4_] **[1-COD][BAr^F^_4_]** (Scheme 5). **[1-COD][BAr^F^_4_]** was isolated as an analytically pure crystalline material in 87% yield and characterized by NMR spectroscopy and single-crystal X-ray diffraction (see Supporting Materials). These data show a κ^3^-P,N,P-coordination of the tridentate ligand and η^2^,η^2^-coordination of the diene, at a *pseudo*-trigonal bipyramidal Ir center with C_s_ symmetry. In the ^1^H NMR spectrum, four distinct isopropyl methyl environments and two alkene signals are observed, with a single environment observed in the ^31^P{^1^H} NMR spectrum.

### 2.4 Synthesis of [Ir(^*i*^Pr-PN^H^P)(H)_2_(H_2_)][BAr^F^_4_]

**Scheme 6:**
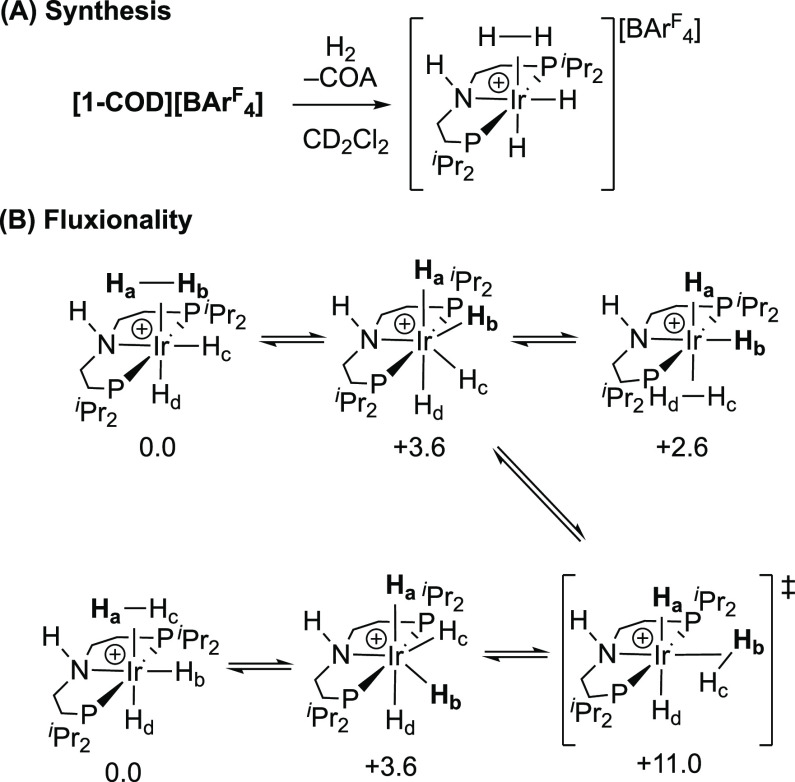
(A) [1-H_4_][BAr^F^_4_] Synthesis and (B) Fluxionality [BAr^F^_4_]^−^ anions are not shown. DFT-computed free energies (kcal/mol) for stationary points along the exchange pathway are also indicated. Level of theory: BP86[D3BJ,CH_2_Cl_2_]/Def2TZVP//BP86/SDD (Ir, P, with polarization on P); 6-31G** on all other atoms.

Addition of hydrogen (2 bar) to a CD_2_Cl_2_ solution of **[1-COD][BAr^F^_4_]** in a J. Youngs NMR tube results in the slow (36 h) but quantitative hydrogenation of the coordinated COD to give free cyclooctane (δ_H_ 1.53). The accompanying organometallic species formed was characterized in situ as [Ir(^*i*^Pr-PN^H^P)(H)_2_(H_2_)][BAr^F^_4_] **[1-H_4_][BAr^F^_4_]**, Scheme 6A. In the room-temperature ^1^H NMR spectrum (500 MHz, CD_2_Cl_2_), a broad triplet at δ −10.17 [*J*(PH) = 7.6 Hz] of relative integral 4H is observed, assigned to hydride ligands, which sharpens to a singlet upon ^31^P decoupling. T_1_ is measured as 232(8) ms at this temperature for the hydride signal. This single hydride environment indicates that rapid hydride site exchange is operating at 298 K, being similar to that reported for [Ir{κ^3^-NH(CH_2_CH_2_P^t^Bu_2_)_2_}(H)_2_(H_2_)][PF_6_].^69^ Cooling broadens and splits this resonance, and at 185 K, it resolves into two very broad signals (fwhm ∼1000 Hz) at δ −6.57 and −13.85, each with relative integral 2H. T_1_ is measured as 150(10) ms for both of these hydride signals. In the ^31^P{^1^H} NMR spectrum, a single peak is observed at δ 53.7 which does not change significantly on cooling.

The observation of two hydride environments (albeit broad) at low temperature is consistent with a low-energy process in which the hydrogen atoms remain as distinct pairs that do not cross the IrP_2_N plane and are undergoing site exchange within each pair via H_2_ rotation (i.e., H_*a*_/H_*b*_ and H_*c*_/H_*d*_, Scheme 6B). DFT calculations indicate a very low barrier of 3.6 kcal/mol for this process (see Supporting Materials for full details).^70^ Similar behavior has been noted for Ir(P^*i*^Pr_3_)_2_ClH_2_(H_2_).^71,72^ At higher temperatures, exchange between all four hydrides becomes accessible, which results in the dihydrogen ligand moving from being *syn* to *anti* with respect to the N–H group. Calculations suggest that this occurs through a *trans*-dihydride dihydrogen transition state that corresponds to rotation of the dihydrogen moiety. A higher barrier of 11.0 kcal/mol is computed for this exchange process. Consistent with a Ir(V) tetrahydride being a common intermediate is the measured T_1_ time (150 ms), which would be expected to be much shorter (<30 ms) for an alternative bis-dihydrogen Ir(I) intermediate.^73^ MXL_2_(H)_4_ complexes are known to be highly fluxional, with potential energy surfaces where tetrahydride and dihydrogen/dihydride structures are close in energy.^74^ Removal of the hydrogen atmosphere from **[1-H_4_][BAr^F^_4_]** prepared in situ results in loss of H_2_ (∼40% after 10 min) and decomposition.

### 2.5 Synthesis of [Ir(^*i*^Pr-PN^H^P)(H)_2_(H_3_B·NMe_3_)][BAr^F^_4_] and H/D Exchange with D_2_

**Figure 2:**
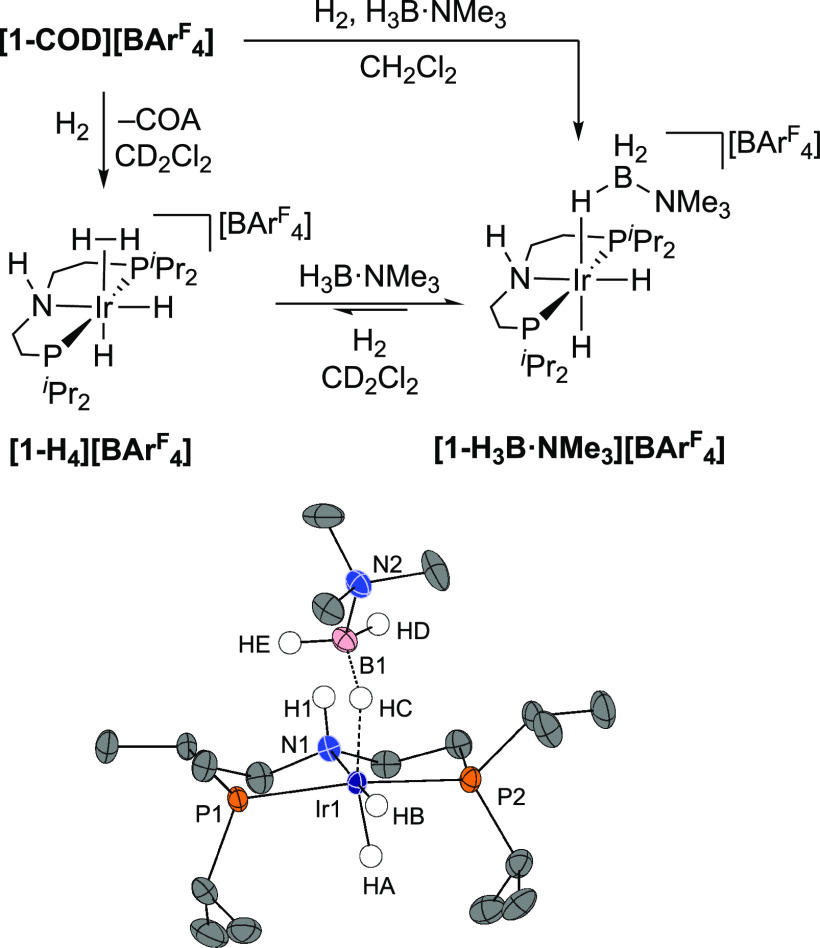
Preparation and molecular structure of **[1-H_3_B·NMe_3_][BAr^F^_4_]**. Anion not shown. Displacement ellipsoids at 50% probability level. Selected bond angles (°) and bond lengths (Å): Ir1···B1, 2.723(7); Ir1–HA, 1.45(6); Ir1–HB, 1.50(4); Ir1–HC, 1.73(6); Ir1–P1, 2.286(2); Ir1–P2, 2.280(2); Ir1–N1, 2.203(4); B1–N2, 1.61(1); P1–Ir1–P2, 165.38(6); HA–Ir1–HC, 164(3); Ir1–HC–B1, 134(4).

While decomposition on removal of a H_2_ atmosphere makes **[1-H_4_][BAr^F^_4_]** less suitable to use as a practical and weighable precatalyst, it is an intermediate in the formation of a more tractable complex, the amine-borane adduct [Ir(^*i*^Pr-PN^H^P)(H)_2_(H_3_B·NMe_3_)][BAr^F^_4_], **[1-H_3_B·NMe_3_][BAr^F^_4_]**. The tertiary amine-borane H_3_B·NMe_3_ was chosen to prevent unwanted onward dehydrocoupling from N–H activation. Addition of 1 equivalent of H_3_B·NMe_3_ to a solution of **[1-H_4_][BAr^F^_4_]** results in the rapid (time of mixing) and quantitative conversion to **[1-H_3_B·NMe_3_][BAr^F^_4_]** and the release of H_2_ (δ_H_ 4.5). On a preparative scale, addition of H_3_B·NMe_3_ to **[1-COD][BAr^F^_4_]** under a hydrogen atmosphere (2 bar, 20 h, unoptimized) allows for the quantitative formation of **[1-H_3_B·NMe_3_][BAr^F^_4_]**, which was isolated in good (76%) yield as analytically pure colorless crystals. The solid-state structure, as determined by single-crystal X-ray diffraction, is presented in Figure 2. This shows a *cis*-dihydride and *mer*-κ^3^-^*i*^Pr-PN^H^P ligand arrangement around the metal center. Hydrogen atoms associated with the borane, amine and iridium-hydrides were located in the final difference map. These structural data show an η^1^-bound H_3_B·NMe_3_, as indicated by a long Ir···B distance [2.723(7) Å] and open Ir–H–B angle [Ir1–HC–B1 134(4)°].^75−77^ There is one B–H in a close approach to the Ir center [Ir1–HC 1.73(6) Å]. The borane is situated *syn* to the N–H group with the N–H proton bifurcating the two B–H groups [HD and HE]. A noncovalent interaction plot (see Figure S70) suggests the presence of some weak B–H···H–N interaction although the H···H distances are longer than those of conventional dihydrogen bonds (>2.29 Å).^78^

In the ^1^H NMR spectrum at 298 K (CD_2_Cl_2_), the borane is observed as a broad resonance at δ −2.18, relative integral 3H. Cooling to 183 K results in this peak resolving into two signals at δ 1.87 (br) and δ −11.07 (1 H), corresponding to terminal and bridging B–H and Ir···H–B environments, respectively. At 298 K, the Ir-hydride resonances are observed at δ −20.24 and δ −22.74, as triplets of doublets, which do not change significantly in their chemical shift or coupling patterns on cooling. Overall, these data indicate that a rapid exchange^79^ between the bridging and terminal B–H sites occurs at room temperature on the NMR timescale that retains the relative stereochemistry of the complex. At 298 K, the N–H group is observed at δ 3.00, only slightly upfield shifted from **[1-COD][BAr^F^_4_]** [δ 3.47] consistent with no significant hydrogen bonding with the borane.^80^

**Scheme 7:**
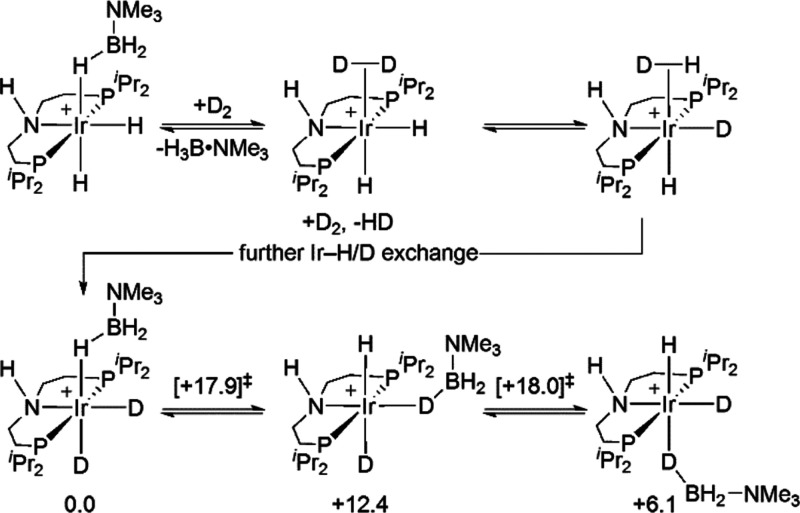
Proposed Pathway for Observed H/D Exchange in [1-H_3_B·NMe_3_][BAr^F^_4_] [BAr^F^_4_]^−^ anions are not shown. DFT-computed free energies (kcal/mol) for key stationary points along the B-H(D) exchange pathway are also indicated. The full pathway involves four transition states linking three intermediates, and only the highest lying transition state energies are shown (see Figure S63 for full details). Level of theory: BP86[D3BJ,CH_2_Cl_2_]/Def2TZVP//BP86/SDD (Ir, P, with polarization on P); 6-31G** on all other atoms.

Addition of H_2_ (2 bar, CD_2_Cl_2_ solution) to a sample of **[1-H_3_B·NMe_3_][BAr^F^_4_]** results in the displacement of the σ-amine-borane ligand by dihydrogen, generating **[1-H_4_][BAr^F^_4_]** in situ, with these two complexes observed in an 8:2 ratio, respectively. Subsequent degassing of the sample results in the quantitative reformation of **[1-H_3_B·NMe_3_][BAr^F^_4_]**, indicating that an equilibrium operates. When **[1-H_3_B·NMe_3_][BAr^F^_4_]** is exposed to D_2_ (1 bar, 60 min), followed by degassing, deuterium incorporation is observed in the ^2^H NMR spectrum at both the hydride positions and the borane. H_2(dissolved)_ and HD_(dissolved)_ [δ_H_ 4.24, 1:1:1 triplet, *J*(HD) = 43 Hz] are also observed in the ^1^H NMR spectrum, alongside a reduction in the relative intensity of the hydride and borane signals (∼95% when compared to ^*i*^Pr). There is no evidence for H/D exchange at the amine proton, which is observed at δ_H_ 2.97 and retains a relative integral 1H when compared to other ligand resonances. A suggested mechanism for H/D exchange informed by DFT calculations is presented in Scheme 7. Displacement of H_3_B·NMe_3_ with D_2_ results in [Ir(^*i*^Pr-PN^H^P)(H)_2_D_2_][BAr^F^_4_] from which H/D exchange at the Ir-hydrides occurs, as described for **[1-H_4_][BAr^F^_4_]** (Scheme 6). Re-coordination of H_3_B·NMe_3_ followed by H/D exchange between the borane and Ir–D provides access to the B–D isotopologues. Calculations suggest that this occurs by transfer of the {BH_2_·NMe_3_} moiety from *syn* to *anti* to the N-H bond via a σ-H_2_DB·NMe_3_ intermediate at +12.4 kcal/mol. Facile exchange between bridging and terminal B–H(D) bonds and reversing the transfer of the {BHD·NMe_3_} unit to the original position *syn* to the N-H bond complexes the exchange with an overall barrier of 18 kcal/mol. Similar H/D exchange processes have been experimentally and computationally studied for [Ir(PCy_3_)_2_(H)_2_(H_3_B·NMe_3_)][BAr^F^_4_].^79^ While the observation of H/D exchange suggests that B–H bond activation at the Ir center may play an important role in the overall mechanism of dehydrogenation, ultimately we show this to proceed by N–*H* transfer to a hydride ligand trans to the NH ligand that then induces B–H cleavage and aminoborane loss (vide infra).^81^

### 2.6 Dehydropolymerization of H_3_B·NMeH_2_

Initial screening of the cationic ^*i*^Pr-PN^H^P-containing complexes **[1-COD][BAr^F^_4_]**, **[1-H_3_B·NMe_3_][BAr^F^_4_]**, and in situ prepared **[1-H_4_][BAr^F^_4_]** for the dehydropolymerization of methylamine-borane, H_3_B·NMeH_2_, was carried out at 1 mol % catalyst loading in 1,2-F_2_C_6_H_4_ solution (0.223 M H_3_B·NMeH_2_). Precatalyst **[1-COD][BAr^F^_4_]** showed no activity, in contrast to the analogous Rh system, where [Rh(κ^3^-{(CH_2_CH_2_P^*i*^Pr_2_)_2_NH}(NBD)]Cl was observed to be an excellent precatalyst, albeit after an induction period to form the active catalyst Rh(^*i*^Pr-PN^H^P)H_3_.^52^ Both **[1-H_3_B·NMe_3_][BAr^F^_4_]** and in situ prepared **[1-H_4_][BAr^F^_4_]** are competent, but rather slow, precatalysts (Table 1), promoting quantitative conversion to poly(*N*-methylaminoborane) (H_2_BNMeH)_*n*_ after 6 h. The polymer was formed selectively, however, with only trace *N*-trimethylborazine or other BN products formed, as shown by a broad signal observed in the ^11^B NMR spectrum at δ −6.1, alongside a sharp signal at δ −6.6 for [BAr^F^_4_]^−^ in the crude, unprecipitated, polymer/catalyst mixture, cf. Figure 2. Analysis of the precipitated polymer by GPC (polystyrene standards, refractive index detector) showed very similar molecular weights for polymer produced by both catalysts (*M*_n_ = 35,100 g/mol and 39,200 g/mol, respectively) with relatively narrow dispersity (*Đ* = 1.7). Interestingly, these are significantly longer than those measured for the **1-H_3_**/[NMeH_3_][BAr^F^_4_] catalysis (*M*_n_ = 18,700 g/mol), which supports the role of ammonium/boronium as chain control agents in dehydropolymerization.^27,52^ The GPC analysis also showed an additional, sharper, signal that has previously been identified as being due to entrained [BAr^F^_4_]^−^ in the polymer sample, likely as the [NMeH_3_]^+^ or [H_2_B(NMeH_2_)_2_]^+^ salt.^25^ The [BAr^F^_4_]^−^ anion coelutes with the polymers, complicating the analysis of the molecular weight distribution, meaning that degrees of polymerization should be considered as approximate only. Signals due to the residual ^*i*^Pr-PN^H^P ligand are also observed by ^1^H NMR spectroscopy, suggesting that the catalyst is coprecipitated with the polymer.

**Table 1:** Dehydropolymerization of H_3_B·NMeH_2_ Using Different Cationic Precursors^a^

catalyst	time/h	*M*_n_^b^/(g/mol)	conv./%^c^ (sel./%)	yield/%
[1-COD][BAr^F^_4_]	16		0	
[1-H_4_][BAr^F^_4_]^d^	5	35,100	99 (99)	79
[1-H_3_B·NMe_3_][BAr^F^_4_]	6	39,200	100 (97)	55
[1-H_3_B·NMe_3_][BAr^F^_4_]^d^	6	45,100	99 (98)	60
[1-H_3_B·NMe_3_][BAr^F^_4_]^e^	6	4800	87 (73)	23
[1-H_3_B·NMe_3_][BAr^F^_4_]^f^	2	g	30^h^	

a Experimental conditions: 1 mol % cat., 0.223 M H_3_B·NMeH_2_ in 1,2-F_2_C_6_H_4_ under a flow of Ar.

b GPC analysis compared to polystyrene standards. *Đ* for all samples measured between 1.5 and 1.7.

c Conversion by ^11^B NMR spectroscopy (selectivity).

d Eudiometric conditions, jacketed-Schlenk flask at 293 K.

e Sealed ampule.

f In THF under eudiometric conditions.

g Low conversion to short-chain oligomers.

h Estimated from H_2_ evolved.

Using **[1-H_3_B·NMe_3_][BAr^F^_4_]** and performing the reaction under a flow of argon to promote the removal of H_2_, or under eudiometric conditions, resulted in the generation of polyaminoborane (*M*_n_ ∼ 40,000 g mol^–1^, *Đ* ∼ 1.6), with high conversion and selectivity (97%). In contrast, performing the reaction in a sealed ampule where hydrogen pressure is allowed to build up resulted in an intractable mixture of short-chain and cyclic products, for example, *N*-methylborazine, incomplete conversion (87%) and polyaminoborane with a significantly lower molecular weight (*M*_n_ ∼ 5000 g/mol) and high dispersity (*Đ* = 2.2). This, again, demonstrates the inhibitory effect that H_2_ has on dehydropolymerization in some catalyst systems.^27−29^ Here, we suggest the equilibrium that was established to occur between **[1-H_4_][BAr^F^_4_]** and **[1-H_3_B·NMe_3_][BAr^F^_4_]** models the conditions of catalysis using H_3_B·NMeH_2_ under conditions that allow for build-up of H_2_. This will slow the dehydrogenation to form the actual monomer, H_2_B=NMeH, and thus the rate of propagation. If any chain termination/transfer is proportionally less unaffected by H_2_, then the resulting degree of polymerization will be lower under closed conditions.

During the course of these dehydropolymerization reactions performed in 1,2-difluorobenzene, it was noted that the solution became visibly very turbid after ca. 10 min, as a result of polymer precipitation from solution. Qualitatively (H_2_BNMeH)_*n*_ is more soluble in THF than 1,2-F_2_C_6_H_4_;^82^ as such, the reaction was also performed in THF—a solvent that has worked well for neutral catalyst systems.^52^ However, the reaction was relatively sluggish and only reached ∼30% conversion before activity ceased. Investigation of the mixture postcatalysis shows the formation of the inactive trihydride, **1-H_3_**.^83^ An alternative solvent toluene has been used previously in dehydropolymerization reactions, but as H_3_B·NMeH_2_ has a limited solubility, this potentially complicates any kinetic analysis.^18^ All subsequent studies were thus carried out in 1,2-F_2_C_6_H_4_ solvent, where complete conversion of H_3_B·NMeH_2_ occurs.

### 2.7 Kinetics of H_3_B·NMeH_2_ Dehydrogenation

**Figure 3:**
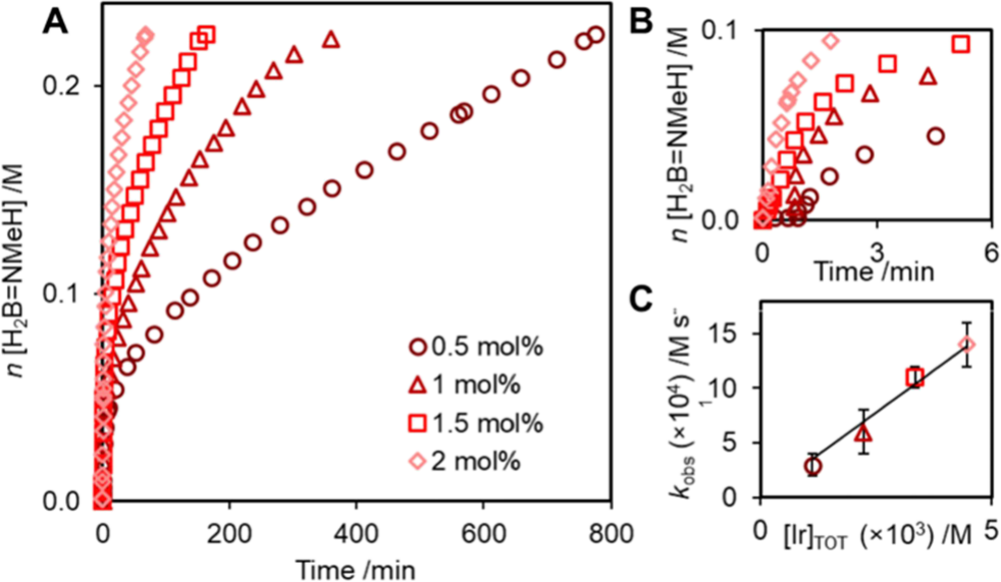
(A) Kinetics of dehydropolymerization of H_3_B·NMeH_2_ using **[1-H_3_B·NMe_3_][BAr^F^_4_]** at 0.5–2 mol %. H_2_B=NMeH equivalents formed from H_2_ evolution (eudiometer). Experimental conditions: 50 mg H_3_B·NMeH_2_, 0.223 M in 1,2-F_2_C_6_H_4_ (5 mL), jacketed-Schlenk flask at 293 K. (B) Early stages of catalysis. (C) First-order relationship of *k*_obs_ with [Ir]_TOT_ at the very early stages of catalysis.

Temporal profiles for the dehydropolymerization of H_3_B·NMeH_2_ (0.223 M) mediated by **[1-H_3_B·NMe_3_][BAr^F^_4_]** in 1,2-F_2_C_6_H_4_ solvent were obtained from hydrogen evolution measurements at various catalyst loadings (0.5–2 mol %) and are shown in Figure 3. The application of simple integrated rate laws or the VTNA methodology^84^ showed that catalysis did not follow a simple rate law (Supporting Materials). Moreover, visual inspection of the temporal profiles indicated a marked deceleration with time, which appears to be more pronounced at lower loadings (i.e., 0.5 mol %). Given the precipitation of the polymer from solution noted above, and the changes in speciation throughout catalysis (vide infra), kinetic analysis was performed through application of initial rates for the very early stages of catalysis where [H_3_B·NMeH_2_] is high and the corresponding concentration of (H_2_BNMeH)_*n*_ is low.

Comparison of initial rates measured as catalyst loading is varied between 0.5 and 2 mol % returns a first-order relationship in [Ir]_TOT_. In each case, there is a short induction period of up to 60 s that broadly scales inversely with catalyst loading. We have previously noted similar induction periods in dehydropolymerization using cationic [Rh(Xantphos-^*i*^Pr)(H)_2_(H_3_B·NMeH_2_)][BAr^F^_4_] catalysts, and suggested trace impurities in the 1,2-F_2_C_6_H_4_ solvent act to modify the catalyst systems before productive turnover starts.^25^ While the initial rate measurements were qualitatively repeatable, there was also some variation between repeat runs, which may be due to trace solvent impurities or mass transfer effects due to polymer precipitation. For this reason, isotope labeling experiments were not carried out.

Polyaminoborane is produced selectively (>95%) in all cases, with close to quantitative conversion as determined by sampling of the reaction using ^11^B NMR spectroscopy (Table 2). While the molecular weight of the resulting polymer appears to follow an inverse relationship with catalyst loading, as described for other cationic systems,^28,29^ accurate measurement of the *M*_n_ is hindered by the overlapping signal from the presence of [BAr^F^_4_]^−^ in the GPC trace. These data are in contrast with those obtained using the neutral Rh(^*i*^Pr-PN^H^P)H_2_Cl catalyst which shows well-behaved zero-order kinetics for H_2_ evolution, in THF solvent, and straightforward GPC analysis in the absence of [BAr^F^_4_]^−^ that shows that *M*_n_ is invariant to catalyst loading.

**Table 2:** Initial Rates for H_2_ Evolution and Polymer Characterization Using [1-H_3_B·NMe_3_][BAr^F^_4_]^a^

[Ir]_TOT_/mol%	*k*_obs_/(×10^–4^ M/s)	*M*_n_^b^/(g/mol)	*Đ*^b^	% conversion^c^ (% yield)
0.5	3(1)	37,300	1.6	97(55)
1.0	6(2)	39,900	1.6	99(60)
1.5	11(1)	11,600	1.7	99(60)
2.0	14(2)	9,100	1.7	94(48)

a 0.223 M H_3_B·NMeH_2_ (50 mg), jacketed-Schlenk flask at 293 K, 1,2-F_2_C_6_H_4_ (5 mL) using **[1-H_3_B·NMe_3_][BAr^F^_4_]**.

b Average of two runs measured by GPC relative to polystyrene standards.

c Measured using ^11^B NMR spectroscopy by sampling the reaction mixture (% yield of the isolated polymer).

### 2.8 In Situ Speciation Studies

**Figure 4:**
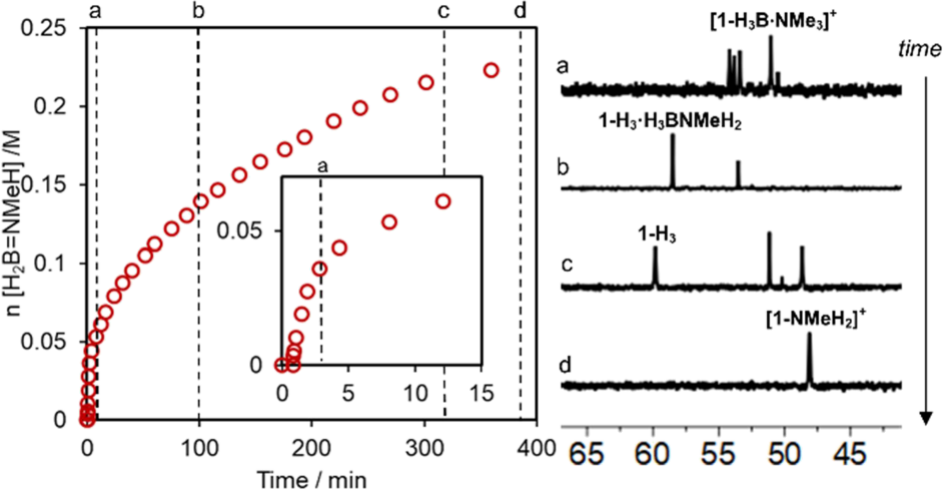
Temporal plot of dehydropolymerization reaction progression at 293 K (first 15 min highlighted in the inset) and corresponding ^31^P{^1^H} NMR spectra at position indicated by the dashed line recorded at 245 K: *a* = 2.5 min, *b* = 1.5 h, *c* = 5.1 h, *d* = 6.3 h. Experimental conditions: 0.223 M H_3_B·NMeH_2_ in 1,2-F_2_C_6_H_4_, 1.0 mol % **[1·H_3_B·NMe_3_][BAr^F^_4_]**.

Dehydropolymerization experiments were performed in a system open to the release of H_2_ at 293 K, with samples taken periodically that were then analyzed using ^1^H and ^31^P{^1^H} NMR spectroscopy. Measurements were carried out at 245 K (m.p. 1,2-F_2_C_6_H_4_ 239 K)^85^ to halt onward reactivity. A catalyst loading of 1 mol % allowed for reasonable spectra to be acquired that allowed for catalyst speciation to be investigated qualitatively. Figure 4 shows the time-course plot for H_2_ evolution and ^31^P{^1^H} NMR spectra at various time points. At the onset of catalysis in the first 2.5 min, there are multiple species observed by ^31^P{^1^H} NMR spectroscopy that lie within the range of 55–48 ppm, similar to **[1-H_3_B·NMe_3_][BAr^F^_4_]** (δ 51.1). The hydride region of the ^1^H NMR spectrum also shows multiple species (δ −20.1 to −22.4), at similar chemical shifts to those reported for [Ir(PCy_3_)_2_H_2_(H(BH_2_NH_2_)_*n*_H][BAr^F^_4_] (*n* = 1 to 5) during the dehydropolymerization of H_3_B·NH_3_.^43^ A small amount of **[1-H_4_][BAr^F^_4_]** is also observed (δ 53.5). A more quantitative assessment of the absolute concentrations of Ir-containing species, and thus [Ir]_TOT_, was hampered by the overall low concentrations of each species, broad lower intensity peaks, and attendant low signal to noise, especially at the earlier stages of catalysis.

After 10 min, a new signal at δ 58.7 is observed which grows in as the reaction progresses and turnover slows. After 1.5 h (∼60% conversion), the ^31^P{^1^H} NMR spectrum simplifies to show this as the major species, the appearance of which tracks the appearance of three new hydride resonances in the ^1^H NMR spectrum [δ −11.60, −12.93, and −22.67]. These are observed as broad multiplets, which collapse to broad singlets upon ^31^P decoupling. These data are similar to those of the previously reported^53^*neutral* trihydride complex Ir(^*i*^Pr-PN^H^P)H_3_**1-H_3_** [δ_P_ = 61.5, δ_H_ = −11.47, −12.26, −22.25 in 1,2-F_2_C_4_H_6_]. After ∼5 h (∼95% conversion), when [H_3_B·NMeH_2_] is very low, these signals shift slightly [δ_P_ = 59.8, δ_H_ = −11.61, −12.65, −22.63]. This change likely reports on the formation of an outer-sphere adduct at higher [H_3_B·NMeH_2_], that is, Ir(^*i*^Pr-PN^H^P)H_3_·H_3_B·NMeH_2_, as shown by DFT calculations (vide infra) and is related to that observed in the analogous Rh system.^52^**[1-H_3_B·NMe_3_][BAr^F^_4_]** is also observed at this point, arising from coordination of the H_3_B·NMe_3_ released at the start of catalysis. At the end of productive catalysis (ca. 6 h), there is only one Ir-containing species observed by ^31^P{^1^H} NMR spectroscopy, δ 48.0. The appearance of this signal tracks with two distinct hydride resonances at δ −20.17 and −24.14 in the corresponding ^1^H NMR spectrum. This new species is identified as the cationic amine adduct [Ir(^*i*^Pr-PN^H^P)(H)_2_(NMeH_2_)][BAr^F^_4_], **[1-NMeH_2_][BAr^F^_4_]**, by its independent synthesis as a crystalline solid, from addition of excess NMeH_2_ to **[1-H_3_B·NMe_3_][BAr^F^_4_]** and subsequent recrystallization. NMeH_2_ comes from B–N bond cleavage in H_3_B·NMeH_2_, as has been commented on before.^27,48,49,52,86,87^

To summarize these in situ studies, at the early stages of catalysis a complex mixture is observed, likely [Ir(^*i*^Pr-PN^H^P)(H)_2_(L)][BAr^F^_4_] adducts. As catalysis proceeds neutral **1-H_3_** becomes dominant, to finally be replaced at the end of catalysis by the cationic amine adduct **[1-NMeH_2_][BAr^F^_4_]**. As **1-H_3_** does not catalyze dehydrogenation, we interpret the significant slowing in turnover as the reaction proceeds to be due to its build-up. In the next section, the relationship between these observed species is explored in more detail.

### 2.9 Relationship between Neutral and Cationic Species Observed during Catalysis and the Role of NMeH_2_ and Proton Sources [NMeH_3_][BAr^F^_4_]/[H_2_B(NMeH_2_)_2_][BAr^F^_4_]

**Scheme 8:**
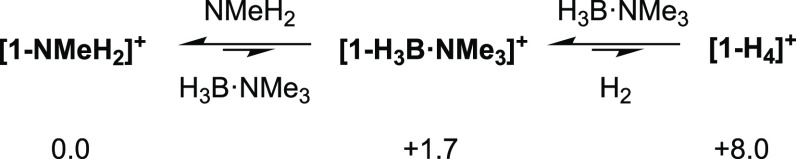
Selected Equilibria Associated with [1-H_3_B·NMe_3_]^+^ [BAr^F^_4_]^−^ anions not shown. Computed free energies (kcal/mol).

As in the catalytic system NMeH_2_ likely comes from slow B–N bond cleavage in H_3_B·NMeH_2_, understanding the role that it plays in speciation is important. Addition of an excess (9 equiv. to [Ir]_TOT_) of H_3_B·NMe_3_ to **[1-NMeH_2_][BAr^F^_4_]** establishes a mixture of **[1-H_3_B·NMe_3_][BAr^F^_4_]**:**[1-NMeH_2_][BAr^F^_4_]** in a ratio 0.05:0.95 showing that NMeH_2_ binds competitively over H_3_B·NMe_3_, but that the amine-borane adduct is still accessible. As, under the conditions used, we have shown that H_2_ binds less competitively than H_3_B·NMe_3_ (Section 2.4) this qualitatively establishes the equilibria shown in Scheme 8. Consistent with this, addition of H_2_ to **[1-NMeH_2_][BAr^F^_4_]** does not form detectable quantities of **[1-H_4_][BAr^F^_4_]** by ^1^H NMR spectroscopy. The equilibrium between **[1-H_3_B·NMe_3_][BAr^F^_4_]** and [**1-NMeH_2_][BAr^F^_4_]** that is biased toward amine-coordination suggests that **[1-NMeH_2_][BAr^F^_4_]** would be a slow catalyst. However, as **[1-NMeH_2_][BAr^F^_4_]** is only observed at the very end of catalysis it is unlikely that it influences the early stages of catalysis significantly. DFT-computed free energies of these three species confirm their relative stabilities (Scheme 8). Finally, addition of [H_2_B(NMeH_2_)_2_][BAr^F^_4_], or [NMeH_3_][BAr^F^_4_], to **1-H_3_** forms **[1-NMeH_2_][BAr^F^_4_]**, consistent with the initial catalytic studies reported (Section 2.2).

**Scheme 9:**
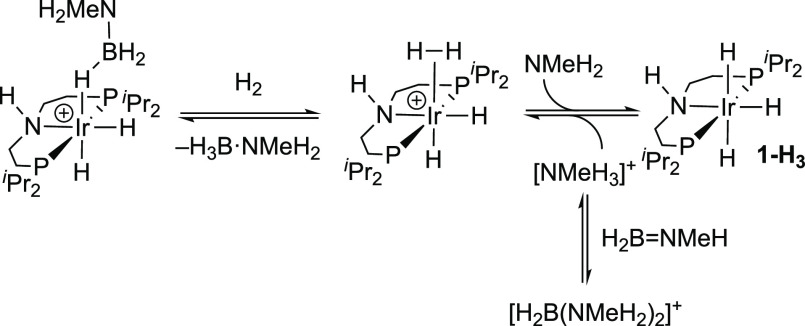
Proposed Mechanism for the Formation of **1-H_3_** and Its Reactivity with [H_2_B(NMeH_2_)_2_][BAr^F^_4_]

Calculations suggest that the generation of neutral **1-H_3_** during catalysis likely occurs by initial formation of dihydrogen/dihydride **[1-H_4_]^+^** followed by deprotonation^88^ by NMeH_2_, Scheme 9. This entails an overall barrier of 17.9 kcal/mol and forms **1-H_3_** and [NMeH_3_]^+^ at +12.8 kcal/mol. We propose that [NMeH_3_]^+^ is then trapped by H_2_B=NMeH (from the main catalytic cycle) to form [H_2_B(NMeH_2_)_2_]^+^, as this renders the formation of **1-H_3_** more thermodynamically reasonable (Δ*G* = +3.5 kcal/mol).^89^ Moreover, the formation of the outer-sphere adduct **1-H_3_·H_3_B·NMeH_2_**, as proposed from the speciation studies and DFT calculations, makes formation even more favorable (Δ*G* = +1.8 kcal/mol). An alternative base-promoted hydride transfer process (cf. **C**, Scheme 2) proved inaccessible,^90^ in contrast to related cationic σ-amine-borane complexes where such processes can occur with relatively low barriers.^48,49,91^ The important consequence of this is that **1-H_3_** must sit off-cycle, as it is not generated from coordination of H_3_B·NMeH_2_. As NMeH_2_ would be expected to build as catalysis proceeds, from B–N bond cleavage, **1-H_3_** does not form immediately, only being observed during the slower phase of catalysis, after ∼10 min.

These stoichiometric observations are supported by catalytic experiments using different precatalysts. Initial rates were used to investigate the relative rate of turnover during the early stages of catalysis. Table 3 reports the results of these experiments. Within error, both **[1-H_3_B·NMe_3_][BAr^F^_4_]** and **[1-H_4_][BAr^F^_4_]** (entries 1 and 2) operate at similar initial rates, consistent with the displacement of H_2_ by H_3_B·NMeH_2_. They also produce polymers of comparable molecular weight (∼40,000 g mol^–1^). **[1-NMeH_2_][BAr^F^_4_]** promotes significantly slower turnover (∼7 times slower), entry 3, likely due to NMeH_2_ binding competitively with amine-borane from the start of catalysis, as well as promoting the formation of **1-H_3_**. Essentially, the same data are obtained if NMeH_2_ is added to **[1-H_3_B·NMe_3_][BAr^F^_4_]** (entry 4). Addition of two equivalents of [NMeH_3_][BAr^F^_4_], entry 5, results in isolated polymers of considerably lower molecular weight, consistent with the role of [NMeH_3_]^+^ as a chain control agent.

**Table 3:** Initial Rates for H_2_ Evolution from Initial Rate Measurements and Polymer Characterization^a^

entry	catalyst	*k*_obs_ (×10^–4^ M/s)	*M*_n_^b^ (g/mol)	% conversion.^c^ (% yield)
1	[1-H_3_B·NMe_3_]^+^	6(2)	39,900	99(60)
2	[1-H_4_]^+^^d^	7.1(8)	35,100	99(79)
3	[1-NMeH_2_]^+^	0.9(1)	49,300	99(54)
4	[1-H_3_B·NMe_3_]^+^/NMeH_2_^e^	0.9(1)	48,500	84(48)
5	[1-H_3_B·NMe_3_]^+^/[NMeH_3_]^+^^f^	8.1(3)	15,800	97(54)
6	[1-H_3_B·NMe]^+^/recharged^g^	0.5(2)	41,600	97^g^

a 1 mol % cat., 0.223 M H_3_B·NMeH_2_ (50 mg), jacketed-Schlenk flask at 293 K, 1,2-F_2_C_6_H_4_ (5 mL).

b GPC relative to polystyrene standards.

c Measured using ^11^B NMR spectroscopy by sampling the reaction mixture, % yield of the isolated polymer.

d **1-H_3_** made in situ.

e 2 equiv. additive to [Ir].

f 2 equiv. relative to [Ir]. *Đ* is between 1.5 and 1.8 for all samples.

g Recharged with 50 mg of H_3_B·NMeH_2_; final isolated yield not determined.

Chain control is suggested to occur by protonation of the polymeryl amine end group.^27^ In support of this, added amine (entries 3 and 4) produces polymer of higher molecular weight. We have previously noted similar effects of added amine in related systems.^28^ Initial rate measurements suggest that turnover may be slightly faster with added ammonium, although within error it is the same as undoped **[1-H_3_B·NMe_3_][BAr^F^_4_]** and **[1-H_4_][BAr^F^_4_]**. Finally, recharging the catalyst mixture with more H_3_B·NMeH_2_ restarts catalysis, but now at a significantly slower rate (entry 6), being closer to that measured for **[1-NMeH_2_]^+^**, consistent with this complex being the final resting state.

### 2.10 Proposed Mechanism of Dehydrogenation

Informed by in situ NMR speciation experiments, H_2_ evolution kinetics, and stoichiometric reactions, a plausible mechanism for the dehydrogenation of H_3_B·NMeH_2_ using **[1-H_3_B·NMe_3_][BAr^F^_4_]** can be suggested. The essential elements of the proposed mechanism capture the following observations:

(i) Neutral, catalytically inactive **1-H_3_** sits off-cycle, and addition of [NMeH_3_][BAr^F^_4_] brings it into the productive, cationic, manifold. **1-H_3_** is formed by deprotonation of **[1-H_4_][BAr^F^_4_]**, rather than a base-promoted hydride transfer from a coordinated amine-borane complex.

(ii) The relative ratios of observed resting states, and thus rate of turnover, will reflect the relative proportions of H_3_B·NMeH_2_ (decreasing with time), dissolved H_2_, NMeH_2_ (formed through B–N bond cleavage), and [H_2_B(NMeH_2_)_2_]^+^ or [NMeH_3_]^+^ (coproducts of the formation of **1-H_3_**).

**Scheme 10:**
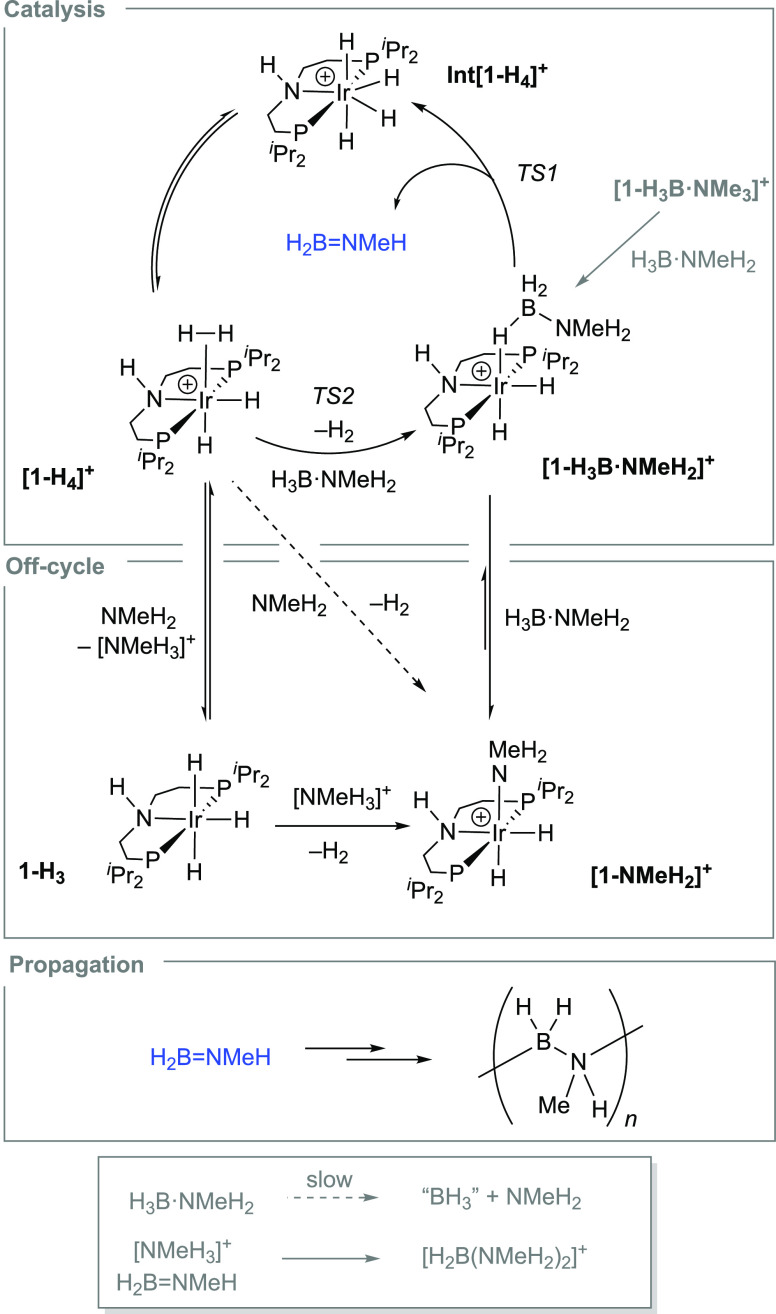
Proposed Catalytic Cycle and Off-Cycle Processes, for Dehydropolymerization of H_3_B·NMeH_2_ [BAr^F^_4_]^−^ anions and outer-sphere H_3_B·NMeH_2_ (see text) not shown.

Scheme 10 shows the proposed mechanism that is also informed by DFT calculations (see below). Starting from **[1-H_3_B·NMe_3_][BAr^F^_4_]** substitution with H_3_B·NMeH_2_ generates [**1-H_3_B·NMeH_2_][BAr^F^_4_]**. A concerted N-H/B–H activation then leads, via its tetrahydride isomer, to **[1-H_4_][BAr^F^_4_]** with release of H_2_B=NMeH for onward polymerization, as initiated by either Ir-hydride species or free amine.^5,12,27,40,41,52^ Associative H_2_/H_3_B·NMeH_2_ substitution then completes the catalytic cycle. This reaction manifold is modified by the slow formation of NMeH_2_ from B–N bond cleavage^92^ in H_3_B·NMeH_2_. This promotes formation of off-cycle **1-H_3_** via **[1-H_4_]^+^** and also produces [H_2_B(NMeH_2_)_2_][BAr^F^_4_] in the presence of aminoborane, H_2_B=NMeH. Reprotonation by [NMeH_3_]^+^ (or [H_2_B(NMeH_2_)_2_]^+^) forms **[1-NMeH_2_][BAr^F^_4_]**, the rate of which may be further attenuated by the formation of off-cycle adducts with H_3_B·NMeH_2_ (as measured experimentally for H_3_B·NMe_3_/[NMe_2_H_2_]^+^)^49^ or entrainment in precipitated polymers (vide infra). The productive catalytic cycle is returned to from **[1-NMeH_2_][BAr^F^_4_]**, by coordination of H_3_B·NMeH_2_ and replacement of NMeH_2_. Only at the end of catalysis, when H_3_B·NMeH_2_ is consumed, and NMeH_2_ has built to its maximum level does the final resting state shift to the amine adduct, **[1-NMeH_2_][BAr^F^_4_]**.

**Figure 5:**
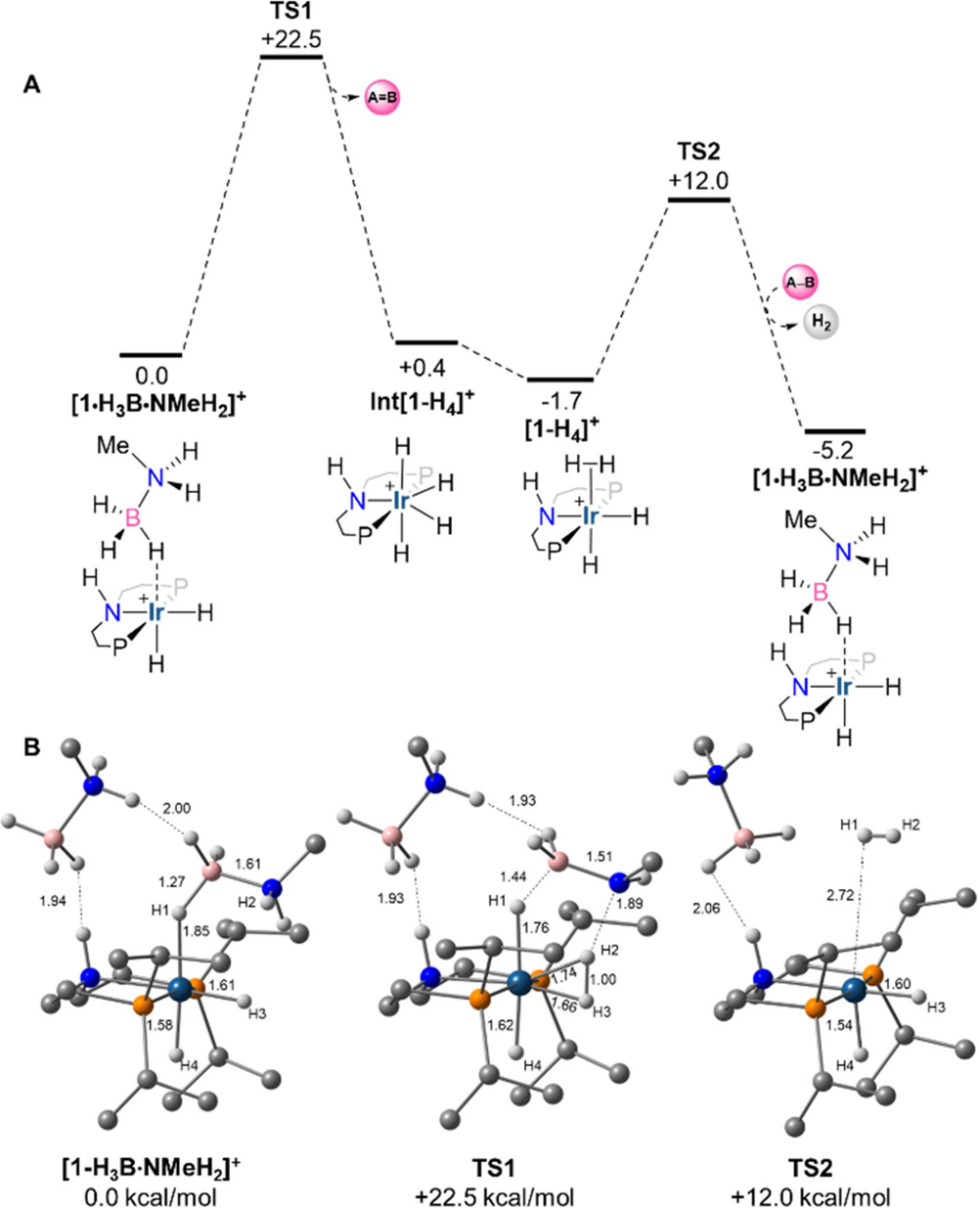
(A) Computed free energy profile (kcal/mol) for amine-borane dehydrogenation from **[1-H_3_B·NMeH_2_]^+^** (P = P^*i*^Pr_2_, **MA** = methylamine, **A–B** = H_3_B·NMeH_2_; **A=B** = H_2_BNMeH; an outer-sphere H_3_B·NMeH_2_ is always present in the computed structures but is omitted from the schematic representations for simplicity). (B) Computed structures of **A** and **TS1** and **TS2** highlighting key distances (Å) and shortest contacts to the outer-sphere H_3_B·NMeH_2_ molecule; C–H hydrogens are omitted for clarity. Key: Ir: blue; H: white; B: pink; C: gray; N: azure; P: orange. Level of theory: BP86[D3BJ,2-hexanone]^93^/Def2TZVP//BP86/SDD (Ir, P, with polarization on P); 6-31G** on all other atoms.

The key steps in this process were modeled by DFT calculations (see Figure 5A). The chemical model adopted for this study includes one outer-sphere H_3_B·NMeH_2_ molecule. This was found to have a significant effect on the overall computed barrier, possibly as it interrupts any intramolecular N–H^δ+^···H^δ−^–B interactions within the [**1-H_3_B·NMeH_2_]^+^** cation. This can be seen in the computed structure of the [**1-H_3_B·NMeH_2_]^+^·H_3_B·NMeH_2_** adduct (Figure 5B) where the Ir-bound amine-borane is tilted away from the N–H of the pincer ligand rather than toward it as in the molecular structure of [**1-H_3_B·NMe_3_]^+^** (Figure 2). This model is also justified by a free energy change of −1.7 kcal/mol for the formation of the adduct from [**1-H_3_B·NMeH_2_]^+^** and free H_3_B·NMeH_2_. The importance of including outer-sphere interactions with the ^*i*^Pr-PN^H^P ligand has recently been pointed out when modeling Ru-catalyzed formic acid dehydrogenation.^57^

A range of mechanisms were assessed for H_3_B·NMeH_2_ dehydrogenation, and the lowest energy process is shown in Figure 5A with computed structures in Figure 5B.^94^ From **[1-H_3_B·NMeH_2_]^+^**, N–H/B–H activation proceeds in a single step via **TS1** at 22.5 kcal/mol. N–H bond cleavage is more advanced in this transition state (N···H2 = 1.89 Å; B···H1 = 1.44 Å), and proton transfer to the metal is assisted by the adjacent hydride forming an η^2^-H_2_ moiety (H2–H3 = 1.00 Å). Throughout the nascent aminoborane maintains N–H^δ+^···H^δ−^–B interactions with the outer-sphere H_3_B·NMeH_2_ molecule. **TS1** leads to the formation of the tetrahydride **Int[1-H_4_]^+^** from which the reductive coupling of H1 and H2 to give **[1-H_4_]^+^** is essentially barrierless (see Scheme 6(70)). H_2_ dissociation from **[1-H_4_]^+^** proceeds via **TS2** at +12.0 kcal/mol and in the presence of the outer-sphere H_3_B·NMeH_2_ reforms **[1-H_3_B·NMeH_2_]^+^** directly to complete the catalytic cycle. The overall barrier for dehydrogenation is 22.5 kcal/mol, with the rate-limiting transition state associated with an asynchronous, concerted N–H/B–H activation step. Without the outer-sphere H_3_B·NMeH_2_ molecule, the computed barrier is 27.5 kcal/mol, similar to previous calculations where N–H activation processes at cationic group 9 amine-boryls report rate-limiting barriers of ∼24–27 kcal/mol.^43,66^ An alternative inner-sphere mechanism featuring stepwise B–H then N–H activation had a slightly higher barrier of 23.6 kcal/mol (see Supporting Materials). The displacement of H_3_B·NMeH_2_ by NMeH_2_ is also computed to be thermodynamically favored (Δ*G* = −2.8 kcal/mol), consistent with the formation of this species toward the end of the catalytic runs and the slowing of catalysis as this species becomes more prevalent.

The off-cycle processes in Scheme 10 imply a competition in the fate of **[1-H_4_]^+^**: deprotonation to form off-cycle **1-H_3_** and [H_2_B(NMeH_2_)_2_]^+^, or H_2_ substitution to reform either **[1-H_3_B·NMeH_2_]^+^** or **[1-NMeH_2_]^+^**. While the latter processes are thermodynamically favored (by 5.7 kcal/mol and 4.8 kcal/mol respectively), the deprotonation is more accessible kinetically, with a barrier (relative to **[1-H_4_]^+^**) of 7.3 kcal/mol cf. 13.7 kcal/mol for H_2_ loss (see Figures 5A and S68 for details).

In contrast to the inner-sphere process characterized here for the cationic system, the computed mechanism for the dehydrogenation of H_3_B·NMeH_2_ by neutral **1-H_3_** proceeds via an outer-sphere pathway analogous to that reported previously for its Rh congener (see Figure S67). This entails an overall barrier of 24.3 kcal/mol, significantly higher than that for Rh (19.7 kcal/mol) consistent with the far greater activity of the latter. Comparison of the computed profiles suggests that the higher barrier for Ir results from the stronger η^2^-H_2_ adduct that is formed prior to rate-limiting H_2_ loss. The barrier associated with **1-H_3_** is also 1.8 kcal/mol higher than that computed for **[1-H_3_B·NMeH_2_]^+^**, consistent with the greater activity of the latter.

### 2.11 Synthesis of [Ir(^*i*^Pr-PN^Me^P)(H)_2_(H_3_B·NMe_3_)][BAr^F^_4_] and Use in Catalysis: Support for the Inner-Sphere Mechanism

**Figure 6:**
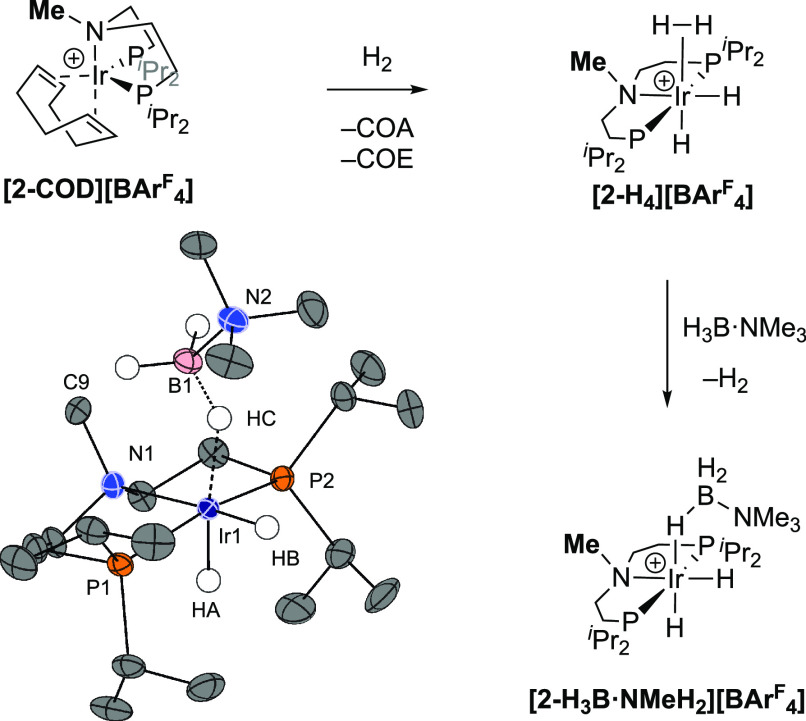
Synthesis of [Ir(^*i*^Pr-PN^Me^P)(H)_2_(H_3_B·NMe_3_)][BAr^F^_4_] and single-crystal X-ray diffraction structure of the cation (50% displacement ellipsoids, see Supporting Materials).

To rule out a ligand cooperative mechanism that involves the N–H group of the ^*i*^Pr-PN^H^P-ligand, the complex [Ir(^*i*^Pr-PN^Me^P)(H)_2_(H_3_B·NMe_3_)][BAr^F^_4_], **[2-H_3_B·NMe_3_][BAr^F^_4_]**, was synthesized in an analogous way to **[1-H_3_B·NMe_3_][BAr^F^_4_]**, Figure 6. Hydrogenation of [Ir(^*i*^Pr-PN^Me^P)(H)_2_(COD)][BAr^F^_4_], **[2-COD][BAr^F^_4_]**, results in the formation of the dihydrogen/dihydride complex **[2-H_4_][BAr^F^_4_]** which displays very similar NMR data to **[1-H_4_][BAr^F^_4_]**, that is, a single hydride resonance at 298 K that integrates to 4H (δ −10.65, *T*_1_ = 242(2) ms), and resolves into two signals at 183 K (δ −4.87, −16.55). Addition of H_3_B·NMe_3_ to in situ formed **[2-H_4_][BAr^F^_4_]** afforded **[2-H_3_B·NMe_3_][BAr^F^_4_]**, which was characterized by NMR spectroscopy and single-crystal X-ray diffraction; with all data being very similar to **[1-H_3_B·NMe_3_][BAr^F^_4_]**. There is a small amount of recalcitrant **[2-H_4_][BAr^F^_4_]** that cocrystallizes with **[2-H_3_B·NMe_3_][BAr^F^_4_]** (∼10% by NMR spectroscopy). As both complexes are likely to offer the same operationally unsaturated^95^ {Ir(^*i*^Pr-PN^Me^P)(H)_2_}^+^ fragment, this mixture was used going forward in catalytic studies.

**Figure 7:**
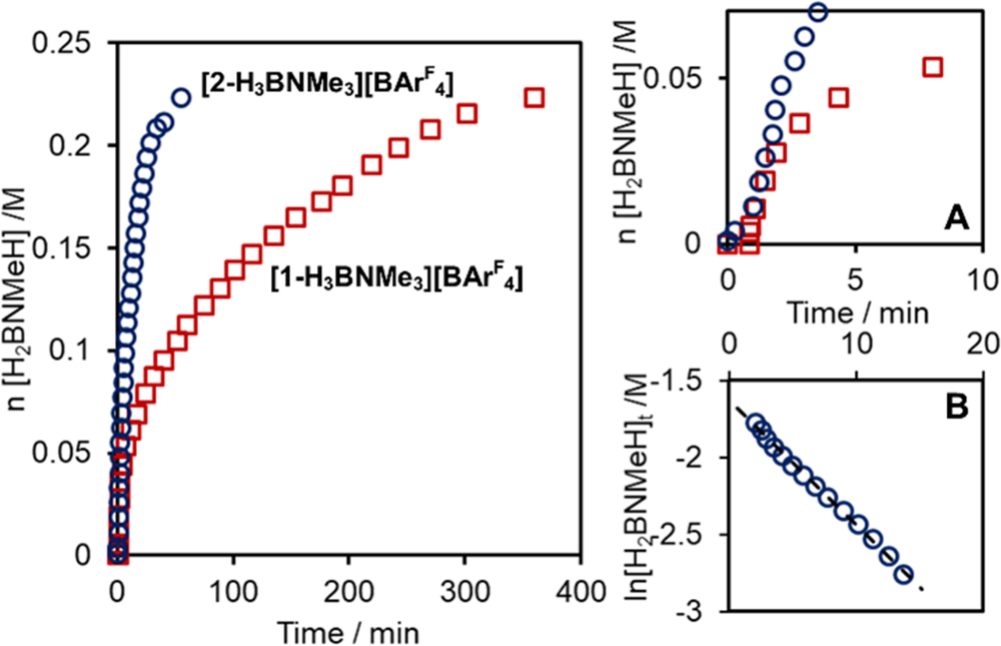
Temporal profiles using the two catalysts and a first-order analysis using catalyst **[2-H_3_B·NMe_3_][BAr^F^_4_]**. H_2_B=NMeH equivalents formed from H_2_ evolution (eudiometer). (A) Expansion showing the early stages of catalysis; (B) First order analysis of H_2_ evolution using **[2-H_3_B·NMe_3_][BAr^F^_4_]**.

When **[2-H_3_B·NMe_3_][BAr^F^_4_]** (1 mol %, 1,2-F_2_C_6_H_4_) was used as a catalyst, there was relatively fast turnover, with one equivalent of H_2_ released in 55 min, in contrast to **[1-H_3_B·NMe_3_][BAr^F^_4_]** (ca. 400 min). This supports an inner-sphere non-ligand-cooperative mechanism. Figure 7 compares the temporal profiles for H_2_ evolution for the two catalysts, while the inset B shows that for **[2-H_3_B·NMe_3_][BAr^F^_4_]** H_2_ evolution occurs with overall pseudo first-order kinetics (*k*_obs_ = 14(2) ×10^–4^ M/s) over ∼2 half-lives—in contrast to the more complex situation for **[1-H_3_B·NMe_3_][BAr^F^_4_]**. However, initial rate measurements show that both catalysts turnover at essentially the same rate [6.4(8) ×10^–4^ M/s]. This shows that the deceleratory processes occurring for **[1-H_3_B·NMe_3_][BAr^F^_4_]** are not occurring for the ^*i*^Pr-PN^Me^P analogue, at least in the early stages of catalysis. Speciation experiments after 10 min turnover show at least three complexes, two of which are spectroscopically identified as [Ir(^*i*^Pr-PN^Me^P)(H)_2_(H_3_B·NMe_3_)][BAr^F^_4_], and the neutral trihydride Ir(^*i*^Pr-PN^Me^P)H_3_, **2-H_3_**.^96^ A further as yet unidentified complex is observed [δ_P_ 59.3; δ_(H-hydride)_ −22.49 and −22.99 ppm]. As the observation of **2-H_3_** demonstrates that deprotonation of **[2-H_4_][BAr^F^_4_]** occurs, why then are the kinetics so different?

### 2.12 Suggested Role of Polymer Precipitation on Observed Reaction Kinetics and Resulting Polymer Products

While we cannot discount that **2-H_3_** is itself a competent catalyst, this is unlikely given the inactivity of **1-H_3_** at 298 K and that Ru(^*i*^Pr-PN^Me^P) complexes are calculated to have significantly higher barriers to amine-borane dehydrogenation than their Ru(^*i*^Pr-PN(*H*)P) analogues.^16^ Instead, we hypothesize that reprotonation of **1-H_3_** by [H_2_B(NMeH_2_)_2_][BAr^F^_4_] or [NMeH_3_][BAr^F^_4_] is attenuated by entrainment of these proton sources in the precipitated polymer, which results in the deceleration observed. If entrainment occurs to a significantly lesser degree for **2-H_3_**, faster turnover would result. Such effects will be amplified by any additional coentrainment of the cationic catalyst in the precipitated polymer that reduces [Ir]_TOT_. Support for this hypothesis comes from a number of observations and experiments.

**Figure 8:**
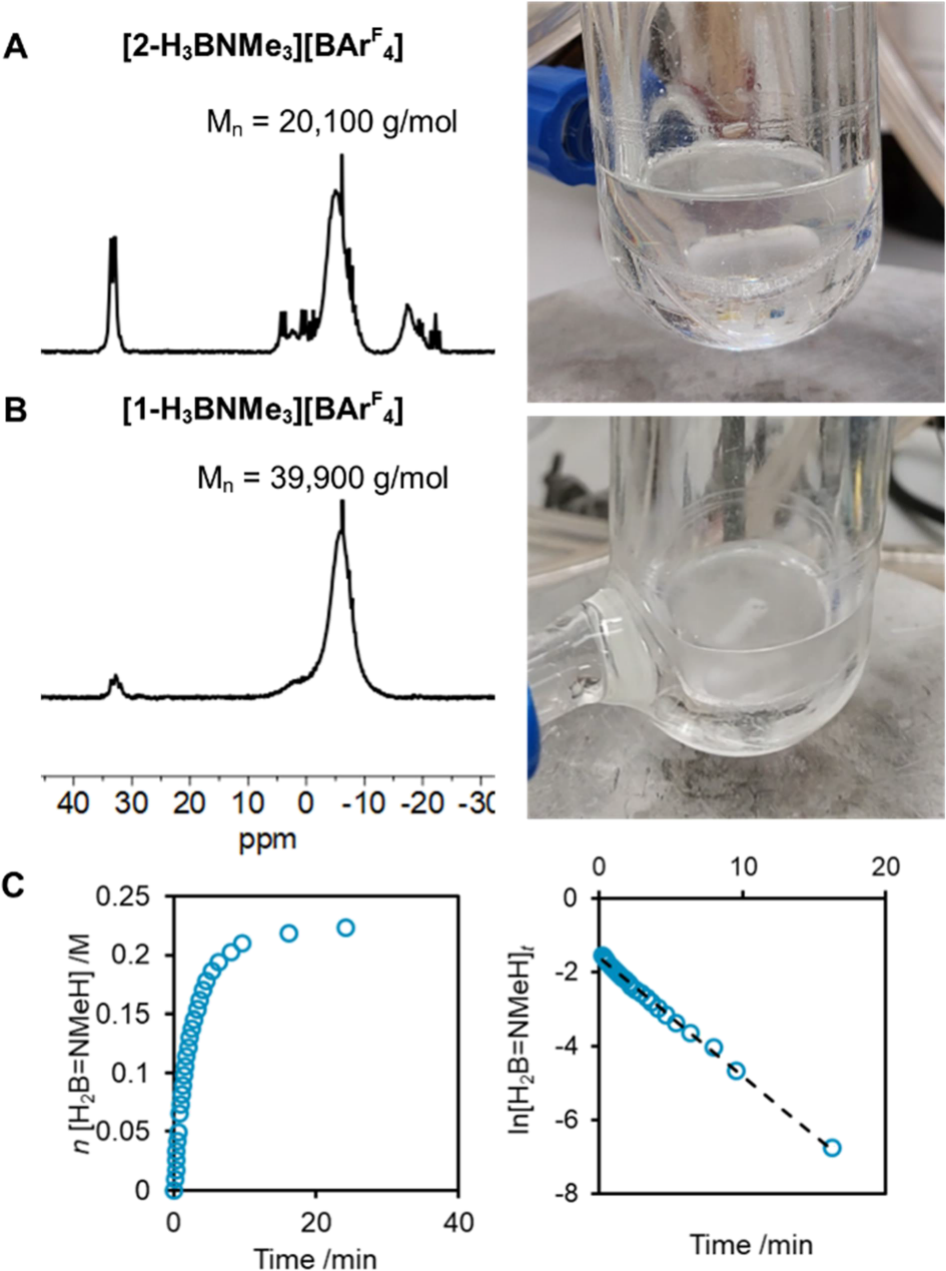
^11^B NMR spectrum and photograph of the final reaction mixture using (A) **[2-H_3_B·NMe_3_][BAr^F^_4_]** and (B) **[1-H_3_B·NMe_3_][BAr^F^_4_]**. (C) Temporal profile and first-order kinetics for **[1-H_3_B·NMe_3_][BAr^F^_4_]** (1 mol %, 10 equiv [NMeH_3_][BAr^F^_4_]). H_2_B=NMeH equivalents from H_2_ evolution (eudiometer).

(i) In comparison to **[1-H_3_B·NMe_3_][BAr^F^_4_]**, catalyst **[2-H_3_B·NMe_3_][BAr^F^_4_]** is less selective, producing shorter, ill-defined, polyaminoboranes and *N*-trismethylborazine, among other B-containing oligomers, as shown by ^11^B NMR spectroscopy (Figure 8A,B) and GPC analysis. These shorter polymers/oligomers are more soluble in 1,2-F_2_C_6_H_4_, and we propose that the [H_2_B(NMeH_2_)_2_][BAr^F^_4_], or [NMeH_3_][BAr^F^_4_], that is formed is thus entrained to a lesser extent. As these also act as chain control agents, the rate of termination now becomes competitive with dehydrogenation/propagation and soluble oligomers of lower molecular weight result. We suggest that the likely more nucleophilic Ir(^*i*^Pr-PN^Me^P)H_3_, **2-H**_**3**,_ may also act as a more efficient initiator^12^ for polymer growth, leading to shorter chains being formed. ^*i*^Pr-PN^Me^P has been shown to be a better N-donor ligand than ^*i*^Pr-PN^H^P on the basis of CO stretching frequencies in Ru-carbonyl complexes.^57,59^

(ii) Adding two equivalents of [NMeH_3_][BAr^F^_4_] to catalysis using **[1-H_3_B·NMe_3_][BAr^F^_4_]** results in a reduction in the degree of polymerization (*M*_n_ = 15,800 g/mol). Although precipitation still occurs, GPC analysis of the isolated polymer shows significantly more [BAr^F^_4_]^−^ is present (Supporting Materials). Notably the time-course plot for H_2_ evolution shows a more pronounced first-order region at the start of catalysis, similar to that for **[2-H_3_B·NMe_3_][BAr^F^_4_]**. In contrast, addition of [NMeH_3_][BAr^F^_4_] to preformed polymer (*M*_n_ = 92,400 g/mol) and work up does not result in an increase in the [BAr^F^_4_]^−^ signal in the resulting GPC chromatogram. Using [BAr^F^_4_]^−^ as a spectroscopic proxy for [NMeH_3_]^+^ thus supports its entrainment during active polymer chain growth.

(iii) Given that [NMeH_3_]^+^, or boronium, acts as a chain control agent and to protonate **1-H_3_**, further increasing its ratio relative to [Ir]_TOT_ should produce even shorter, more soluble oligomers, stop precipitation, and provide simpler kinetics as more catalysts will sit on-cycle. This is the case, and using 10 equivalents results in oligomers (*M*_n_ < 5000 g/mol),^97^ and first-order behavior (*k*_obs_ = 53.7(6) ×10^–4^ M/s) being measured over at least 7 half-lives when using **[1-H_3_B·NMe_3_][BAr^F^_4_] (**1 mol %), Figure 8C. The initial rate measured also showed a significant increase in the rate of turnover, 17.8(1) ×10^–4^ M/s. Speciation using ^31^P{^1^H} NMR spectroscopy at ∼75% conversion (4 min) shows at least five complexes by multiple signals observed in the range δ_P_ of 57–51. We assign these to cationic [Ir(^*i*^Pr-PN^H^P)(H)_2_(L)][BAr^F^_4_] species, being very similar to those observed in the early stages of catalysis using undoped **[1-H_3_B·NMe_3_][BAr^F^_4_]**, cf. Figure 4.

(iv) Finally, entrainment of the [NMeH_3_]^+^/boronium/cationic catalyst may be expected to have a disproportionally greater effect at lower catalyst concentrations, assuming that the degree of polymer precipitation is unchanged. When compared with off-cycle neutral **1-H_3_**, proportionally more of these cationic species would likely be entrained in the precipitated polymer, with the result that turnover slows—as is observed at 0.5 mol % (Figure 3).

## 3 Conclusions

In this contribution, we demonstrate that the mechansim of amine-borane dehydropolymerization continues to offer unexpected and nuanced differences depending on the choice of metal/ligand fragment.^2,3^ While the perceived wisdom would be that neutral precatalysts such as **1-H_3_**, that offer MLC mechanistic pathways, would be effective catalysts for amine-borane dehydrogenation, we show here that they are not fully consistent with Fagnou’s very brief observation nearly 15 years ago on related systems.^62^ That the cationic catalyst manifold offers a lower energy pathway and produces polymer selectively (albeit at 1 mol % catalyst loading) via a non-MLC route was also initially surprising.^25,27,49,52^ Moreover, the role of NMeH_2_, formed from slow B–N bond cleavage, is different from other recently reported systems. Here, it has an inhibitory role, by promoting the formation **1-H_3_**, in contrast to other systems where its role is accelatory by bringing a cationic precatalyst onto the catalytic cycle through promoting hydride transfer to form an active, neutral, catalyst. Finally, and perhaps a more obvious comment, is that significant caution needs to be excercised in interpreting kinetic data when polyaminoborane precipitates from solution, due to the possibility of entrainment of coproducts or catalyst in the catalytic manifold. Moving forward, while the systems under discussion here do not match the efficency of other catalysts in terms of ToF or ToN,^16,19,25,52^ the ability to switch on productive catalytic turnover by simple addition of [NMeH_3_][BAr^F^_4_] to **1-H_3_** offers the possibility for temporally and spatially controlled dehydropolymerization^98^ catalysis that may offer benefits in both H_2_ release profiles and the resulting material properties

## Supporting Information Available

Full details of experimental and characterization data for the new complexes, kinetics data, and computational studies (PDF)
Coordinates of computed intermediates and transition states (XYZ)

## Supplementary Material

cs2c03778_si_001.pdf

cs2c03778_si_003.xyz

## Accession Codes

CCDC 2172743–2172745 and 2183097, 2183098 contain the supplementary crystallographic data for this paper. These data can be obtained free of charge via www.ccdc.cam.ac.uk/data_request/cif, or by emailing data_request@ccdc.cam.ac.uk, or by contacting The Cambridge Crystallographic Data Centre, 12 Union Road, Cambridge CB2 1EZ, UK; fax: +441,223 336,033.
