# Supplementary material for: Dehydropolymerization of H3B·NMeH2 Mediated by Cationic Iridium(III) Precatalysts Bearing κ3-iPr-PNRP Pincer Ligands (R = H, Me): An Unexpected Inner-Sphere Mechanism
Source: ACS Catal. 2022 Oct 12;12(20):13050–64. doi: 10.1021/acscatal.2c03778 (PMC9594342; doi:10.1021/acscatal.2c03778)
Supplement: Supplementary file 1 — cs2c03778_si_001.pdf [file cs2c03778_si_001.pdf]

Electronic Supporting Information

Dehydropolymerisation of $H_3B \cdot NMeH_2$ Mediated by Cationic Iridium(III) Precatalysts Bearing κ^3-^{i}Pr-PN(R)P Pincer Ligands (R = H, Me). An Unexpected Innersphere Mechanism.

Claire N. Brodie,*[a] Lia Sotorrios,[b] Timothy M. Boyd,[a,c] Stuart A. Macgregor*[b] and Andrew S. Weller*[a]

[a] Department of Chemistry, University of York, Heslington, York, Y010 5DD, UK;

[b] Institute of Chemical Sciences, Heriot-Watt University, Edinburgh, EH14 4AS, UK.

[c] Chemistry Research Laboratories, University of Oxford, Oxford, OX1 3TA, UK

* claire.brodie@york.ac.uk

* S.A.Macgregor@hw.ac.uk

* andrew.weller@york.ac.uk

1. Experimental ... 3
1.1 General Procedures ... 3
1.2 Synthesis ... 4
1.3 Catalytic Dehydropolymerisation of $H_3B{\cdot}NMeH_2$ ... 17
1.4 Kinetics of Dehydropolymerisation and GPC traces ... 18
1.4.1 Reaction Order plots ... 22
1.4.2 VTNA ... 23
1.4.3 Effect of Solvent ... 18
1.4.4 Catalyst loading ... 18
1.4.5 Additive Doping ... 20
1.4.6 Alternate Pre-Catalysts ... 21
2. NMR Spectra ... 24
2.1 NMR Spectra for **[1-COD]Cl** ... 24
2.2 NMR Spectra for **[1-COD][BAr$^F_4$]** ... 25
2.3 NMR Spectra for **[1-H$_4$][BAr$^F_4$]** *in situ* ... 26
2.4 NMR Spectra for **[1-H$_3$B·NMe$_3$][BAr$^F_4$]** ... 28
2.5 NMR Spectra for **[1-NMeH$_2$][BAr$^F_4$]** ... 29
2.6 NMR Spectra for **1-H$_3$** *in situ* ... 30
2.7 NMR spectra for solution state reactivity of **[1-L][BAr$^F_4$]** complexes ... 31
2.9 VT NMR for **[1-H$_4$][BAr$^F_4$]** under a pressure of H_2 ... 33
2.10 VT NMR Spectra for **[1-H$_3$B·NMe$_3$][BAr$^F_4$]** ... 34
2.11 *in situ* NMR spectra for the dehydropolymerisaiton of $H_3B{\cdot}NMeH_2$ ... 36
2.12 NMR Spectra for **[2-COD][BAr$^F_4$]** ... 41
2.13 NMR Spectra for **[2-H$_3$B·NMe$_3$][BAr$^F_4$]** ... 42
2.14 NMR Spectra for **[2-H$_4$][BAr$^F_4$]** *in situ* ... 43
2.15 NMR Spectra for **[2-NMeH$_2$][BAr$^F_4$]** *in situ* ... 46
2.16 NMR Spectra for $(H_2BNMeH)_n$ ... 47
4. Crystallographic Information ... 48
5. Computational Information ... 52
6. References ... 100

1. Experimental

1.1 General Procedures

All manipulations, unless otherwise stated, were performed under an argon atmosphere using standard Schlenk line and glove-box techniques. Glassware was oven-dried at 130 °C overnight and flamed under vacuum prior to use. CH_2Cl_2 and pentane were dried using a Grubbs-type solvent purification system (Innovative Technologies SPS) and degassed by three successive freeze-pump-thaw (FPT) cycles. CD_2Cl_2 and 1,2-$C_6H_4F_2$ (pre-treated with alumina) were dried over CaH_2, vacuum distilled, degassed (3 × FPT) and stored over 3 Å molecular sieves. THF (pre-treated over KOH) was distilled from sodium/fluorenone and degassed (3 × FPT) and stored over 3 Å molecular sieves. Isopropanol was dried over CaO, distilled from CaH_2 and treated with 3 Å molecular sieves before use. Na[BAr^F_4],[S1] [Ir(COD)Cl]$_2$,[S2] [Ir(COD)$_2$][BAr^F_4],[S3] [NMe_2H_2][Cl],[S4] [BH_2NMeH]$_2$[S5] and iPr-PN^{Me}P[S6] were prepared by literature methods. $H_3B{\cdot}NMe_3$ and $H_3B{\cdot}NMeH_2$ were recrystallised from Et_2O. iPr-PN^{H}P (10% wt. solution in THF) was degassed and stored over 3 Å molecular sieves.

NMR spectra were recorded on a Bruker Avance III 500 MHz NMR spectrometer or a Bruker Ultrashield 600 MHz NMR spectrometer at 298 K unless otherwise specified. Residual protio solvent was used as reference for 1H spectra in deuterated solvent samples. $^{31}P\{^1H\}$ NMR spectra were externally referenced to 85% H_3PO_4. ^1H assignments were aided by $^1H\{^{31}P\}$ experiments. All chemical shifts (δ) are quoted in ppm and coupling constants (*J*) in Hz. NMR assignments were aided by 2D spectra (^1H,^1H-COSY, ^1H,^{13}C-HSQC, ^1H,^{13}C-HMBC, ^1H,^1H-NOESY) where required. Elemental microanalyses were performed by Graeme McAllister at the University of York. Gel permeation chromatography (GPC) was performed on a Malvern Viscotek GPCmax chromatograph fitted with a refractive index (RI) detector. The triple-column (plus guard column) setup was contained within an oven (35 °C) and consisted of a porous styrene divinylbenzene copolymer with a maximum pore size of 1,500 Å. THF containing 0.1% w/w [NBu_4]Br was used as the eluent at a flow rate of 1.0 mL min^{-1} . Samples for GPC anaylsis were prepared fresh and run immediately by dissolution in the eluent (2.0 mg mL^{-1}) and filtration (0.2 µm pore size). The calibration was conducted using a series of monodisperse polystyrene standards (M_n = 474 – 476,800 g mol^{-1}) obtained from Sigma–Aldrich. It should be noted that this technique relies upon the hydrodynamic radius of the polymer in solution, which may be influenced by hydrogen bonding.[S7] Indeed, work by the Manners group has shown that refractive index measurement relative to polystyrene standards may overestimate the molecular weight by a factor of 3 – 6.[S8,S9] As such, all molecular weights quoted herein should be taken in context relative to one another, and the absolute value given less emphasis.

1.2 Synthesis

Synthesis of **[1-COD]Cl**

To a red solution of $[Ir(COD)Cl]_2$ (140 mg, 0.21 mmol) in 1,2-difluorobenzene (5 mL) was added iPr-PN^{H}P (1.43 mL, 10% wt. soln. in THF, 0.42 mmol). The solution was left to stir at room temperature for 16 hours, after which time a clear yellow colour was observed. The reaction was concentrated *in vacuo* until approximately of 1 mL solvent remained before precipitation was induced through addition of pentane. The crude solid was collected and dried under reduced pressure overnight before recrystallisation from layering a solution of the crude product in 1,2-difluorobenzene with pentane to yield [Ir(iPr-PN^{H}P)(COD)]Cl as colourless crystals (194 mg, 0.30 mmol, 72 %) which were suitable for single crystal X-ray diffraction.

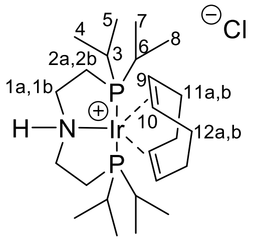

^{1}H NMR (500 MHz, CD_2Cl_2): 7.48 (br s, $\nu_{1/2}$ 17 Hz 1H, N-<u>H</u>), 4.13 (m, 2H, H-9) 3.27 (m, 2H, H-10), 2.96-2.85 (m, 4H, overlapping signals, H-3 /H-1a), 2.58-2.40 (m, 4H, overlapping signals, H-1b/H-11a), 2.27 (m, 2H, H-12a) 2.05 (m, 2H, H-2a), 1.93-1.82 (m, 4H, overlapping signals, H-6 /H-11b) 1.75 (m, 2H, H-12b), 1.56-1.51 (m, 2H, H-2b), 1.49 (dd, 6H, $^3J_{HP}$ 12.7 Hz, $^3J_{HH}$ 7.1 Hz, H-4), 1.42 (dd, 6H, $^3J_{HP}$ 13.0 H, $^3J_{HH}$ 7.8 Hz, H-5), 1.28 (dd, 6H, $^3J_{HP}$ 10.5 Hz, $^3J_{HH}$ 7.1 Hz, H-7/H-8), 1.18 (dd, 6H, $^3J_{HP}$ 14.4 Hz, $^3J_{HH}$ 7.5 Hz, H-7/H-8).

^{13}C{^{1}H} NMR (126 MHz, CD_2Cl_2): 58.0 (overlapping signals, C-9/C-1), 51.0 (m, C-10), 33.7 (C-12), 33.4 (t, J = 6.3 Hz, C-6), 32.5 (C-11), 29.8 (t, J = 12.9 Hz, C-3), 28.6 (m, C-2), 20.7 (C-5), 20.3 (C-4), 19.8 (C-7/C-8).

^{31}P{^{1}H} NMR (203 MHz, CD_2Cl_2): 20.0 (s).

Elemental analysis Calcd. for $C_{24}H_{49}IrClNP_2$ (641.28): C, 44.95; H, 7.70; N, 2.18; Found: C, 44.87; H, 7.55; N, 1.94.

Synthesis of **[1-COD][BAr^F$_4$]**

To an ampoule containing a deep purple solution of [Ir(COD)$_2$][BAr^F$_4$] (223 mg, 0.18 mmol) and 1,5-cyclooctadiene (0.15 mL) in 1,2-difluorobenzene (7 mL), iPr-PN^{H}P (0.53 mL, 10 % wt. soln. in THF, 0.18 mmol) is added. The reaction is left to stir at room temperature for 16 hours, after which time the solution is colourless. The solution was transferred to a crystallisation tube via cannula filter and layered with pentane to afford [Ir(iPr-PN^{H}P)(COD)][BAr^F$_4$] (220 mg, 0.15 mmol, 87 %) as colourless crystals that were suitable for single crystal X-ray diffraction.

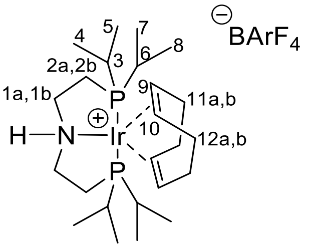

^{1}H NMR (500 MHz, CD$_2$Cl$_2$) 7.72 (m, 8H, BAr^F$_4$), 7.56 (s, 4H, BAr^F$_4$), 4.30 (m, 2H, H-9), 3.47 (s, 1H, N-H), 2.93-2.85 (m, 2H, H-3), 2.85-2.78 (m, 2H, H-1a), 2.77-2.72 (m, 2H, H-1b), 2.51-2.47 (m, 4H, H-12), 2.37-2.30 (m, 2H, H-10) 2.19-2.13 (m, 2H, H-2a), 2.02-1.95 (m, 2H, H-11a/H-11b), 1.94-1.87 (m, 2H, H-6), 1.86-1.81 (m, 2H, H-11a/H-11b), 1.53-1.50 (m, 2H, H-2b), 1.48 (dd, J = 13.3, 7.2 Hz, H-4/H-5), 1.42 (dd, J = 13.8, 7.3 Hz, H-4/H-5), 1.31 (dd, J = 10.9, 7.1 Hz, H-7/H-8), 1.23 (dd, J = 15.1, 7.5 Hz, H-7/H-8).

^{13}C{^{1}H} NMR (126 MHz, CD$_2$Cl$_2$): 162.2 (q, J = 50 Hz, BAr^F$_4$), 135.2 (BAr^F$_4$), 129.3 (q, J = 30.5 Hz, BAr^F$_4$), 128.7-121.2 (m, BAr^F$_4$), 117.9 (BAr^F$_4$), 60.0 (C-9), 59.7 (C-1), 50.7 (C-10), 34.0 (C-11), 33.7 (t, J = 7.2 Hz, C-6), 32.4 (C-12), 30.1 (m, C-2), 29.7 (t, J = 13.1 Hz, C-3), 20.4 (C-4/C5), 20.0 (C-4/C-5) 19.8 (C-7/C-8), 19.6 (C-7/C-8).

^{31}P{^{1}H} NMR (203 MHz, CD$_2$Cl$_2$): 20.1 (s).

Elemental analysis Calcd. for $C_{56}H_{61}BF_{24}IrNP_2$ (1469.05): C, 45.79; H, 4.19; N, 0.95; Found: C, 45.72; H, 4.0; N, 0.71.

in situ Preparation of **[1-H$_4$][BAr$^F_4$]**

To an NMR tube fitted with a J-Young's valve containing **[1-COD][BAr$^F_4$]** (4 mg, 2.7 μmol) was added CD_2Cl_2 (0.4 mL). The NMR tube was degassed via freeze-pump-thaw three times (<10^{-3} mbar) before being charged with H_2 gas (2 bar). The contents of the NMR tube were mixed via inversion for 36 hours, after which time there quantitative conversion to [Ir(iPr-PN^{H}P)(H_2)($H)_2$][BAr$^F_4$] was observed by NMR spectroscopic analysis.

^{1}H NMR (500 MHz, CD_2Cl_2, 298 K): 7.72 (m, 8H, BAr$^F_4$) 7.57 (s, 4H, BAr$^F_4$), 4.60 (s, $\nu_{1/2}$ 2 Hz, free H_2), 3.98 (s, 1H, N-H), 3.52 (m, 2H, N-C$\underline{H}_2$), 2.40 (m, 2H, N-C$\underline{H}_2$), 2.27 (m, 2H, P-C$\underline{H}_2$), 2.05 (m, 2H, C$\underline{H}$-($CH_3)_2$), 1.96 (m, 2H, C$\underline{H}$-($CH_3)_2$), 1.74 (m, 2H, P-C$\underline{H}_2$), 1.53 (s, 16H, free cyclooctane), 1.13 (m, 24H, CH-(C$\underline{H}_3)_2$), –10.17 (br t, $^2J_{HP}$ = 7.6 Hz, 4H, Ir-$\underline{H}$).

^{31}P{^{1}H} NMR (203 MHz, CD_2Cl_2, 298 K): 53.7 (s)

At 298 K, the Ir-H resonance is observed as a triplet, which resolved into a singlet upon ^{31}P decoupling. Variable-temperature (VT) NMR analysis was conducted upon the sample under 2 bar pressure of hydrogen and the signal associated with Ir-H resolved into two broad signals at 185 K.

^{1}H NMR (500 MHz, CD_2Cl_2, 185 K): 4.54 (s, $\nu_{1/2}$ 1.3 Hz, free $\underline{H}_2$) –6.57 (br s, $\nu_{1/2}$ 1046 Hz, 2H, Ir-$\underline{H}$) and –13.85 (br s, $\nu_{1/2}$ 1050 Hz, 2H, Ir-$\underline{H}$).

^{31}P{^{1}H} NMR (203 MHz, CD_2Cl_2, 185 K): 53.7 (s)

T_1 measurements were conducted at 295 K: 232(8) ms, and 185 K: 150(10) ms.

A sample prepared as above was degassed thoroughly (10 × freeze-pump-thaw cycles) and interrogated by NMR spectroscopy. The ^{31}P{^{1}H} NMR spectrum obtained was identical to that above, with some decomposition products observed (ca. 45% after 10 minutes). The ^{1}H NMR spectrum showed identical signals to those above, however the Ir-H signal now integrates to relative 2H; decomposition products are also observed.

Synthesis of **[1-H$_3$B·NMe$_3$][BAr$^F{}_4$]**

To an ampoule containing **[1-COD][BAr$^F{}_4$]** (0.40 g, 0.27 mmol) and H$_3$B·NMe$_3$ (25 mg, 0.27 mmol) was added 1,2-diflourobenzene (10 mL). The solution was degassed via freeze-pump-thaw three times (<10^{-3} mbar) and before charging with H$_2$ gas (2 bar). The reaction was vigorously stirred at room temperature for 20 h, after which time the H$_2$ atmosphere was removed by freeze pump-thaw-degassing, and replaced with argon. The solution was transferred via cannula filter to a recrystallisation tube and layered with pentane to afford colourless crystals of [Ir(iPr-PN^{H}P)(H)$_2$(H$_3$B·NMe$_3$)][BAr$^F{}_4$] (297 mg, 0.21 mmol, 76%) which were suitable for single crystal X-ray diffraction.

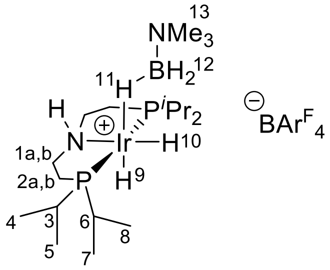

^{1}H NMR (500 MHz, CD$_2$Cl$_2$): 7.73 (s, 8H, BAr$^F{}_4$ *o*-H), 7.57 (s, 4H, BAr$^F{}_4$, *p*-H), 3.36 (m, 2H, H-1b), 3.00 (br s, ν$_{1/2}$ 30 Hz, 1H, N-H), 2.67 (s, 9H, H-13), 2.47 (m, 2H, H-1a), 2.17 (m, 6H, overlapping signals, H-2b/H-3/H-6), 1.69 (m, 2H, H-2a), 1.29 (m, 12H, H-4/H-5), 1.09 (m, 6H, H-7/H-8), 0.99 (m, 6H, H-7/H-8), –2.18 (br s, ν$_{1/2}$ 391 Hz, 3H, H-11/H-12), –20.25 (td, 1H, $^2J_{HP}$ 12.9, $^3J_{HB}$ 6.2 Hz, H-9), –22.74 (br td, 1H, $^2J_{HP}$ 15.9, $^3J_{HB}$ 5.6 Hz, H-10).

^{13}C{^{1}H} NMR (126 MHz, CD$_2$Cl$_2$): 163.1-161.1 (m, BAr$^F{}_4$), 135.2 (BAr$^F{}_4$), 129.3 (q, J = 30.8 Hz, BAr$^F{}_4$), 128.3-121.8 (m, BAr$^F{}_4$), 117.9 (BAr$^F{}_4$), 56.2 (C-1), 54.4 (C-13), 33.3-33.1 (m, C-2), 29.2 (t, J = 14.3 Hz, C-3/C-6) 22.9 (C-7/C-8), 22.3 (t, J = 22.8 Hz, C-3/C-6), 22.0 (C-7/C-8), 19.0 (C-7/C-8), 16.7 (C-4/C-5).

^{31}P{^{1}H} NMR (203 MHz, CD$_2$Cl$_2$): 51.1 (s).

^{11}B NMR (193 MHz, CD$_2$Cl$_2$): –6.62 (s, BAr$^F{}_4$), –14.44 (br s, BH$_3$)

Elemental analysis Calcd. for C$_{51}$H$_{63}$B$_2$F$_{24}$IrN$_2$P$_2$ (1435.83): C, 42.66; H, 4.42; N, 1.95; Found: C, 42.99; H, 4.51; N, 1.43.

VT NMR was conducted on a sample and found that the BH$_3$ moiety (μH-B and terminal B-H are in rapid fluxional exchange at room temperature) is resolved into two singlets at 183 K; corresponding to the bridging Ir-μH-B and terminal B-H signals.

^{1}H NMR (500 MHz, CD$_2$Cl$_2$, 183K): 1.87 (br s, 2H, ν$_{1/2}$ 78 Hz, H-12), –11.07 (s, 1H, ν$_{1/2}$ 29 Hz, H-11).

^{31}P{^{1}H} NMR (203 MHz, CD$_2$Cl$_2$, 183K): 51.6 (s).

Addition of H$_2$ (2 bar) to a sample of **[1-H$_3$B·NMe$_3$][BAr$^F{}_4$]** in CD$_2$Cl$_2$ reveals an equilibrium between this and [(HPNP^{iPr})Ir(H)$_2$(H$_2$)][BAr$^f{}_4$] which is likely pressure sensitive. Subsequent degassing of the sample through freeze-pump-thaw allows quantitative formation of **[1-H$_3$B·NMe$_3$][BAr$^F{}_4$]** again:

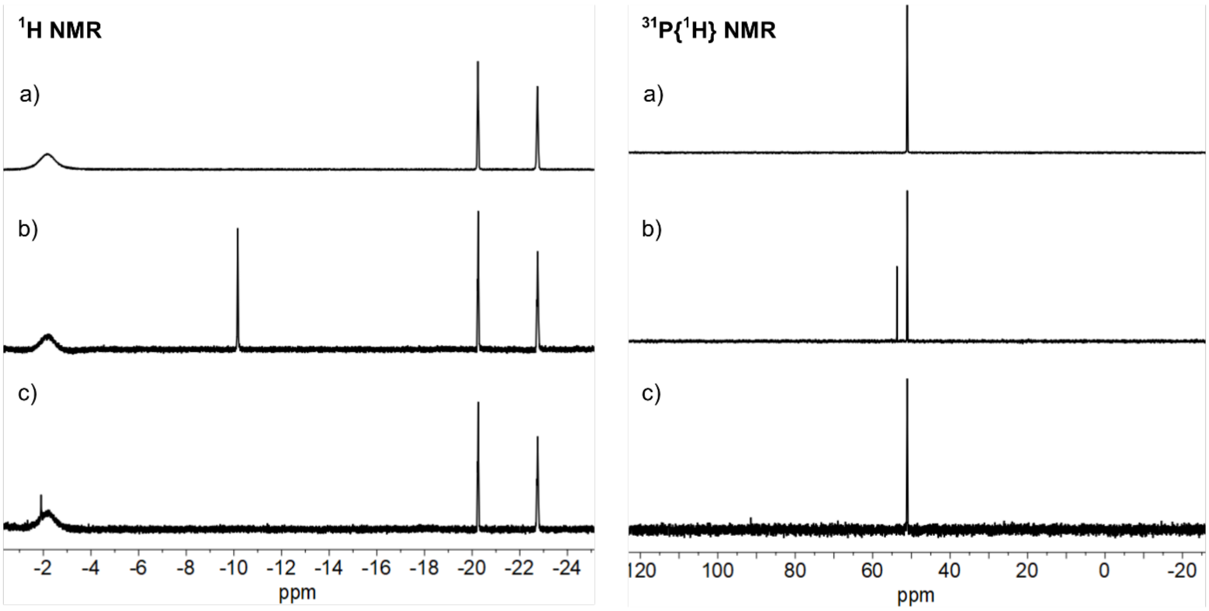

Figure S1 ^{1}H (500 MHz) and $^{31}P\{^{1}H\}$ (203 MHz) NMR spectra (CD_2Cl_2) for reaction of **[1-H_3B·NMe_3][$BAr^F{}_4$]** (a) with H_2 (b) and subsequent degassing (c) showing displacement of the amine-borane moiety by H_2 under an atmosphere of H_2.

Addition of D_2 (1 bar) to a sample of **[1-H_3B·NMe_3][$BAr^F{}_4$]** in CH_2Cl_2 shows H/D exchange at the hydride positions and borane group, along with the same equilibrium observed above between [Ir(PN^HP^{iPr})(H/D)$_2$(H/D_3B·NMe_3)][$BAr^F{}_4$] and [Ir(PN^HP^{iPr})(H/D)$_2$(H/D_2)][$BAr^F{}_4$]:

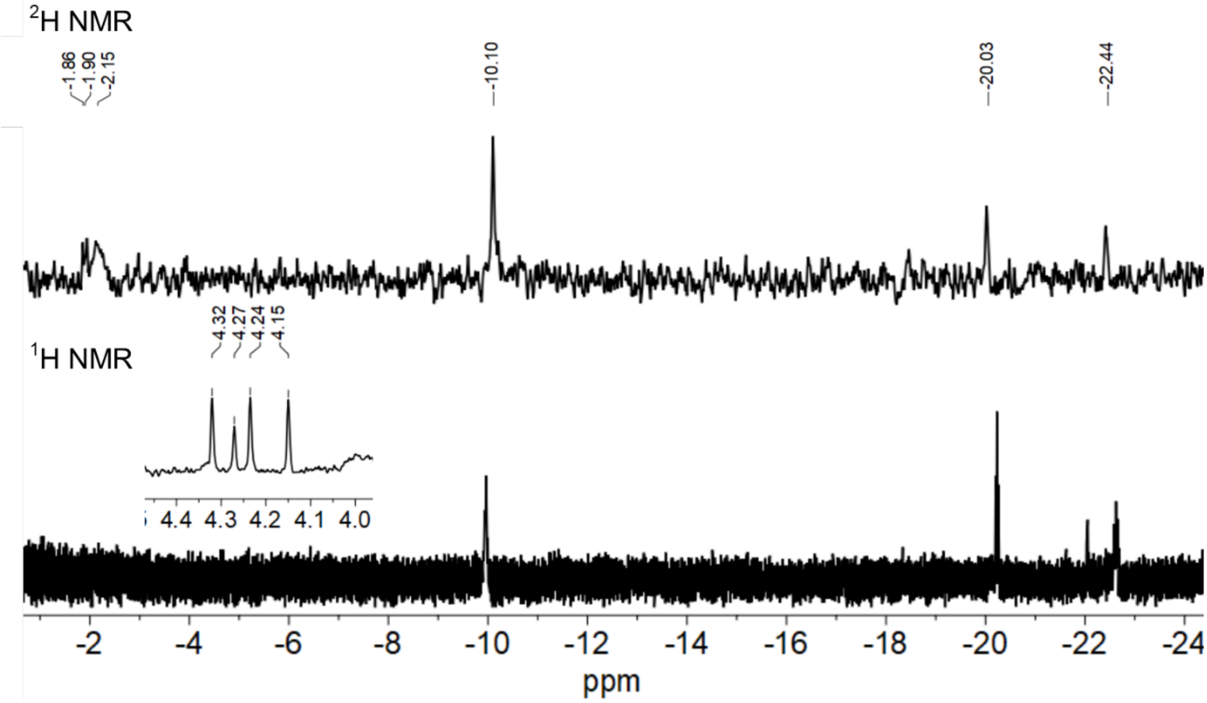

Figure S2 ^{1}H (500 MHz) and ^{2}H (77 MHz) NMR spectra (CH_2Cl_2) for reaction of **[1-H_3B·NMe_3][$BAr^F{}_4$]** with D_2 showing equilibrium with [Ir(iPr-PN^HP)(H/D)$_2$(H/D_2)][$BAr^F{}_4$] and H/D exchange at Ir-H and borane group. Dissolved HD gas is observed in the ^{1}H NMR spectrum (inset) as the expected 1:1:1 triplet with J = 43 Hz.

Synthesis of **[1-NMeH$_2$][BAr$^F{}_4$]**

To an ampoule containing a solution of **[1-H$_3$B·NMe$_3$][BAr$^F{}_4$]** (60 mg, 42 μmol) in 1,2-$C_6H_4F_2$ (2.5 mL) was added $NMeH_2$ (0.2 mL, 1M soln. in THF, 0.2 mmol) and the reaction left to stir at room temperature for 16 h. The reaction mixture was concentrated to 0.5 mL before addition of pentane (30 mL) to induce precipitation at –78 °C. The resulting white solid was collected by filtration, washed with pentane (2 × 10 mL) at –78 °C and dried under reduced pressure to yield [Ir(iPr-PN^{H}P)(H)$_2$(NMeH$_2$)][BAr$^F{}_4$] as a white solid (18 mg, 13 μmol, 31%). Crystals suitable for single crystal X-ray diffraction were grown from layering a solution of **[1-NMeH$_2$][BAr$^F{}_4$]** in 1,2-$C_6H_4F_2$ with pentane, however, all crystals trialled suffered from severe twinning, and so a satisfactory solution was not obtained.

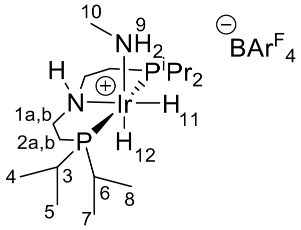

^{1}H NMR (500 MHz, CD_2Cl_2): δ_H 7.73 (s, 8H, BAr$^F{}_4$), 7.57 (s, 4H, BAr$^F{}_4$), 3.56-3.45 (m, 2H, H-2a), 3.20 (br s, 1H, N-H), 2.66-2.59 (overlapping signals, 4H, H-1a/H-9), 2.47 (t, 3H, J = 6.7 Hz, H-10), 2.22-2.15 (m, 2H, H-2b), 2.15-2.09 (m, 2H, H-3), 1.98-1.89 (m, 2H, H-6), 1.62 (td, 2H, J = 14.9, 5.5 Hz, H-1b), 1.35-1.38 (overlapping dd, 12H, H-7/H-8), 1.09 (dd, 6H, J = 17.1, 6.9 Hz, H-4), 0.99 (dd, 6H, J = 14.4, 6.8 Hz, H-5), –20.57 (td, 1H, J = 13.1, 6.4 Hz, H-11), –23.98 (td, 1H, J = 16.5, 6.5 Hz, H-12).

^{13}C{^{1}H} NMR (126 MHz, CD_2Cl_2): δ_C 162.2 (dd, J = 99.7, 49.8 Hz, BAr$^F{}_4$), 135.2 (BAr$^F{}_4$), 129.3 (q, J = 31.6 Hz, BAr$^F{}_4$), 117.9 (BAr$^F{}_4$), 56.6 (C-2), 35.8 (C-10), 34.4 (t, J = 12.3 Hz, C-1), 27.0 (t, J = 13.6 Hz, C-6), 23.4 (t, J = 17.3 Hz, C-3), 22.1 (C-4), 21.0 (C-7/C-8), 18.7 (C-5), 17.2 (C-7/C-8).

^{31}P{^{1}H} NMR (203 MHz, CD_2Cl_2): δ_P 48.4 (s).

Despite multiple attempts, satisfactory microanalysis was not obtained (returned consistently low in N). Elemental analysis Calcd. for $C_{49}H_{56}BF_{24}IrN_2P_2$ (1393.92): C, 42.22; H, 4.05; N, 2.01. Calcd. For $C_{48}H_{51}BF_{24}IrNP_2$ (1362.86): C, 42.30; H, 3.77; N, 1.03. Found: C, 41.97; H, 3.43; N, 1.16.

Synthesis of **1-H$_2$Cl**

A modification to the literature procedure[S10] was used to prepare **1-H$_2$Cl**, that is an adaptation to the reported procedures for preparation of Rh(iPr-PN^{H}P)ClH$_2$, adapted for iridium.[S10]

To a solution of [Ir(COD)Cl]$_2$ (108 mg, 0.16 mmol) in 1,2-$C_6H_4F_2$ (3 mL) was added iPr-PN^{H}P (101 mg, 0.33 mmol). The resulting solution was degassed by freeze-pump-thaw, placed under a pressure of H_2 (4 bar) and allowed to stir at room temperature for 48 hours. Removal of volatile components yields a crude white solid that was washed with pentane (3 × 5 mL) at –78 °C. The resulting white solid was dried under reduced pressure to yield **1-H$_2$Cl** (142 mg, 0.27 mmol, 82%).

Or

A solution of **[1-COD]Cl** (65 mg, 0.101 mmol) in 1,2-$C_6H_4F_2$ (5 mL) was degassed by freeze-pump-thaw and placed under a pressure of H_2 (4 bar) and heated to 70 °C for 4 days. Slow cooling of the solution yields **1-H$_2$Cl** as colourless crystals (54 mg, 0.064 mmol, 63 %).

NMR spectroscopic data are in line with those that have been reported previously.[S10]

Preparation *of* **1-H₃**

1-H_3 was prepared (unoptimized) with a modification to the literature procedure.[S10] To an ampoule containing **1-H_2Cl** (66 mg, 120 μmol) and KOtBu (13.8 mg, 0.12 mmol) was added isopropanol (3 mL) and the resulting solution stirred for 2 hours at room temperature. Volatile components were removed in vacuo and the resulting crude product extracted into toluene (10 mL). Removal of volatiles yields a crude solid that was washed with pentane (3 × 5 mL) at 78 °C to yield a white solid of **1-H_3** (25 mg, 50 μmmol, 42 %). Spectroscopic data matches that previously reported (THF-d_8).[S10]

in situ preparation: The reported procedure for preparation of Rh(PN^{H}P)H_3 was adapted for iridium.[S11]
To an ampoule containing **1-H_2Cl** (66 mg, 0.12 mmol) and KOtBu (13.8 mg, 0.12 mmol) was added 1,2-$C_6H_4F_2$ (3 mL) and the resulting solution immediately degassed via freeze-pump-thaw (3 cycles). H_2 (4 bar) was added and the resulting mixture stirred at room temperature for 16 hours, after which time, complete, clean conversion to **1-H_3** was observed by *in situ* NMR spectroscopic monitoring. This reaction was also successfully performed in THF and C_6D_6 solvents.

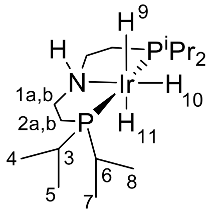

^{1}H NMR (600 MHz, 1,2-$C_6H_4F_2$): δ_H 3.36 (br t, $\nu_{1/2}$ 33 Hz, 1H, N-H), 3.26 – 3.14 (m, 2H, H-1b), 2.14 –2.09 (m, 2H, H-1a), 2.09 – 2.03 (m, 2H, H-2b), 1.93 – 1.84 (2 × overlapping heptets, $^2H_{PH}$ 6.90 Hz, 6.74 Hz, 4 H, H-3/H-6), 1.59 – 1.53 (m, 2H, H-1a), 1.35 – 1.22 (4 × overlapping doublets, 24H, H-4/5/7/8), 1.32 (s, 9H, <u>tBu</u>OH), –11.47 (td, 1H, $^2J_{PH}$ 16.4 Hz, $^2J_{HH}$ 5.5 Hz, H-11), –12.26 (td, 1H, $^2J_{PH}$ 17.3 Hz, $^2J_{HH}$ 5.2 Hz, H-9), –22.25 (tt, 1H, $^2J_{PH}$ 14.6 Hz, $^2J_{HH}$ 5.4 Hz, H-10). z

^{31}P{^{1}H} (243 MHz, 1,2-$C_6H_4F_2$): δ_P 61.5 (s).

A sample of **1-H_3** was prepared as above (*in situ*) from **1-H_2Cl** (8 mg, 0.015 mol) and KOtBu (1.8 mg, 0.015 mmol) in THF-d$_8$ (0.5 mL) in an NMR tube equipped with a J-Youngs valve. To this, [NMeH_3][BAr$^F_4$] (13.4 mg, 0.015 mmol) was added and the resultant solution interrogated by NMR spectroscopies where complete conversion to **[1-NMeH_2][BAr$^F_4$]** was observed.

Reaction between $[NMe_2H_2][BAr^F{}_4]$ and $[H_2BNMe_2]_2$

An NMR tube fitted with a J-Young NMR valve was charged with $[NMe_2H_2][BAr^F{}_4]$† (20 mg, 22 μmol), $[H_2BNMe_2]_2$ (1.5 mg, 11 μmol) and 1,2-$C_6H_4F_2$ (0.5 mL). The tube was heated to 50 °C to promote dimer dissociation for 72 h, after which time the resulting solution was interrogated by NMR spectroscopies. The resulting solution was found to contain $[BH_2(NMe_2H)_2][BAr^F{}_4]$[S12] (100% conversion by ^{11}B NMR).

^{1}H NMR (600 MHz, 1,2-$C_6H_4F_2$): 8.32 (s,8H, $BAr^F{}_4$), 7.69 (s, 4H, $BAr^F{}_4$), 4.30 (br s, 2H, FWHM 55 Hz, $NMe_2\underline{H}$), 2.77 (d, 12H, $^3J_{BH}$ 5 Hz, $N\underline{Me_2}H$), 2.12 (br q, 2H, FWHM 109 Hz $^1J_{BH}$ 120 Hz, $B\underline{H_2}$).

$^1H\{^{11}B\}$ NMR (600 MHz, 1,2-$C_6H_4F_2$): 8.32 (s, 8H, $BAr^F{}_4$), 7.69 (s, 4H, $BAr^F{}_4$), 4.30 (br s, 2H, FWHM 51 Hz, $NMe_2\underline{H}$), 2.78 (s, 12H, $N\underline{Me_2}H$) and 2.12 (s, 2H, $B\underline{H_2}$).

^{11}B NMR (193 MHz, 1,2-$C_6H_4F_2$): –2.12 (t, J_{BH} 119 Hz, $[\underline{B}H_2(NMe_2)_2]^+$), –6.16 (s, $[\underline{B}Ar^F{}_4]^-$).

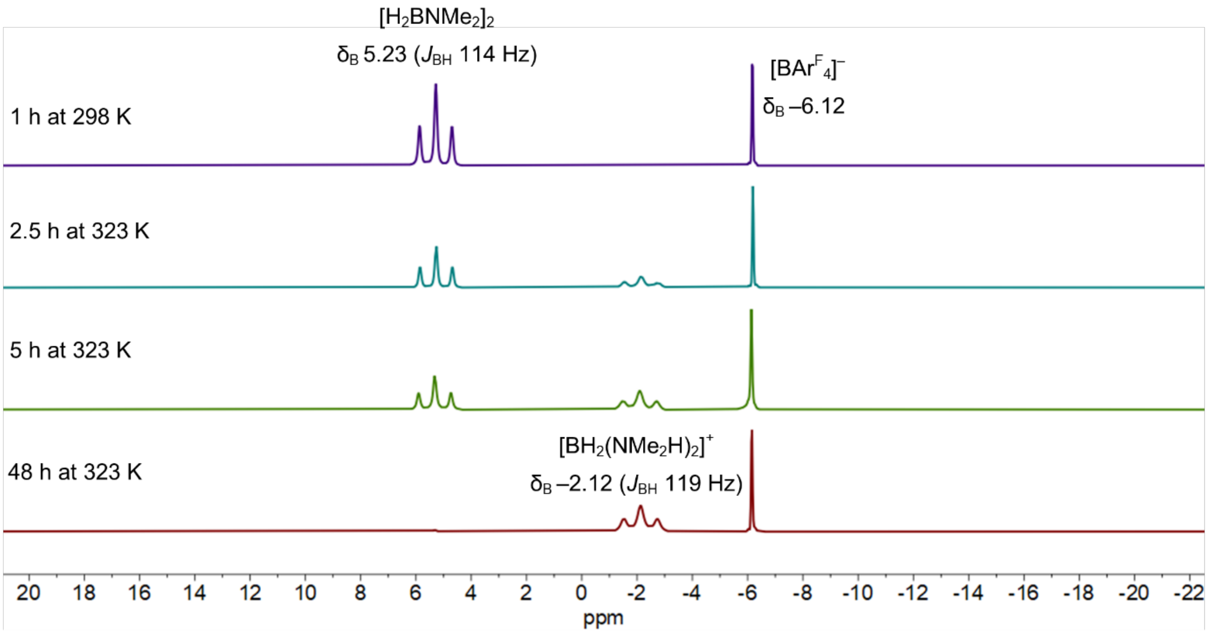

Figure S3 ^{11}B NMR spectra (193 MHz, 1,2-$C_6H_4F_2$) showing the progression of the reaction between $[NMe_2H_2][BAr^F{}_4]$ with ½ $[H_2BNMe_2]_2$ over time.

† *$[NMe_2H_2][Cl]$ was prepared in line with that reported for $[NMeH_3]Cl$ by Im.*[S4] To a solution of $NMeH_2$ in diethyl ether at 0 °C was added 1.1 eq. HCl in ether (1 M) dropwise and the solution stirred for one hour from which the product precipitates. Washing with pentane and drying under reduced pressure yields $[NMe_2H_2]Cl$. *$[NMe_2H_2][BAr^F{}_4]$ was prepared in line with that reported for $[BH_2(NMeH)_2][BAr^F{}_4]$ by Conejero.*[S12] $[NMe_2H_2]Cl$ and one equivalent of $NaBAr^F{}_4$ are dissolved in DCM and the reaction stirred for 1 h before collection by filtration. The resulting solid was washed with pentane and dried under reduced pressure to yield $[NMe_2H_2][BAr^F{}_4]$.

Synthesis of **[2-COD][BAr$^F_4$]**

To an ampoule containing a deep purple solution of [Ir(COD)$_2$][BAr$^F_4$] (172 mg, 0.14 mmol) and 1,5-cyclooctadiene (0.1 mL) in 1,2-difluorobenzene (3 mL), iPr-PN^{Me}P (1.25 mL, 0.12 M in 1,2-$C_6H_4F_2$, 0.15 mmol) is added. The reaction is left to stir at room temperature for 5 hours, after which time the solution is colourless. The solution was transferred to a crystallisation tube via cannula filter and layered with hexamethyldisiloxane to afford [Ir(iPr-PN^{Me}P)(COD)][BAr$^F_4$] (152 mg, 0.10 mmol, 71 %) as colourless crystals that were suitable for single crystal X-ray diffraction.

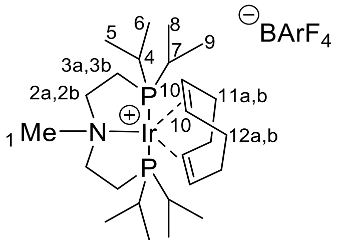

^{1}H NMR (600 MHz, CD_2Cl_2) 7.72 (m, 8H, BAr$^F_4$), 7.56 (s, 4H, BAr$^F_4$), 3.25 (br s, FWHM 105 Hz, 4H, H-10), 3.02-2.94 (m, 2H, H-4), 2.60-2.48 (m, 4H, H-2a,2b), 2.40-2.35 (br m, 4H, H-11a,12a), 2.16 (s, 3H, H-1), 2.00-1.91 (m, 4H, H-3a,H-7), 1.86-1.81 (m, 2H, H-3b) 1.72-1.69 (br m, 4H, H-11b,12b), 1.52-1.48 (m, 12H, H-5,6), 1.29 (dd, J = 11.0, 7.4 Hz, 6H, H-8), 1.17 (dd, J = 14.9, 7.2 Hz, H-9).

^{13}C{^{1}H} NMR (151 MHz, CD_2Cl_2): 162.2 (q, J = 50 Hz, BAr$^F_4$), 135.2 (BAr$^F_4$), 129.3 (m, BAr$^F_4$), 129.0 (C-10), 125.0 (q, J = 272 Hz, BAr$^F_4$), 117.9 (m, BAr$^F_4$), 67.6 (t, J = 5.3 Hz, C-2), 49.6 (C-1), 33.6 (m, C-7), 32.5 (br s, C-11/12), 29.0 (m, C-4), 28.4 (C-11/12), 23.1 (m, C-3), 20.5 (C-5/6), 20.1 (m, C-5/6), 19.5 (t, J = 3.6 Hz, C-8), 19.2 (C-9).

^{31}P{^{1}H} NMR (203 MHz, CD_2Cl_2): 20.6 (s).

Elemental analysis Calcd. for $C_{57}H_{63}BF_{24}IrNP_2$ (1483.07): C, 46.16; H, 4.28; N, 0.94; Found: C, 46.59; H, 3.73; N, 0.41.

Synthesis of **[2-$H_3B\cdot NMe_3$][BAr^F_4]**

To an ampoule containing **[2-COD][BAr^F_4]** (0.10 g, 0.07 mmol) and $H_3B\cdot NMe_3$ (8 mg, 0.11 mmol) was added 1,2-diflourobenzene (4 mL). The solution was degassed via freeze-pump-thaw three times (<10^{-3} mbar) and before charging with H_2 gas (4 bar). The reaction was vigorously stirred at room temperature for 4 h, after which time the H_2 atmosphere was removed by freeze pump-thaw-degassing, and replaced with argon. The solution was transferred via cannula filter to a recrystallisation tube and layered with pentane to afford colourless crystals of [Ir(iPr-PN^{Me}P)(H)$_2$($H_3B\cdot NMe_3$)][BAr^F_4] (58 mg, 0.04 mmol, 57%) which were suitable for single crystal X-ray diffraction. The NMR spectroscopic data show a ca. 10% impurity of the intermediary species, **[2-H_4][BAr^F_4]** alongside a small 1,2-$C_6H_4F_2$ impurity that is persistent and present in the crystallographic lattice (*vide infra*).

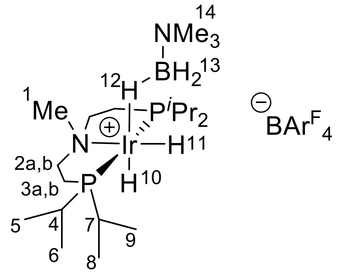

^{1}H NMR (600 MHz, CD_2Cl_2): 7.72 (s, 8H, BAr^F_4 *o*-H), 7.56 (s, 4H, BAr^F_4, *p*-H), 2.89-2.81 (m, 2H, H-3a), 2.65 (s, 12H, H-14), 2.53 (s, 3H, H-1), 2.51-2.46 (m, 4H, H-2b,H-4), 2.45-2.40 (m, 2H, H-3b), 2.15-2.09 (m, 2H, H-7), 2.07-2.01 (m, 2H, H-2a), 1.31-1.23 (m, 12H, H-5,6), 1.09 (dd, J = 16.3 7.7 Hz, 6H, H-8), 1.03 (dd, J = 14.7, 7.2 Hz, 6H, H-9), –1.98 (br s, $\nu_{1/2}$ 273 Hz, 3H, H-12/H-13), –22.69 (s, 1H, H-10), –24.32 (s, 1H, H-11).

^{f13}C{^{1}H} NMR (151 MHz, CD_2Cl_2): 162.2 (q, J= 50 Hz, BAr^F_4), 135.2 (BAr^F_4), 129.3 (qq, J = 31.2, 2.9 Hz, BAr^F_4), 125.0 (q, J = 273.2 Hz, BAr^F_4), 117.9 (BAr^F_4), 67.1 (C-3), 54.4 (C-14), 49.4 (C-1), 30.9 (t, J = 12.2 Hz, C-2), 28.0 (m, C-4), 23.5 (m, C-7), 22.8 (C-8), 20.6 (C-5/6), 19.1 (C-9), 17.4 (C-5/6).

^{31}P{^{1}H} NMR (243 MHz, CD_2Cl_2): 43.5 (s).

Elemental analysis Calcd. for $C_{58}H_{69}B_2F_{26}IrN_2P_2$ (1563.95): C, 44.54; H, 4.45; N, 1.79; Found: C, 44.14; H, 3.7; N, 0.72.

in situ Preparation of **[2-H$_4$][BAr$^F_4$]**

To an NMR tube fitted with a J-Young's valve containing **[2-COD][BAr$^F_4$]** (5 mg, 3.4 μmol) was added CD_2Cl_2 (0.5 mL). The NMR tube was degassed via freeze-pump-thaw three times (<10^{-3} mbar) before being charged with H_2 gas (2 bar). The contents of the NMR tube were mixed via inversion for 36 hours, after which time there quantitative conversion to [Ir(iPr-PN^{Me}P)(H_2)(H)$_2$][BAr$^F_4$] was observed by NMR spectroscopic analysis.

^{1}H NMR (500 MHz, CD_2Cl_2, 298 K): 7.72 (m, 8H, BAr$^F_4$) 7.56 (s, 4H, BAr$^F_4$), 3.05-2.96 (m, 2H, P-C$\underline{H}_2$), 2.93 (s, 3H, N-C$\underline{H}_3$), 2.65-2.59 (m, 2H, N-C$\underline{H}_2$), 2.47-2.41 (m, 2H, N-C$\underline{H}_2$), 2.21-2.13 (m, 2H, P-C$\underline{H}_2$), 2.11-2.07 (m, 2H, C$\underline{H}$-(CH$_3$)$_2$), 2.05-1.99 (m, 2H, C$\underline{H}$-(CH$_3$)$_2$), 1.53 (s, free cyclooctane), 1.16 (m, 24H, CH-(C$\underline{H}_3$)$_2$), –10.65 (br s, 4H Ir-$\underline{H}$).

^{31}P{^{1}H} NMR (203 MHz, CD_2Cl_2, 298 K): 53.0 (s)

A T_1 measurement was conducted at 298 K: 242(2) ms.

At 298 K, the Ir-H resonance is observed as a triplet, which resolved into a singlet upon ^{31}P decoupling. Cooling the sample to 185 K allows resolution of the signals associated with Ir-H into two signals.

^{1}H NMR (500 MHz, CD_2Cl_2, 185 K): 4.53 (s, $\nu_{1/2}$ 3.7 Hz, free $\underline{H}_2$) –4.87 (br s, $\nu_{1/2}$ 55 Hz, 2H, Ir-$\underline{H}$) and –16.55 (br t, $\nu_{1/2}$ 37 Hz, 2H, Ir-$\underline{H}$).

^{31}P{^{1}H} NMR (203 MHz, CD_2Cl_2, 185 K): 52.5 (s)

Degassing a sample prepared as above reveals ca. 12% decomposition after 10 minutes – likely dihydrogen loss as the signal associated with Ir-H –10.65 decreases in intensity when compared to ligand resonances. No free phosphine ligand is observed.

in situ Preparation of **[2-NMeH$_2$][BAr^F$_4$]**

To an NMR tube fitted with a J-Young's valve containing a solution of **[2-H$_4$][BAr^F$_4$]** (prepared *in situ* from hydrogenation of **[2-COD][BAr^F$_4$]**, 5 mg, 3.4 μmol) in CD_2Cl_2 (0.5 mL) was added $NMeH_2$ (50 μL, 1M soln. in THF, 50 μmol) and the reaction left to stir at room temperature for 1h. The sample was interrogated by NMR spectroscopy and the resultant sole organometallic species characterised *in situ* as [Ir(iPr-PN^{Me}P)(H)$_2$(NMeH$_2$)][BAr^F$_4$].

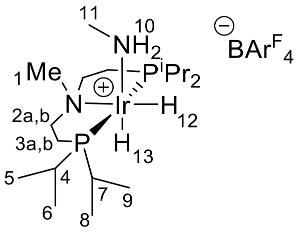

^{1}H NMR (600 MHz, CD_2Cl_2): δ_H 7.71 (m, 8H, BAr^F$_4$), 7.55 (s, 4H, BAr^F$_4$), 3.01-2.91 (m, 2H, H-3a), 2.79 (t, 3H, H-11), 2.64 (s, 2H, H-10), 2.55-2.50 (m, 2H, H-3b), 2.48-2.42 (m, 2H, H-2a), 2.41 (s, 3H, H-1), 2.18-2.08 (m, 4H, H-4,7), 2.02-1.96 (m, 2H, H-2b), 1.33 (dd, 6H, J = 14.8, 7.2 Hz, H-5/6), 1.29 (dd, 6H, J = 13.2, 6.7 Hz, H-5/6), 1.08 (m, 6H, H-8), 0.99 (dd, 6H, J = 15.1, 7.2 Hz, H-9), –22.12 (td, 1H, J = 13.6, 6.9 Hz, H-12), –24.21 (td, 1H, J = 16.8, 6.7 Hz, H-13).

^{31}P{^{1}H} NMR (243 MHz, CD_2Cl_2): δ_P 44.1 (s).

1.3 Catalytic Dehydropolymerisation of $H_3B \cdot NMeH_2$

Typical dehydropolymerisation procedure under open conditions:

In a typical experiment (e.g. 0.223 M [$H_3B \cdot NMeH_2$], 1 mol% catalyst loading), $H_3B \cdot NMeH_2$ (50.0 mg, 1110.0 μmol) was suspended in 1,2–$F_2C_6H_4$ in a J-Young's ampoule. 1.5 × the desired amount of the catalyst was weighed into a separate flask and dissolved in 1,2–$F_2C_6H_4$. The correct volume of the catalyst solution (such that the total volume of the final system was 5.0 mL; and that the catalyst loading was 1 mol%) was added to the reaction mixture, a bleed needle was added to the suba seal present and argon flow dictated by a needle valve present prior to the Schlenk line. The resultant solution was stirred at 400 rpm for the desired reaction time. Upon completion of desired reaction time, 40.0 mL of pentane was added to the reaction mixture with rapid stirring to give a suspension which was stirred for 5 minutes to allow polymer precipitation. The resulting polymer was then isolated by filtration. The off-white to white solid $(H_2BNMeH)_n$ was dried under vacuum. Isolated yields of $(H_2BNMeH)_n$ varied from 34 – 76%.

Typical dehydropolymerisation procedure under eudiometric (H_2 evolution) measurement conditions:

In a typical experiment (e.g. 0.223 M [$H_3B \cdot NMeH_2$], 1 mol% catalyst loading), $H_3B \cdot NMeH_2$ (50.0 mg, 1110.0 μmol) was suspended in 1,2–$F_2C_6H_4$ in a jacketed two–neck Schlenk flask connected to a recirculating cooler and the temperature set at 20 °C. 1.5 × the desired amount of the catalyst was weighed into a separate flask and dissolved in 1,2–$F_2C_6H_4$. The $H_3B \cdot NMeH_2$–containing flask was sealed off from the argon supply and connected to a water–filled 100.0 mL gas burette. The correct volume of the catalyst solution (such that the total volume of the final system was 5.0 mL; and that the catalyst loading was 1 mol%) was added to the reaction mixture and the resultant solution was stirred at 400 rpm. The time taken at least per 1.0 mL of hydrogen gas to evolve was recorded (as measured by water displacement from the attached burette), aided by videography when required, to obtain a temporal plot of the dehydrogenation reaction progress for use in kinetic analyses. Upon completion of gas evolution, 40.0 mL of pentane was added to the reaction mixture with rapid stirring to give a suspension which was stirred for 5 minutes to allow polymer precipitation. The resulting polymer was then isolated by filtration. The off–white to white solid $(H_2BNMeH)_n$ was dried under vacuum. Isolated yields of $(H_2BNMeH)_n$ varied from 23 – 79%.

Typical dehydropolymerisation procedure under additive doped conditions:

In a typical experiment, (e.g. 0.223 M [$H_3B \cdot NMeH_2$], 1 mol% catalyst loading), $H_3B \cdot NMeH_2$ (50.0 mg, 1110.0 μmol) and additive (2 eq. to [Ir]) were suspended in 1,2–$F_2C_6H_4$ in a jacketed two–neck Schlenk flask connected to a recirculating cooler and the temperature set at 20 °C. 1.5 × the desired amount of the catalyst was weighed into a separate flask and dissolved in 1,2–$F_2C_6H_4$. The $H_3B \cdot NMeH_2$–containing flask was sealed off from the argon supply and connected to a water–filled 100.0 mL gas burette. The correct volume of the catalyst solution (such that the total volume of the final system was 5.0 mL; and that the catalyst loading was 1mol%) was added to the reaction mixture and the resultant solution was stirred at 400 rpm. Upon completion of gas evolution, 40.0 mL of pentane was added to the reaction mixture with rapid stirring to give an off–white suspension which was stirred for 5 minutes to allow polymer precipitation. The resulting polymer was then isolated by filtration. The off–white solid $(H_2BNMeH)_n$ was dried under vacuum. Isolated yields of $(H_2BNMeH)_n$ varied from 44 – 54%.

1.3.1 Catalyst loading

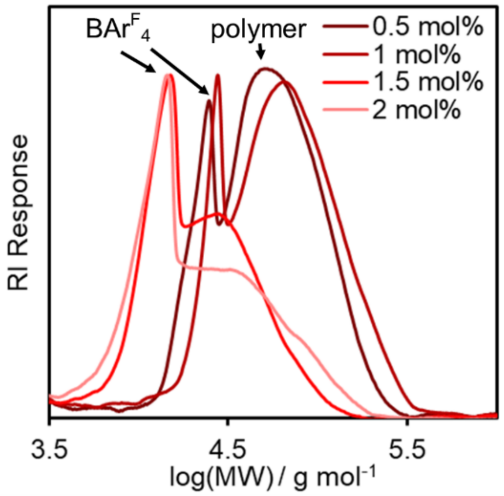

Figure S4 GPC trace obtained of polymer samples prepared using various catalyst loadings. Experimental conditions: 0.5 – 2.0 mol% **[1-H_3B·NMe_3][$BAr^F{}_4$]**, 0.223 M H_3B·$NMeH_2$ in 1,2-$C_6H_4F_2$, jacketed-Schlenk, 293 K.

1.3.2 Effect of Solvent

The kinetic profile obtained using 1,2-$C_6H_4F_2$ as a solvent suggests there is a solubility effect in play. In an attempt to remedy this, dehydropolymerisation was also trialled in THF solvent (Figure S5). However, using H_3B·$NMeH_2$ substrate at 0.223 M in THF results in a slower rate of reaction (Table S1) and poor conversion, possibly due to solvent inhibition though coordination to [Ir] during turnover.

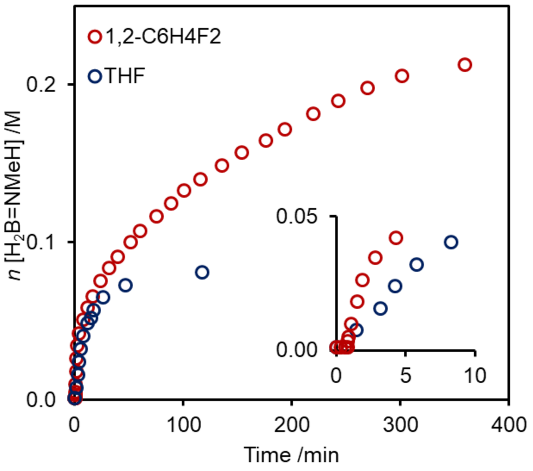

Figure S5 Kinetics of hydrogen evolution for dehydropolymerisation of H_3B·$NMeH_2$ using THF and 1,2-$C_6H_4F_2$ solvent. Experimental conditions: 1 mol% **[1-H_3B·NMe_3][$BAr^F{}_4$]**, 0.223 M [H_3B·$NMeH_2$], jacketed-schlenk, 293 K. Inset: expansion of first 10 minutes of reaction.

Table S1 Effect of solvent on $H_3B{\cdot}NMeH_2$ dehydropolymerisation.[a]

**Solvent**	k_{obs}[b] **/ ×10⁴ M s⁻¹**
1,2-$C_6H_4F_2$	6(2)
THF	1.0(1)

[a] Experimental conditions: 1 mol% **[1-H₃B·NMe₃][BArF₄]**, 0.223 M [$H_3B{\cdot}NMeH_2$], jacketed-schlenk, 293 K. [b] Initial rate measurement.

Indeed, increasing the substrate concentration to 1.11 M in THF results in a significant increase in the rate of reaction than that in 1,2-$C_6H_4F_2$ where $H_3B{\cdot}NMeH_2$ is 0.223 M. [$H_3B{\cdot}NMeH_2$] decreases as the reaction progresses, inhibition by THF coordinaiton is again observed through rapid decelleration and imcomplete conversion (Figure S6). For this reason, combined with no great benefit to the rate from substrate solubility garnered in THF, the solvent of choice for this [Ir] system is 1,2-$C_6H_4F_2$.

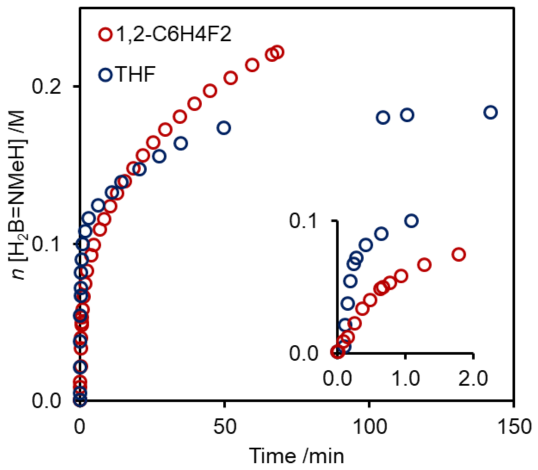

Figure S6 Kinetics of hydrogen evolution for dehydropolymerisation using THF (1.11 M $H_3B{\cdot}NMeH_2$) and 1,2-$C_6H_4F_2$ (0.223 M $H_3B{\cdot}NMeH_2$) solvent. Experimental conditions: 2 mol% **[1-H₃B·NMe₃][BArF₄]**, jacketed-schlenk, 293 K. Inset: expansion of first 2 minutes of reaction.

Table S2 Effect of solvent on $H_3B{\cdot}NMeH_2$ dehydropolymerisation.[a]

**Solvent**	**[H₃B·NMeH₂]**	k_{obs}[b] **/ ×10² M s⁻¹**
1,2-$C_6H_4F_2$	0.223	0.014(2)
THF	1.11	1.1(2)

[a] Experimental conditions: 2 mol% **[1-H₃B·NMe₃][BArF₄]**, jacketed-schlenk, 293 K. [b] Initial rate measurement.

1.3.3 Additive Doping

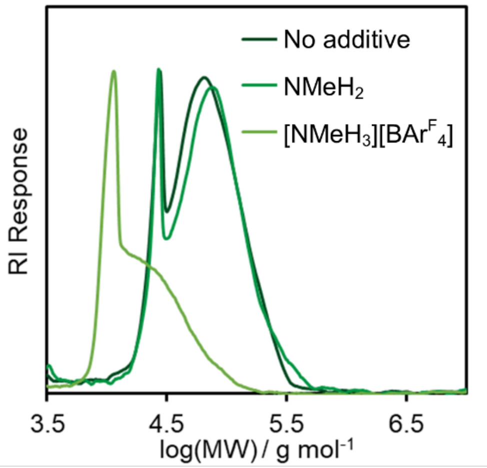

Figure S7 GPC trace obtained of polymer samples prepared with and without additive (2 eq. to [Ir]). Experimental conditions: 1 mol% **[1-H_3B·NMe_3][$BAr^F{}_4$]**, 0.223 M H_3B·$NMeH_2$ in 1,2-$C_6H_4F_2$, jacketed-Schlenk, 293 K.

1.3.4 Alternate Pre-Catalysts

Table S3 Attempted amine-borane dehydropolymerisation using **1-H₂Cl** and **1-H₃**.[a]

Cat.	Solvent	Temp /K	Time /h	$[H_3B{\cdot}NMeH_2]_0$ /M	Conversion[b] /%
**1-H_2Cl**	THF	293	3	1	4
**1-H_2Cl**	THF	293	3	0.223	15
**1-H_2Cl**	1,2-$C_6H_4F_2$	293	6	0.223	39
**1-H_3**	1,2-$C_6H_4F_2$	293	3	0.223	0
**1-H_3**	1,2-$C_6H_4F_2$	333	0.5	0.223	99

[a] Eudiometric conditions, 1 mol% cat. [b] Measured by ^{11}B NMR spectroscopy.

Attempts to perform dehydropolymerisation using 1-**H_3** at room temperature were unsuccessful (0% conversion over 3 hours). However, heating this reaction to 60 °C allows 99% conversion over 30 minutes. The resulting mixture contains: polyaminoborane (bimodal), $[BH_2NMeH]_3$, $[BHNMe]_3$, $BH(NMeH)_2$ and $NMeHBH_2NMeHBH_3$. The *in situ* ^{11}B NMR of the resulting mixture, and GPC of the isolated polymeric material are shown below:

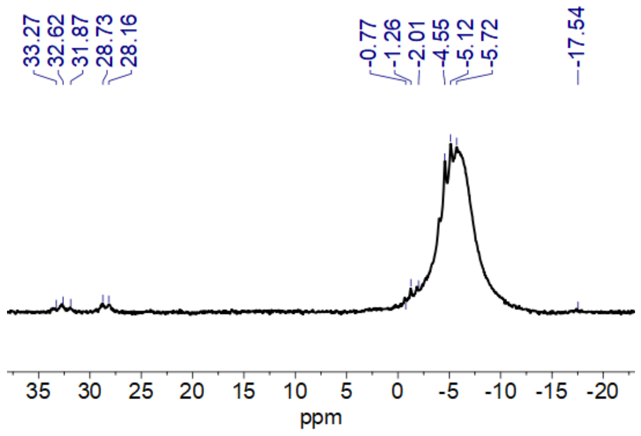

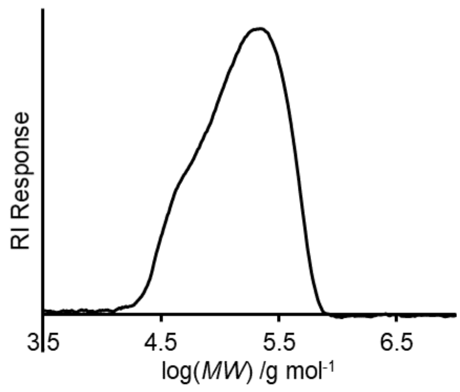

Figure S8 ^{11}B NMR spectrum at the end of catalysis and GPC trace of resulting isolated polymer from the dehydropolymerisation of $H_3B{\cdot}NMeH_2$ at 60 °C using **1-H_3**. Experimental conditions: 0.223 M $[H_3B{\cdot}NMeH_2]$ in 1,2-$C_6H_4F_2$, 333 K, 1 mol% 1-H_3 (30 min).

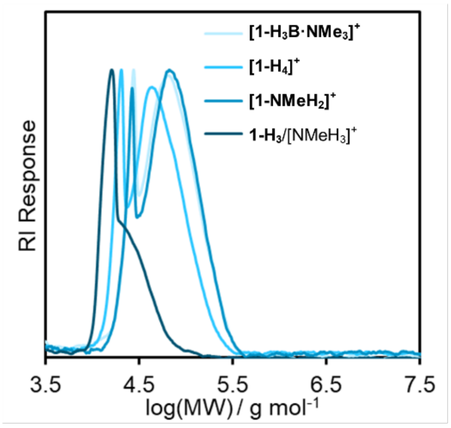

Figure S9 GPC trace obtained of polymer samples prepared with various pre-catalysts. Experimental conditions: 1 mol% **[cat]**, 0.223 M $H_3B{\cdot}NMeH_2$ in 1,2-$C_6H_4F_2$, jacketed-Schlenk, 293 K. All cationic species have $[BAr^F{}_4]^-$ anion.

1.4 Kinetics of Dehydropolymerisation and GPC traces

Throughout, the initial rate method is applied when determining k_{obs} from H_2 evolution experiments and linear regression applied to determine the error - this was consistently larger than the error associated with pre-catalyst solution concentration (propagated weight and volume).

1.4.1 Reaction Order plots

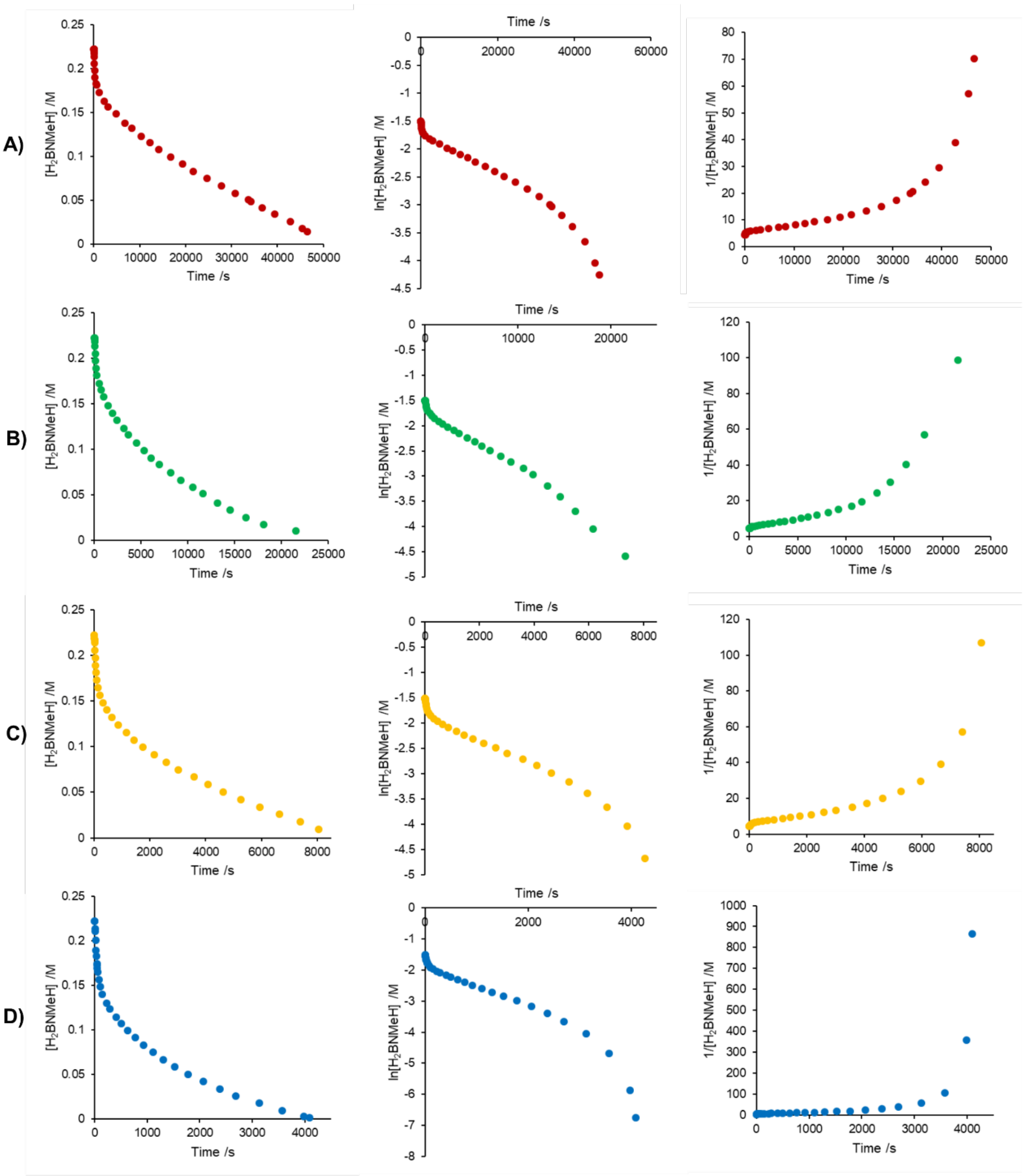

Figure S10 Reaction order rate plots (0th, 1st, 2nd) for dehydropolymerisation reactions carried out using [1-$H_3B{\cdot}NMe_3$][$BAr^F{}_4$] at loadings of: A) 0.5 mol%; B) 1.0 mol%; C) 1.5 mol% and D) 2.0 mol%.

1.4.2 VTNA

Kinetic analysis of the temporal data plots from H_2 evolution experiments at various catalyst loadings was also attempted using Variable Time Normalisation Analysis (VTNA).[S13]

In the case where **[1-$H_3B\cdot NMe_3$][$BAr^F{}_4$]** was used a pre-catalyst for the dehydropolymerisation reaction of [$H_3B\cdot NMeH_2$], VTNA analysis was carried out in two parts: during the initial "burst phase" clearly visible from the temporal plots and over the entire reaction course where catalytic activity is decreasing. However, in each, good overlap of the resulting VTNA plots was not obtained (zeroth, first and second order trialled).

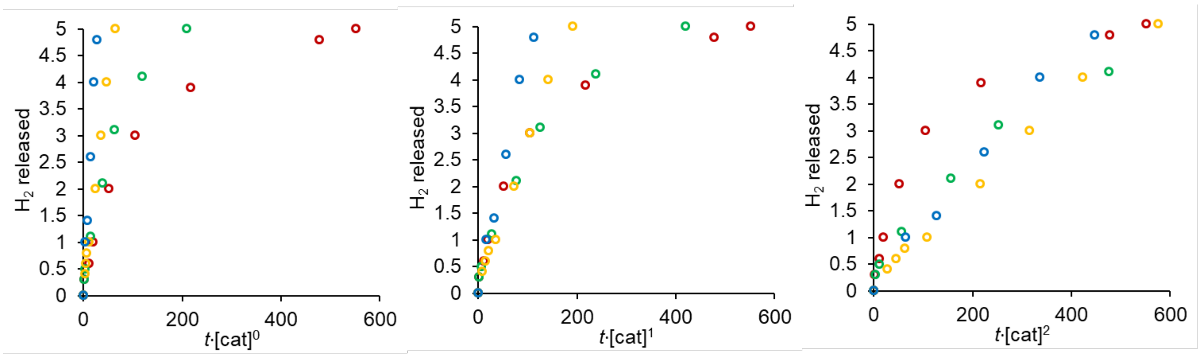

Figure S11 VTNA analysis of the temporal profile of dehydropolymerisation reactions (first 5 cm^3 H_2 evolved) carried out with **[1-$H_3B\cdot NMe_3$][$BAr^F{}_4$]** at various catalyst loadings (0.5 – 2.0 mol%).

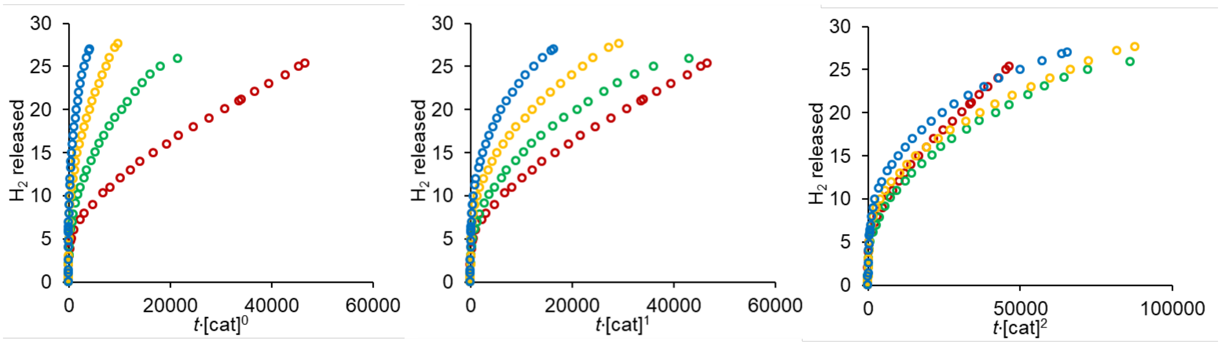

Figure S12 VTNA analysis of temporal profile of dehydropolymerisation reactions carried out with **[1-$H_3B\cdot NMe_3$][$BAr^F{}_4$]** at various catalyst loadings (0.5 – 2.0 mol%).

2. NMR Spectra

2.1 NMR Spectra for **[1-COD]Cl**

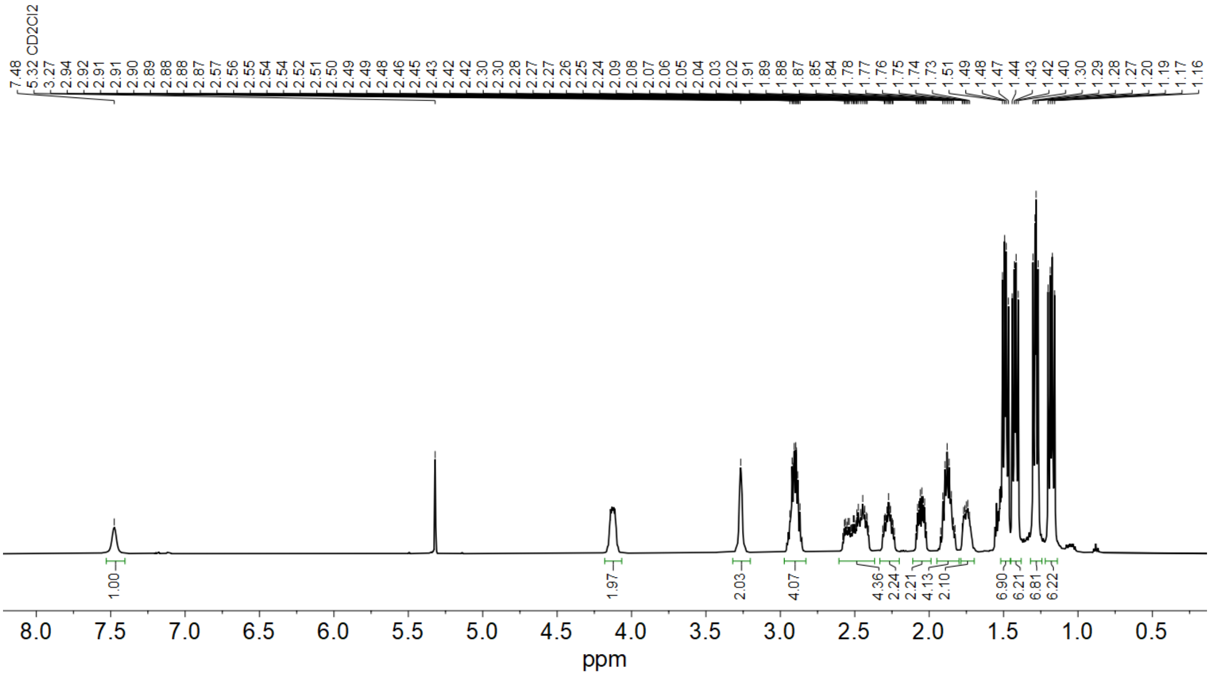

Figure S13 ^{1}H NMR (500 MHz, CD_2Cl_2) of **[1-COD]Cl**.

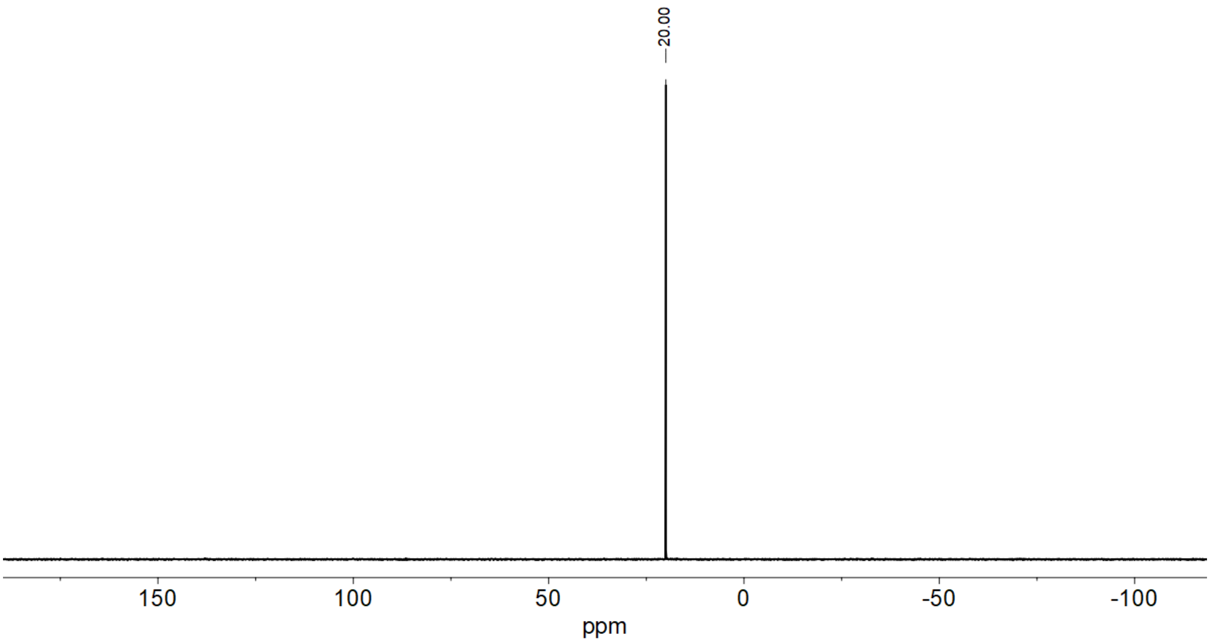

Figure S14 $^{31}P\{^{1}H\}$ NMR (203 MHz, CD_2Cl_2) of **[1-COD]Cl**.

2.2 NMR Spectra for **[1-COD][BAr^F$_4$]**

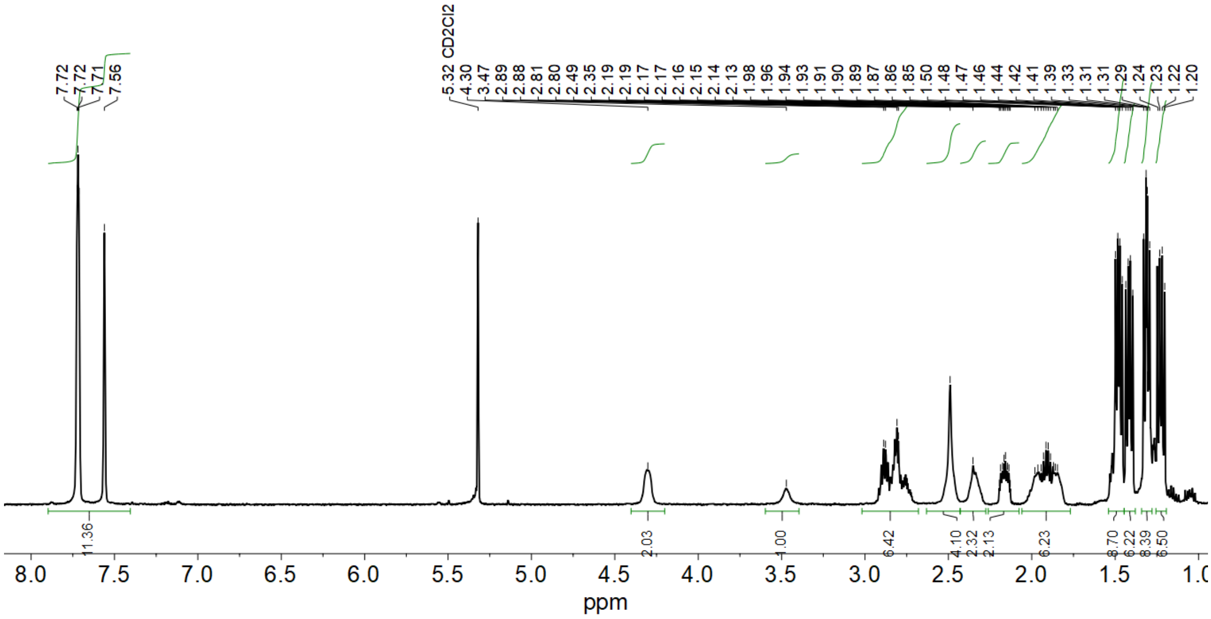

Figure S15 ^{1}H NMR (500 MHz, CD_2Cl_2) for **[1-COD][BAr^F$_4$]**.

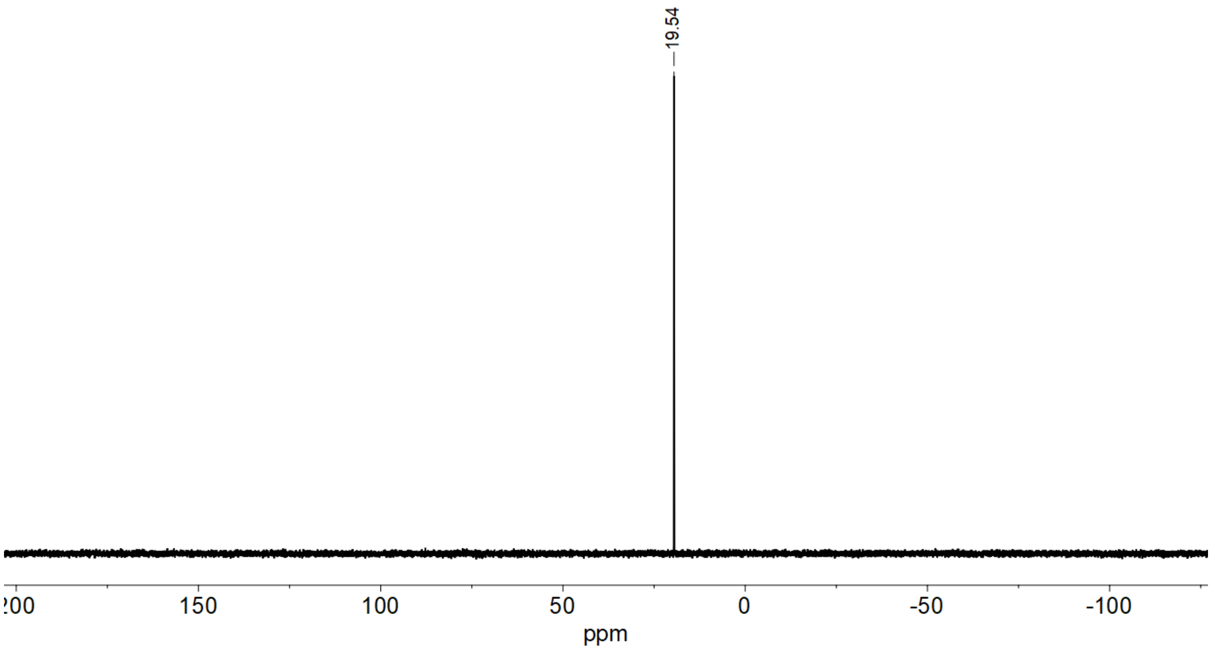

Figure S16 $^{31}P\{^1H\}$ NMR (203 MHz, CD_2Cl_2) for **[1-COD][BAr^F$_4$]**.

2.3 NMR Spectra for **[1-H$_4$][BAr^F$_4$]** *in situ*

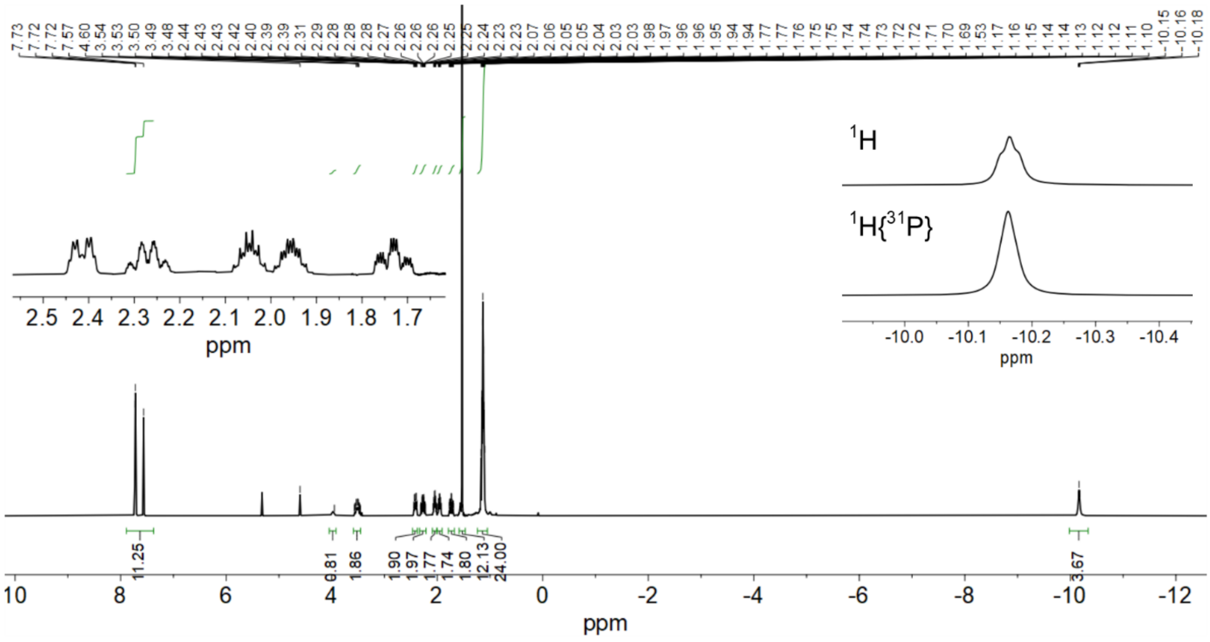

Figure S17 ^{1}H NMR (500 MHz, CD_2Cl_2) for **[1-H$_4$][BAr^F$_4$]** under a pressure of H_2(4 bar).

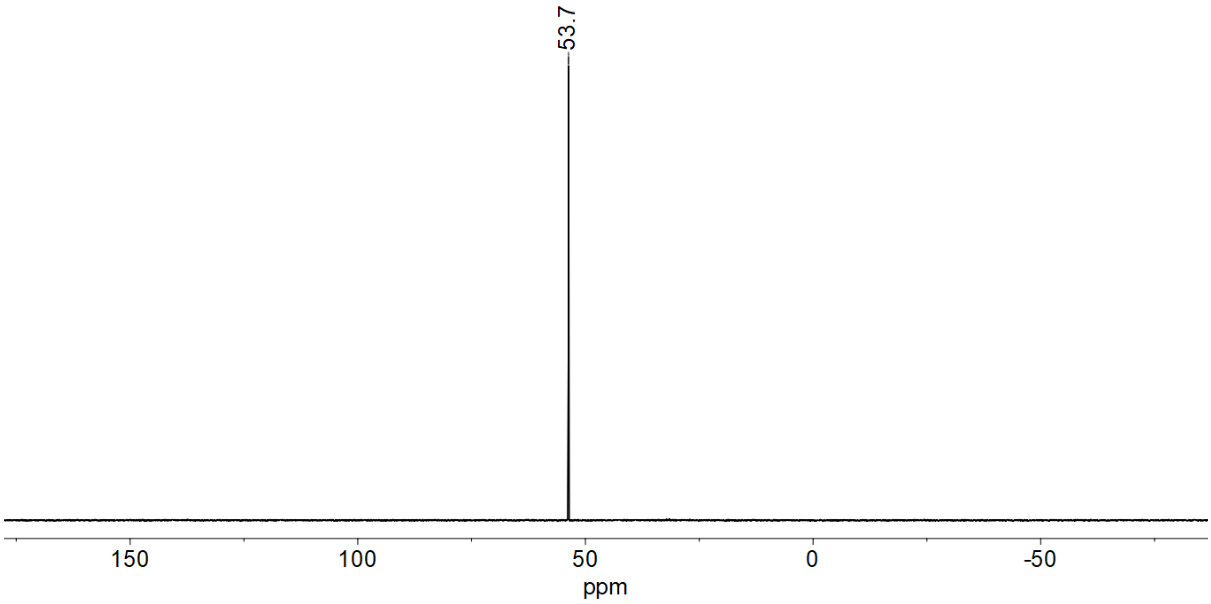

Figure S18 ^{31}P{^{1}H} NMR (203 MHz, CD_2Cl_2) for **[1-H$_4$][BAr^F$_4$]** under a pressure of H_2(4 bar).

In the absence of a hydrogen atmosphere, ~40 % of the sample of the sample was seen to begin to decompose over 10 minutes at 298 K.

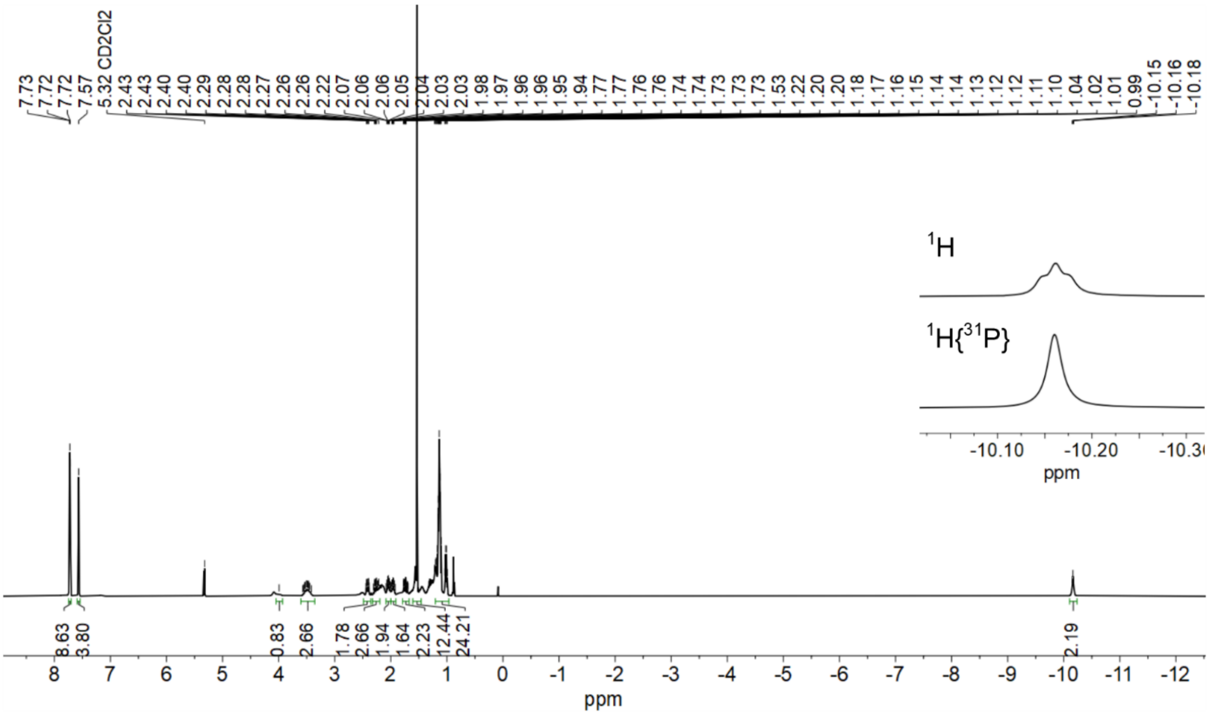

Figure S19 ^{1}H NMR (500 MHz, CD_2Cl_2) for **[1-H$_4$][BAr$^F{}_4$]** without a H_2 atmosphere.

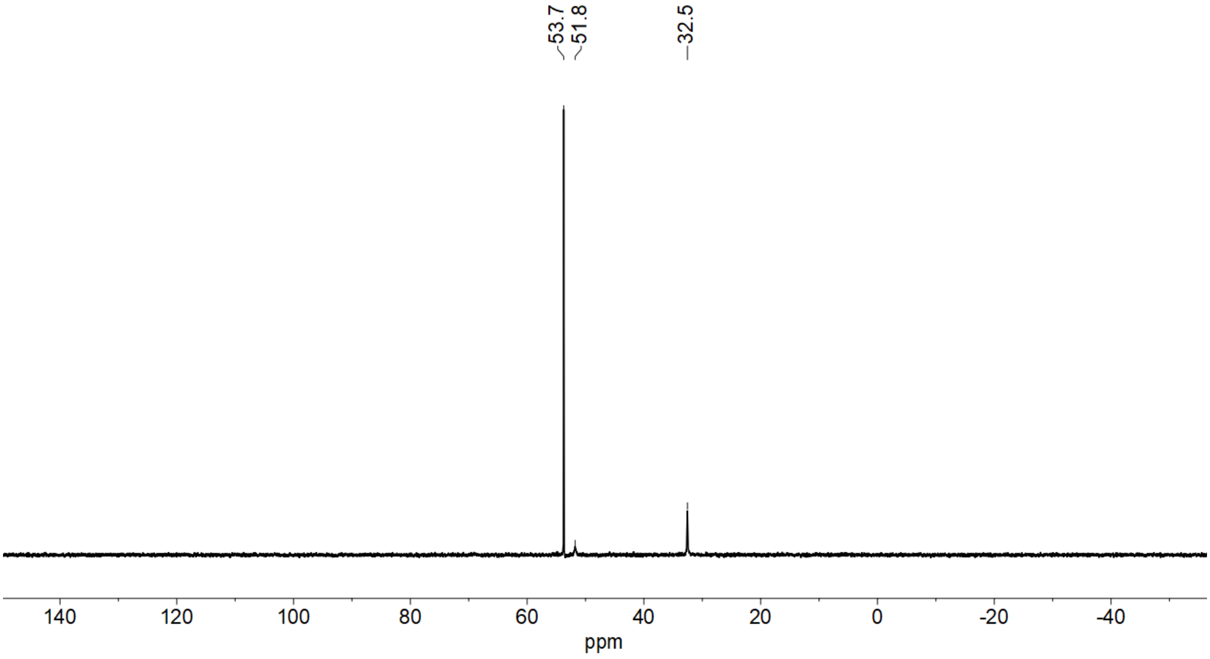

Figure S20 $^{31}P\{^{1}H\}$ NMR (203 MHz, CD_2Cl_2) for **[1-H$_4$][BAr$^F{}_4$]** without a H_2 atmosphere.

2.4 NMR Spectra for **[1-H$_3$B·NMe$_3$][BAr^F$_4$]**

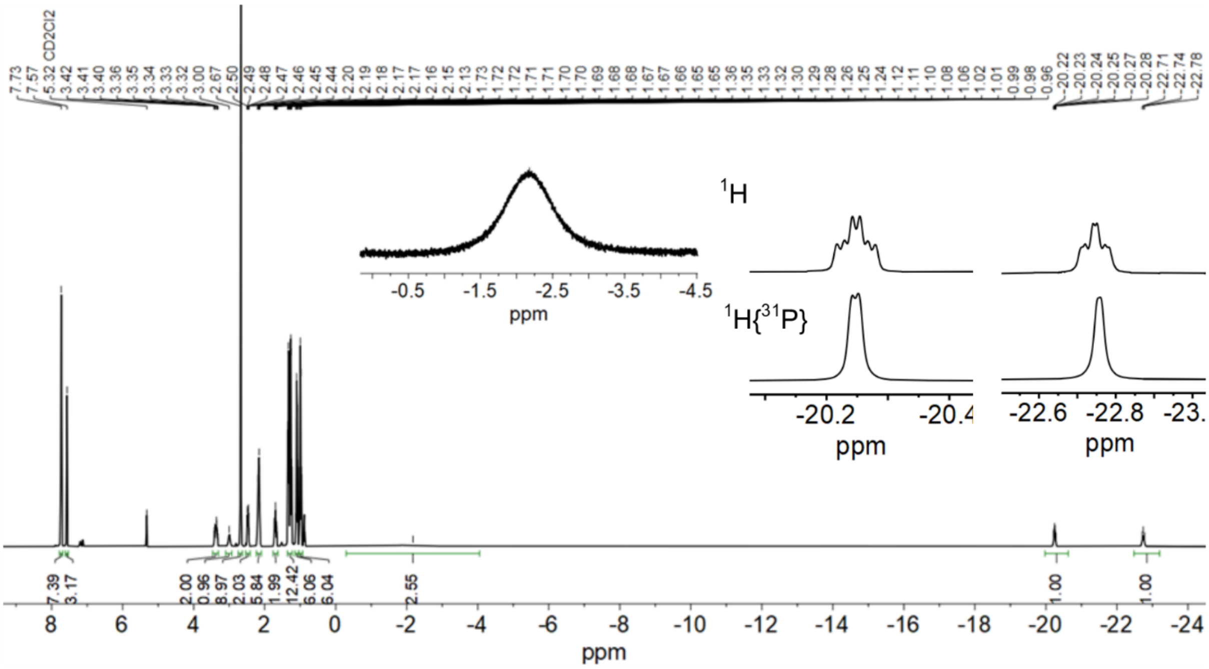

Figure S21 ^{1}H NMR (500 MHz, CD_2Cl_2) for **[1-H$_3$B·NMe$_3$][BAr^F$_4$].**

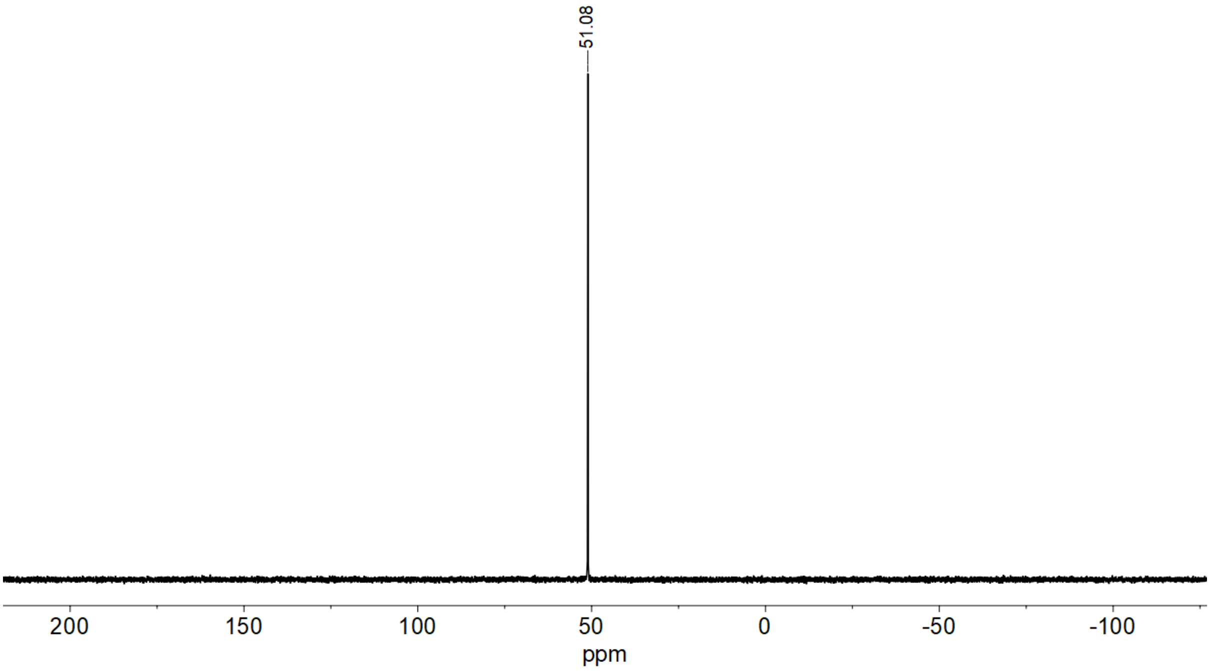

Figure S22 ^{31}P{^{1}H} NMR (203 MHz, CD_2Cl_2) for **[1-H$_3$B·NMe$_3$][BAr^F$_4$]**.

2.5 NMR Spectra for **[1-NMeH$_2$][BAr$^F_4$]**

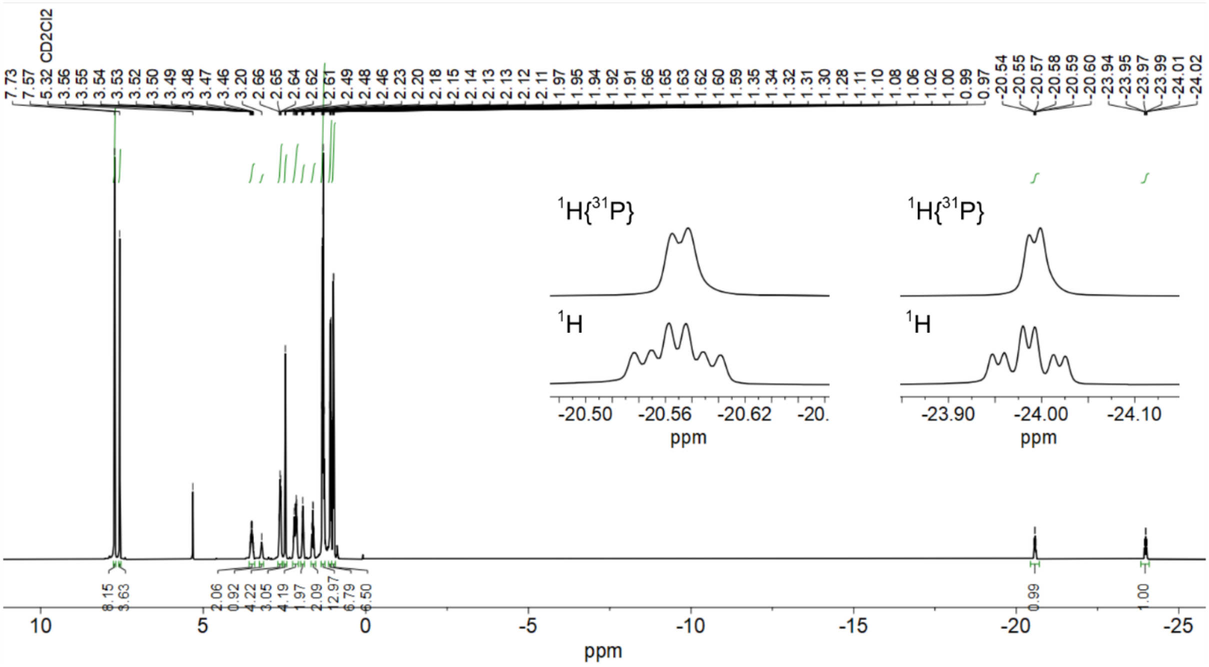

Figure S23 ^{1}H NMR spectrum (500 MHz, CD_2Cl_2) for **[1-NMeH$_2$][BAr$^F_4$]**.

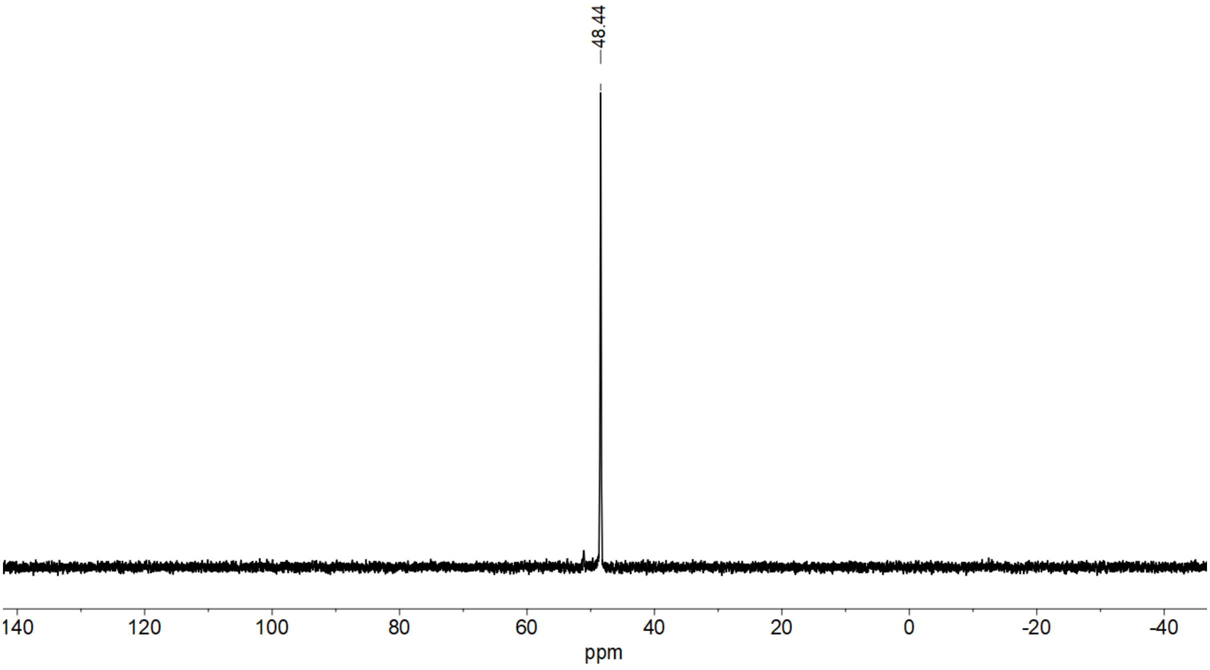

Figure S24 ^{31}P{^{1}H} NMR spectrum (203 MHz, CD_2Cl_2) for **[1-NMeH$_2$][BAr$^F_4$]**.

2.6 NMR Spectra for **1-H$_3$** *in situ*

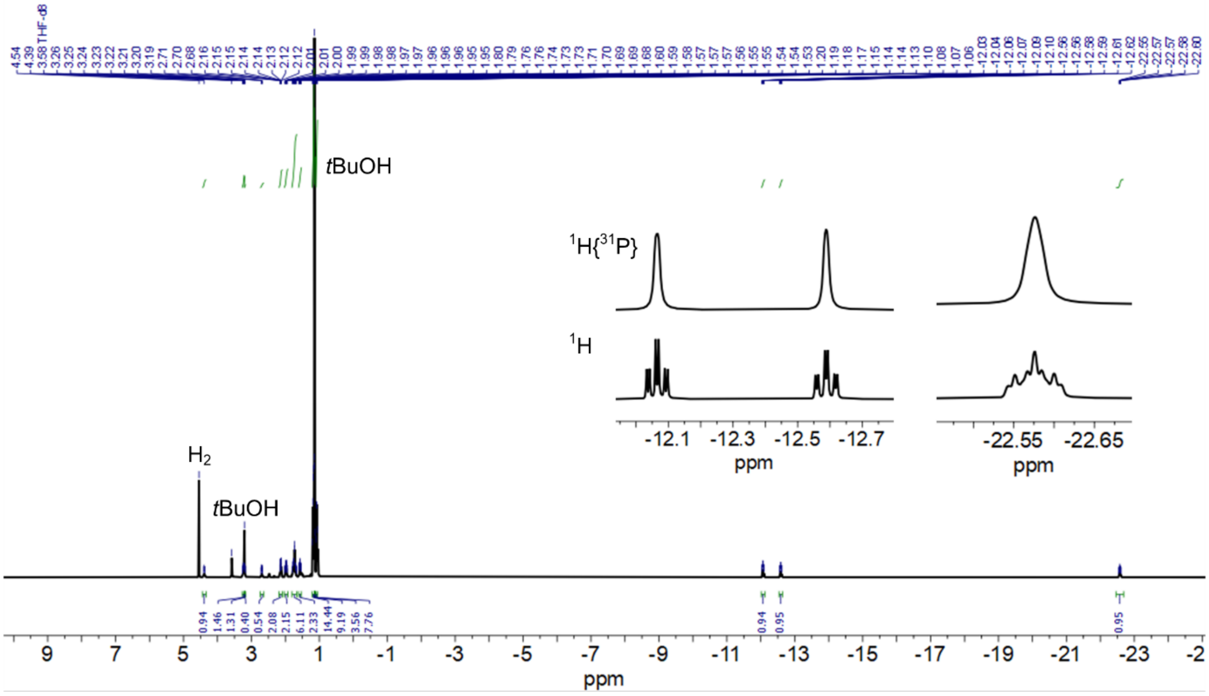

Figure S25 ^{1}H NMR (600 MHz, THF-d$_8$) spectrum for **1-H$_3$** produced *in situ* under a pressure of H_2 (2 bar).

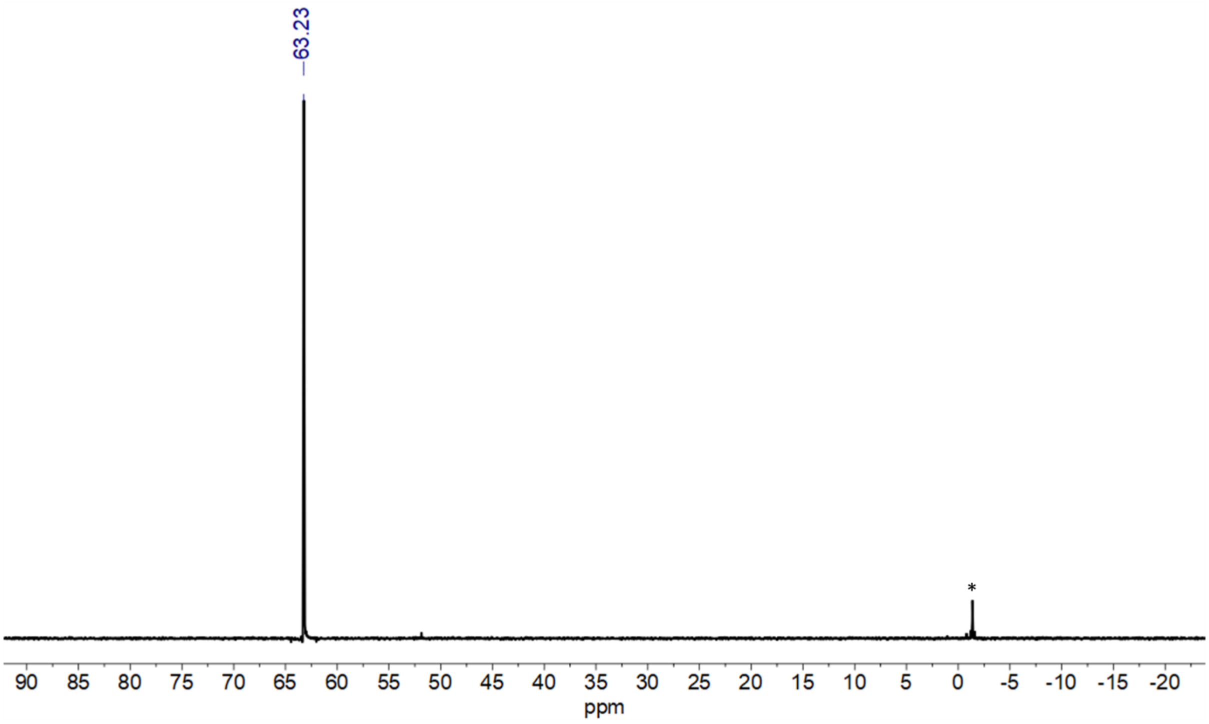

Figure S26 ^{31}P{^{1}H} NMR (243 MHz, THF-d$_8$) spectrum for **1-H$_3$** produced *in situ* under a pressure of H_2 (2 bar). * denotes *ca*. 4% {HN(CH$_2$CH$_2$P^{i}Pr$_2$)$_2$} impurity.

2.7 NMR spectra for solution state reactivity of **[1-L][BAr$^F_4$]** complexes

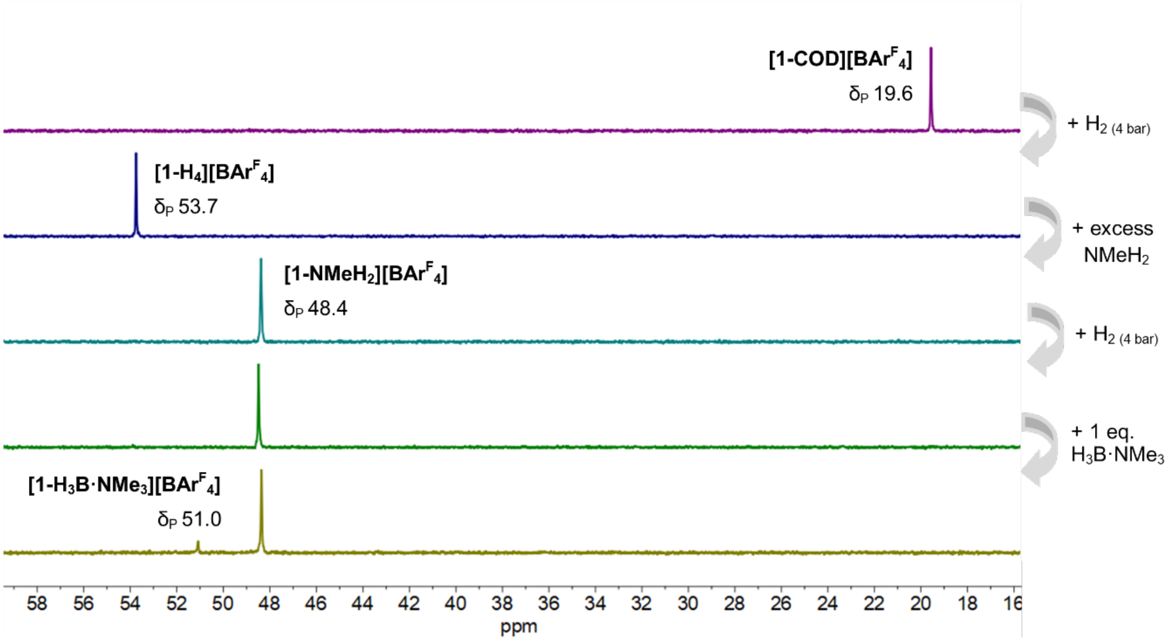

Figure S27 $^{31}P\{^1H\}$ NMR spectra (243 MHz, CD_2Cl_2) showing preparation of **[1-H$_4$][BAr$^F_4$]** from COD-hydrogenation, and subsequent substitution of σ-bound ligand within **[1-L][BAr$^F_4$]** complexes (where L is a σ-bound ligand).

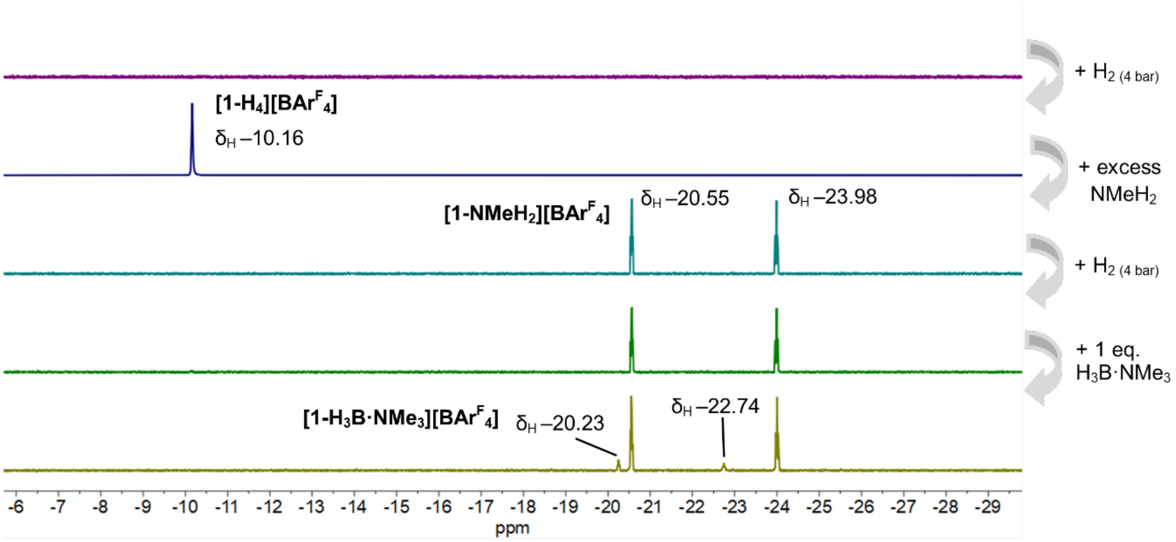

Figure 28 ^1H NMR spectra (600 MHz, CD_2Cl_2) showing preparation of **[1-H$_4$][BAr$^F_4$]** from COD-hydrogenation, and subsequent substitution of σ-bound ligand within **[1-L][BAr$^F_4$]** complexes (where L is a σ-bound ligand).

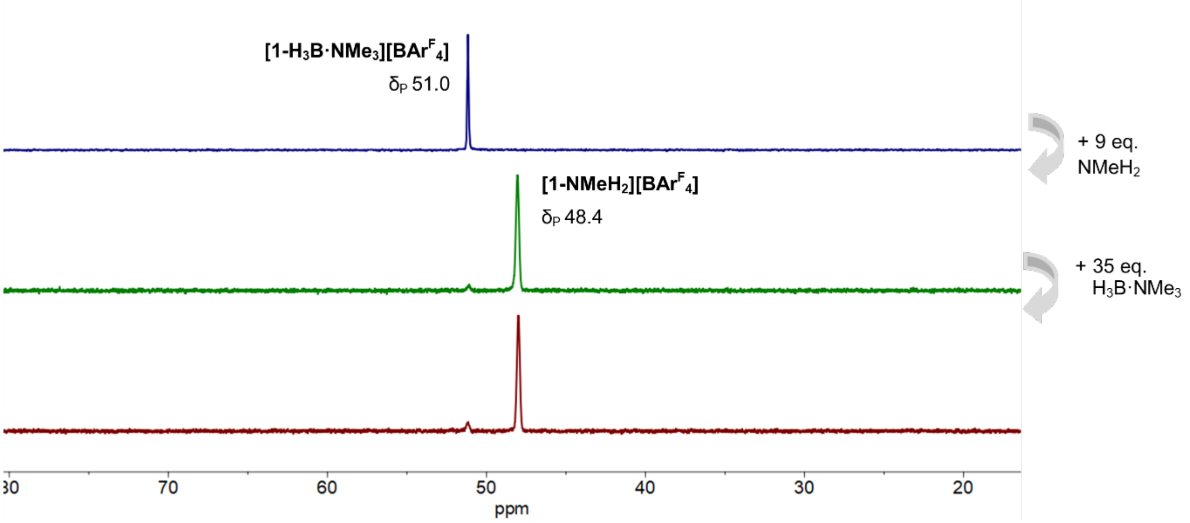

Figure S29 ${}^{31}P\{{}^{1}H\}$ NMR spectra (243 MHz, CD_2Cl_2) showing substitution of σ-bound ligand within **[1-L][BAr$^F{}_4$]** complexes (where L is a σ-bound ligand).

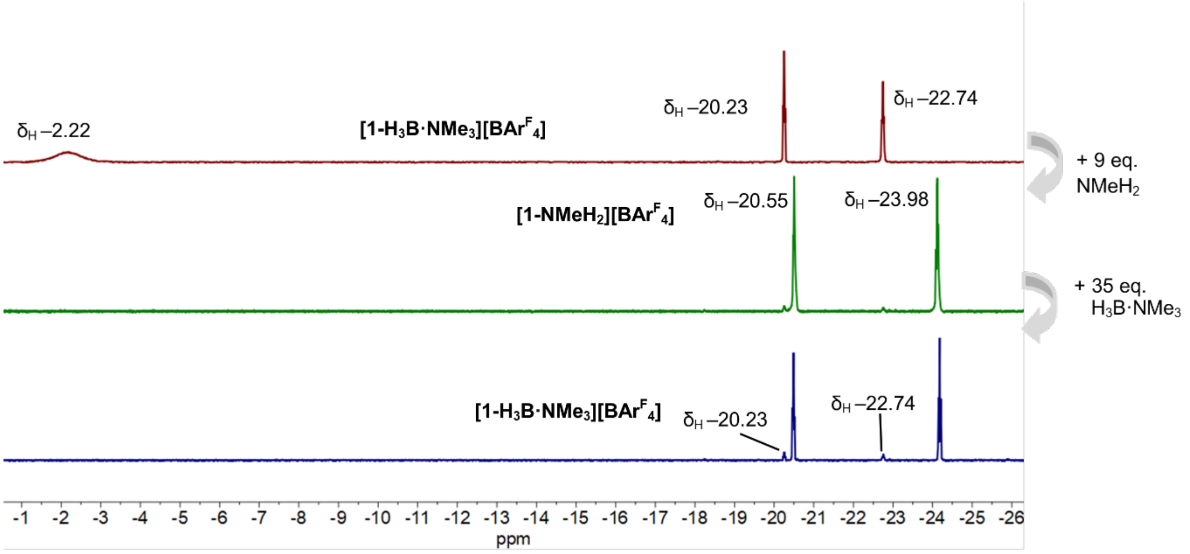

Figure S30 ${}^{1}H$ NMR spectra (600 MHz, CD_2Cl_2) showing substitution of σ-bound ligand within **[1-L][BAr$^F{}_4$]** complexes (where L is a σ-bound ligand).

2.9 VT NMR for **[1-H$_4$][BAr^F$_4$]** under a pressure of H_2 (4 bar at 298 K)

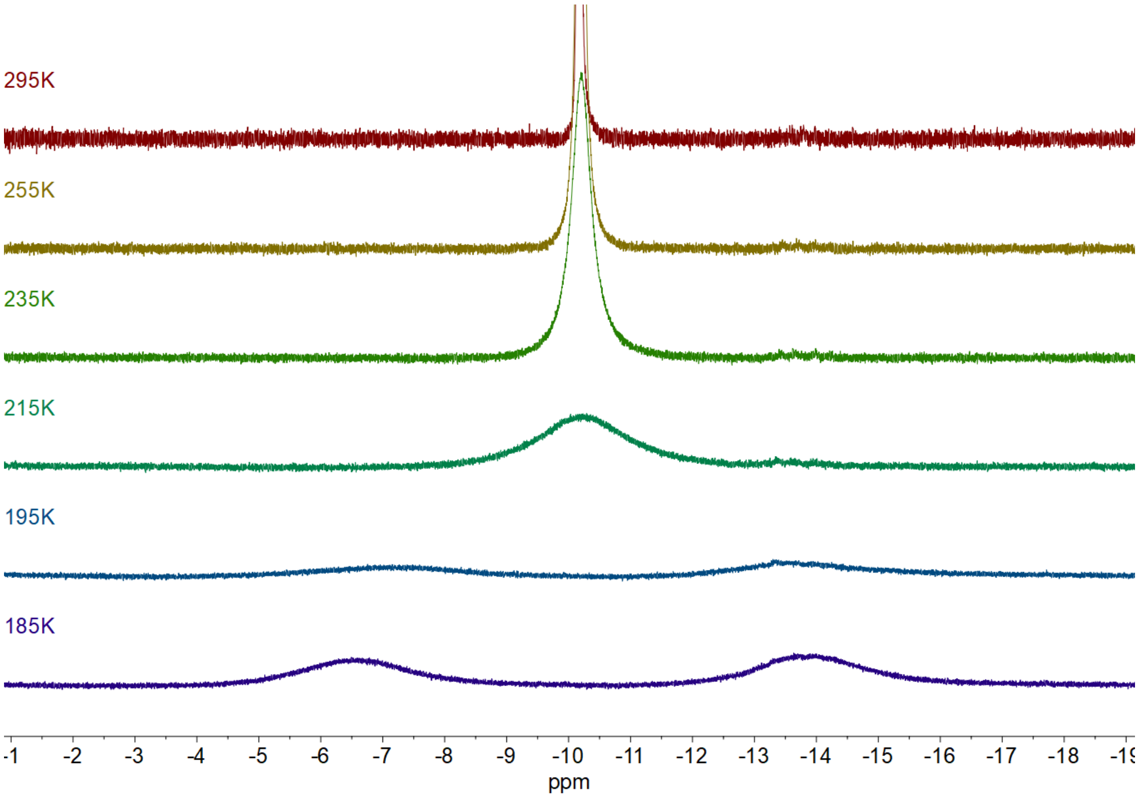

Figure S31 VT ^{1}H NMR spectra (CD_2Cl_2, 500 MHz) of **[1-H$_4$][BAr^F$_4$]** between 298 K and 185 K.

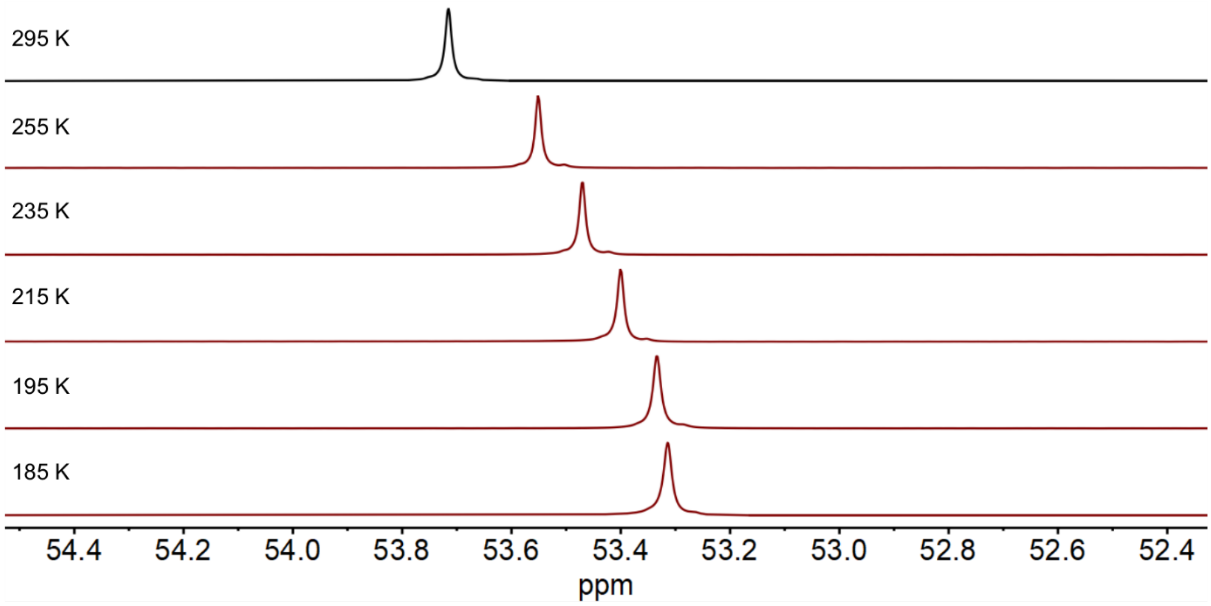

Figure S32 VT ^{31}P{^{1}H} NMR spectra (CD_2Cl_2, 203 MHz) of **[1-H$_4$][BAr^F$_4$]** between 298 K and 185 K.

2.10 VT NMR Spectra for **[1-H$_3$B·NMe$_3$][BAr^F$_4$]**

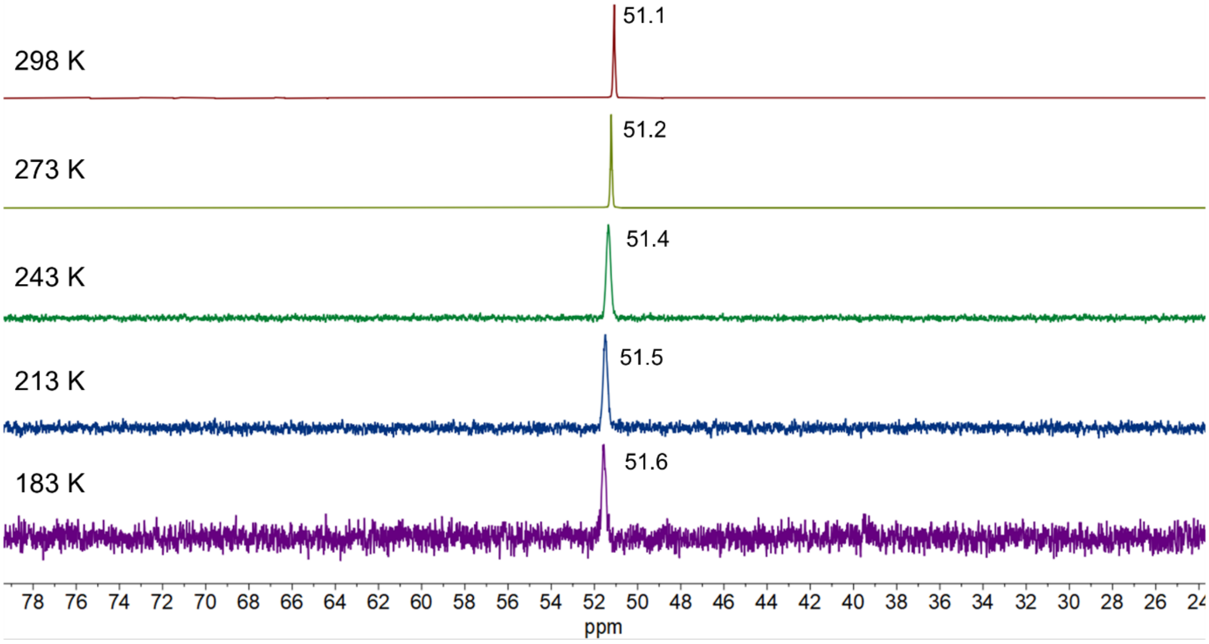

Figure S33 $^{31}P\{^1H\}$ VT-NMR (203 MHz, CD$_2$Cl$_2$) for **[1-H$_3$B·NMe$_3$][BAr^F$_4$]** between 298 K and 183 K.

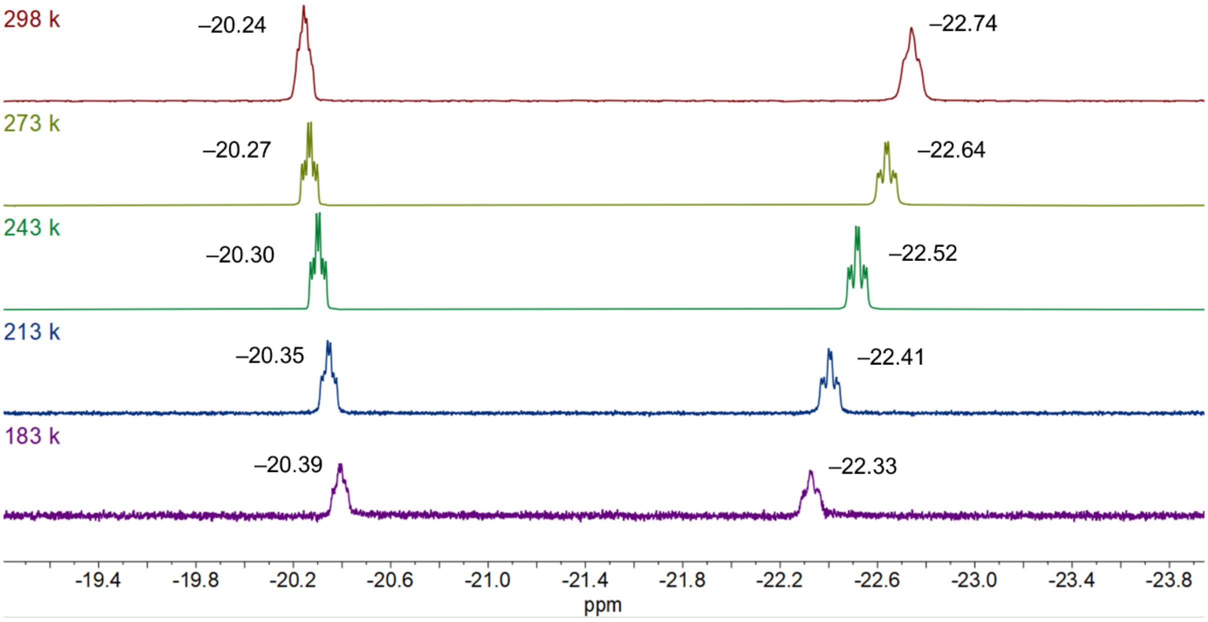

Figure S34 ^1H NMR (500 MHz, CD$_2$Cl$_2$) for **[1-H$_3$B·NMe$_3$][BAr^F$_4$]** between 298 K and 183 K.

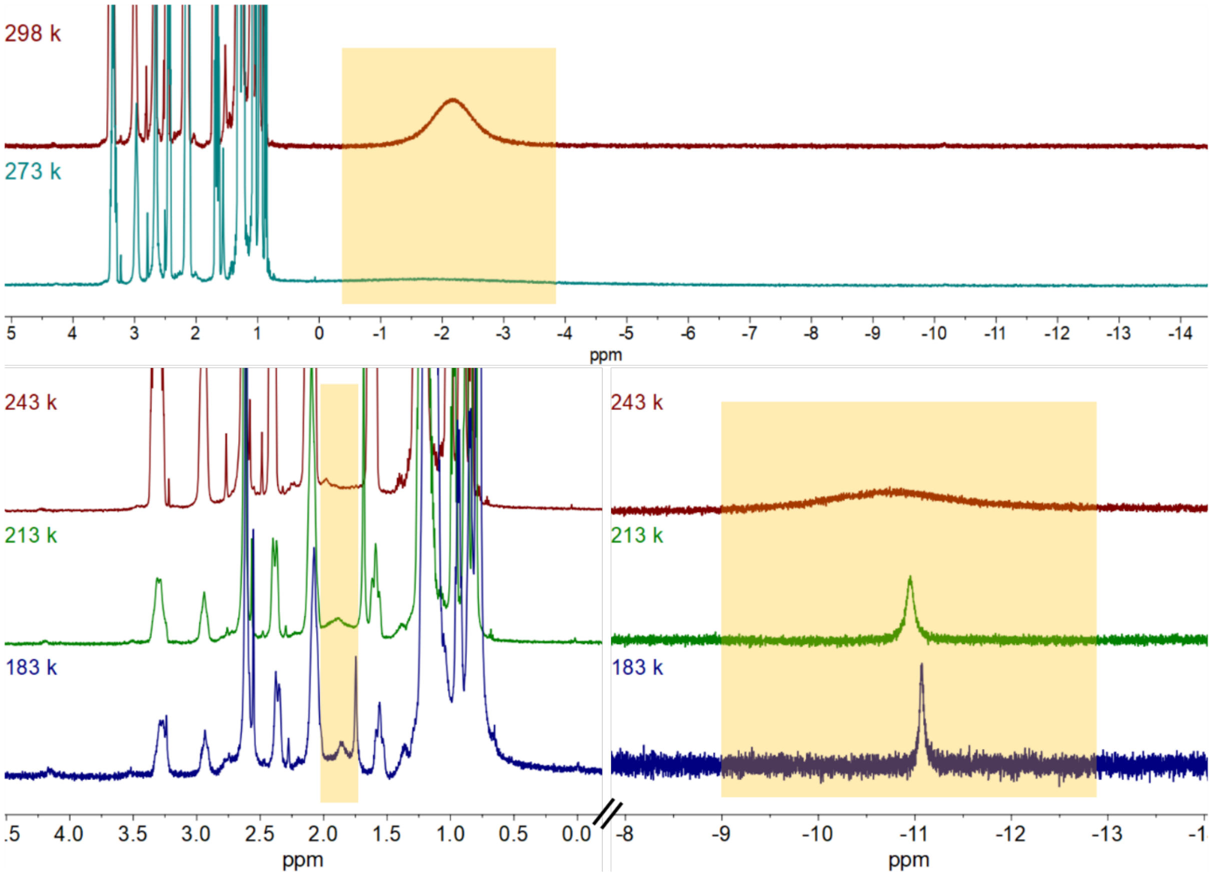

Figure S35 ^{1}H NMR (500 MHz, CD_2Cl_2) with BH_3 signals highlighted for **[1-H_3B·NMe_3][$BAr^F{}_4$]** between 298 K and 183 K.

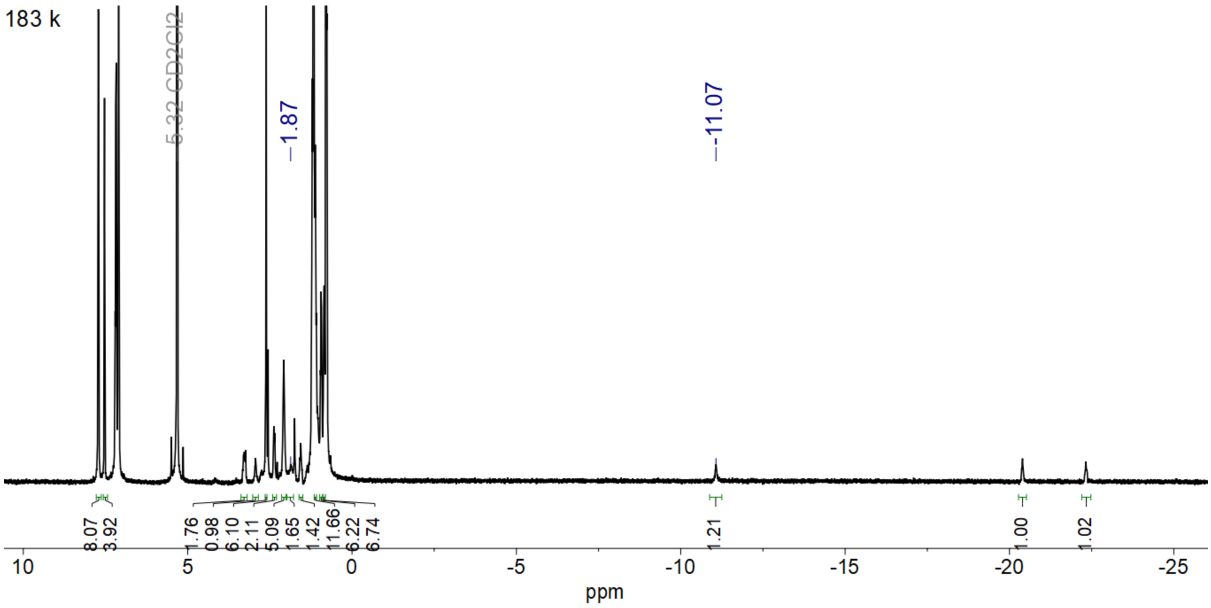

Figure S36 ^{1}H NMR (500 MHz, CD_2Cl_2) at 183 K for **[1-H_3B·NMe_3][$BAr^F{}_4$]**.

2.11 *in situ* NMR spectra for the dehydropolymerisaiton of $H_3B{\cdot}NMeH_2$

Typical experiment: To a jacketed-schlenk flask at 293 K, $H_3B{\cdot}NMeH_2$ (50 mg, 1.11 mol) (and, if required, additive at 2 mol% loading e.g. 19.9 mg $[NMeH_3][BAr^F_4]$) was added and dissolved in 4.5 mL 1,2-$C_6H_4F_2$. In a separate ampoule, 1.5 × precatalyst (e.g. **$[1\text{-}H_3B{\cdot}NMe_3][BAr^F_4]$**) was dissolved in 1,2-$C_6H_4F_2$ to a concentration of 0.0223 M L^{-1}. Where **1-H_3** was used a catalyst, this was prepared fresh before application and used as prepared in situ. Under a flow of argon, precatalyst solution (0.5 mL, 1 mol% [Ir]), was added to the amine-borane containing flask. Samples (0.5 mL, placed in an NMR tube fitted with a J-Youngs valve) were periodically taken at set time points, as determined from H_2 evolution experiments that has been recorded previously. Samples were immediately immersed in liquid nitrogen to halt the reaction. Spectra were collected on a Bruker 500 MHz NMR spectrometer that was pre-cooled to 245 K to prevent onward activity.

Table S4 Experiments performed for *in situ* Speciation observation.[a]

[cat.]	Additive
**$[1\text{-}H_3B{\cdot}NMe_3][BAr^F_4]$**	-
**$[1\text{-}H_3B{\cdot}NMe_3][BAr^F_4]$**	$[NMeH_3][BAr^F_4]$
**$[1\text{-}H_3B{\cdot}NMe_3][BAr^F_4]$**	$NMeH_2$ [b]
**1-H_3**	-
**1-H_3**	$[NMeH_3][BAr^F_4]$

[a] Experimental conditions: 0.223 M $[H_3B{\cdot}NMe_3]$ in 1,2-$C_6H_4F_2$. 1 mol% [cat.], 2 mol% additive, 293 K in a jacketed-schlenk under continuous flow of argon. [b] 1 mol% additive.

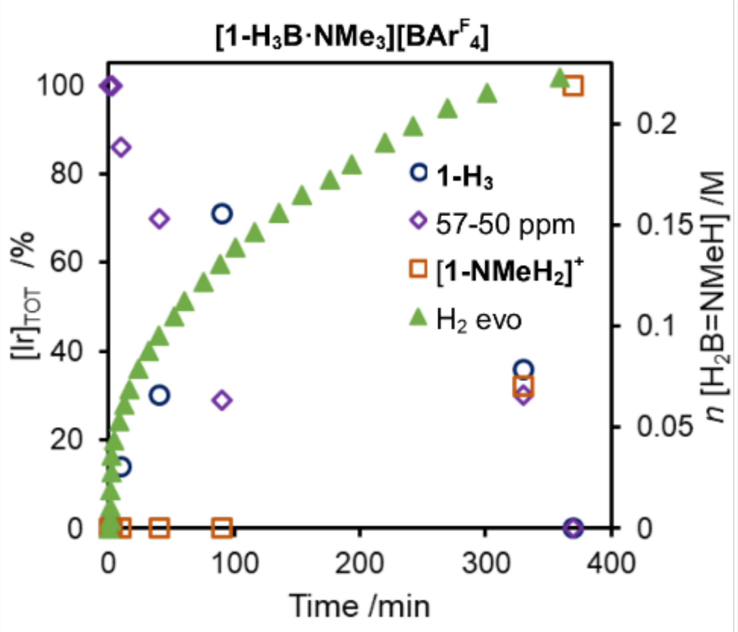

Figure S37 Summary of observed *in situ* speciation ($^{31}P\{^1H\}$ and ^1H NMR) under various conditions.

2.11.1 *in situ* spectra for dehydropolymerisation of $H_3B{\cdot}NMeH_2$ using **[1-H$_3$B·NMe$_3$]**

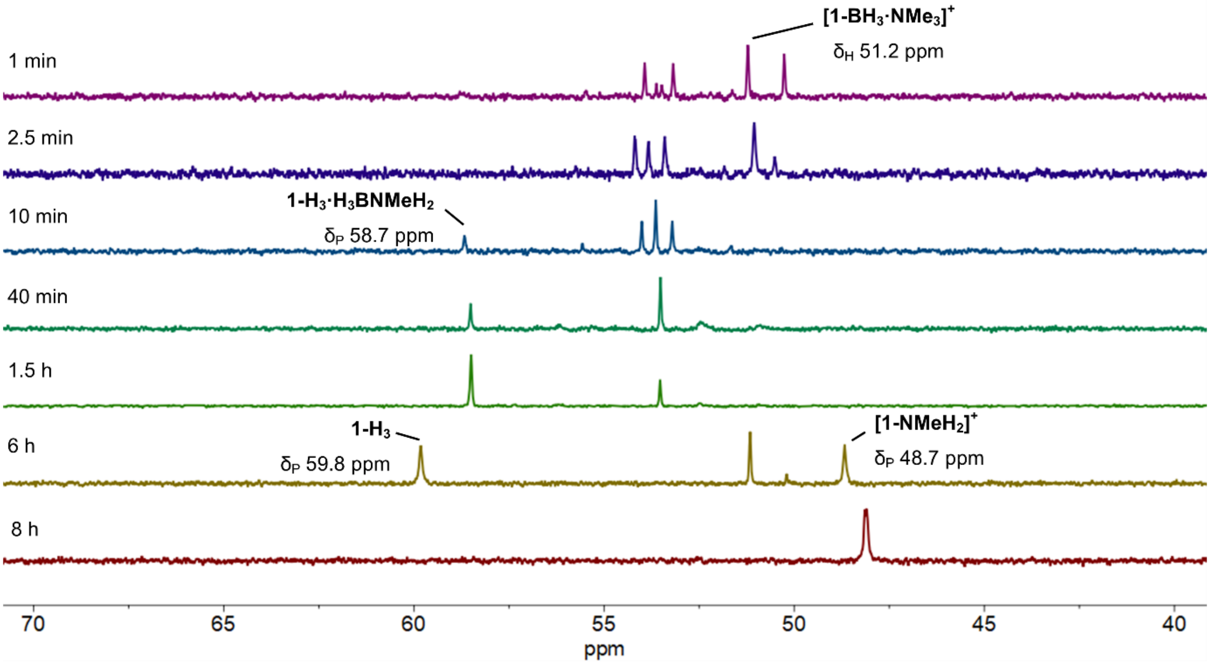

Figure S38 $^{31}P\{^1H\}$ NMR spectra (1,2-$C_6H_4F_2$, 245 K, 203 MHz).

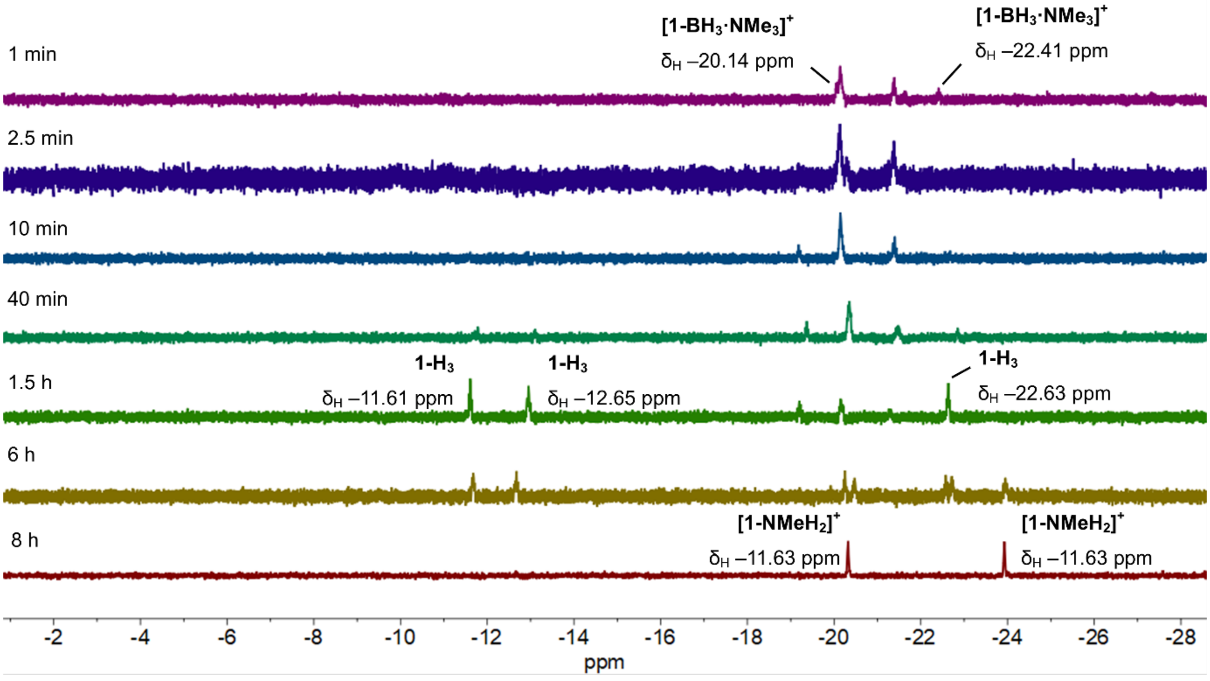

Figure S39 Hydride region, ^1H NMR spectra (1,2-$C_6H_4F_2$, 245 K, 500 MHz).

2.11.5 Typical *in situ* NMR spectra at the end of catalysis

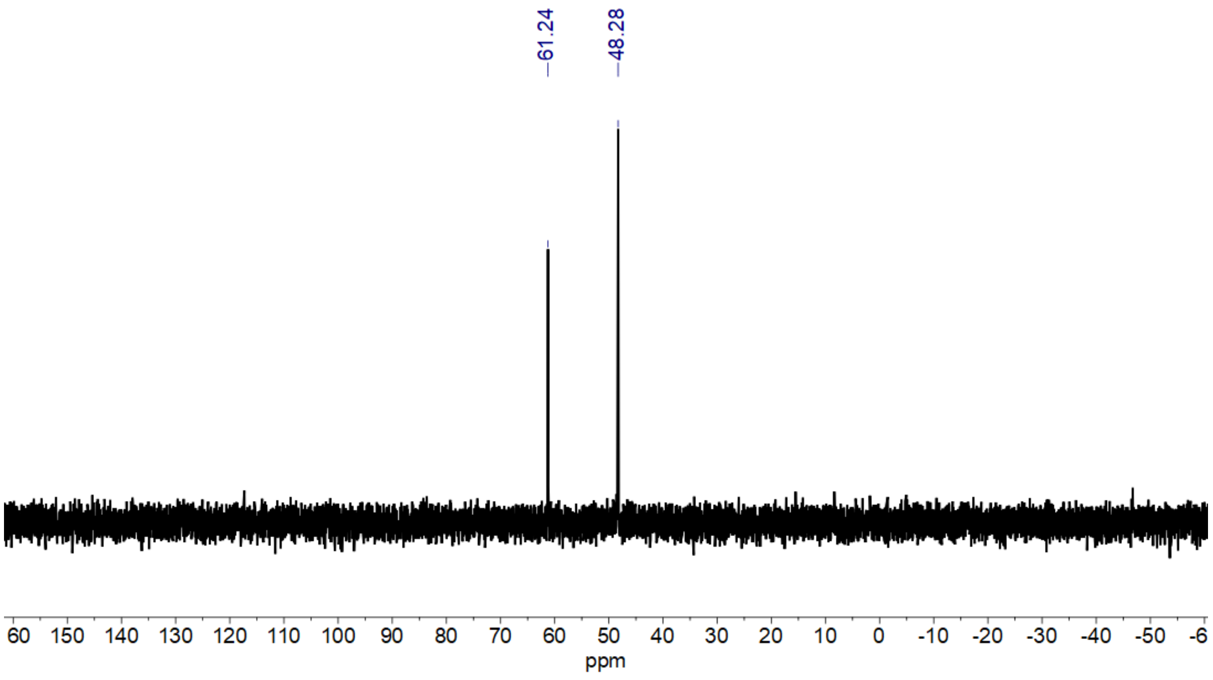

Figure S40 Typical $^{31}P\{^{1}H\}$ NMR spectrum (243 MHz, 1,2-$C_6H_4F_2$) obtained at the end of catalysis at 95-99% conversion.

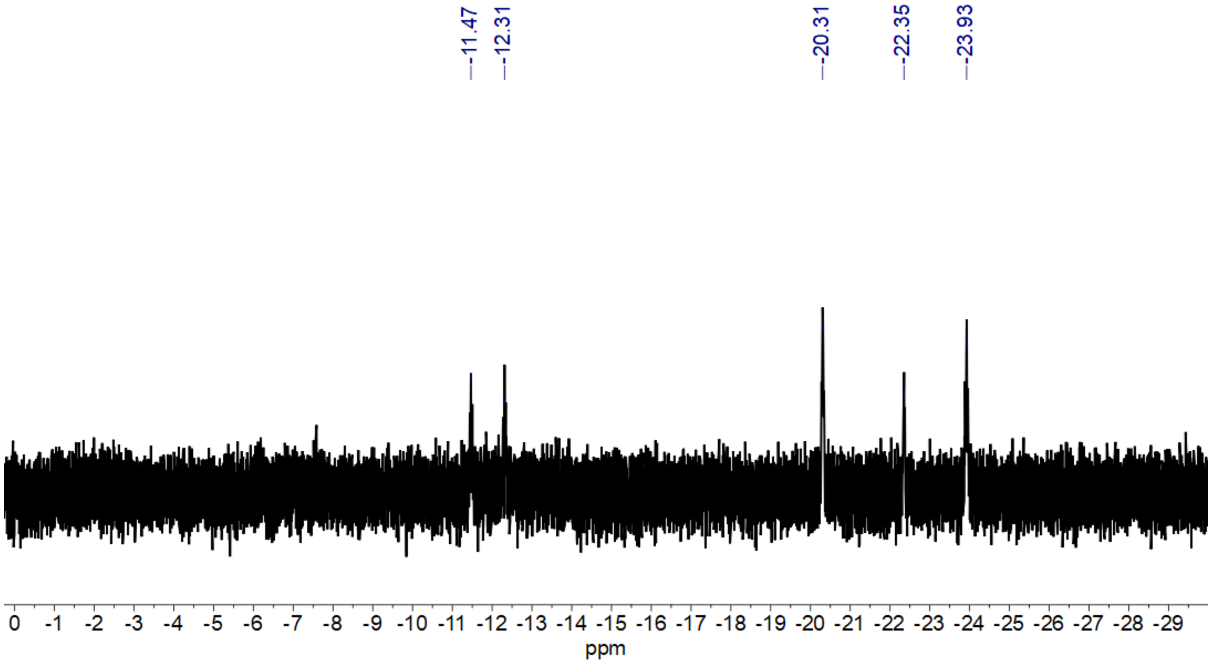

Figure S41 Typical ^{1}H NMR spectrum (hydride region, 600 MHz, 1,2-$C_6H_4F_2$) obtained at the end of catalysis at 95-99% conversion.

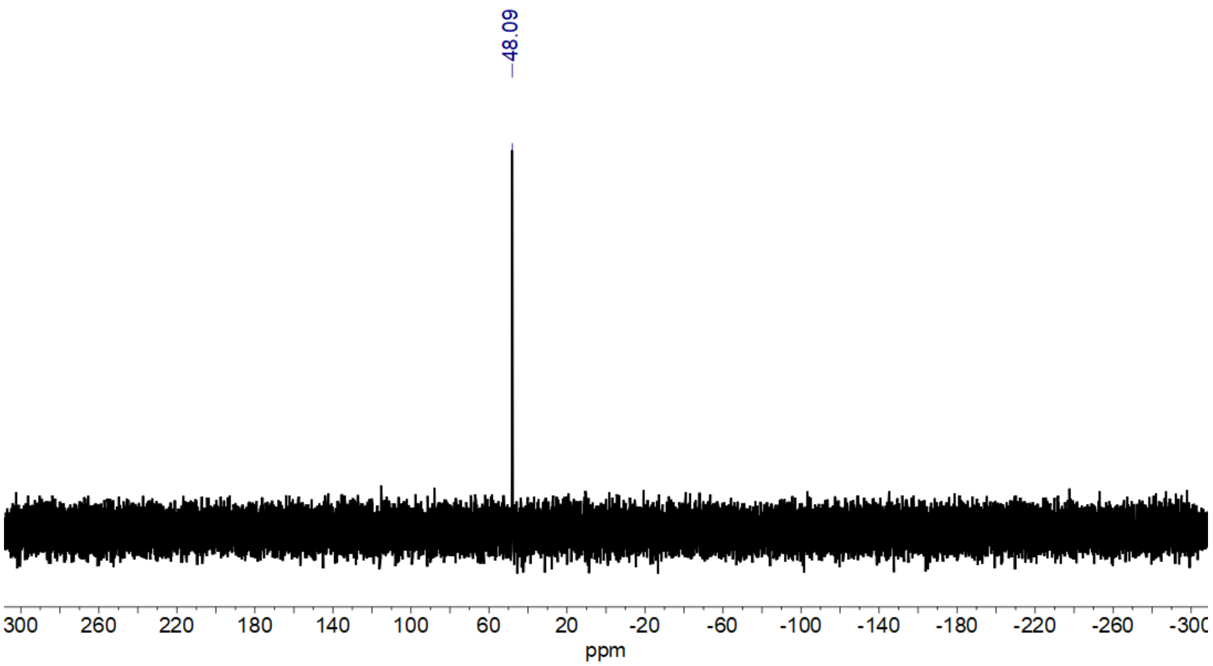

Figure S42 Typical $^{31}P\{^{1}H\}$ NMR spectrum (243 MHz, 1,2-$C_6H_4F_2$) obtained at the end of catalysis at 100% conversion.

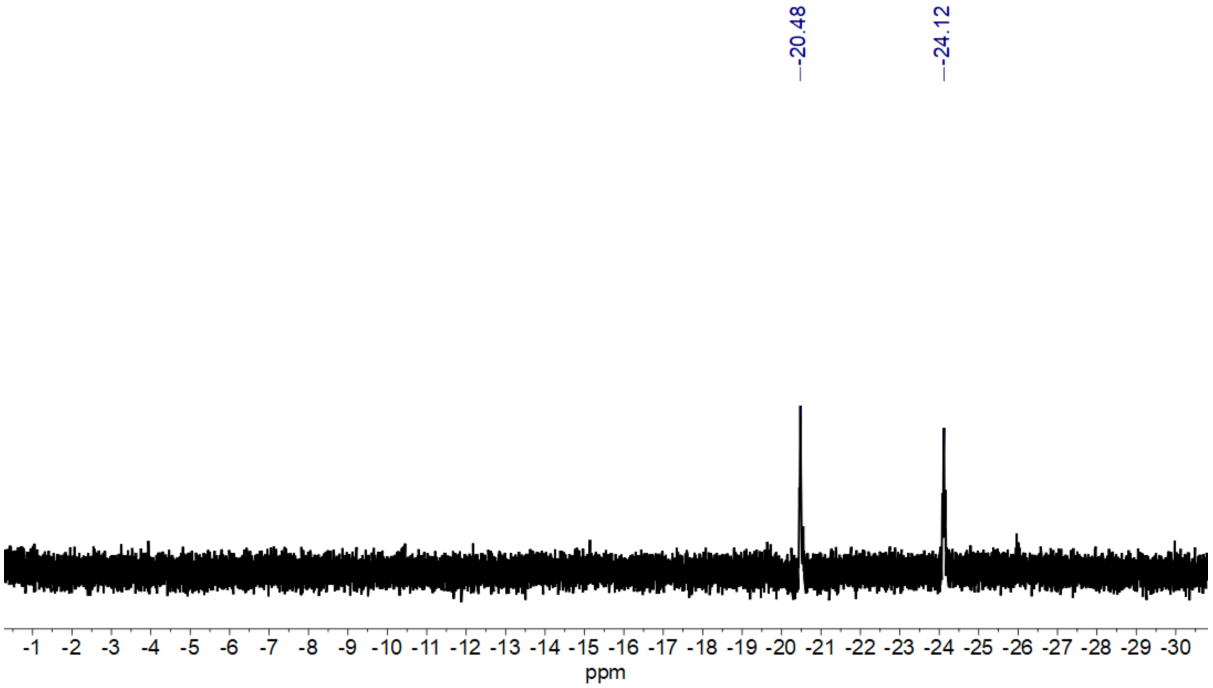

Figure S43 Typical ^{1}H NMR spectrum (hydride region, 600 MHz, 1,2-$C_6H_4F_2$)) obtained at the end of catalysis at 100% conversion.

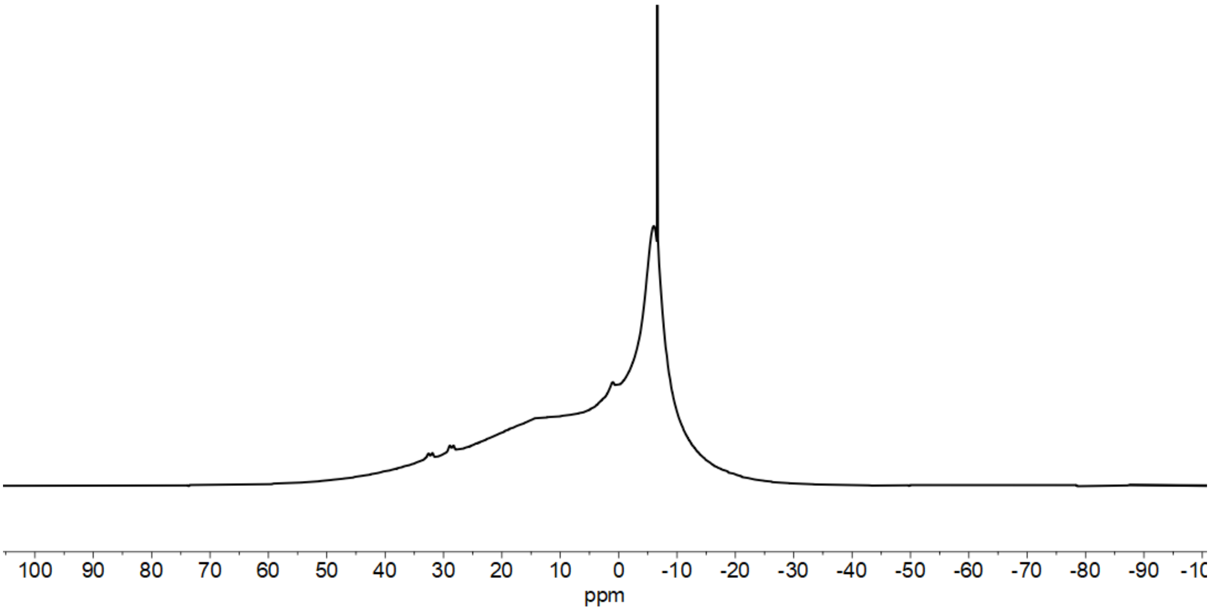

Figure S44 Typical ^{11}B NMR spectrum (193 MHz, 1,2-$C_6H_4F_2$) obtained at the end of catalysis (100% conversion) before baseline correction.

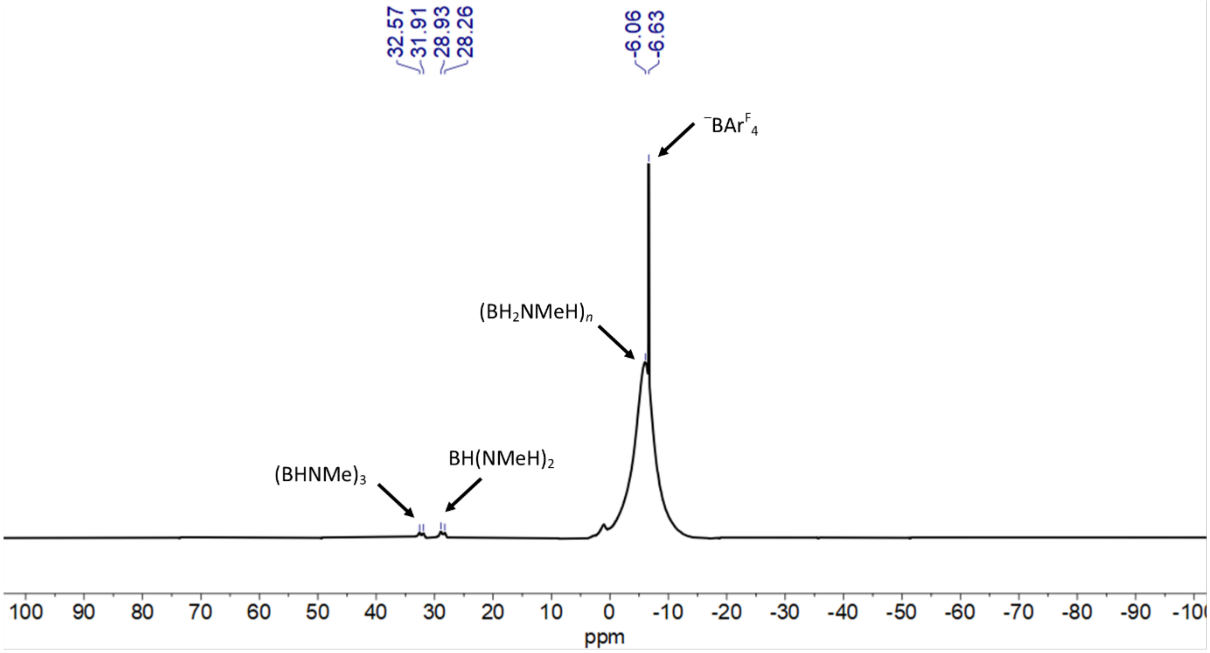

Figure S45 Typical ^{11}B NMR spectrum (193 MHz, 1,2-$C_6H_4F_2$) obtained at the end of catalysis (100% conversion) after baseline correction.

2.12 NMR Spectra for **[2-COD][BArF4]**

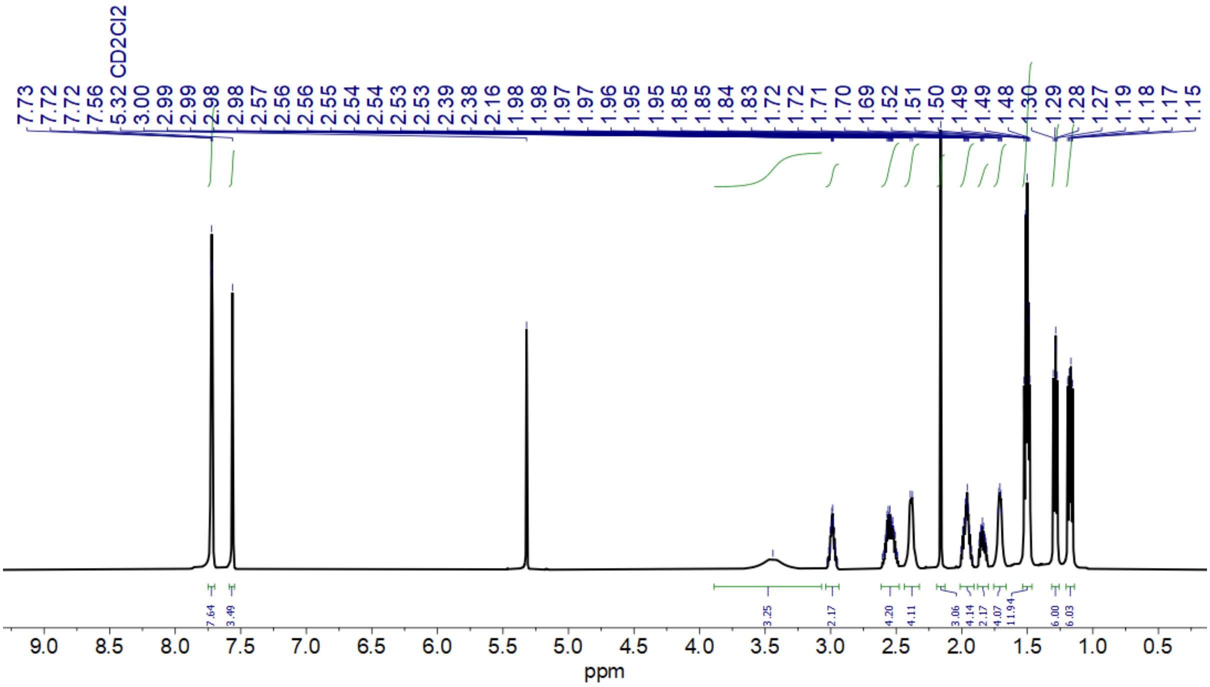

Figure S46 ^{1}H NMR (600 MHz, CD_2Cl_2) for **[2-COD][BAr$^F_4$]**.

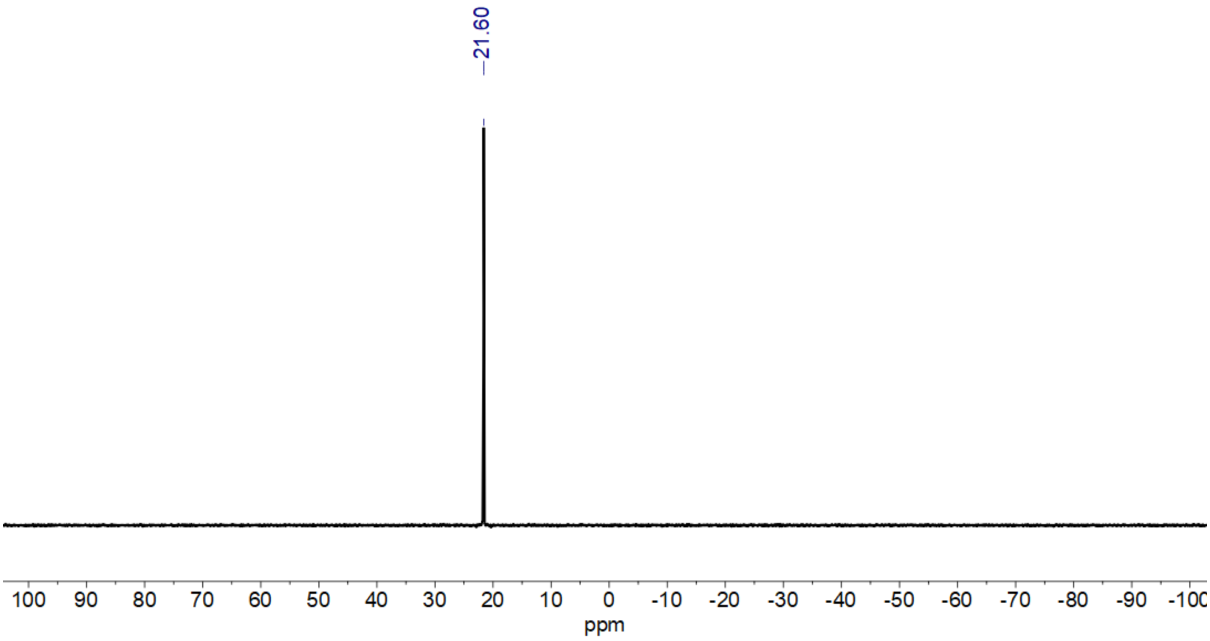

Figure S47 ^{31}P{^{1}H} NMR (243 MHz, CD_2Cl_2) for **[2-COD][BAr$^F_4$]**.

2.13 NMR Spectra for **[2-H$_3$B·NMe$_3$][BAr^F$_4$]**

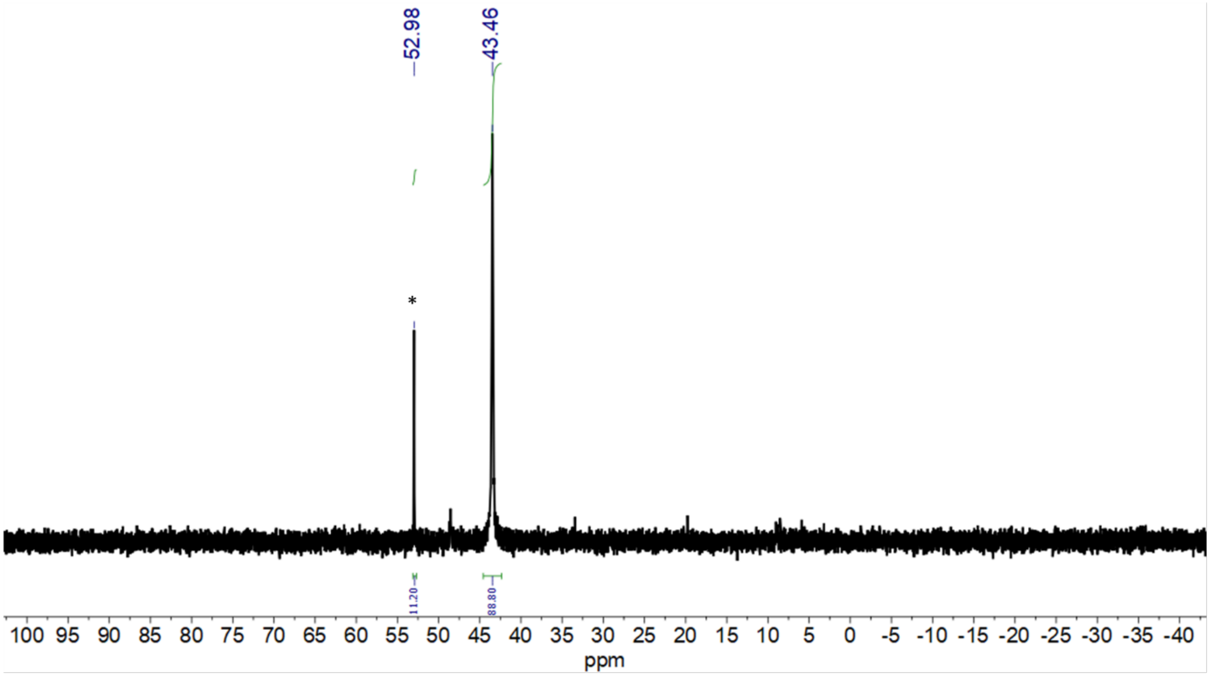

Figure S48 ^{31}P{^{1}H} NMR (243 MHz, CD_2Cl_2) for **[2-H$_3$B·NMe$_3$][BAr^F$_4$]**. *denotes an **[2-H$_4$][BAr^F$_4$]** impurity.

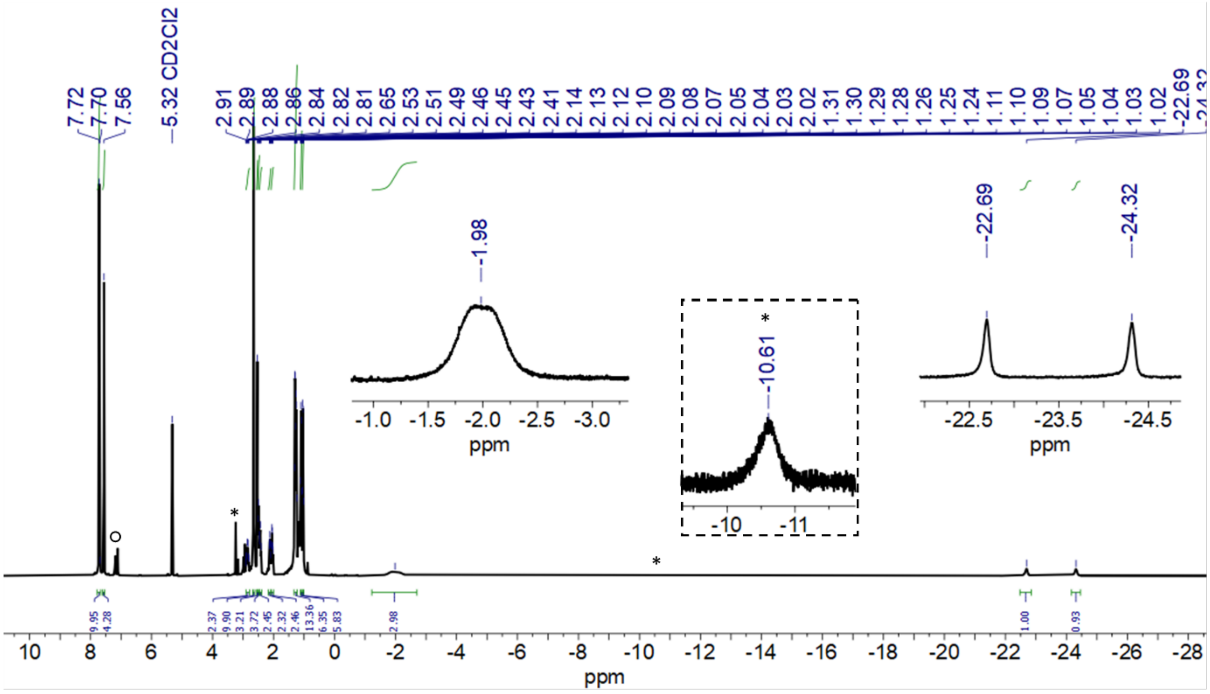

Figure S49 ^{31}P{^{1}H} NMR (243 MHz, CD_2Cl_2) for **[2-H$_3$B·NMe$_3$][BAr^F$_4$]**. *denotes an **[2-H$_4$][BAr^F$_4$]** impurity, °denotes an 1,2-$C_6H_4F_2$ impurity (which is persistent and is also observed within the crystal lattice in solid-state X-ray studies).

2.14 NMR Spectra for **[2-H4][BArF4]** *in situ*

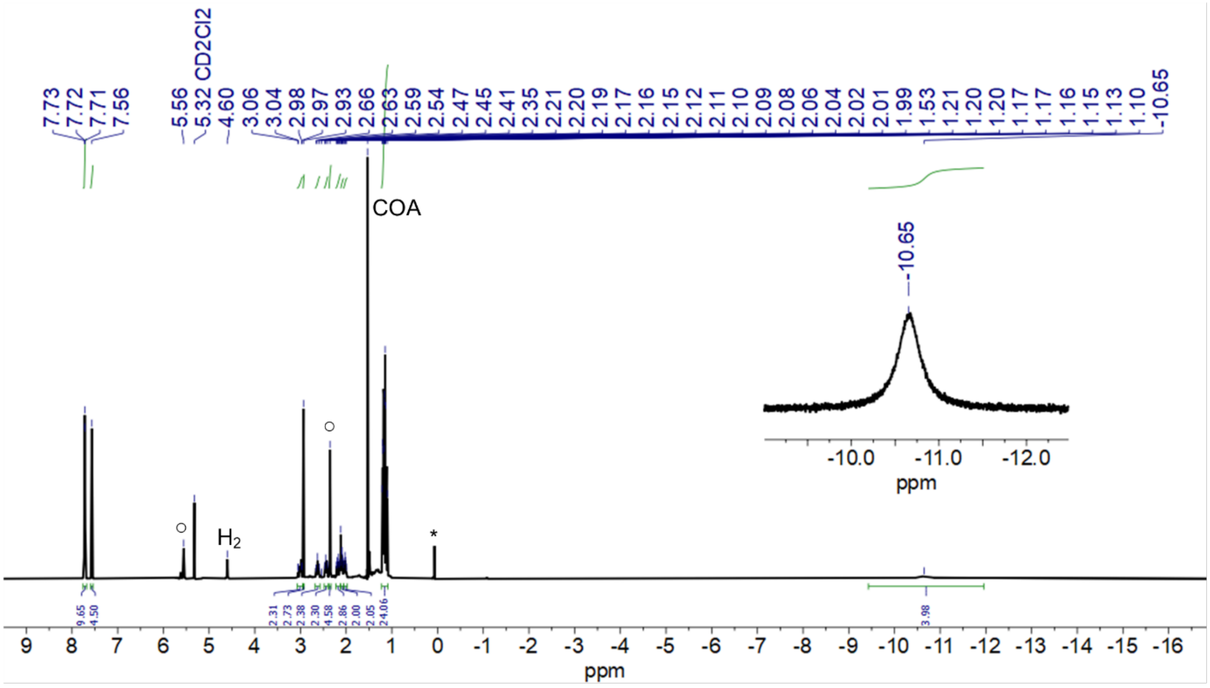

Figure S50 ^{1}H NMR (500 MHz, CD_2Cl_2, 298 K) for **[2-H4][BArF4]** under a pressure of H_2 (4 bar). ○denotes free 1,5-COD; *denotes grease. COA = cyclooctane.

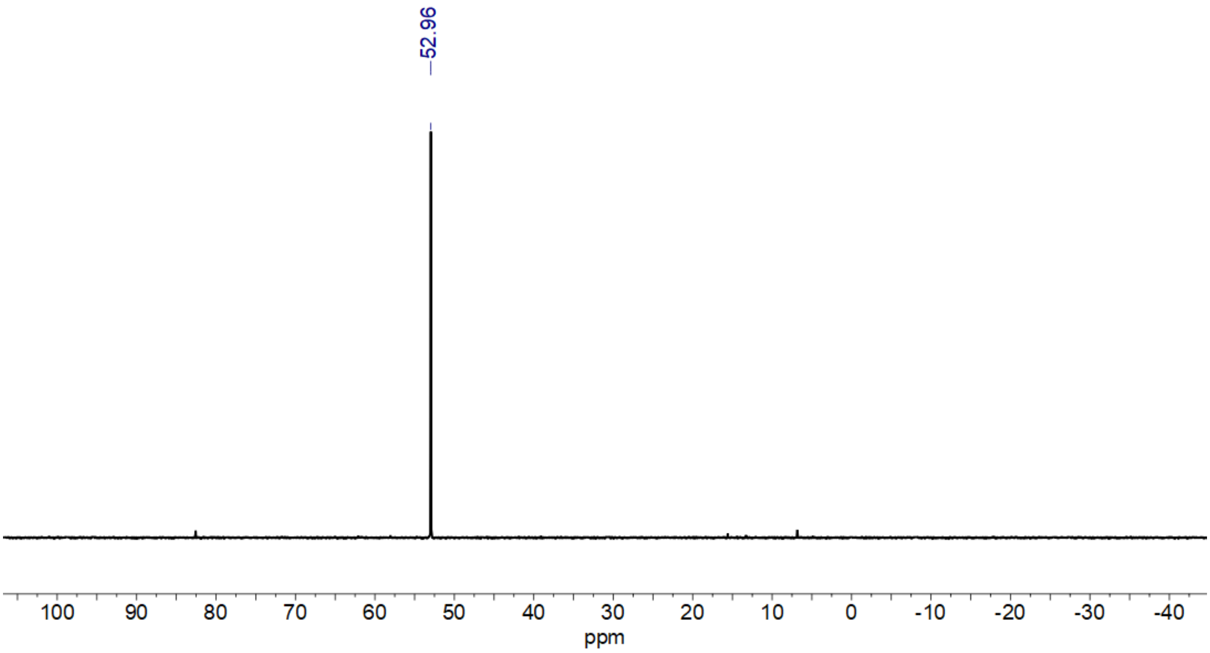

Figure S51 $^{31}P\{^{1}H\}$ NMR (203 MHz, CD_2Cl_2, 298 K) for **[2-H4][BArF4]** under a pressure of H_2 (4 bar).

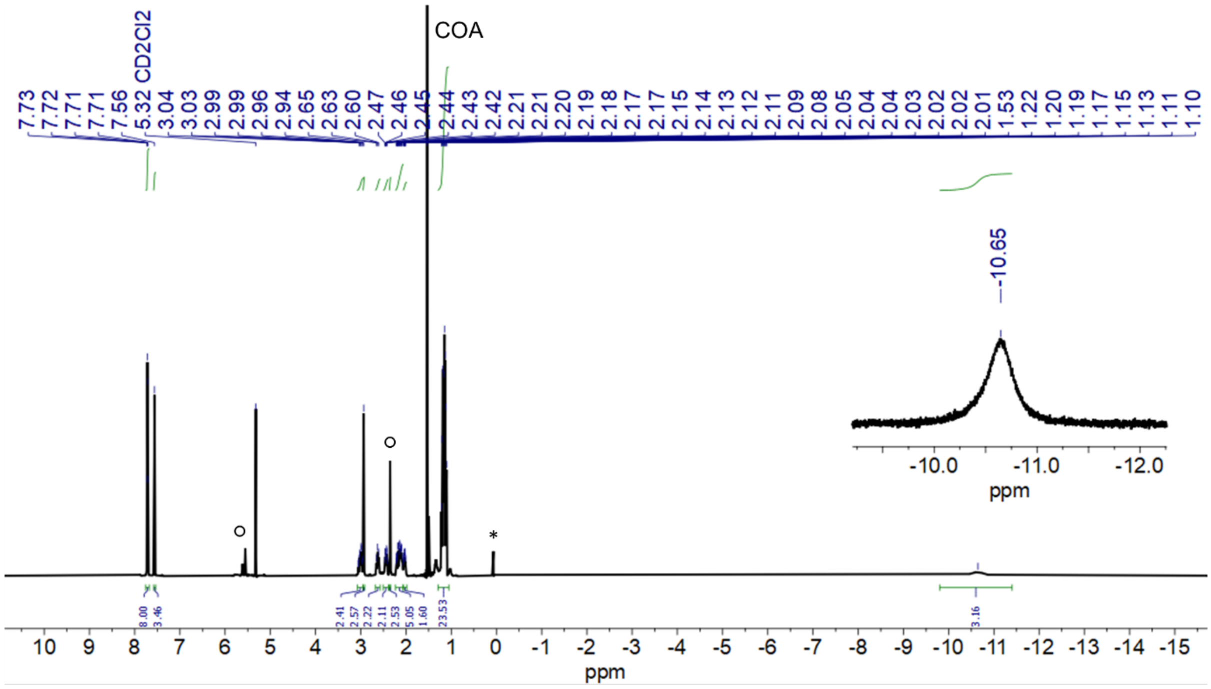

Figure S52 ^{1}H NMR (500 MHz, CD_2Cl_2, 298 K) for **[2-H$_4$][BAr^F$_4$]** under an argon atmosphere showing reduction in intensity to the Ir-H signal comparative to that of the ligand resonances. ○denotes free 1,5-COD; *denotes grease. COA = cyclooctane.

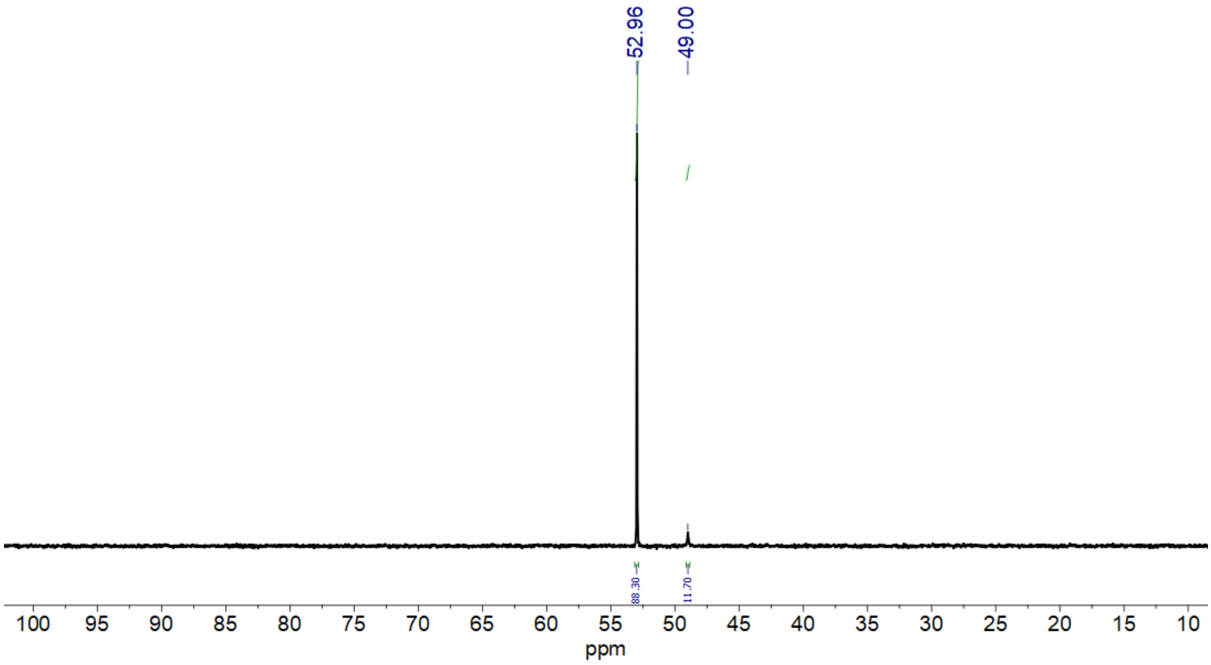

Figure S53 ^{31}P{^{1}H} NMR (203 MHz, CD_2Cl_2, 298 K) for **[2-H$_4$][BAr^F$_4$]** under an argon atmosphere showing ~12% decomposition after 10 minutes.

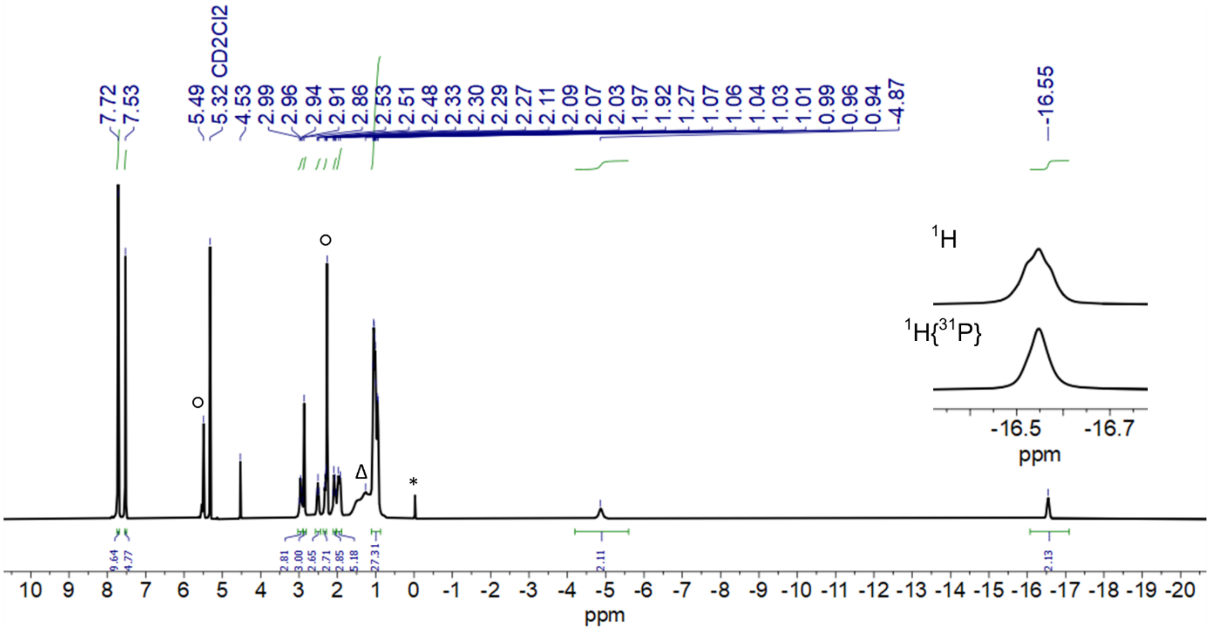

Figure S54 ^{1}H NMR (500 MHz, CD_2Cl_2, 185 K) for **[2-H$_4$][BAr$^F{}_4$]** under a pressure of H_2 (4 bar). ○denotes free 1,5-COD; *denotes grease, $^\Delta$denotes cyclooctane (m.p. 11-15 °C[S14]).

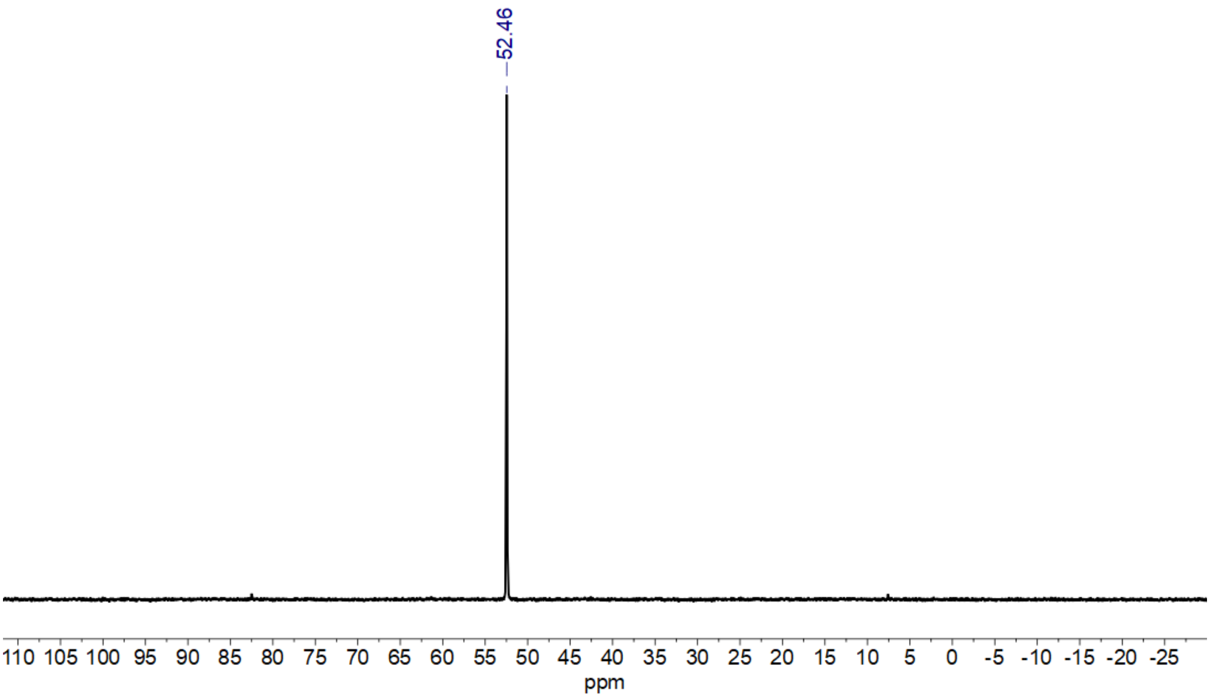

Figure S55 ^{31}P{^{1}H} NMR (203 MHz, CD_2Cl_2, 185 K) for **[2-H$_4$][BAr$^F{}_4$]** under a pressure of H_2 (4 bar).

2.15 NMR Spectra for **[2-NMeH$_2$][BAr^F$_4$]** *in situ*

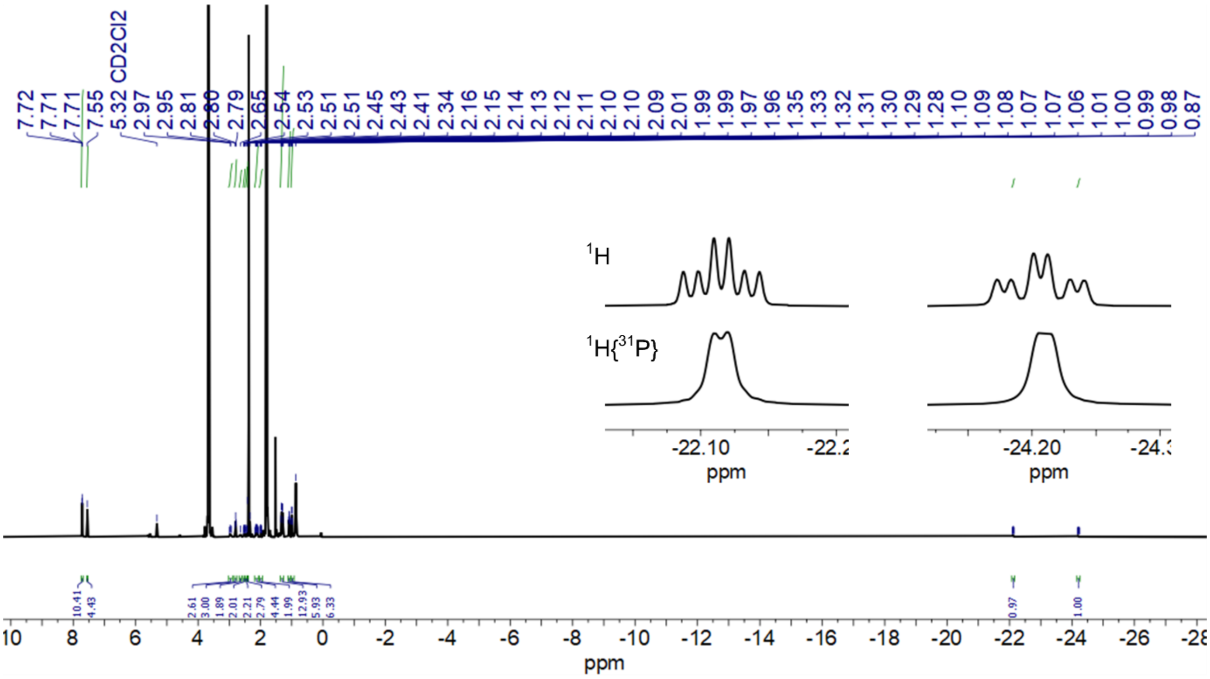

Figure S56 ^{1}H NMR (600 MHz, CD_2Cl_2) for **[2-NMeH$_2$][BAr^F$_4$]** *in situ*.

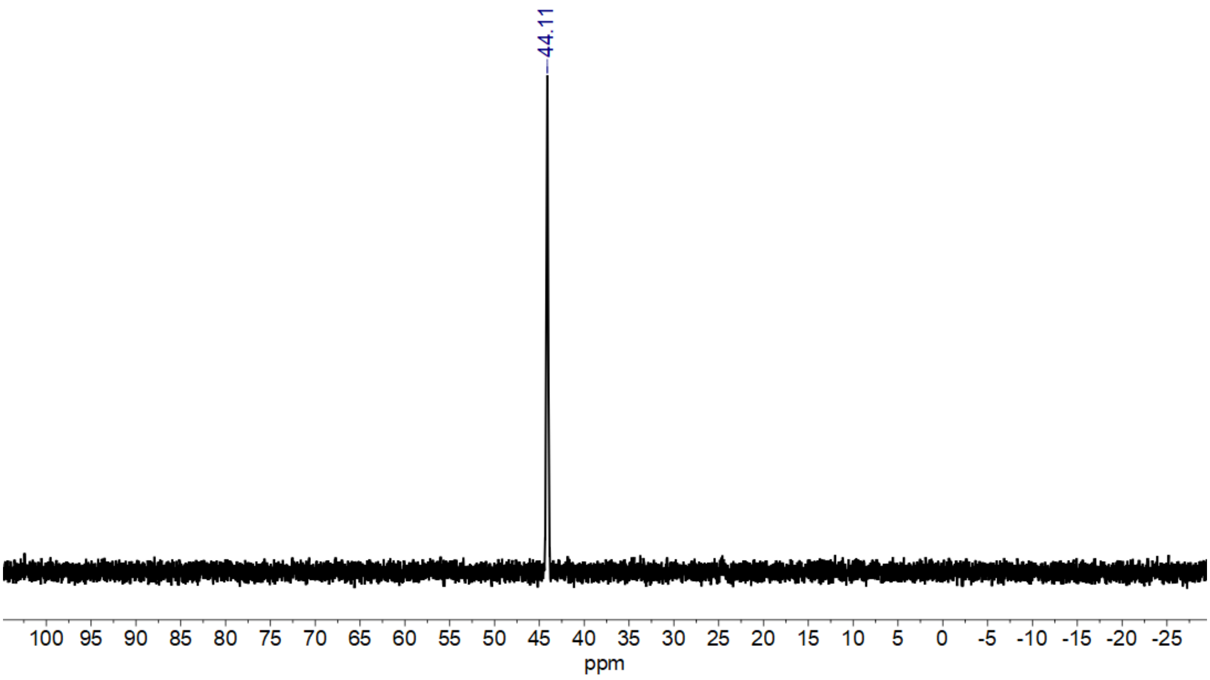

Figure S57 ^{31}P{^{1}H} NMR (203 MHz, CD_2Cl_2) for **[2-NMeH$_2$][BAr^F$_4$]** *in situ*.

2.16 NMR Spectra for $(H_2BNMeH)_n$

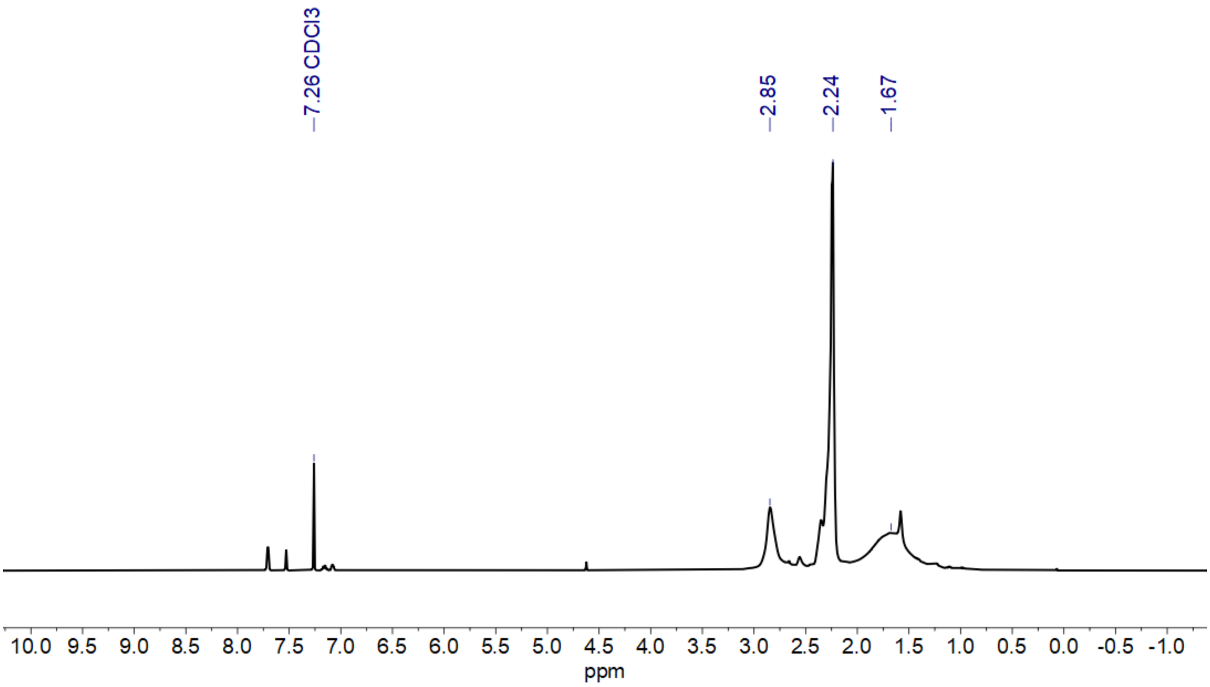

Figure S58 Typical ^{1}H NMR spectrum (500 MHz, $CDCl_3$) obtained of isolated polymer sample.

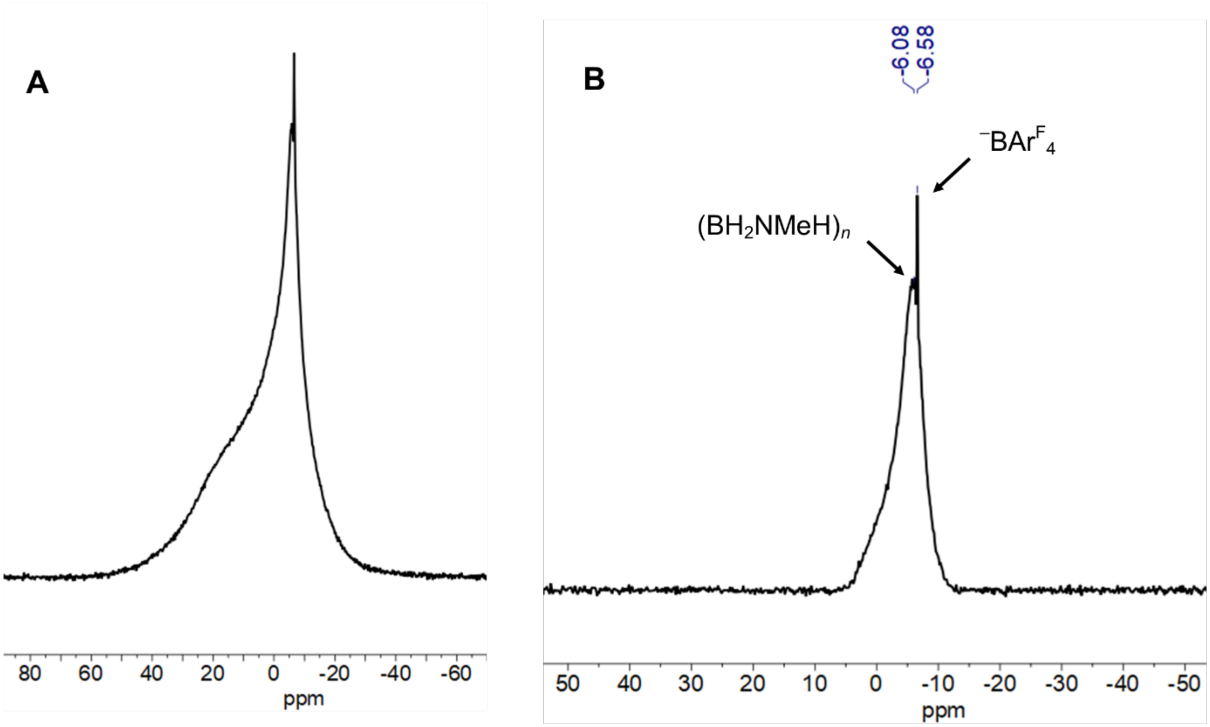

Figure S59 Typical ^{11}B NMR spectrum (161 MHz, $CDCl_3$) obtained of isolated polymer sample. **A**: Before baseline correction, **B**: After baseline correction.

4. Crystallographic Information

Single crystal X-ray diffraction data for all samples were collected as follows: a typical crystal was mounted on a MiTeGen Micromounts using perfluoropolyether oil and cooled rapidly to the collection temperature in a stream of nitrogen gas using an Oxford Cryosystems Cryostream unit.[S15] The structures were collected at the Department of chemistry, University of York on an Oxford Diffraction SuperNova diffractometer using an EOS CCD camera. Crystals with ligand **1 ([1-COD]Cl, [1-COD][BAr^F$_4$]** and **[1-H$_3$B·NMe$_3$][BAr^F$_4$])** had a tendency to contain multiple crystallographic domains, multiple crystals were selected and this habit persisted. The structures for such complexes were therefore modelled using a non-merohedral twin model.

Raw frame data were reduced using CrysAlisPro.[S16] The structures were solved using SHELXT[S17] and refined using full-matrix least squares refinement on all F2 data using the ShelXL-2018[S18] using the interface OLEX2.[S19] Most hydrogen atoms were placed at geometric position; except those on Ir, N and B which were located in the fourier difference map with the exception of the hydrogen on backbone N in **[1-H$_3$B·NMe$_3$][BAr^F$_4$]** which was located using a riding model to maintain reasonable bond length. All other hydrogen atoms were placed in calculated positions (riding model). Disorder of a -CF$_3$ group in **[1-H$_3$B·NMe$_3$][BAr^F$_4$]** and **[2-COD][BAr^F$_4$]** was treated by introducing a split site model and restraining geometries and displacement parameters. Disorder of an -iPr group in **[2-COD][BAr^F$_4$]** was treated by introducing a split site model.

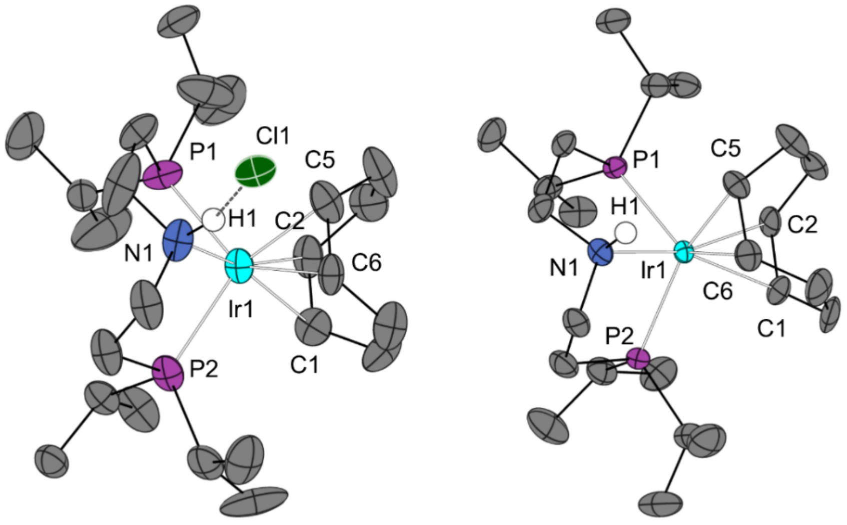

[1-COD]Cl **[1-COD][BAr^F$_4$]**

Figure S60 Molecular structures of **[1-COD]Cl** and **[1-COD][BAr^F$_4$]**. Displacement ellipsoids at 50% probability. Selected key bond angles (°) and bond lengths (Å) for **[1-COD]Cl**: P1-Ir1-P2, 105.79(7); Ir1-N1, 2.156(6); C1-C2, 1.39(1); C5-C6, 1.42(1). **[1-COD][BAr^F$_4$]**: P1-Ir1-P2, 106.54(5); Ir1-N1, 2.162(5); C1-C2, 1.403(8); C5-C6, 1.451(8). [BAr^F$_4$]$^-$ anion not shown in **[1-COD][BAr^F$_4$]**.

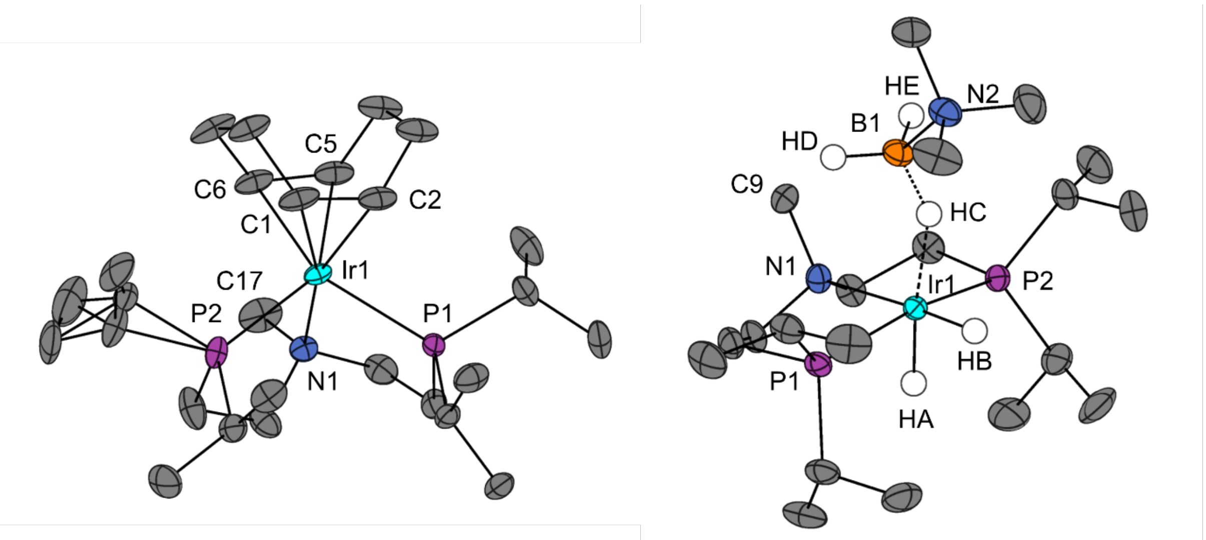

[2-COD][BAr$^F{}_4$] **[2-H$_3$B·NMe$_3$][BAr$^F{}_4$]**

Figure S61 Molecular structures of **[2-COD][BAr$^F{}_4$]** and **[2-H$_3$B·NMe$_3$][BAr$^F{}_4$]**. Displacement ellipsoids at 50% probability. Selected key bond angles (°) and bond lengths (Å) for **[2-COD][BAr$^F{}_4$]**: P1-Ir1-P2, 107.86(2); Ir1-N1, 2.231(2); C1-C2, 1.399(4); C5-C6, 1.434(4); N1-C17, 1.486(4). **[2-H$_3$B·NMe$_3$][BAr$^F{}_4$]**: B1-HC-Ir1, 139(3); HA-Ir1-HC, 171(2); P1-Ir1-P2, 162.17(3); Ir1···B1, 2.825(4); Ir1-HA, 1.40(3); Ir1-HB, 1.45(3); Ir1-HC, 1.86(4); Ir1-P1, 2.2811(9); Ir1-P2, 2.2780(8); Ir1-N1, 2.269(3); B1-N2, 1.604(5); N1-C9, 1.481(4). [BAr$^F{}_4$]$^-$ anions not shown.

Crystallographic points of note:

- In **[1-COD]Cl**, the hydrogen bond that is observed in solution between N1-H1···Cl1 is visible in the solid state, with a distance of 2.207(2)Å measured, (sum of Van der Waals radii 2.95 Å).[S20]
- In the case of **[1-COD]Cl**, **[2-COD][BAr$^F{}_4$]** and **[2-H$_3$B·NMe$_3$][BAr$^F{}_4$]** the following B-level alert is raised:
  PLAT910_ALERT_3_B Missing # of FCF Reflection(s) Below Theta(Min).
  Response: these structures were collected using Mo radiation, which causes this alert systematically on our diffractometer.
- In the case of **[2-H$_3$B·NMe$_3$][BAr$^F{}_4$]** the following B-level alert is raised: PLAT213_ALERT_2_B: Atom F5 has ADP max/min Ratio 5.0 prolat. *Response*: structures containing [BAr$^F{}_4$]$^-$ often suffer from thermal movement of the -CF$_3$ moieties. Where beneficial, this disorder is modelled (see above). Here, split site/restraints did not improve the overall data model.

	**[1-COD]Cl ·$C_6H_4F_2$·0.5C_5H_{12}**	**[1-COD][BAr^F_4]·CCl_2H_2**	**[1-H_3B·NMe_3][BAr^F_4]·0.5C_5H_{12}**
Chemical Formula	$C_{32.5}H_{59}ClF_2IrNP_2$	$C_{57}H_{63}BCl_2F_{24}IrNP_2$	$C_{53.5}H_{69}B_2F_{24}IrN_2P_2$
Formula Weight	791.39	1553.93	1471.86
Temperature /K	110	150	110
Crystal system	monoclinic	monoclinic	triclinic
Space group	C2/c	$P2_1/c$	P-1
a (Å)	25.2098(5)	12.6452(2)	14.0880(4)
b (Å)	17.8404(5)	18.7381(2)	14.6728(6)
c (Å)	15.3499(4)	25.9785(2)	17.7727(7)
α (deg)	90	90	109.222(4)
β (deg)	97.121(2)	90.0190(10)	97.898(3)
γ (deg)	90	90	112.957(3)
Volume ($Å^3$)	6850.4(3)	6155.53(13)	3041.3(2)
Z	8	4	2
ρ_{calc} g/cm^3	1.535	1.677	1.607
μ/mm^{-1}	4.103	6.496	5.747
Reflection collected	15435	11589	21730
Independent reflections	15435	11589	21730
Restraints /parameters	226/411	30/806	242/850
R_{int}	Twin Model	Twin Model	Twin Model
R_1 [I > 2σ(I)]	0.0477	0.0430	0.0475
wR_2 [all data]	0.1209	0.1111	0.1281
GooF	0.908	1.054	0.994
Residual electron density (e $Å^{-3}$)	1.68/-1.33	0.88/-1.31	3.28/-1.35
CCDC no.	2172745	2172744	2172743

	[2-COD][BAr$^F{}_4$]	[2-$H_3B\cdot NMe_3$][BAr$^F{}_4$]·($C_6H_4F_2$)
Chemical Formula	$C_{57}H_{61}BF_{24}IrNP_2$	$C_{58}H_{69}B_2F_{26}IrN_2P_2$
Formula Weight	1481.02	1563.91
Temperature /K	110.0	110
Crystal system	triclinic	triclinic
Space group	P-1	P-1
a (Å)	11.2498(2)	13.0185(3)
b (Å)	16.4343(4)	13.3058(4)
c (Å)	16.8957(4)	19.3467(4)
α (deg)	78.648(2)	80.001(2)
β (deg)	72.266(2)	80.829(2)
γ (deg)	85.259(2)	86.223(2)
Volume (Å^3)	2916.22(12)	3255.66(14)
Z	2	2
ρ_{calc} g/cm^3	1.687	1.595
μ/mm^{-1}	2.460	2.213
Reflection collected	58397	47513
Independent reflections	19028	15473
Restraints /parameters	135/860	6/852
R_{int}	0.0371	0.0367
R_1 [I > 2σ(I)]	0.0287	0.0331
wR_2 [all data]	0.0665	0.0769
GooF	1.048	1.079
Residual electron density (e Å^{-3})	1.13/-1.57	1.22/-0.80
CCDC no.	2183097	2183098

5. Computational Information

5.1 Computational Details

All geometry optimizations were run with Gaussian16 (Revision A.03)[S21] using the BP86 functional.[S22,S23] Ir and P centers were described with the Stuttgart RECPs and associated basis sets[S24] and 6-31G** basis sets were used for all other atoms.[S25,S26] A set of d-orbital polarization functions was also added to P (ζ^d=0.387).[S27] Stationary points were characterized with analytical frequency calculations. Transition states (one negative frequency) were characterized via IRC calculations and subsequent geometry optimizations to confirm the adjacent minima. Final free energies were computed using the triple-ζ basis set Def2-TZVP[S28,S29] and include corrections for dispersion using the D3BJ method[S30] and solvation with either CH_2Cl_2 or 2-hexanone as determined by the experimental conditions.[S31] 2-hexanone (ε=14.1) was employed to mimic the dielectric constant of 1,2-difluorobenzene as the latter is not available in Gaussian16. All geometries are supplied as a separate XYZ file. The NCI calculation on **[1-H$_3$B·NMe$_3$]$^+$** was based on the promolecular densities and used NCIPLOT[S32] with visualisation via VMD.[S33]

5.2 Fluxionality in **[1-H$_4$]$^+$**

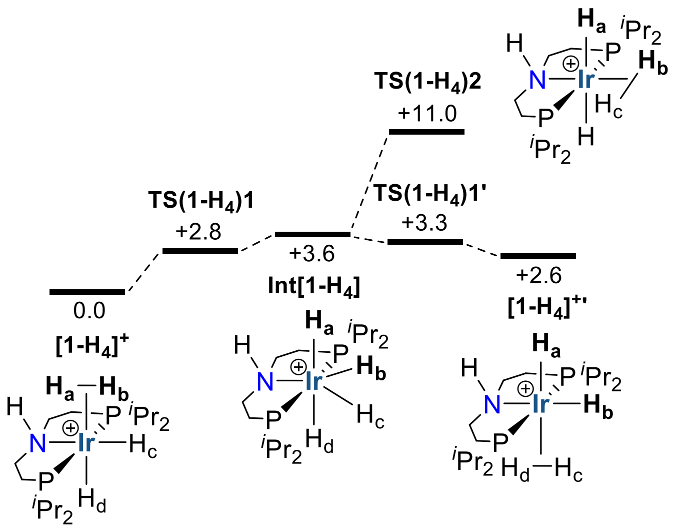

Figure S62 Full computed reaction profile (kcal/mol, in CH_2Cl_2) for H/D exchange processes in **[1-H$_4$]$^+$**. **Int[1-H$_4$]** is a genuine minimum that lies below **TS(1-H$_4$)1** and **TS(1-H$_4$)1'** on the electronic energy surface, but falls below these transition states upon inclusion of the energy corrections.

5.3 H/D exchange in **[1-H_3B·NMe_3)]$^+$**

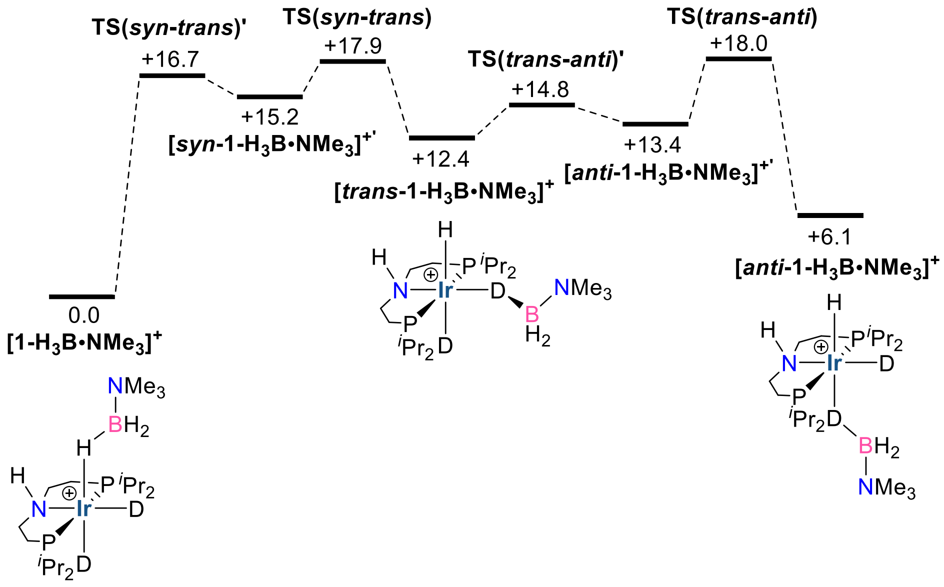

Figure S63. Full computed reaction profile (kcal/mol, in CH_2Cl_2) for H/D exchange in **[1-H_3B·NMe_3]$^+$**. **[*syn*-1-H_3B·NMe_3]$^{+\prime}$** and **[*anti*-1-H_3B·NMe_3]$^{+\prime}$** are different conformers of **[*trans*-1-H_3B·NMe_3]$^+$**. H/D exchange is completed by exchange of the bridging B–D bond and terminal B–H bonds in **[*anti*-1-H_3B·NMe_3]$^+$** and reversing the profile. A similar rearrangement in **[*trans*-1-H_3B·NMe_3]$^+$** was high in energy (+27.7 kcal/mol).

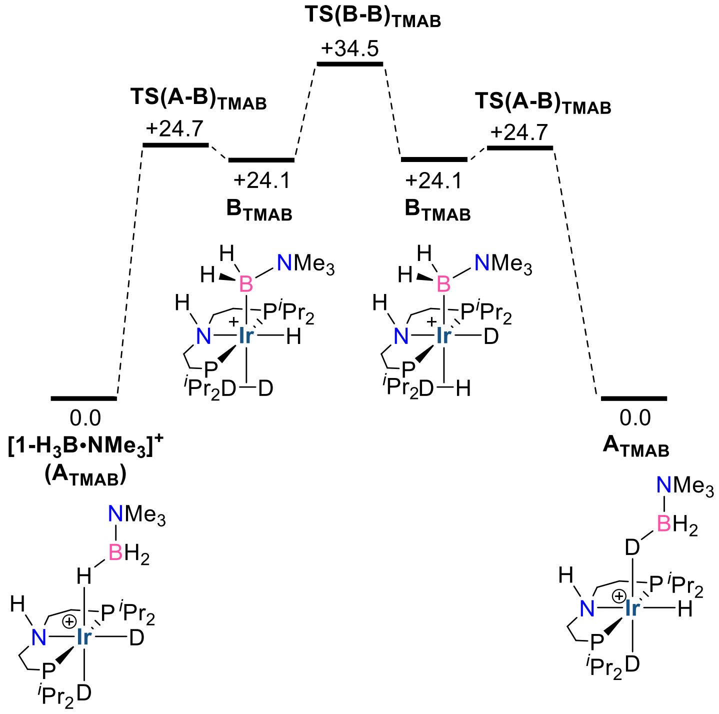

Figure S64. Computed reaction profile (kcal/mol) for the alternative pathway for H/D exchange in **[1-H_3B·NMe_3]$^+$** via initial H-transfer.

5.4 Amine-borane concerted dehydrogenation in **[1-H$_3$B·NMeH$_2$]$^+$** in the absence of outer-sphere H$_3$B·NMeH$_2$.

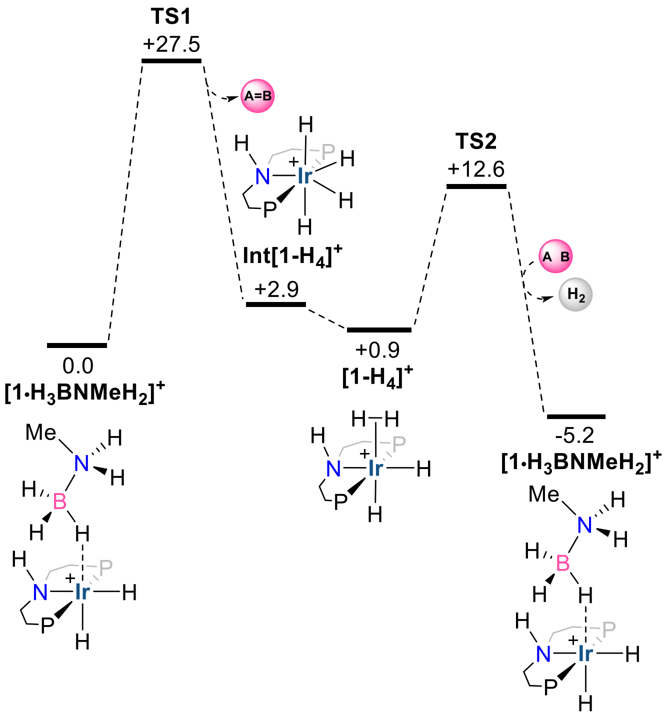

Figure S65. Computed free energy profile (kcal/mol) for the concerted H$_3$B·NMeH$_2$ dehydrogenation in **[1-H$_3$B·NMeH$_2$]$^+$** computed in the absence of an outer-sphere H$_3$B·NMeH$_2$.

5.5 Amine-borane stepwise dehydrogenation in **[1-$H_3B{\cdot}NMeH_2$]**$^+$ in the presence of an outer-sphere $H_3B{\cdot}NMeH_2$.

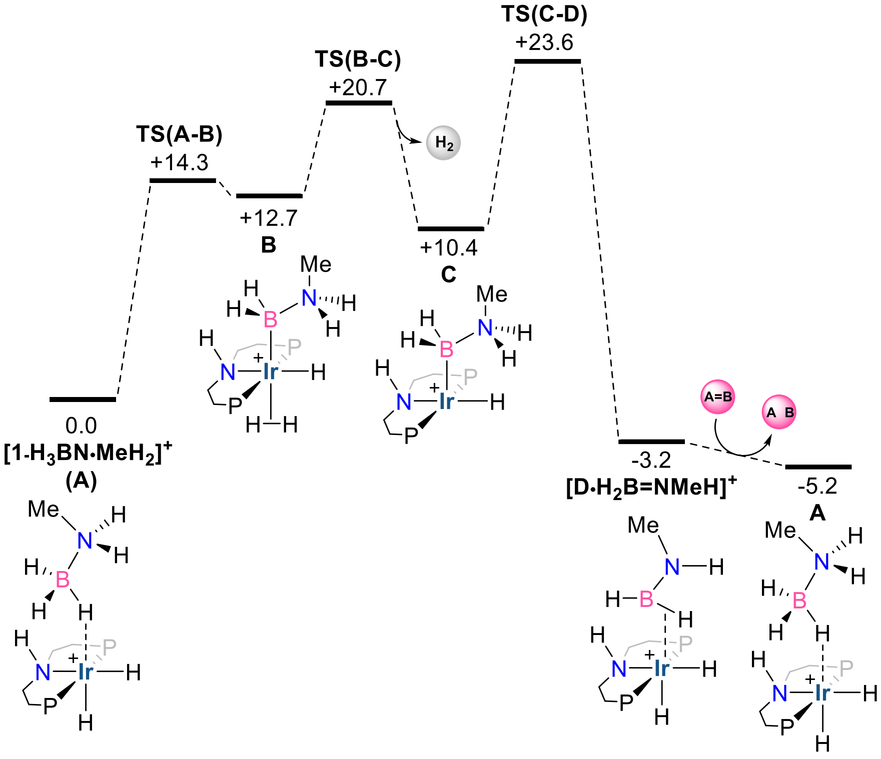

Figure S66. Computed free energy profile (kcal/mol) for the stepwise $H_3B{\cdot}NMeH_2$ dehydrogenation in **[1-$H_3B{\cdot}NMeH_2$]**$^+$ computed in the presence of an outer-sphere $H_3B{\cdot}NMeH_2$.

5.6 Amine-borane stepwise dehydrogenation in **[1-$H_3B{\cdot}NMeH_2$]$^+$** in the absence of an outer-sphere $H_3B{\cdot}NMeH_2$.

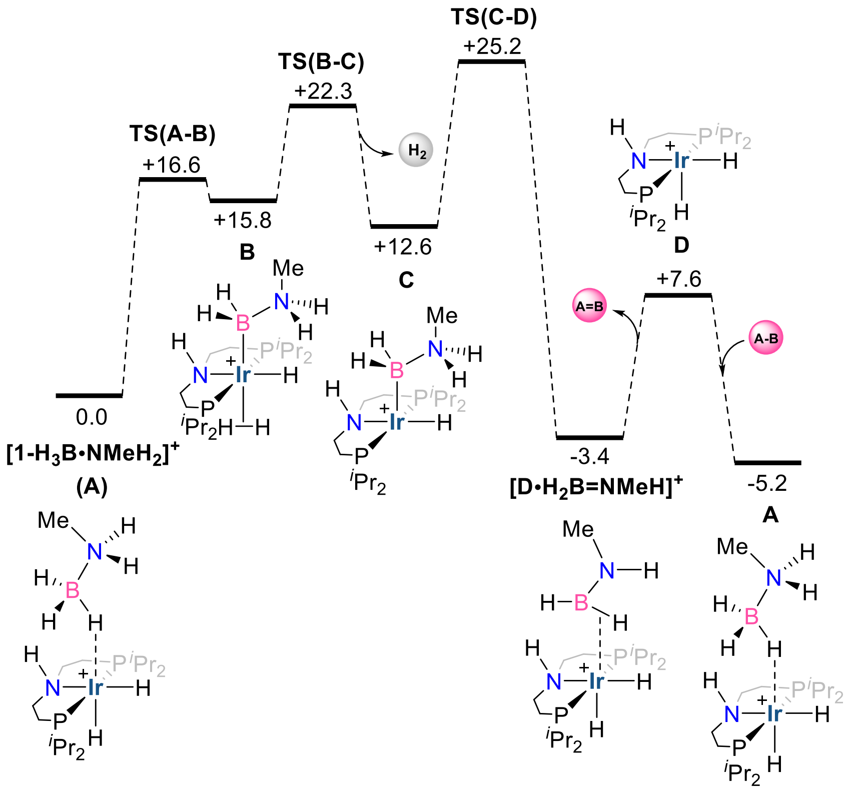

Figure S67. Computed free energy profile (kcal/mol) for the stepwise $H_3B{\cdot}NMeH_2$ dehydrogenation in **[1-$H_3B{\cdot}NMeH_2$]$^+$** computed in the absence of an outer-sphere $H_3B{\cdot}NMeH_2$.

5.7 Competing deprotonation vs. H_2/$NMeH_2$ substitution in **[1-H$_4$]$^+$**

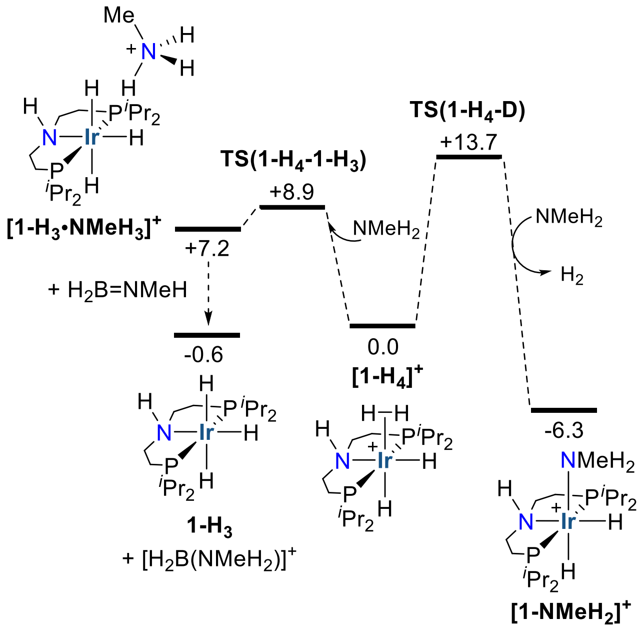

Figure S68. Computed free energy profile (kcal/mol) for the $NMeH_2$-assisted deprotonation of **[1-H$_4$]$^+$** (left) and loss of hydrogen and $NMeH_2$ coordination to the Ir center (right). All species are computed with an outer-sphere $H_3B{\cdot}NMeH_2$ molecule included in the model, but these are omitted in the schematic drawings for clarity.

5.8 H_3B•$NMeH_2$ dehydrogenation at **1-H₃**

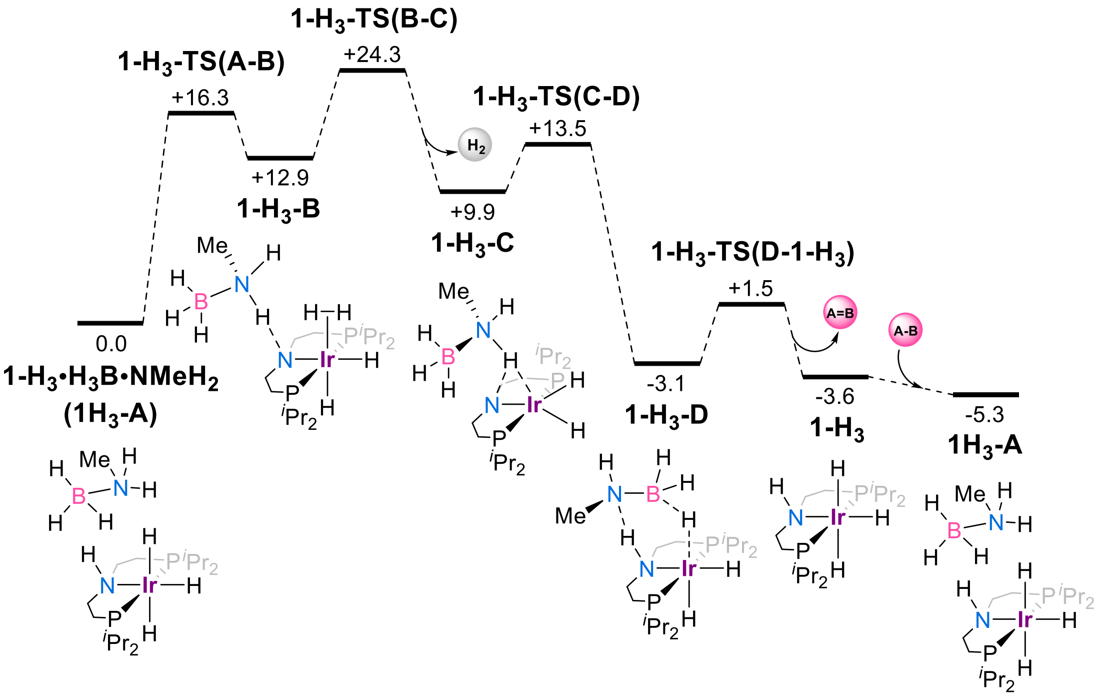

Figure S69. Computed free energy profile (kcal/mol) for dehydrogenation of $H_3B{\cdot}NMeH_2$ at **1-H$_3$**.

5.9 Non-covalent Interaction (NCI) plot for **[1-H_3B·$NMeH_2$]$^+$**

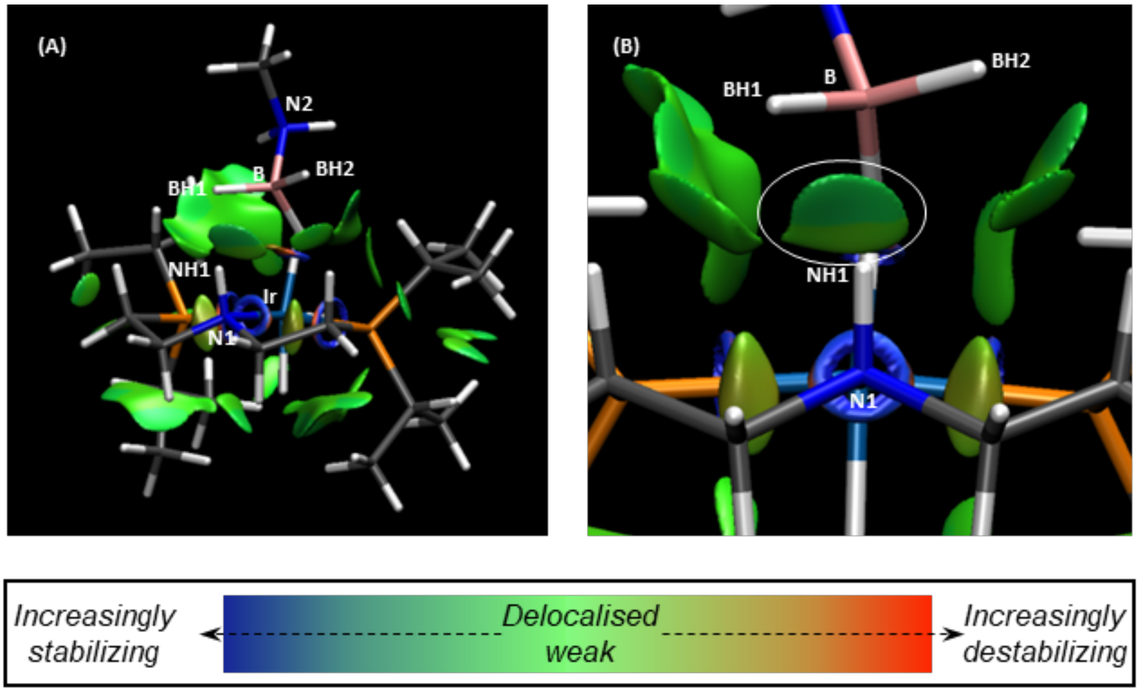

Figure S70. Two views of the NCI plots for **[1-H_3B·$NMeH_2$]$^+$**. (A) General view of the **[1-H_3B·$NMeH_2$]$^+$** cation; (B) Detail looking down the Ir-N bond of the ^{i}Pr-PN^{H}P ligand with the region of stabilisation between the N-H proton of the ligand and the amine-borane B-H bonds highlighted. Isosurfaces are generated for s = 0.3 au and $-0.07 < \rho < 0.07$ au. and a key showing the color scheme employed is also provided. Atom coloring scheme: Ir: blue; H: white; B: pink; C: gray; N: azure; P: orange.

5.10 Computed Structures (Å) and Energies (atomic units)

H_2

```
BP86 energy = -1.17646508218
Enthalpy 0K = -1.166532
Enthalpy 298K = -1.163228
Free energy 298K = -1.178038
Low freq. = 4360.0292 cm-1

H   0.00000  0.00000  0.37512
H   0.00000  0.00000 -0.37512
```

$H_3B{\cdot}NMe_3$

```
BP86 energy = -201.129590304
Enthalpy 0K = -200.978721
Enthalpy 298K = -200.970719
Free energy 298K = -201.007890
Low freq. = 189.9292 cm-1
Second freq. = 278.0601 cm-1

N   0.00508 -0.00001  0.00001
C  -0.49296  1.37013  0.32474
C  -0.49615 -0.40396 -1.34765
C  -0.49331 -0.96538  1.02479
B   1.66335 -0.00086 -0.00211
H   1.98491  0.80770 -0.86062
H   1.98729  0.33769  1.12691
H   1.98464 -1.14853 -0.27391
H  -0.10221  2.06899 -0.42831
H  -0.10033  1.65763  1.31030
H  -1.59788  1.39483  0.33177
H  -1.59825 -0.98177  1.04422
H  -0.10061 -0.66350  2.00602
H  -0.10291 -1.96340  0.78037
H  -1.60113 -0.41111 -1.36963
H  -0.10519 -1.40518 -1.57761
H  -0.10611  0.30629 -2.09033
```

$H_3B{\cdot}NMeH_2$

```
BP86 energy = -122.516499919
Enthalpy 0K = -122.420154
Enthalpy 298K = -122.414305
Free energy 298K = -122.446179
Low freq. = 186.6510 cm-1
Second freq. = 244.5598 cm-1

N   0.00490  0.55312 -0.00000
C   1.23647 -0.28560 -0.00000
H   0.00713  1.17101 -0.82159
H   0.00713  1.17100  0.82160
B  -1.40308 -0.30947 -0.00000
H  -1.36589 -0.97735 -1.02228
H  -1.36583 -0.97751  1.02216
H  -2.28681  0.53341  0.00010
H   1.20568 -0.92869 -0.89077
H   1.20618 -0.92801  0.89127
H   2.15467  0.32530 -0.00048
```

$BH_2{=}NMeH$

```
BP86 energy = -121.338180687
Enthalpy 0K = -121.263433
Enthalpy 298K = -121.258144
Free energy 298K = -121.288670
Low freq. = 188.8576 cm-1
Second freq. = 387.0399 cm-1

H   1.76920 -0.12687 -0.89317
H  -0.13924 -1.44081  0.00005
B  -1.37054  0.25365 -0.00017
C   1.18965  0.17193  0.00019
N  -0.14563 -0.42002 -0.00008
H  -1.36942  1.46061 -0.00030
H   1.76817 -0.12526  0.89475
H   1.09217  1.26796 -0.00082
H  -2.38671 -0.39532 -0.00029
```

$NMeH_2$

```
BP86 energy = -95.8564889607
Enthalpy 0K = -95.794042
Enthalpy 298K = -95.789678
Free energy 298K = -95.816970
Low freq. = 323.3364 cm-1
Second freq. = 836.1742 cm-1

N   0.75714 -0.00000 -0.12876
C  -0.70724 -0.00000  0.01804
H   1.14376 -0.81567  0.35883
H   1.14376  0.81567  0.35883
H  -1.12183 -0.88647 -0.49218
H  -1.12183  0.88647 -0.49218
H  -1.10043  0.00000  1.05971
```

$NMeH_3$

```
BP86 energy = -96.2194971491
Enthalpy 0K = -96.142014
Enthalpy 298K = -96.137586
Free energy 298K = -96.165233
Low freq. = 291.7746 cm-1
Second freq. = 892.1186 cm-1
```

```
N   0.71475 -0.00000  0.00000
C  -0.80574  0.00000  0.00000
H   1.09424 -0.08065 -0.95890
H   1.09424 -0.79013  0.54927
H  -1.15051  0.85552 -0.59512
H  -1.15052  0.08763  1.03846
H  -1.15053 -0.94314 -0.44334
H   1.09429  0.87077  0.40960
```

$[H_2B(NMeH_2)_2]^+$

```
BP86 energy = -217.611260532
Enthalpy 0K = -217.453558
Enthalpy 298K = -217.445032
Free energy 298K = -217.484599
Low freq. = 91.1540 cm-1
Second freq. = 98.3088 cm-1

N   1.32828 -0.51597 -0.00044
C   2.56719  0.35334  0.00017
H   1.37921 -1.13417  0.82372
H   1.37894 -1.13271 -0.82571
B   0.00021  0.40630  0.00015
H   0.00038  1.04402  1.02536
H   0.00015  1.04446 -1.02476
H   2.54328  0.98608  0.89704
H   2.54263  0.98828 -0.89513
H   3.46801 -0.27627 -0.00095
H  -2.54261  0.98893  0.89448
H  -1.37913 -1.13251  0.82569
C  -2.56744  0.35328 -0.00031
N  -1.32817 -0.51571  0.00044
H  -3.46794 -0.27677  0.00169
H  -1.37932 -1.13409 -0.82363
H  -2.54398  0.98526 -0.89772
```

$[1\text{-}H_4]^+$

```
BP86 energy = -806.712550247
Enthalpy 0K = -806.178848
Enthalpy 298K = -806.147918
Free energy 298K = -806.238068
Low freq. = 24.3860 cm-1
Second freq. = 29.1709 cm-1

Ir -0.00000  0.27384 -0.02998
H   0.00004 -0.98048  0.97808
H  -0.00003  1.10606  1.33171
H   0.00009  1.94179 -0.47322
P  -2.33621  0.01303 -0.10419
P   2.33620  0.01309 -0.10415
N   0.00003 -1.28281 -1.67652
H   0.00003 -0.78248 -2.57536
C  -2.50239 -1.24345 -1.51820
H  -3.39571 -1.87901 -1.39680
H  -2.64612 -0.65997 -2.44648
C  -1.24604 -2.11672 -1.62233
H  -1.15206 -2.76250 -0.73417
H  -1.30000 -2.77126 -2.51410
C   1.24612 -2.11667 -1.62231
H   1.30011 -2.77122 -2.51409
H   1.15216 -2.76245 -0.73415
C   2.50243 -1.24334 -1.51819
H   2.64607 -0.65979 -2.44644
H   3.39580 -1.87883 -1.39686
C  -3.22954 -0.74654  1.38389
H  -4.30434 -0.70286  1.12425
C  -2.83379 -2.21792  1.60263
H  -3.36963 -2.61180  2.48339
H  -1.75282 -2.31539  1.80404
H  -3.09543 -2.86512  0.74908
C  -2.97130  0.08607  2.65415
H  -3.51949 -0.35781  3.50305
H  -3.30226  1.13134  2.55544
H  -1.89766  0.09225  2.90632
C  -3.38901  1.46097 -0.73798
H  -2.89679  1.66777 -1.71012
C  -4.86041  1.08685 -0.99755
H  -5.37146  1.92521 -1.50171
H  -5.40333  0.89860 -0.05594
H  -4.97158  0.19951 -1.64269
C  -3.26241  2.72268  0.13633
H  -3.69465  3.58637 -0.39749
H  -2.21401  2.96191  0.37857
H  -3.81633  2.61748  1.08364
C   3.38907  1.46102 -0.73784
H   2.89671  1.66810 -1.70984
C   3.26279  2.72256  0.13678
H   3.69498  3.58632 -0.39694
H   3.81693  2.61708  1.08393
H   2.21446  2.96182  0.37932
C   4.86038  1.08679 -0.99776
H   5.37145  1.92522 -1.50178
H   4.97133  0.19961 -1.64317
H   5.40345  0.89820 -0.05630
C   3.22942 -0.74647  1.38401
H   4.30427 -0.70250  1.12461
C   2.83398 -2.21796  1.60250
H   3.36974 -2.61183  2.48331
H   3.09591 -2.86501  0.74892
H   1.75298 -2.31571  1.80372
C   2.97073  0.08592  2.65433
H   3.51897 -0.35787  3.50324
H   1.89706  0.09168  2.90636
H   3.30131  1.13132  2.55578
H  -0.00027  1.52540 -1.28458
```

TS(1-H$_4$)1

BP86 energy = -806.710023668
Enthalpy 0K = -806.178283
Enthalpy 298K = -806.147665
Free energy 298K = -806.237226
Low freq. = -527.8891 cm-1
Second freq. = 23.0157 cm-1

```
Ir -0.00000  0.24435 -0.00075
H   0.00000 -1.10197  0.95089
H  -0.00000  0.83927  1.47951
H  -0.00000  1.40894 -1.20361
P  -2.34345  0.02191 -0.10847
P   2.34345  0.02191 -0.10847
N   0.00000 -1.26367 -1.65894
H  -0.00000 -0.71994 -2.53217
C  -2.49971 -1.21317 -1.54198
H  -3.39990 -1.84205 -1.44079
H  -2.62488 -0.61572 -2.46387
C  -1.24943 -2.09487 -1.64033
H  -1.17162 -2.75788 -0.76355
H  -1.28804 -2.72783 -2.54800
C   1.24943 -2.09487 -1.64033
H   1.28804 -2.72783 -2.54800
H   1.17162 -2.75788 -0.76355
C   2.49971 -1.21316 -1.54198
H   2.62488 -0.61572 -2.46387
H   3.39990 -1.84205 -1.44079
C  -3.27834 -0.74351  1.35110
H  -4.34664 -0.67100  1.07153
C  -2.91801 -2.22628  1.55244
H  -3.47712 -2.62207  2.41771
H  -1.84278 -2.34950  1.76919
H  -3.17961 -2.85407  0.68444
C  -3.02820  0.06176  2.64047
H  -3.61224 -0.37749  3.46751
H  -3.32314  1.11857  2.55039
H  -1.96241  0.02792  2.92249
C  -3.35418  1.49679 -0.74292
H  -2.83265  1.70915 -1.69793
C  -4.82514  1.15102 -1.04169
H  -5.30873  2.00396 -1.54836
H  -5.39445  0.96220 -0.11591
H  -4.93654  0.27318 -1.69974
C  -3.22728  2.74293  0.15272
H  -3.64115  3.61886 -0.37558
H  -2.17954  2.96751  0.41051
H  -3.79702  2.63107  1.08987
C   3.35418  1.49679 -0.74292
H   2.83265  1.70916 -1.69793
C   3.22728  2.74293  0.15272
H   3.64115  3.61886 -0.37558
H   3.79702  2.63107  1.08987
H   2.17954  2.96751  0.41051
C   4.82514  1.15102 -1.04169
H   5.30873  2.00396 -1.54836
H   4.93654  0.27319 -1.69974
H   5.39445  0.96220 -0.11591
C   3.27834 -0.74351  1.35110
H   4.34664 -0.67100  1.07153
C   2.91801 -2.22628  1.55244
H   3.47712 -2.62207  2.41771
H   3.17962 -2.85407  0.68444
H   1.84278 -2.34950  1.76919
C   3.02820  0.06176  2.64047
H   3.61224 -0.37749  3.46751
H   1.96241  0.02792  2.92249
H   3.32314  1.11857  2.55039
H   0.00000  1.86144  0.01262
```

Int[1-H$_4$]

BP86 energy = -806.711520046
Enthalpy 0K = -806.178029
Enthalpy 298K = -806.147326
Free energy 298K = -806.236498
Low freq. = 28.5405 cm-1
Second freq. = 36.4830 cm-1

```
Ir  0.00001  0.18566  0.05059
H  -0.00020 -1.22944  0.91738
H   0.00023  0.41723  1.62852
H   0.00015  1.34431 -1.14063
P  -2.34633  0.02999 -0.11838
P   2.34630  0.02989 -0.11849
N  -0.00005 -1.22992 -1.65827
H  -0.00003 -0.62880 -2.49365
C  -2.49812 -1.16965 -1.58383
H  -3.40229 -1.79538 -1.50109
H  -2.61451 -0.55157 -2.49293
C  -1.25272 -2.05668 -1.69471
H  -1.19147 -2.75537 -0.84481
H  -1.27606 -2.64901 -2.62976
C   1.25254 -2.05680 -1.69471
H   1.27581 -2.64914 -2.62975
H   1.19121 -2.75546 -0.84479
C   2.49804 -1.16989 -1.58384
H   2.61462 -0.55198 -2.49301
H   3.40212 -1.79574 -1.50089
C  -3.34910 -0.75251  1.28548
H  -4.40006 -0.69567  0.94337
C  -2.97731 -2.23042  1.50135
H  -3.58415 -2.64132  2.32649
H  -1.91601 -2.33949  1.78333
H  -3.17338 -2.85604  0.61457
C  -3.19095  0.05245  2.58918
H  -3.80122 -0.40985  3.38404
```

```
H  -3.51670  1.09928  2.48836
H  -2.14127  0.05155  2.92893
C  -3.29597  1.54740 -0.74581
H  -2.78031  1.72611 -1.71054
C  -4.78629  1.26666 -1.01489
H  -5.23610  2.13028 -1.53428
H  -5.34911  1.12650 -0.07660
H  -4.95030  0.38039 -1.65070
C  -3.09095  2.79544  0.13162
H  -3.49480  3.67968 -0.39071
H  -2.02538  2.98304  0.33908
H  -3.62433  2.71524  1.09297
C   3.29594  1.54726 -0.74607
H   2.78062  1.72557 -1.71105
C   3.09039  2.79556  0.13084
H   3.49445  3.67966 -0.39156
H   3.62325  2.71573  1.09251
H   2.02469  2.98313  0.33768
C   4.78639  1.26661 -1.01452
H   5.23627  2.13008 -1.53412
H   4.95073  0.38010 -1.64990
H   5.34890  1.12692 -0.07597
C   3.34925 -0.75263  1.28525
H   4.40009 -0.69641  0.94267
C   2.97682 -2.23028  1.50170
H   3.58377 -2.64123  2.32675
H   3.17229 -2.85628  0.61505
H   1.91557 -2.33877  1.78407
C   3.19205  0.05281  2.58876
H   3.80211 -0.40977  3.38362
H   2.14245  0.05290  2.92877
H   3.51868  1.09933  2.48757
H  -0.00012  1.68290  0.60188
```

TS(1-H$_4$)1'

```
BP86 energy = -806.709694164
Enthalpy 0K = -806.177658
Enthalpy 298K = -806.147120
Free energy 298K = -806.236326
Low freq. = -536.6142 cm-1
Second freq. = 26.0119 cm-1
```

```
Ir -0.00000  0.13476  0.10372
H  -0.00001 -1.27836  1.00083
H  -0.00001 -0.19991  1.68709
H   0.00001  1.34328 -1.01896
P  -2.34269  0.03732 -0.13082
P   2.34269  0.03732 -0.13079
N   0.00001 -1.23411 -1.65955
H   0.00001 -0.59612 -2.46783
C  -2.49349 -1.14792 -1.61138
H  -3.40562 -1.76356 -1.54042
H  -2.59821 -0.52005 -2.51517
C  -1.25652 -2.04857 -1.72865
H  -1.21207 -2.76893 -0.89542
H  -1.28088 -2.61980 -2.67721
C   1.25654 -2.04857 -1.72863
H   1.28092 -2.61979 -2.67720
H   1.21209 -2.76893 -0.89541
C   2.49351 -1.14792 -1.61135
H   2.59824 -0.52005 -2.51514
H   3.40564 -1.76356 -1.54039
C  -3.40977 -0.73695  1.23015
H  -4.44249 -0.68997  0.83498
C  -3.04366 -2.21189  1.47541
H  -3.70025 -2.62454  2.26060
H  -2.00313 -2.31779  1.82760
H  -3.17632 -2.84174  0.57998
C  -3.32419  0.08075  2.53267
H  -3.95507 -0.38963  3.30647
H  -3.67184  1.11783  2.40782
H  -2.29027  0.11267  2.91711
C  -3.23137  1.58811 -0.76441
H  -2.71011  1.74148 -1.73051
C  -4.73159  1.36554 -1.03142
H  -5.14701  2.24367 -1.55507
H  -5.29873  1.25285 -0.09203
H  -4.93114  0.48310 -1.66274
C  -2.97075  2.83003  0.10689
H  -3.34280  3.72743 -0.41670
H  -1.89671  2.97115  0.30567
H  -3.49932  2.77515  1.07264
C   3.23138  1.58812 -0.76437
H   2.71013  1.74149 -1.73048
C   2.97075  2.83003  0.10693
H   3.34280  3.72743 -0.41665
H   3.49931  2.77514  1.07269
H   1.89671  2.97115  0.30571
C   4.73160  1.36554 -1.03137
H   5.14702  2.24368 -1.55501
H   4.93116  0.48311 -1.66269
H   5.29873  1.25285 -0.09197
C   3.40976 -0.73695  1.23019
H   4.44248 -0.68996  0.83503
C   3.04365 -2.21189  1.47544
H   3.70023 -2.62455  2.26064
H   3.17632 -2.84173  0.58001
H   2.00311 -2.31779  1.82761
C   3.32415  0.08074  2.53271
H   3.95503 -0.38963  3.30651
H   2.29023  0.11266  2.91714
H   3.67179  1.11783  2.40787
H  -0.00001  1.46615  0.98508
```

[1-H$_4$]$^{+\prime}$

```
BP86 energy = -806.711761780
```

```
Enthalpy 0K = -806.177860
Enthalpy 298K = -806.147024
Free energy 298K = -806.236720
Low freq. = 24.3939 cm-1
Second freq. = 35.1325 cm-1

Ir  0.00000  0.10942  0.13615
H   0.00022 -1.35163  1.12416
H   0.00016  1.30721  1.19364
H   0.00012  1.33503 -0.91273
P  -2.33664  0.04022 -0.13707
P   2.33664  0.04022 -0.13705
N  -0.00000 -1.24724 -1.65578
H   0.00001 -0.59278 -2.45112
C  -2.48842 -1.13823 -1.62571
H  -3.40819 -1.74356 -1.56476
H  -2.58000 -0.50560 -2.52774
C  -1.25916 -2.05188 -1.74205
H  -1.22932 -2.78189 -0.91589
H  -1.28640 -2.61629 -2.69507
C   1.25915 -2.05189 -1.74204
H   1.28639 -2.61630 -2.69506
H   1.22930 -2.78190 -0.91587
C   2.48843 -1.13825 -1.62568
H   2.58004 -0.50563 -2.52772
H   3.40818 -1.74360 -1.56470
C  -3.45208 -0.72305  1.19339
H  -4.47091 -0.67232  0.76399
C  -3.10603 -2.20016  1.45447
H  -3.80112 -2.61021  2.20718
H  -2.08543 -2.31523  1.85856
H  -3.19788 -2.82927  0.55350
C  -3.40534  0.09914  2.49491
H  -4.05078 -0.37343  3.25535
H  -3.75979  1.13217  2.35643
H  -2.38142  0.14484  2.90395
C  -3.18388  1.61222 -0.77578
H  -2.65317  1.75449 -1.73855
C  -4.68684  1.42800 -1.05490
H  -5.07692  2.31811 -1.57782
H  -5.26362  1.32518 -0.12024
H  -4.90280  0.55314 -1.69149
C  -2.89608  2.84467  0.10081
H  -3.23666  3.75378 -0.42407
H  -1.82058  2.95159  0.31262
H  -3.43527  2.80262  1.06126
C   3.18381  1.61224 -0.77578
H   2.65308  1.75451 -1.73854
C   2.89606  2.84469  0.10082
H   3.23670  3.75378 -0.42404
H   3.43522  2.80259  1.06128
H   1.82056  2.95166  0.31260
C   4.68677  1.42801 -1.05495
H   5.07685  2.31813 -1.57784
H   4.90269  0.55317 -1.69157
H   5.26356  1.32513 -0.12030
C   3.45213 -0.72302  1.19337
H   4.47094 -0.67231  0.76395
C   3.10608 -2.20014  1.45444
H   3.80120 -2.61022  2.20711
H   3.19788 -2.82923  0.55344
H   2.08550 -2.31520  1.85858
C   3.40541  0.09919  2.49488
H   4.05078 -0.37343  3.25535
H   2.38147  0.14498  2.90387
H   3.75996  1.13218  2.35639
H  -0.00082 -0.62476  1.68931
```

TS(1-H$_4$)2

```
BP86 energy = -806.694532736
Enthalpy 0K = -806.162004
Enthalpy 298K = -806.131719
Free energy 298K = -806.220579
Low freq. = -468.2754 cm-1
Second freq. = 27.3857 cm-1

Ir  0.00001  0.17989  0.06173
H   0.00000 -1.12088  1.12068
H   0.49686  1.17357  1.28663
H   0.00002  1.49425 -0.98666
P  -2.34673  0.01848 -0.14023
P   2.34673  0.01841 -0.14028
N  -0.00002 -1.16227 -1.63317
H   0.00004 -0.53711 -2.45262
C  -2.49530 -1.14564 -1.62047
H  -3.39449 -1.77881 -1.54649
H  -2.60782 -0.51765 -2.52265
C  -1.23914 -2.01120 -1.71197
H  -1.18466 -2.71794 -0.86867
H  -1.22014 -2.58745 -2.65671
C   1.23904 -2.01131 -1.71189
H   1.22002 -2.58761 -2.65660
H   1.18448 -2.71798 -0.86855
C   2.49526 -1.14583 -1.62043
H   2.60786 -0.51793 -2.52265
H   3.39441 -1.77906 -1.54636
C  -3.34267 -0.77973  1.25977
H  -4.39789 -0.68642  0.93977
C  -2.99781 -2.26980  1.42955
H  -3.58501 -2.68575  2.26629
H  -1.92876 -2.40174  1.66952
H  -3.23988 -2.87018  0.53659
C  -3.13708 -0.01466  2.58065
H  -3.75438 -0.47333  3.37211
H  -3.42458  1.04647  2.51252
H  -2.08355 -0.07212  2.90451
```

```
C  -3.28009  1.56036 -0.72831
H  -2.77928  1.74074 -1.70003
C  -4.78029  1.30589 -0.96737
H  -5.22473  2.17829 -1.47648
H  -5.32714  1.17322 -0.01861
H  -4.97225  0.42407 -1.60190
C  -3.03117  2.79416  0.15689
H  -3.45603  3.68680 -0.33348
H  -1.95454  2.97588  0.30640
H  -3.51824  2.70356  1.14191
C   3.28011  1.56024 -0.72848
H   2.77948  1.74041 -1.70033
C   3.03095  2.79420  0.15644
H   3.45592  3.68676 -0.33399
H   3.51776  2.70379  1.14160
H   1.95428  2.97591  0.30564
C   4.78037  1.30581 -0.96721
H   5.22485  2.17813 -1.47644
H   4.97251  0.42386 -1.60150
H   5.32705  1.17340 -0.01831
C   3.34270 -0.77973  1.25973
H   4.39789 -0.68669  0.93956
C   2.99757 -2.26971  1.42981
H   3.58481 -2.68563  2.26654
H   3.23941 -2.87027  0.53691
H   1.92854 -2.40140  1.66993
C   3.13746 -0.01439  2.58050
H   3.75471 -0.47309  3.37198
H   2.08394 -0.07151  2.90449
H   3.42523  1.04665  2.51216
H  -0.49684  1.17374  1.28649
```

[1-$H_3B{\cdot}NMe_3$]$^+$

```
BP86 energy = -1006.67882552
Enthalpy 0K = -1006.007669
Enthalpy 298K = -1005.969349
Free energy 298K = -1006.074927
Low freq. = 22.6033 cm-1
Second freq. = 36.9828 cm-1

Ir  0.00015 -0.27918 -0.16684
H   0.00050 -1.83546 -0.46701
H   0.00004 -0.13771 -1.75736
P   2.31209 -0.54316  0.03919
P  -2.31164 -0.54424  0.03919
N   0.00026 -0.56057  2.07964
H  -0.00008  0.40215  2.45244
C   2.50280 -0.65179  1.92445
H   3.38951 -1.23786  2.21986
H   2.66096  0.38397  2.27720
C   1.24101 -1.24369  2.56548
H   1.13977 -2.30849  2.29933
H   1.30387 -1.17961  3.67021
C  -1.24008 -1.24449  2.56536
H  -1.30300 -1.18064  3.67011
H  -1.13823 -2.30920  2.29904
C  -2.50222 -0.65321  1.92444
H  -2.66090  0.38244  2.27730
H  -3.38860 -1.23977  2.21988
C   3.08854 -2.12891 -0.66329
H   4.18126 -1.99001 -0.55716
C   2.67616 -3.38102  0.13172
H   3.13629 -4.27414 -0.32579
H   1.58181 -3.52616  0.10750
H   3.00522 -3.34913  1.18356
C   2.73427 -2.29228 -2.15409
H   3.20881 -3.20752 -2.54940
H   3.07544 -1.44677 -2.77086
H   1.64337 -2.38355 -2.28641
C   3.51738  0.86390 -0.38573
H   3.05632  1.70293  0.17163
C   4.95150  0.65432  0.13440
H   5.55339  1.56112 -0.05354
H   5.45685 -0.17766 -0.38502
H   4.98910  0.45478  1.21784
C   3.50021  1.20930 -1.88652
H   3.99331  2.18282 -2.05699
H   2.47434  1.25984 -2.28863
H   4.05775  0.46314 -2.47684
C  -3.51783  0.86212 -0.38551
H  -3.05738  1.70136  0.17205
C  -3.50076  1.20780 -1.88623
H  -3.99453  2.18100 -2.05659
H  -4.05769  0.46135 -2.47676
H  -2.47487  1.25916 -2.28820
C  -4.95184  0.65149  0.13450
H  -5.55431  1.55793 -0.05332
H  -4.98936  0.45176  1.21791
H  -5.45662 -0.18074 -0.38508
C  -3.08721 -2.13033 -0.66353
H  -4.18001 -1.99203 -0.55749
C  -2.67424 -3.38232  0.13137
H  -3.13389 -4.27563 -0.32626
H  -3.00338 -3.35071  1.18320
H  -1.57982 -3.52691  0.10720
C  -2.73274 -2.29338 -2.15432
H  -3.20690 -3.20875 -2.54977
H  -1.64179 -2.38421 -2.28655
H  -3.07416 -1.44792 -2.77102
H  -1.02106  2.24410  1.48481
H  -0.89885  4.68865  1.86863
C  -1.22097  4.01544 -0.70407
B  -0.00063  2.32718  0.82818
C  -0.00257  4.83575  1.25006
N  -0.00149  3.81391  0.14718
H   0.00031  1.56834 -0.21829
```

```
H  -0.00301  5.85120  0.82063
C   1.21826  4.01716 -0.70325
H   0.89338  4.68973  1.86937
H   1.01980  2.24533  1.48500
H   2.11127  3.90304 -0.07310
H   1.20358  5.02515 -1.14980
H   1.22999  3.25542 -1.49522
H  -1.20739  5.02347 -1.15057
H  -2.11423  3.90004 -0.07452
H  -1.23110  3.25369 -1.49606
```

TS(*syn-trans*)'

```
BP86 energy = -1006.64769912
Enthalpy 0K = -1005.978719
Enthalpy 298K = -1005.941304
Free energy 298K = -1006.043668
Low freq. = -94.1575 cm-1
Second freq. = 20.9640 cm-1

Ir  0.00000 -0.18677 -0.04442
H   0.00003 -0.99390  1.41242
H   0.00000  0.35848 -1.60712
P  -2.33937 -0.49890 -0.06626
P   2.33942 -0.49879 -0.06623
N   0.00007 -2.24257 -0.98065
H   0.00010 -2.05876 -1.99315
C  -2.49450 -2.19113 -0.90219
H  -3.38700 -2.73080 -0.54318
H  -2.63105 -2.01549 -1.98313
C  -1.23534 -3.02250 -0.65463
H  -1.15343 -3.30262  0.40804
H  -1.25647 -3.95430 -1.25366
C   1.23548 -3.02246 -0.65453
H   1.25667 -3.95428 -1.25353
H   1.15353 -3.30254  0.40814
C   2.49463 -2.19106 -0.90206
H   2.63125 -2.01549 -1.98300
H   3.38712 -2.73070 -0.54296
C  -3.29175 -0.73571  1.56193
H  -4.32182 -0.97602  1.23877
C  -2.75788 -1.91028  2.39878
H  -3.32868 -1.97826  3.34144
H  -1.69420 -1.76749  2.65428
H  -2.86869 -2.88000  1.88557
C  -3.31307  0.56225  2.38613
H  -3.96157  0.43589  3.27038
H  -3.69670  1.42518  1.81583
H  -2.29991  0.80376  2.75068
C  -3.46417  0.62338 -1.12062
H  -3.22807  1.63438 -0.73948
C  -3.05661  0.57710 -2.60590
H  -3.52817  1.41775 -3.14304
H  -3.40797 -0.34927 -3.09324
H  -1.96661  0.66124 -2.74206
C  -4.97215  0.35705 -0.95126
H  -5.53881  1.01883 -1.62957
H  -5.33344  0.55356  0.07056
H  -5.23864 -0.68087 -1.21811
C   3.46418  0.62344 -1.12068
H   3.22809  1.63447 -0.73961
C   4.97217  0.35712 -0.95136
H   5.53881  1.01887 -1.62971
H   5.23865 -0.68081 -1.21818
H   5.33349  0.55367  0.07044
C   3.05658  0.57708 -2.60595
H   3.52812  1.41771 -3.14314
H   1.96658  0.66121 -2.74210
H   3.40795 -0.34930 -3.09326
C   3.29185 -0.73547  1.56194
H   4.32189 -0.97589  1.23876
C   2.75794 -1.90993  2.39893
H   3.32878 -1.97786  3.34156
H   2.86866 -2.87970  1.88580
H   1.69429 -1.76705  2.65446
C   3.31329  0.56256  2.38601
H   3.96191  0.43630  3.27018
H   2.30018  0.80413  2.75067
H   3.69684  1.42545  1.81557
H   1.01966  2.28207 -1.47119
H   0.89481  4.56502 -1.23881
C   1.21681  3.37582  1.12160
B   0.00014  2.04658 -0.85756
C   0.00032  4.57310 -0.60125
N  -0.00017  3.33502  0.25395
H  -0.00007  0.99409  1.01316
H  -0.00000  5.46975  0.04119
C  -1.21792  3.37605  1.12052
H  -0.89348  4.56506 -1.23978
H  -1.01898  2.28216 -1.47184
H  -2.10759  3.38307  0.47439
H  -1.21010  4.28184  1.75041
H  -1.23755  2.48077  1.75563
H   1.20870  4.28171  1.75134
H   2.10706  3.38247  0.47627
H   1.23562  2.48063  1.75687
```

[*syn*-1-$H_3B·NMe_3$]$^{+}$'

```
BP86 energy = -1006.65253936
Enthalpy 0K = -1005.981975
Enthalpy 298K = -1005.944069
Free energy 298K = -1006.048267
Low freq. = 23.6426 cm-1
Second freq. = 28.3476 cm-1

Ir  0.00572 -0.25968 -0.08436
H   0.00365 -0.48928  1.58288
```

H	0.00799	-0.11664	-1.74776
P	-2.32485	-0.56889	-0.06826
P	2.35273	-0.51542	-0.05283
N	0.03777	-2.38905	-0.49640
H	0.06577	-2.39361	-1.52628
C	-2.45300	-2.38797	-0.56531
H	-3.36006	-2.85665	-0.14878
H	-2.52921	-2.43559	-1.66578
C	-1.20260	-3.12193	-0.08050
H	-1.17641	-3.17853	1.01916
H	-1.16322	-4.15305	-0.48237
C	1.27044	-3.09160	-0.01242
H	1.26157	-4.13471	-0.38447
H	1.20420	-3.11445	1.08709
C	2.52427	-2.35102	-0.47544
H	2.64407	-2.43850	-1.56974
H	3.42330	-2.78637	-0.00869
C	-3.31813	-0.52351	1.55782
H	-4.33421	-0.84513	1.26195
C	-2.78100	-1.50704	2.61116
H	-3.35330	-1.39104	3.54831
H	-1.71804	-1.30832	2.83182
H	-2.88798	-2.55945	2.29998
C	-3.39060	0.89993	2.13182
H	-4.05468	0.91873	3.01342
H	-3.78393	1.63353	1.40772
H	-2.39022	1.22635	2.46592
C	-3.43642	0.35038	-1.31293
H	-3.30588	1.40999	-1.01970
C	-2.90673	0.19165	-2.74987
H	-3.44143	0.88767	-3.41897
H	-3.08777	-0.82673	-3.13767
H	-1.82762	0.40264	-2.81381
C	-4.93019	-0.01692	-1.22117
H	-5.49154	0.53208	-1.99735
H	-5.38121	0.24287	-0.25036
H	-5.09904	-1.09287	-1.40309
C	3.45217	0.36130	-1.33831
H	3.33498	1.42995	-1.07543
C	4.94312	-0.01911	-1.25192
H	5.50199	0.50733	-2.04532
H	5.10104	-1.10032	-1.41120
H	5.40583	0.25815	-0.29154
C	2.90489	0.16800	-2.76387
H	3.44985	0.82855	-3.46014
H	1.83089	0.40372	-2.82462
H	3.05582	-0.86662	-3.12101
C	3.35211	-0.38078	1.56281
H	4.35928	-0.74376	1.28425
C	2.79743	-1.28370	2.67678
H	3.38025	-1.12951	3.60186
H	2.87119	-2.35524	2.42621
H	1.74106	-1.04593	2.88940
C	3.45895	1.07717	2.03678
H	4.12998	1.14099	2.91097
H	2.47070	1.45228	2.34871
H	3.86257	1.75046	1.26123
H	1.18539	2.24245	-1.22597
H	1.26126	4.60224	-1.14720
C	0.86873	3.62438	1.29142
B	0.07335	2.16644	-0.76457
C	0.22211	4.69610	-0.80402
N	-0.08528	3.56668	0.13944
H	-0.05073	1.35812	0.36799
H	0.07787	5.66041	-0.28874
C	-1.48074	3.74321	0.65227
H	-0.45089	4.62582	-1.67011
H	-0.84917	2.22193	-1.53966
H	-2.17266	3.74140	-0.20174
H	-1.56700	4.69553	1.20196
H	-1.72289	2.90666	1.32060
H	0.78635	4.59583	1.80683
H	1.89037	3.48915	0.91071
H	0.63036	2.81047	1.99151

TS(*syn-trans*)

BP86 energy = -1006.64984179
Enthalpy 0K = -1005.979361
Enthalpy 298K = -1005.942262
Free energy 298K = -1006.043538
Low freq. = -21.3802 cm-1
Second freq. = 29.9520 cm-1

Ir	-0.03566	-0.23515	-0.09308
H	-0.07559	-0.65712	1.53485
H	0.00696	0.17218	-1.71743
P	-2.38543	-0.44754	-0.07209
P	2.29008	-0.62629	-0.04804
N	-0.10786	-2.28975	-0.77425
H	-0.09395	-2.17267	-1.79784
C	-2.60104	-2.18731	-0.77223
H	-3.51427	-2.66667	-0.38208
H	-2.71216	-2.10127	-1.86723
C	-1.37148	-3.01870	-0.41841
H	-1.32204	-3.20890	0.66550
H	-1.38046	-3.99414	-0.94262
C	1.08539	-3.11564	-0.39424
H	1.02815	-4.09082	-0.91570
H	1.00362	-3.29753	0.68920
C	2.38147	-2.38509	-0.72913
H	2.51739	-2.31331	-1.82297
H	3.24929	-2.92969	-0.32209
C	-3.34294	-0.53405	1.57207
H	-4.37041	-0.80071	1.25953
C	-2.81932	-1.63699	2.50690
H	-3.38501	-1.61216	3.45478

H	-1.75167	-1.48367	2.73930
H	-2.94783	-2.64752	2.08383
C	-3.37372	0.82609	2.28967
H	-4.01098	0.75488	3.18853
H	-3.77750	1.63367	1.65705
H	-2.36144	1.11849	2.61367
C	-3.48178	0.63341	-1.19232
H	-3.31671	1.64955	-0.79017
C	-2.97571	0.60893	-2.64605
H	-3.47468	1.40557	-3.22442
H	-3.21792	-0.34659	-3.14509
H	-1.88594	0.76338	-2.70169
C	-4.98311	0.29504	-1.11271
H	-5.53938	0.93507	-1.81987
H	-5.41152	0.46917	-0.11307
H	-5.18655	-0.75203	-1.39877
C	3.54212	0.30303	-1.15438
H	3.64169	1.29039	-0.66200
C	4.93929	-0.34766	-1.20418
H	5.60216	0.25661	-1.84789
H	4.90182	-1.35989	-1.64205
H	5.42340	-0.41718	-0.21726
C	2.97946	0.51352	-2.57142
H	3.62944	1.20519	-3.13505
H	1.95268	0.91195	-2.55487
H	2.95677	-0.43558	-3.13628
C	3.19413	-0.73874	1.62413
H	4.24170	-0.98064	1.36623
C	2.63910	-1.85490	2.52446
H	3.16063	-1.83753	3.49749
H	2.79344	-2.85916	2.09553
H	1.56130	-1.70855	2.71142
C	3.15240	0.61676	2.34869
H	3.75169	0.57160	3.27455
H	2.11372	0.85927	2.63322
H	3.55986	1.44046	1.73641
H	-1.01814	2.33386	-1.12641
H	-0.88913	4.73724	-1.08308
C	1.47541	3.53915	-0.75118
B	-0.66863	2.29611	0.02184
C	-0.49284	4.80289	-0.06048
N	0.33584	3.57801	0.21938
H	0.12249	1.32177	0.56881
H	0.13248	5.70448	0.04723
C	0.86516	3.67173	1.61987
H	-1.32845	4.83667	0.65217
H	-1.53537	2.44060	0.85162
H	0.01501	3.69582	2.31600
H	1.47284	4.58450	1.73512
H	1.47998	2.78645	1.82984
H	2.11427	4.42891	-0.62355
H	1.06877	3.50665	-1.77135
H	2.05686	2.62581	-0.57124

[*trans*-1-$H_3B{\cdot}NMe_3$]$^+$

BP86 energy = -1006.65259749
Enthalpy 0K = -1005.982061
Enthalpy 298K = -1005.944053
Free energy 298K = -1006.048444
Low freq. = 26.0637 cm-1
Second freq. = 30.1362 cm-1

Ir	0.02524	-0.23485	-0.05327
H	0.02093	-0.93638	1.46362
H	0.01343	0.47801	-1.57958
P	-2.31397	-0.55448	-0.08492
P	2.36283	-0.49475	-0.03955
N	0.05785	-2.17189	-1.01028
H	0.09819	-1.93944	-2.01317
C	-2.44307	-2.18777	-1.02638
H	-3.33111	-2.76281	-0.71499
H	-2.55777	-1.95501	-2.09922
C	-1.17558	-3.00115	-0.78372
H	-1.12458	-3.34472	0.26164
H	-1.13328	-3.88968	-1.44311
C	1.27789	-2.98423	-0.67538
H	1.28043	-3.89958	-1.29843
H	1.16559	-3.28193	0.37956
C	2.55619	-2.17602	-0.87664
H	2.74215	-1.99784	-1.95059
H	3.42479	-2.72568	-0.47803
C	-3.28079	-0.91617	1.51639
H	-4.28348	-1.21095	1.15265
C	-2.68791	-2.09448	2.30717
H	-3.26335	-2.23502	3.23887
H	-1.63643	-1.90052	2.57872
H	-2.73913	-3.04645	1.75224
C	-3.41584	0.33055	2.40791
H	-4.05349	0.09206	3.27718
H	-3.87604	1.18380	1.88250
H	-2.43287	0.65402	2.78628
C	-3.46716	0.60608	-1.06794
H	-3.34011	1.57448	-0.54900
C	-2.98582	0.76839	-2.52162
H	-3.50464	1.62065	-2.99403
H	-3.22547	-0.12246	-3.12894
H	-1.89831	0.94022	-2.57741
C	-4.95447	0.20600	-1.01553
H	-5.54341	0.90059	-1.64007
H	-5.37519	0.24738	0.00128
H	-5.12171	-0.80849	-1.41839
C	3.54141	0.61303	-1.05767
H	3.52047	1.57794	-0.51502
C	4.99844	0.10969	-1.08859
H	5.61075	0.79326	-1.70237
H	5.07482	-0.89097	-1.54820

```
H   5.46317  0.06663 -0.09111
C   3.00378  0.84159 -2.48157
H   3.58528  1.63643 -2.97992
H   1.93811  1.12238 -2.48018
H   3.11011 -0.06669 -3.10146
C   3.25468 -0.66645  1.62929
H   4.31485 -0.84779  1.37261
C   2.74093 -1.85902  2.45224
H   3.27380 -1.89532  3.41844
H   2.91773 -2.82520  1.94959
H   1.66166 -1.76060  2.65764
C   3.13994  0.64914  2.42096
H   3.68593  0.56154  3.37626
H   2.08468  0.87216  2.65439
H   3.56989  1.50832  1.87764
H  -1.75690  2.03715  0.84845
H  -1.83865  4.50049  0.97739
C  -0.51631  3.50651 -1.15534
B  -0.58012  2.14378  1.05411
C  -0.74980  4.64809  0.99637
N  -0.09120  3.49652  0.28456
H   0.33480  1.23518  0.73671
H  -0.48843  5.59276  0.49196
C   1.39477  3.67517  0.35940
H  -0.40527  4.66805  2.03955
H  -0.27081  2.25965  2.22079
H   1.70557  3.62572  1.41267
H   1.68134  4.64760 -0.07395
H   1.87031  2.86244 -0.20589
H  -0.14136  4.41764 -1.65024
H  -1.61422  3.48474 -1.19629
H  -0.12231  2.59887 -1.63697
```

TS(*trans-anti*)'

```
BP86 energy = -1006.65184000
Enthalpy 0K = -1005.981365
Enthalpy 298K = -1005.944118
Free energy 298K = -1006.046198
Low freq. = -8.4218 cm-1
Second freq. = 27.5491 cm-1

Ir  0.00675 -0.25801 -0.09330
H  -0.00629 -0.64644  1.53303
H   0.00468  0.11009 -1.73619
P  -2.33607 -0.52949 -0.07058
P   2.34453 -0.54791 -0.02326
N   0.01245 -2.34127 -0.65862
H   0.09045 -2.28769 -1.68433
C  -2.48337 -2.28182 -0.76456
H  -3.40271 -2.78026 -0.41397
H  -2.54178 -2.20756 -1.86460
C  -1.25402 -3.08508 -0.34426
H  -1.24839 -3.25770  0.74350
H  -1.22398 -4.06965 -0.84991
C   1.20484 -3.09820 -0.14899
H   1.19070 -4.12125 -0.57281
H   1.07783 -3.17075  0.94323
C   2.50059 -2.37194 -0.50221
H   2.69303 -2.42487 -1.58844
H   3.35641 -2.84464  0.00744
C  -3.34545 -0.66060  1.54524
H  -4.31995 -1.05118  1.19430
C  -2.74667 -1.67201  2.53703
H  -3.33706 -1.66316  3.47000
H  -1.70399 -1.41250  2.78747
H  -2.77044 -2.70589  2.15411
C  -3.57501  0.69969  2.22716
H  -4.29646  0.57552  3.05384
H  -3.98490  1.46135  1.54284
H  -2.63795  1.09132  2.65223
C  -3.45473  0.51696 -1.20624
H  -3.32345  1.53772 -0.79784
C  -2.94706  0.50748 -2.65964
H  -3.46379  1.29112 -3.24046
H  -3.16802 -0.45396 -3.15639
H  -1.85984  0.67824 -2.71835
C  -4.94862  0.14303 -1.13929
H  -5.51740  0.77351 -1.84508
H  -5.38757  0.29471 -0.14092
H  -5.11984 -0.90657 -1.43632
C   3.49885  0.32914 -1.26632
H   3.44084  1.39009 -0.95505
C   4.96893 -0.12635 -1.18086
H   5.56586  0.40877 -1.94001
H   5.07472 -1.20514 -1.39055
H   5.42833  0.07991 -0.20136
C   2.96405  0.22087 -2.70556
H   3.53638  0.89301 -3.36822
H   1.89542  0.48300 -2.76821
H   3.08790 -0.80112 -3.10671
C   3.31049 -0.44661  1.61305
H   4.30549 -0.86201  1.36370
C   2.68258 -1.31430  2.71537
H   3.27204 -1.21380  3.64350
H   2.67353 -2.38590  2.45265
H   1.64615 -1.00103  2.92420
C   3.47525  1.01252  2.07329
H   4.07909  1.04724  2.99681
H   2.49413  1.46412  2.29535
H   3.98983  1.63763  1.32335
H  -1.44959  2.13418  1.18982
H  -1.35202  4.55970  1.45524
C  -0.86495  3.68445 -1.03956
B  -0.26804  2.13479  0.96875
C  -0.30645  4.66315  1.13347
N   0.01515  3.55649  0.16865
```

```
H   0.15086  1.40874 -0.08220
H  -0.15551  5.64010  0.64487
C   1.44712  3.68283 -0.25542
H   0.35147  4.57280  2.00896
H   0.47151  2.11972  1.91942
H   2.08923  3.56201  0.62833
H   1.62115  4.66938 -0.71619
H   1.66923  2.88957 -0.98380
H  -0.65890  4.63460 -1.55968
H  -1.91409  3.65850 -0.71336
H  -0.67069  2.83416 -1.70999
```

[*anti*-1-H$_3$B·NMe$_3$]$^{+\cdot}$

```
BP86 energy = -1006.65224976
Enthalpy 0K = -1005.982189
Enthalpy 298K = -1005.944204
Free energy 298K = -1006.048517
Low freq. = 21.1276 cm-1
Second freq. = 28.1429 cm-1

Ir  0.00488 -0.27470  0.00557
H   0.00549 -0.26451  1.68530
H  -0.04785  1.38161  0.25600
P  -2.32575 -0.58105  0.06493
P   2.34977 -0.52464  0.08067
N   0.02438 -2.43785 -0.09620
H  -0.00497 -2.77069  0.87861
C  -2.47847 -2.44887 -0.21730
H  -2.69618 -2.92950  0.75105
H  -3.32493 -2.67352 -0.88705
C  -1.18054 -2.98463 -0.81366
H  -1.14936 -4.09091 -0.77670
H  -1.07356 -2.66470 -1.86238
C   1.27464 -2.97954 -0.73371
H   1.22004 -2.69113 -1.79564
H   1.26029 -4.08486 -0.66895
C   2.52689 -2.40289 -0.08247
H   3.41887 -2.66264 -0.67627
H   2.67537 -2.82322  0.92675
C  -3.30337 -0.36436  1.68685
H  -4.29748 -0.79758  1.46631
C  -2.67505 -1.13038  2.86333
H  -3.26901 -0.95223  3.77679
H  -1.64307 -0.78630  3.04743
H  -2.65173 -2.22189  2.70645
C  -3.47053  1.12122  2.04778
H  -4.13880  1.22552  2.92005
H  -3.90198  1.71941  1.22768
H  -2.49523  1.55509  2.32943
C  -3.43840  0.19745 -1.26997
H  -3.26171  1.28268 -1.14423
C  -2.94913 -0.20283 -2.67400
H  -3.49568  0.37746 -3.43733
H  -3.14686 -1.27031 -2.87984
H  -1.87056 -0.01643 -2.79772
C  -4.93975 -0.09508 -1.09058
H  -5.50493  0.35916 -1.92318
H  -5.35112  0.31437 -0.15442
H  -5.15387 -1.17849 -1.11314
C   3.44979  0.17806 -1.30757
H   3.28843  1.26981 -1.22812
C   4.95054 -0.12439 -1.13681
H   5.50802  0.29681 -1.99168
H   5.15531 -1.20978 -1.12732
H   5.37982  0.31134 -0.22091
C   2.93992 -0.27540 -2.68743
H   3.48550  0.26451 -3.48053
H   1.86273 -0.07921 -2.80787
H   3.12167 -1.35336 -2.84953
C   3.34704 -0.18954  1.66898
H   4.32504 -0.67122  1.47789
C   2.71282 -0.83134  2.91384
H   3.33530 -0.61087  3.79869
H   2.63564 -1.92931  2.84133
H   1.70034 -0.43106  3.08978
C   3.56566  1.31698  1.89132
H   4.23921  1.47620  2.75116
H   2.60989  1.81357  2.12547
H   4.01741  1.82102  1.02052
H   1.15575  2.04368 -1.48032
H   1.20629  4.39672 -1.71805
C   0.90096  3.73928  0.83809
B   0.05439  2.02401 -0.98743
C   0.17861  4.53119 -1.35437
N  -0.08893  3.53066 -0.26502
H   0.01038 -0.36945 -1.66280
H   0.04513  5.55317 -0.96192
C  -1.46891  3.76367  0.26632
H  -0.52233  4.34893 -2.18104
H  -0.88558  1.98390 -1.74303
H  -2.18724  3.65229 -0.55808
H  -1.54656  4.77658  0.69589
H  -1.68368  3.01548  1.04082
H   0.82819  4.76859  1.22721
H   1.91076  3.55967  0.44397
H   0.69126  3.02148  1.64458
```

TS(*trans-anti*)

```
BP86 energy = -1006.64797818
Enthalpy 0K = -1005.978173
Enthalpy 298K = -1005.941093
Free energy 298K = -1006.041762
Low freq. = -64.6078 cm-1
Second freq. = 31.8722 cm-1

Ir  0.00004 -0.20501  0.07696
```

```
H   0.00005 -0.78698  1.63422
H   0.00013  1.09507  0.98483
P  -2.33959 -0.52446  0.06421
P   2.33968 -0.52434  0.06418
N   0.00009 -2.36216 -0.56685
H   0.00015 -2.87062  0.32850
C  -2.48176 -2.33116 -0.50162
H  -2.58089 -2.96107  0.39910
H  -3.39582 -2.47994 -1.10003
C  -1.24459 -2.74066 -1.30310
H  -1.24775 -3.83144 -1.49870
H  -1.21022 -2.21774 -2.27258
C   1.24476 -2.74056 -1.30319
H   1.21029 -2.21760 -2.27266
H   1.24798 -3.83133 -1.49883
C   2.48196 -2.33099 -0.50179
H   3.39597 -2.47965 -1.10029
H   2.58122 -2.96095  0.39887
C  -3.32895 -0.52571  1.68905
H  -4.32532 -0.91285  1.40378
C  -2.72515 -1.45209  2.75790
H  -3.34122 -1.40343  3.67276
H  -1.69911 -1.14243  3.01742
H  -2.69621 -2.50988  2.44740
C  -3.48156  0.90345  2.24010
H  -4.16148  0.90026  3.10946
H  -3.89339  1.61044  1.50068
H  -2.50703  1.28801  2.58590
C  -3.44767  0.40827 -1.17537
H  -3.22811  1.47211 -0.96932
C  -3.01393  0.10828 -2.62292
H  -3.54579  0.78424 -3.31445
H  -3.27781 -0.92287 -2.91772
H  -1.93297  0.25752 -2.77152
C  -4.95547  0.15464 -0.98826
H  -5.51635  0.69511 -1.77082
H  -5.33548  0.50267 -0.01509
H  -5.21154 -0.91480 -1.09361
C   3.44769  0.40859 -1.17529
H   3.22799  1.47239 -0.96920
C   4.95552  0.15516 -0.98811
H   5.51637  0.69571 -1.77063
H   5.21174 -0.91425 -1.09346
H   5.33543  0.50322 -0.01491
C   3.01406  0.10861 -2.62288
H   3.54589  0.78466 -3.31435
H   1.93310  0.25776 -2.77153
H   3.27806 -0.92249 -2.91771
C   3.32898 -0.52565  1.68905
H   4.32544 -0.91255  1.40377
C   2.72532 -1.45230  2.75774
H   3.34124 -1.40350  3.67271
H   2.69679 -2.51008  2.44717
H   1.69914 -1.14300  3.01714
C   3.48128  0.90347  2.24029
H   4.16119  0.90032  3.10966
H   2.50666  1.28776  2.58612
H   3.89296  1.61065  1.50096
H   1.01841  2.01914 -1.70716
H   0.89377  4.31015 -1.80547
C   1.21690  3.47412  0.70205
B  -0.00013  1.87450 -1.06349
C  -0.00036  4.41017 -1.17525
N  -0.00022  3.30818 -0.15049
H   0.00009  0.09359 -1.55134
H  -0.00037  5.39019 -0.66905
C  -1.21729  3.47390  0.70219
H  -0.89458  4.31006 -1.80534
H  -1.01853  2.01904 -1.70741
H  -2.10805  3.37737  0.06495
H  -1.21323  4.46472  1.18747
H  -1.22992  2.68520  1.46596
H   1.21271  4.46494  1.18735
H   2.10759  3.37780  0.06468
H   1.22979  2.68544  1.46582
```

[*anti*-1-$H_3B{\cdot}NMe_3$]$^+$

```
BP86 energy = -1006.66961416
Enthalpy 0K = -1005.998598
Enthalpy 298K = -1005.960124
Free energy 298K = -1006.066663
Low freq. = 19.8850 cm-1
Second freq. = 23.9025 cm-1
```

```
Ir  0.00644 -0.33505 -0.14383
H   0.02734 -1.86644 -0.53570
H   0.01700 -0.09078 -1.72232
P   2.32203 -0.55954  0.13518
P  -2.30265 -0.66189  0.05980
N   0.01569 -0.81218  2.06885
H   0.11468 -1.83631  2.10825
C   2.50862 -0.60516  2.03789
H   2.82322 -1.62143  2.32987
H   3.31713  0.07685  2.34901
C   1.20289 -0.21074  2.74922
H   1.23647 -0.52240  3.81271
H   1.06157  0.88058  2.71667
C  -1.29327 -0.46675  2.69866
H  -1.37028  0.63328  2.69399
H  -1.31786 -0.80709  3.75374
C  -2.43959 -1.10957  1.90619
H  -3.41322 -0.81462  2.32921
H  -2.37707 -2.20830  1.98525
C   3.10973 -2.20631 -0.40436
H   4.14852 -2.16512 -0.02430
C   2.40673 -3.42431  0.22173
```

```
H   2.93272 -4.34594 -0.08224
H   1.36391 -3.50361 -0.13025
H   2.40085 -3.40803  1.32494
C   3.12972 -2.33305 -1.93968
H   3.53072 -3.32080 -2.22677
H   3.76188 -1.57061 -2.41965
H   2.11149 -2.24567 -2.35575
C   3.54973  0.80303 -0.37540
H   3.19939  1.63994  0.26016
C   5.01786  0.49395 -0.03081
H   5.64005  1.38871 -0.20906
H   5.42190 -0.31191 -0.66663
H   5.16232  0.20162  1.02299
C   3.38248  1.21847 -1.84949
H   3.87855  2.18948 -2.02666
H   2.32109  1.30523 -2.13179
H   3.85082  0.49246 -2.53352
C  -3.52453  0.78442 -0.15239
H  -3.03330  1.58283  0.43717
C  -3.59214  1.23013 -1.62655
H  -4.09336  2.21118 -1.70508
H  -4.18464  0.52169 -2.22963
H  -2.59212  1.30713 -2.08678
C  -4.93603  0.54246  0.41292
H  -5.55266  1.44723  0.26787
H  -4.93937  0.32072  1.49215
H  -5.45156 -0.28295 -0.10736
C  -3.08374 -2.11334 -0.89945
H  -4.17621 -1.94307 -0.86008
C  -2.77587 -3.48419 -0.26989
H  -3.14683 -4.28259 -0.93575
H  -3.26124 -3.62911  0.70872
H  -1.68815 -3.63835 -0.15243
C  -2.61749 -2.08302 -2.36971
H  -3.12554 -2.88527 -2.93303
H  -1.52940 -2.24637 -2.43486
H  -2.83812 -1.12899 -2.87183
H  -0.69265  2.46957  1.65922
H  -0.30301  4.95081  1.71790
C  -1.45220  4.02592 -0.52415
B   0.14111  2.44836  0.77534
C   0.34204  4.97557  0.82833
N  -0.03488  3.83379 -0.07479
H  -0.10645  1.53127 -0.09354
H   0.21357  5.93237  0.29557
C   0.86043  3.84572 -1.27832
H   1.38926  4.85382  1.13718
H   1.28851  2.43643  1.16774
H   1.89922  3.71337 -0.94665
H   0.75749  4.80238 -1.81697
H   0.58037  3.01001 -1.93480
H  -1.54907  4.97727 -1.07342
H  -2.10484  4.03805  0.36009
H  -1.73283  3.18913 -1.17749
```

TS(A-B)TMAB

```
BP86 energy = -1006.63691372
Enthalpy 0K = -1005.969066
Enthalpy 298K = -1005.931740
Free energy 298K = -1006.032907
Low freq. = -650.4429 cm-1
Second freq. = 34.1567 cm-1

Ir -0.00238 -0.17231 -0.15904
H  -0.00833 -1.83874 -0.52751
H  -0.00530 -1.17759 -1.46594
P   2.34301 -0.42422  0.05377
P  -2.34745 -0.40991  0.05538
N  -0.00225 -0.74880  2.01319
H   0.00412  0.18276  2.46229
C   2.48874 -0.77938  1.90735
H   3.38695 -1.37512  2.13904
H   2.60722  0.20585  2.39314
C   1.23352 -1.47612  2.43593
H   1.15413 -2.50073  2.03752
H   1.26676 -1.54433  3.54137
C  -1.24560 -1.46036  2.44082
H  -1.27784 -1.52311  3.54659
H  -1.17803 -2.48766  2.04729
C  -2.49439 -0.75296  1.91076
H  -2.60156  0.23625  2.39119
H  -3.39836 -1.33797  2.14751
C   3.13331 -1.92447 -0.82418
H   4.22137 -1.75829 -0.70791
C   2.77849 -3.27136 -0.16897
H   3.27473 -4.08866 -0.72087
H   1.69198 -3.46493 -0.20891
H   3.11062 -3.34044  0.87984
C   2.79400 -1.95163 -2.32736
H   3.36605 -2.75813 -2.81852
H   3.03341 -1.00954 -2.84298
H   1.72286 -2.15933 -2.48827
C   3.63346  0.95622 -0.15831
H   3.13995  1.79489  0.36641
C   4.97914  0.67275  0.53782
H   5.64573  1.54401  0.41077
H   5.50061 -0.19390  0.09624
H   4.87613  0.49707  1.62011
C   3.84758  1.33373 -1.63596
H   4.38988  2.29303 -1.70537
H   2.90124  1.43554 -2.19184
H   4.46474  0.58000 -2.15319
C  -3.63154  0.97416 -0.16861
H  -3.13532  1.81596  0.34709
C  -3.84315  1.33981 -1.64978
H  -4.37610  2.30355 -1.72801
H  -4.46735  0.58690 -2.15976
H  -2.89644  1.42675 -2.20829
C  -4.97881  0.70307  0.52936
```

```
H  -5.64181  1.57574  0.39346
H  -4.87676  0.53772  1.61339
H  -5.50372 -0.16578  0.09630
C  -3.14540 -1.91174 -0.81377
H  -4.23251 -1.73779 -0.70000
C  -2.80045 -3.25661 -0.14927
H  -3.30145 -4.07415 -0.69650
H  -3.13444 -3.31666  0.89950
H  -1.71521 -3.45773 -0.18668
C  -2.80487 -1.95171 -2.31638
H  -3.38172 -2.75803 -2.80222
H  -1.73500 -2.16766 -2.47477
H  -3.03772 -1.01193 -2.83914
H  -0.96836  1.94019  1.61022
H  -0.76035  4.29404  1.84559
C  -1.22894  3.56514 -0.68659
B   0.02374  1.86818  0.88196
C   0.09534  4.40836  1.16616
N   0.01858  3.34005  0.10842
H  -0.00456  0.76363 -1.46159
H   0.07636  5.40685  0.69676
C   1.18209  3.51730 -0.81610
H   1.02541  4.27644  1.73548
H   1.04021  1.92758  1.57563
H   1.13844  4.51126 -1.29309
H   1.14592  2.72738 -1.57753
H   2.11175  3.43100 -0.23843
H  -1.18751  4.54710 -1.18799
H  -2.09145  3.53944 -0.00710
H  -1.31756  2.76247 -1.42991
```

BTMAB

BP86 energy = -1006.63830981
Enthalpy 0K = -1005.968937
Enthalpy 298K = -1005.931225
Free energy 298K = -1006.033470
Low freq. = 22.3879 cm-1
Second freq. = 37.0962 cm-1

```
Ir -0.00005 -0.15676 -0.15774
H   0.00050 -1.90065 -0.65918
H  -0.00052 -1.40343 -1.40417
P   2.34804 -0.39825  0.06300
P  -2.34793 -0.39836  0.06301
N   0.00003 -0.84678  1.99158
H  -0.00010  0.06477  2.47812
C   2.48697 -0.83971  1.89939
H   3.39423 -1.42948  2.10994
H   2.58519  0.12369  2.43186
C   1.23969 -1.57897  2.38736
H   1.17613 -2.58504  1.94077
H   1.27073 -1.70141  3.48846
C  -1.23957 -1.57922  2.38721
H  -1.27057 -1.70192  3.48829
H  -1.17585 -2.58517  1.94038
C  -2.48690 -0.84000  1.89938
H  -2.58520  0.12331  2.43199
H  -3.39413 -1.42985  2.10980
C   3.15612 -1.85674 -0.87379
H   4.24131 -1.68394 -0.74110
C   2.81795 -3.23662 -0.28087
H   3.33735 -4.02030 -0.85965
H   1.73691 -3.45399 -0.34290
H   3.13755 -3.34356  0.76847
C   2.83339 -1.82225 -2.38078
H   3.42278 -2.59799 -2.90037
H   3.06504 -0.85498 -2.85072
H   1.76808 -2.03692 -2.56778
C   3.65185  0.98279 -0.07561
H   3.14918  1.81441  0.45073
C   4.97317  0.67632  0.65710
H   5.65393  1.53977  0.55401
H   5.49656 -0.19434  0.22543
H   4.83754  0.49650  1.73490
C   3.92165  1.38955 -1.53656
H   4.46854  2.34826 -1.56520
H   2.99876  1.50628 -2.12743
H   4.55643  0.64458 -2.04520
C  -3.65167  0.98277 -0.07551
H  -3.14880  1.81434  0.45071
C  -3.92168  1.38946 -1.53645
H  -4.46848  2.34822 -1.56507
H  -4.55663  0.64450 -2.04490
H  -2.99890  1.50605 -2.12752
C  -4.97293  0.67645  0.65737
H  -5.65360  1.53998  0.55435
H  -4.83721  0.49660  1.73516
H  -5.49646 -0.19415  0.22575
C  -3.15609 -1.85678 -0.87381
H  -4.24127 -1.68401 -0.74094
C  -2.81780 -3.23672 -0.28108
H  -3.33728 -4.02033 -0.85989
H  -3.13726 -3.34380  0.76828
H  -1.73677 -3.45406 -0.34329
C  -2.83357 -1.82214 -2.38083
H  -3.42290 -2.59793 -2.90041
H  -1.76824 -2.03663 -2.56799
H  -3.06542 -0.85487 -2.85067
H  -1.00535  1.90027  1.60531
H  -0.89462  4.25550  1.77321
C  -1.20572  3.47496 -0.76471
B  -0.00029  1.82231  0.89459
C  -0.00001  4.36982  1.14591
N  -0.00004  3.28795  0.10090
H   0.00003  0.65911 -1.53951
H   0.00001  5.36180  0.66256
C   1.20564  3.47481 -0.76464
H   0.89463  4.25546  1.77317
H   1.00453  1.90004  1.60569
H   1.15938  4.45290 -1.27329
H   1.23046  2.66188 -1.50179
H   2.10475  3.43428 -0.13588
H  -1.15919  4.45294 -1.27354
```

```
H  -2.10480  3.43474 -0.13590
H  -1.23066  2.66190 -1.50171
```

TS(B-B)TMAB

```
BP86 energy = -1006.61602156
Enthalpy 0K = -1005.948573
Enthalpy 298K = -1005.911145
Free energy 298K = -1006.012996
Low freq. = -632.7961 cm-1
Second freq. = 30.7780 cm-1

Ir  0.00062 -0.14113 -0.13000
H   0.00316 -1.77729 -0.58536
H  -0.51702  0.38294 -1.59820
P   2.33416 -0.48167  0.06068
P  -2.33270 -0.48893  0.06005
N   0.00065 -0.63172  1.99798
H  -0.00193  0.30711  2.43862
C   2.49878 -0.71644  1.92056
H   3.38275 -1.32069  2.18228
H   2.63542  0.29343  2.34681
C   1.22841 -1.35985  2.46544
H   1.13420 -2.39812  2.10966
H   1.23511 -1.37366  3.57226
C  -1.22286 -1.36825  2.46317
H  -1.23014 -1.38482  3.56996
H  -1.12205 -2.40504  2.10476
C  -2.49670 -0.73105  1.91922
H  -2.63991  0.27660  2.34849
H  -3.37719 -1.34149  2.17824
C   3.07769 -2.02188 -0.77621
H   4.17204 -1.86702 -0.71622
C   2.71827 -3.32551 -0.04334
H   3.16622 -4.18096 -0.57811
H   1.62445 -3.47591 -0.03283
H   3.09650 -3.35620  0.99215
C   2.65084 -2.10284 -2.25431
H   3.12693 -2.97905 -2.72787
H   2.93890 -1.21379 -2.83696
H   1.55656 -2.22417 -2.32654
C   3.59372  0.90068 -0.26819
H   3.11287  1.75199  0.24751
C   4.97319  0.64932  0.37241
H   5.62211  1.52626  0.20115
H   5.48550 -0.21948 -0.07524
H   4.91875  0.49256  1.46144
C   3.73318  1.23150 -1.76495
H   4.27048  2.18761 -1.89154
H   2.75884  1.32332 -2.27511
H   4.31909  0.46092 -2.29323
C  -3.59489  0.89296 -0.26134
H  -3.11660  1.74131  0.26247
C  -3.73306  1.23447 -1.75569
H  -4.27498  2.18884 -1.87590
H  -4.31437  0.46524 -2.29094
H  -2.75808  1.33578 -2.26217
C  -4.97447  0.63330  0.37567
H  -5.62462  1.51078  0.21202
H  -4.92098  0.46638  1.46322
H  -5.48489 -0.23205 -0.08075
C  -3.07190 -2.02773 -0.78262
H  -4.16674 -1.87738 -0.72046
C  -2.70609 -3.33302 -0.05588
H  -3.15081 -4.18806 -0.59400
H  -3.08320 -3.36985  0.97983
H  -1.61159 -3.47852 -0.04692
C  -2.64644 -2.09998 -2.26158
H  -3.11919 -2.97607 -2.73870
H  -1.55174 -2.21620 -2.33563
H  -2.93914 -1.20945 -2.83968
H  -1.03580  2.01052  1.55326
H  -0.99683  4.36041  1.75945
C  -1.17847  3.63370 -0.79651
B  -0.01909  1.94717  0.85150
C  -0.07176  4.49958  1.18379
N  -0.00739  3.44379  0.11358
H   0.51750  0.37894 -1.60008
H  -0.05406  5.50609  0.73026
C   1.23828  3.66806 -0.68253
H   0.78893  4.37164  1.85431
H   0.97113  2.01327  1.59124
H   2.10338  3.60872 -0.00785
H   1.21386  4.66187 -1.16202
H   1.31499  2.88247 -1.44588
H  -1.14731  4.63577 -1.25812
H  -2.10209  3.52881 -0.21124
H  -1.14979  2.85889 -1.57396
```

Geometries without an outer-sphere **$H_3B{\cdot}NMeH_2$**:

$[1\text{-}NMeH_2]^+$

```
BP86 energy = -901.408412497
Enthalpy 0K = -900.824040
Enthalpy 298K = -900.790141
Free energy 298K = -900.886111
Low freq. = 34.3386 cm-1
Second freq. = 42.2581 cm-1

Ir -0.00001  0.10850 -0.16616
H   0.00000 -1.47154 -0.36789
H   0.00002  0.22200 -1.75796
P   2.31158 -0.11904  0.03779
P  -2.31163 -0.11902  0.03779
N  -0.00003 -0.20998  2.08648
H  -0.00002  0.71408  2.54189
C   2.50441 -0.31184  1.91828
H   3.38645 -0.91917  2.18345
H   2.67833  0.70087  2.32815
C   1.23995 -0.92961  2.52743
H   1.12900 -1.97301  2.19076
H   1.30520 -0.93724  3.63375
C  -1.24002 -0.92958  2.52743
```

```
H  -1.30528 -0.93721  3.63375
H  -1.12910 -1.97299  2.19076
C  -2.50446 -0.31178  1.91827
H  -2.67835  0.70095  2.32813
H  -3.38651 -0.91908  2.18345
C   3.15547 -1.63310 -0.73479
H   4.24028 -1.47276 -0.58703
C   2.75104 -2.93990 -0.02858
H   3.25185 -3.79120 -0.52141
H   1.66278 -3.11018 -0.10024
H   3.04436 -2.96293  1.03417
C   2.84359 -1.70997 -2.24176
H   3.34542 -2.59065 -2.67911
H   3.18726 -0.82287 -2.79589
H   1.75829 -1.81164 -2.40838
C   3.45539  1.36725 -0.30202
H   2.99160  2.14863  0.33624
C   4.90961  1.17266  0.16646
H   5.46972  2.11742  0.05317
H   5.43180  0.41426 -0.44094
H   4.98153  0.87291  1.22486
C   3.39163  1.83965 -1.76835
H   3.83643  2.84561 -1.86317
H   2.36052  1.87380 -2.16185
H   3.96746  1.17048 -2.42880
C  -3.45539  1.36730 -0.30201
H  -2.99157  2.14868  0.33623
C  -3.39164  1.83967 -1.76836
H  -3.83636  2.84566 -1.86320
H  -3.96754  1.17053 -2.42877
H  -2.36054  1.87371 -2.16188
C  -4.90961  1.17276  0.16649
H  -5.46969  2.11753  0.05319
H  -4.98151  0.87303  1.22489
H  -5.43184  0.41437 -0.44088
C  -3.15550 -1.63309 -0.73479
H  -4.24031 -1.47275 -0.58705
C  -2.75104 -2.93986 -0.02855
H  -3.25186 -3.79118 -0.52133
H  -3.04431 -2.96285  1.03422
H  -1.66277 -3.11014 -0.10024
C  -2.84360 -1.71000 -2.24177
H  -3.34544 -2.59068 -2.67908
H  -1.75830 -1.81167 -2.40836
H  -3.18726 -0.82291 -2.79592
H   0.00036  4.28135  0.91280
C   0.00032  3.19174  1.09222
H  -0.81408  2.66910 -0.73109
H   0.81383  2.66881 -0.73143
N  -0.00005  2.40135 -0.16650
H  -0.89392  2.93697  1.68282
H   0.89482  2.93681  1.68237
```

[1-H$_3$B·NMeH$_2$]$^+$

```
BP86 energy = -928.068251682
Enthalpy 0K = -927.451792
Enthalpy 298K = -927.415609
Free energy 298K = -927.518207
Low freq. = 20.0176 cm-1
Second freq. = 24.2403 cm-1

Ir  0.04429 -0.10371 -0.16867
H   0.15915 -1.66617 -0.42634
H   0.03808 -0.00325 -1.76288
P   2.37013 -0.14440  0.04150
P  -2.24718 -0.48123  0.04689
N   0.06256 -0.36613  2.07783
H  -0.03539  0.58946  2.45398
C   2.56368 -0.22270  1.92882
H   3.50028 -0.72249  2.22952
H   2.62445  0.82416  2.27915
C   1.36075 -0.93023  2.56737
H   1.35919 -1.99920  2.29846
H   1.41408 -0.86205  3.67225
C  -1.11118 -1.16841  2.54834
H  -1.17321 -1.14293  3.65471
H  -0.91815 -2.21137  2.24776
C  -2.42403 -0.66910  1.92957
H  -2.67857  0.33108  2.32545
H  -3.25144 -1.34719  2.19959
C   3.32414 -1.63666 -0.63918
H   4.39173 -1.40224 -0.46686
C   2.98073 -2.93441  0.11405
H   3.54109 -3.77541 -0.33024
H   1.90595 -3.17249  0.02986
H   3.25099 -2.89327  1.18231
C   3.07001 -1.79781 -2.15011
H   3.63589 -2.66596 -2.53097
H   3.38303 -0.91665 -2.73137
H   1.99910 -1.97010 -2.34950
C   3.38760  1.40205 -0.38780
H   2.88954  2.15725  0.25140
C   4.87120  1.31218  0.01330
H   5.35564  2.29494 -0.12426
H   5.42089  0.59232 -0.61668
H   5.01216  1.02254  1.06795
C   3.21072  1.83418 -1.85556
H   3.59950  2.85870 -1.99407
H   2.15219  1.81349 -2.16421
H   3.77464  1.18249 -2.54337
C  -3.48544  0.92225 -0.30742
H  -3.14109  1.68138  0.42361
C  -3.31943  1.50861 -1.72292
H  -3.85442  2.47306 -1.80302
H  -3.75104  0.84723 -2.49231
H  -2.25688  1.65432 -1.98682
C  -4.95197  0.57375  0.00331
H  -5.58037  1.47886 -0.07199
H  -5.08515  0.16573  1.01910
H  -5.35539 -0.15865 -0.71615
C  -2.99699 -2.06050 -0.68847
H  -4.07639 -2.01090 -0.45026
C  -2.41094 -3.32660 -0.03816
```

H -2.86184 -4.22007 -0.50396
H -2.61750 -3.38549 1.04350
H -1.31967 -3.38600 -0.19207
C -2.80623 -2.08816 -2.21674
H -3.22645 -3.02269 -2.62766
H -1.73540 -2.04823 -2.47810
H -3.30991 -1.24970 -2.72340
H -1.03755 2.28495 1.46379
H -1.74569 4.78802 1.33558
H -1.55369 3.52482 -0.73333
B -0.16792 2.54080 0.65494
C -0.98057 5.03298 0.58634
N -0.70186 3.80152 -0.22309
H 0.02506 1.70433 -0.30782
H -1.32875 5.85504 -0.05697
H -0.00670 4.02545 -0.94909
H -0.05512 5.32223 1.10271
H 0.89073 2.87072 1.14856

1-H$_3$

BP86 energy = -806.286502705
Enthalpy 0K = -805.764079
Enthalpy 298K = -805.733672
Free energy 298K = -805.822310
Low freq. = 30.8835 cm-1
Second freq. = 36.2971 cm-1

Ir 0.00001 0.19894 0.04885
H 0.00002 -0.99558 1.23427
H 0.00001 1.27278 1.22731
H -0.00002 1.49791 -1.02751
P -2.27998 0.02647 -0.11584
P 2.27999 0.02651 -0.11577
N 0.00004 -1.30281 -1.65286
H 0.00005 -0.71244 -2.49494
C -2.48751 -1.22322 -1.54484
H -3.39499 -1.84164 -1.43273
H -2.60768 -0.63271 -2.47252
C -1.24431 -2.11948 -1.64527
H -1.17631 -2.77679 -0.76208
H -1.29896 -2.76109 -2.55137
C 1.24441 -2.11946 -1.64522
H 1.29910 -2.76107 -2.55131
H 1.17641 -2.77676 -0.76202
C 2.48758 -1.22316 -1.54476
H 2.60773 -0.63262 -2.47243
H 3.39509 -1.84156 -1.43268
C -3.32911 -0.71237 1.29341
H -4.38213 -0.67973 0.95340
C -2.93621 -2.17370 1.57023
H -3.50534 -2.55865 2.43599
H -1.85938 -2.24149 1.80507
H -3.15043 -2.83876 0.71561
C -3.16354 0.14030 2.56492
H -3.71726 -0.31671 3.40553
H -3.54073 1.16775 2.43558
H -2.09710 0.20335 2.84165
C -3.28362 1.50393 -0.78348
H -2.81069 1.62636 -1.77811
C -4.78817 1.24322 -0.96911
H -5.25787 2.08415 -1.51143
H -5.30673 1.15711 0.00182
H -4.99279 0.32369 -1.54490
C -3.00354 2.79017 0.01314
H -3.39929 3.66722 -0.53090
H -1.91958 2.92415 0.16011
H -3.49046 2.77282 1.00361
C 3.28367 1.50395 -0.78339
H 2.81055 1.62657 -1.77791
C 3.00388 2.79011 0.01348
H 3.39959 3.66719 -0.53052
H 3.49105 2.77257 1.00383
H 1.91997 2.92417 0.16073
C 4.78816 1.24312 -0.96932
H 5.25785 2.08407 -1.51161
H 4.99259 0.32364 -1.54526
H 5.30688 1.15683 0.00152
C 3.32898 -0.71224 1.29363
H 4.38209 -0.67925 0.95395
C 2.93641 -2.17371 1.57016
H 3.50545 -2.55863 2.43600
H 3.15097 -2.83860 0.71550
H 1.85956 -2.24180 1.80480
C 3.16279 0.14023 2.56521
H 3.71641 -0.31673 3.40591
H 2.09624 0.20292 2.84162
H 3.53970 1.16781 2.43612

TS1

BP86 energy = -928.016574164
Enthalpy 0K = -927.408930
Enthalpy 298K = -927.373736
Free energy 298K = -927.472114
Low freq. = -579.7331 cm-1
Second freq. = 28.9323 cm-1

Ir 0.00509 0.00564 -0.03885
H 0.03392 -1.18297 1.09133
H -0.05471 0.91677 1.32046
P -2.34005 -0.31293 -0.10462
P 2.36242 -0.24335 -0.09078
N 0.03424 -1.53377 -1.59566
H 0.02884 -0.97425 -2.46171
C -2.45727 -1.53217 -1.54521
H -3.35664 -2.16547 -1.47617
H -2.55480 -0.92458 -2.46347
C -1.19619 -2.39411 -1.61088
H -1.12623 -3.06081 -0.73673
H -1.19209 -3.02053 -2.52350
C 1.28929 -2.35743 -1.60227
H 1.30980 -2.98447 -2.51428

```
H   1.23323 -3.02496 -0.72782
C   2.52466 -1.45930 -1.52966
H   2.61099 -0.85018 -2.44807
H   3.44118 -2.06673 -1.45357
C  -3.16030 -1.15241  1.39149
H  -4.24595 -1.00713  1.22966
C  -2.86763 -2.66153  1.46695
H  -3.37235 -3.08625  2.35187
H  -1.78627 -2.85044  1.58690
H  -3.23306 -3.21859  0.58870
C  -2.74255 -0.46020  2.70383
H  -3.28966 -0.91330  3.54868
H  -2.95589  0.61948  2.70988
H  -1.66333 -0.59737  2.88510
C  -3.52161  1.07386 -0.63121
H  -2.93499  1.59526 -1.41166
C  -4.84191  0.55705 -1.23524
H  -5.46169  1.41783 -1.54097
H  -5.43288 -0.01997 -0.50264
H  -4.69460 -0.06964 -2.12903
C  -3.79401  2.06158  0.51849
H  -4.30585  2.95496  0.12136
H  -2.87071  2.39804  1.01661
H  -4.45814  1.62036  1.28099
C   3.51754  1.17153 -0.60615
H   2.92920  1.67399 -1.39779
C   3.75362  2.17338  0.54125
H   4.23166  3.08470  0.14269
H   4.43344  1.75950  1.30514
H   2.82093  2.47179  1.04787
C   4.85864  0.68832 -1.19172
H   5.45946  1.56375 -1.49397
H   4.73795  0.05478 -2.08461
H   5.45561  0.12944 -0.44999
C   3.19292 -1.05951  1.41322
H   4.27556 -0.88560  1.25993
C   2.94085 -2.57584  1.48924
H   3.45052 -2.98530  2.37850
H   3.32776 -3.12390  0.61451
H   1.86420 -2.79370  1.60149
C   2.74585 -0.37644  2.72064
H   3.29687 -0.81361  3.57132
H   1.66896 -0.54088  2.89228
H   2.93093  0.70854  2.72707
H   1.05431  2.69621 -2.10164
H  -0.44431  5.33671  0.13979
H   1.24077  3.57006  0.20848
B   0.08765  2.75137 -1.37258
C  -0.65138  4.32449  0.53690
N   0.27335  3.30897  0.00451
H  -0.06237  1.17983 -1.31873
H  -0.60081  4.37419  1.63921
H   0.08131  1.65511  0.54910
H  -1.68046  4.06460  0.24783
H  -1.00183  2.91417 -1.87532
```

TS2

```
BP86 energy = -806.681066359
Enthalpy 0K = -806.153326
Enthalpy 298K = -806.119691
Free energy 298K = -806.216833
Low freq. = -80.6332 cm-1
Second freq. = 30.0453 cm-1

Ir  0.00001  0.18063  0.06854
H  -0.00000 -1.02829  1.02510
H   0.00001  1.01240  1.42655
H  -0.00310  2.97371 -2.27050
P  -2.32647  0.00732 -0.09881
P   2.32648  0.00736 -0.09882
N   0.00000 -1.01560 -1.85587
H  -0.00003 -0.35612 -2.64931
C  -2.50796 -1.00540 -1.70071
H  -3.39483 -1.66096 -1.66871
H  -2.67251 -0.28517 -2.52348
C  -1.24846 -1.84341 -1.96415
H  -1.15576 -2.64443 -1.21257
H  -1.30317 -2.32247 -2.96159
C   1.24849 -1.84336 -1.96419
H   1.30320 -2.32241 -2.96164
H   1.15584 -2.64440 -1.21263
C   2.50798 -1.00532 -1.70076
H   2.67248 -0.28506 -2.52351
H   3.39486 -1.66085 -1.66880
C  -3.29930 -0.95536  1.21073
H  -4.34504 -0.95688  0.84861
C  -2.80960 -2.41177  1.31019
H  -3.40627 -2.94778  2.06831
H  -1.75319 -2.46007  1.62739
H  -2.91676 -2.96603  0.36286
C  -3.22831 -0.24811  2.57676
H  -3.77268 -0.84412  3.32966
H  -3.68419  0.75406  2.55828
H  -2.18405 -0.14158  2.91599
C  -3.30172  1.58852 -0.51005
H  -2.87862  1.85275 -1.50037
C  -4.81454  1.35246 -0.67150
H  -5.29346  2.26839 -1.05887
H  -5.29270  1.12053  0.29504
H  -5.04809  0.53663 -1.37634
C  -2.98585  2.74406  0.45886
H  -3.41055  3.68330  0.06441
H  -1.90072  2.88420  0.59393
H  -3.42811  2.57839  1.45459
C   3.30169  1.58859 -0.51003
H   2.87855  1.85286 -1.50033
C   2.98585  2.74410  0.45892
H   3.41052  3.68336  0.06448
H   3.42815  2.57840  1.45463
H   1.90072  2.88422  0.59403
C   4.81451  1.35255 -0.67154
H   5.29340  2.26850 -1.05890
H   5.04805  0.53675 -1.37643
H   5.29271  1.12059  0.29497
```

```
C   3.29935 -0.95534  1.21068
H   4.34509 -0.95683  0.84855
C   2.80969 -2.41176  1.31010
H   3.40636 -2.94778  2.06821
H   2.91686 -2.96599  0.36276
H   1.75327 -2.46009  1.62729
C   3.22835 -0.24814  2.57673
H   3.77273 -0.84416  3.32961
H   2.18408 -0.14164  2.91596
H   3.68420  0.75405  2.55828
H   0.00085  2.82673 -3.00794
```

TS(A-B)

```
BP86 energy = -928.039516910
Enthalpy 0K = -927.426407
Enthalpy 298K = -927.391222
Free energy 298K = -927.489236
Low freq. = -656.1656 cm-1
Second freq. = 32.9156 cm-1

Ir -0.00001 -0.10311 -0.16744
H  -0.00002 -1.76486 -0.48879
H  -0.00002 -1.05949 -1.49821
P   2.33796 -0.21914  0.05633
P  -2.33798 -0.21910  0.05633
N  -0.00001 -0.65688  1.99382
H   0.00000  0.26992  2.45049
C   2.49415 -0.61904  1.90350
H   3.40486 -1.20199  2.11928
H   2.58933  0.35095  2.42344
C   1.25147 -1.36721  2.40561
H   1.20036 -2.38110  1.97631
H   1.27997 -1.46337  3.50896
C  -1.25150 -1.36718  2.40561
H  -1.27999 -1.46334  3.50897
H  -1.20041 -2.38108  1.97632
C  -2.49417 -0.61899  1.90351
H  -2.58933  0.35100  2.42345
H  -3.40489 -1.20193  2.11929
C   3.26974 -1.61298 -0.84394
H   4.33828 -1.41465 -0.63548
C   2.91740 -3.00710 -0.29427
H   3.48062 -3.77383 -0.85406
H   1.84369 -3.23074 -0.42140
H   3.17825 -3.12665  0.77020
C   3.03028 -1.55215 -2.36464
H   3.61907 -2.34113 -2.86400
H   3.32267 -0.58810 -2.80814
H   1.96725 -1.72450 -2.60429
C   3.42481  1.32935 -0.11158
H   2.90279  2.01837  0.58006
C   4.87243  1.14204  0.37905
H   5.39390  2.11538  0.37758
H   5.44662  0.46978 -0.28111
H   4.92728  0.74482  1.40583
C   3.38021  1.93299 -1.52883
H   3.76375  2.96850 -1.51191
H   2.35945  1.94086 -1.95144
H   4.01782  1.37033 -2.23087
C  -3.42481  1.32940 -0.11158
H  -2.90278  2.01840  0.58008
C  -3.38017  1.93306 -1.52882
H  -3.76370  2.96857 -1.51190
H  -4.01778  1.37042 -2.23088
H  -2.35940  1.94092 -1.95141
C  -4.87243  1.14211  0.37903
H  -5.39388  2.11546  0.37757
H  -4.92731  0.74487  1.40581
H  -5.44663  0.46987 -0.28114
C  -3.26978 -1.61293 -0.84393
H  -4.33832 -1.41459 -0.63547
C  -2.91746 -3.00706 -0.29426
H  -3.48069 -3.77378 -0.85406
H  -3.17830 -3.12660  0.77020
H  -1.84375 -3.23071 -0.42140
C  -3.03033 -1.55210 -2.36464
H  -3.61913 -2.34108 -2.86399
H  -1.96730 -1.72447 -2.60429
H  -3.32271 -0.58805 -2.80813
H  -0.99851  2.08050  1.55277
H  -0.89255  4.61863  1.13880
H  -0.81673  3.12436 -0.78175
B   0.00002  1.93277  0.85027
C   0.00013  4.54940  0.50199
N   0.00009  3.20565 -0.16065
H  -0.00001  0.83707 -1.47110
H   0.00018  5.36167 -0.24178
H   0.81694  3.12429 -0.78171
H   0.89279  4.61857  1.13884
H   0.99850  2.08043  1.55284
```

B

```
BP86 energy = -928.042368348
Enthalpy 0K = -927.427576
Enthalpy 298K = -927.392125
Free energy 298K = -927.490414
Low freq. = 36.0128 cm-1
Second freq. = 43.8137 cm-1

Ir -0.00002 -0.08553 -0.16659
H  -0.00009 -1.83552 -0.68960
H   0.00021 -1.34539 -1.42402
P   2.33746 -0.20348  0.06445
P  -2.33750 -0.20337  0.06447
N  -0.00003 -0.76490  1.96741
H   0.00010  0.14197  2.46142
C   2.48872 -0.69384  1.89149
H   3.40829 -1.26950  2.08806
H   2.56223  0.25170  2.45857
C   1.25360 -1.48418  2.34891
H   1.21697 -2.47586  1.86810
```

```
H   1.28275 -1.63963  3.44583
C  -1.25377 -1.48394  2.34903
H  -1.28292 -1.63927  3.44596
H  -1.21731 -2.47568  1.86832
C  -2.48880 -0.69346  1.89157
H  -2.56220  0.25212  2.45858
H  -3.40844 -1.26900  2.08818
C   3.27941 -1.55350 -0.89754
H   4.34665 -1.34167 -0.69574
C   2.96773 -2.97488 -0.39394
H   3.54827 -3.70661 -0.98227
H   1.90107 -3.22903 -0.52424
H   3.23657 -3.12431  0.66449
C   3.02439 -1.44630 -2.41363
H   3.63708 -2.19507 -2.94536
H   3.27744 -0.45698 -2.82357
H   1.96718 -1.64724 -2.65613
C   3.43477  1.34485 -0.03101
H   2.88987  2.02499  0.65128
C   4.86203  1.14467  0.51104
H   5.38936  2.11464  0.53214
H   5.45647  0.47125 -0.12996
H   4.87664  0.74387  1.53755
C   3.44777  1.96899 -1.44017
H   3.82949  3.00407 -1.39278
H   2.44574  1.98369 -1.90539
H   4.11425  1.41569 -2.12282
C  -3.43481  1.34494 -0.03131
H  -2.88991  2.02520  0.65084
C  -3.44782  1.96887 -1.44057
H  -3.82953  3.00397 -1.39326
H  -4.11431  1.41549 -2.12313
H  -2.44580  1.98353 -1.90580
C  -4.86208  1.14485  0.51075
H  -5.38936  2.11484  0.53182
H  -4.87671  0.74408  1.53727
H  -5.45657  0.47146 -0.13024
C  -3.27939 -1.55361 -0.89727
H  -4.34665 -1.34188 -0.69546
C  -2.96754 -2.97489 -0.39350
H  -3.54808 -3.70674 -0.98169
H  -3.23630 -3.12419  0.66497
H  -1.90087 -3.22898 -0.52383
C  -3.02439 -1.44651 -2.41337
H  -3.63686 -2.19551 -2.94504
H  -1.96712 -1.64715 -2.65584
H  -3.27777 -0.45731 -2.82340
H  -0.99760  2.04083  1.56594
H  -0.89203  4.57417  1.09487
H  -0.81586  3.03385 -0.79264
B  -0.00002  1.87842  0.86369
C   0.00033  4.49002  0.45934
N   0.00013  3.13272 -0.17290
H  -0.00006  0.71621 -1.56260
H   0.00020  5.28572 -0.30198
H   0.81604  3.03362 -0.79272
H   0.89293  4.57412  1.09451
H   0.99754  2.04063  1.56607
```

TS(B-C)

```
BP86 energy = -928.027714220
Enthalpy 0K = -927.416997
Enthalpy 298K = -927.379931
Free energy 298K = -927.482377
Low freq. = -123.8803 cm-1
Second freq. = 23.5398 cm-1

Ir  0.00001 -0.02156 -0.12371
H   0.00001 -2.24084 -2.74822
H   0.00001 -1.58001 -2.37705
P   2.32931 -0.22715  0.08352
P  -2.32929 -0.22719  0.08355
N   0.00003 -0.78944  1.99982
H   0.00002  0.08982  2.53981
C   2.49440 -0.76476  1.89864
H   3.39854 -1.37561  2.06087
H   2.61039  0.15950  2.49357
C   1.24683 -1.53349  2.35539
H   1.18613 -2.51546  1.85758
H   1.28521 -1.71551  3.44803
C  -1.24676 -1.53352  2.35539
H  -1.28513 -1.71556  3.44804
H  -1.18604 -2.51548  1.85757
C  -2.49435 -0.76481  1.89867
H  -2.61034  0.15945  2.49360
H  -3.39848 -1.37567  2.06090
C   3.24763 -1.60037 -0.86607
H   4.27559 -1.57121 -0.45625
C   2.65123 -2.99081 -0.57396
H   3.19675 -3.75396 -1.15619
H   1.58960 -3.04958 -0.86768
H   2.73809 -3.27407  0.48769
C   3.30421 -1.32268 -2.37949
H   3.81204 -2.16076 -2.88831
H   3.86543 -0.40705 -2.61912
H   2.29593 -1.22864 -2.81334
C   3.47368  1.29577  0.02816
H   2.99964  1.93231  0.79981
C   4.92565  1.00895  0.45148
H   5.48677  1.95716  0.52580
H   5.45075  0.38082 -0.28816
H   4.99258  0.51298  1.43366
C   3.41282  2.04687 -1.31767
H   3.75229  3.08950 -1.18782
H   2.39769  2.05311 -1.75426
H   4.07406  1.58881 -2.07108
C  -3.47369  1.29571  0.02822
H  -2.99963  1.93227  0.79985
C  -3.41291  2.04680 -1.31763
H  -3.75240  3.08942 -1.18776
H  -4.07417  1.58872 -2.07100
H  -2.39780  2.05308 -1.75426
C  -4.92563  1.00887  0.45160
```

```
H  -5.48677  1.95707  0.52594
H  -4.99251  0.51292  1.43379
H  -5.45076  0.38072 -0.28800
C  -3.24760 -1.60041 -0.86604
H  -4.27556 -1.57126 -0.45622
C  -2.65119 -2.99085 -0.57394
H  -3.19671 -3.75401 -1.15616
H  -2.73805 -3.27411  0.48772
H  -1.58957 -3.04962 -0.86766
C  -3.30418 -1.32272 -2.37946
H  -3.81200 -2.16081 -2.88827
H  -2.29590 -1.22868 -2.81331
H  -3.86542 -0.40710 -2.61909
H  -1.01417  2.04191  1.55959
H  -0.89351  4.58801  0.98213
H  -0.81756  2.94604 -0.82568
B  -0.00001  1.89335  0.88703
C  -0.00013  4.46938  0.35378
N  -0.00010  3.08138 -0.21319
H  -0.00001  0.59078 -1.61221
H  -0.00019  5.22206 -0.44926
H   0.81734  2.94611 -0.82574
H   0.89328  4.58808  0.98207
H   1.01420  2.04198  1.55950
```

C

```
BP86 energy = -926.855631964
Enthalpy 0K = -926.257339
Enthalpy 298K = -926.222040
Free energy 298K = -926.322899
Low freq. = 11.9831 cm-1
Second freq. = 29.1336 cm-1
```

```
Ir -0.00000  0.03372 -0.10007
P   2.31310 -0.22804  0.06246
P  -2.31311 -0.22801  0.06247
N  -0.00001 -0.81305  1.99186
H   0.00002  0.03815  2.57465
C   2.50029 -0.82340  1.85465
H   3.39663 -1.45042  1.99564
H   2.63760  0.08729  2.46620
C   1.24514 -1.58115  2.30903
H   1.16513 -2.54833  1.78574
H   1.29606 -1.79425  3.39534
C  -1.24518 -1.58109  2.30905
H  -1.29610 -1.79418  3.39536
H  -1.16521 -2.54829  1.78577
C  -2.50030 -0.82330  1.85468
H  -2.63756  0.08741  2.46620
H  -3.39667 -1.45027  1.99569
C   3.06381 -1.59243 -1.03523
H   4.16062 -1.49492 -0.92522
C   2.65073 -3.00335 -0.57584
H   3.09860 -3.75547 -1.24855
H   1.55499 -3.13774 -0.62189
H   2.99068 -3.23906  0.44583
C   2.67439 -1.36835 -2.51008
H   3.14044 -2.14619 -3.14012
H   2.99739 -0.38925 -2.89509
H   1.58037 -1.42929 -2.64003
C   3.53859  1.22637  0.00503
H   3.06827  1.92910  0.72033
C   4.95129  0.88080  0.51093
H   5.57160  1.79415  0.53241
H   5.46096  0.16205 -0.15353
H   4.94939  0.46403  1.53088
C   3.58628  1.89541 -1.38203
H   4.07237  2.88399 -1.30972
H   2.58231  2.03017 -1.82265
H   4.17744  1.29920 -2.09728
C  -3.53859  1.22641  0.00498
H  -3.06810  1.92930  0.72000
C  -3.58659  1.89510 -1.38223
H  -4.07261  2.88373 -1.31005
H  -4.17797  1.29876 -2.09719
H  -2.58273  2.02967 -1.82315
C  -4.95118  0.88097  0.51128
H  -5.57146  1.79434  0.53274
H  -4.94904  0.46440  1.53131
H  -5.46103  0.16210 -0.15291
C  -3.06383 -1.59242 -1.03518
H  -4.16063 -1.49501 -0.92503
C  -2.65057 -3.00332 -0.57590
H  -3.09848 -3.75546 -1.24857
H  -2.99034 -3.23909  0.44582
H  -1.55482 -3.13762 -0.62212
C  -2.67462 -1.36823 -2.51007
H  -3.14069 -2.14608 -3.14010
H  -1.58061 -1.42908 -2.64017
H  -2.99775 -0.38914 -2.89499
H  -1.01969  2.07870  1.59447
H  -0.89326  4.63169  0.99745
H  -0.81577  2.96886 -0.79467
B   0.00003  1.93202  0.92896
C   0.00019  4.50715  0.37035
N   0.00012  3.11323 -0.18250
H   0.00000  0.65321 -1.58597
H   0.00026  5.25204 -0.43991
H   0.81603  2.96876 -0.79464
H   0.89364  4.63159  0.99748
H   1.01972  2.07862  1.59454
```

TS(C-D)

```
BP86 energy = -926.829773062
Enthalpy 0K = -926.238781
Enthalpy 298K = -926.203768
Free energy 298K = -926.302134
Low freq. = -827.7057 cm-1
Second freq. = 27.2957 cm-1
```

```
Ir  0.01722  0.09640  0.04345
P  -2.30244 -0.24673 -0.05916
```

P	2.34636	-0.19067	-0.05370
N	0.03469	-0.95995	-1.92322
H	0.05523	-0.17963	-2.59647
C	-2.47228	-0.97848	-1.79890
H	-3.36187	-1.62272	-1.89694
H	-2.61226	-0.11832	-2.47882
C	-1.20980	-1.75934	-2.17990
H	-1.12695	-2.67859	-1.57743
H	-1.24429	-2.06151	-3.24452
C	1.27575	-1.77818	-2.13249
H	1.32423	-2.11851	-3.18514
H	1.17558	-2.67432	-1.49819
C	2.53847	-0.99260	-1.76151
H	2.69987	-0.16552	-2.47661
H	3.42342	-1.64854	-1.81456
C	-2.99879	-1.54345	1.15100
H	-4.09862	-1.44038	1.08326
C	-2.61679	-2.98369	0.76341
H	-3.04006	-3.68662	1.50188
H	-1.52101	-3.12360	0.77294
H	-3.00344	-3.28137	-0.22501
C	-2.54594	-1.23644	2.59240
H	-3.01792	-1.95212	3.28811
H	-2.81574	-0.22178	2.92211
H	-1.45139	-1.33688	2.68648
C	-3.57961	1.16054	-0.08519
H	-3.11816	1.86789	-0.80114
C	-4.95829	0.73867	-0.62745
H	-5.62093	1.62085	-0.66883
H	-5.45035	-0.00128	0.02713
H	-4.91014	0.31998	-1.64537
C	-3.70287	1.85114	1.28618
H	-4.27032	2.79214	1.17974
H	-2.72197	2.09196	1.72834
H	-4.25681	1.22282	2.00376
C	3.55140	1.27652	-0.17961
H	3.08134	1.86831	-0.99040
C	3.58239	2.13196	1.10212
H	4.06439	3.10304	0.89283
H	4.17553	1.64652	1.89474
H	2.57623	2.32542	1.51171
C	4.97223	0.88184	-0.62459
H	5.57891	1.79234	-0.77277
H	4.98510	0.32373	-1.57458
H	5.48600	0.27408	0.13969
C	3.10490	-1.41094	1.19205
H	4.19878	-1.35214	1.03544
C	2.64568	-2.85718	0.92787
H	3.08649	-3.52707	1.68645
H	2.96073	-3.23448	-0.05887
H	1.54765	-2.94906	1.00859
C	2.76964	-0.99238	2.63684
H	3.24836	-1.69162	3.34439
H	1.68049	-1.02190	2.80961
H	3.11987	0.02145	2.88318
H	0.96858	1.81122	-2.05059
H	-0.67563	4.90260	-0.58126
H	1.16459	3.29293	-0.05810
B	0.00094	1.99462	-1.33589
C	-0.76814	4.05440	0.12015
N	0.19751	2.99068	-0.21035
H	-0.00824	0.52528	1.59446
H	-0.61001	4.42330	1.14636
H	0.07594	1.75301	0.53684
H	-1.78806	3.65301	0.03891
H	-1.09539	2.03208	-1.85383

$[D{\cdot}H_2B{=}NMeH]^+$

BP86 energy = -926.880894035
Enthalpy 0K = -926.286008
Enthalpy 298K = -926.250728
Free energy 298K = -926.350306
Low freq. = 30.2200 cm-1
Second freq. = 32.5690 cm-1

Ir	0.07737	0.02653	0.16601
P	-2.22231	-0.44282	-0.02309
P	2.41309	-0.04459	-0.05560
N	0.08974	-0.21477	-2.08860
H	0.02436	0.74414	-2.46496
C	-2.40238	-0.51808	-1.90922
H	-3.23698	-1.16999	-2.21804
H	-2.65392	0.50803	-2.23575
C	-1.09990	-0.98961	-2.56985
H	-0.90883	-2.04577	-2.32004
H	-1.17902	-0.91555	-3.67217
C	1.37556	-0.81387	-2.57282
H	1.42830	-0.75556	-3.67779
H	1.35188	-1.88002	-2.29464
C	2.59217	-0.12650	-1.94151
H	2.67223	0.91852	-2.29360
H	3.51885	-0.64321	-2.24351
C	-2.82004	-2.11541	0.65097
H	-3.92359	-2.06439	0.59160
C	-2.34176	-3.30651	-0.19790
H	-2.71596	-4.24452	0.24767
H	-1.23959	-3.36937	-0.21418
H	-2.71127	-3.27111	-1.23608
C	-2.39514	-2.28959	2.12207
H	-2.79762	-3.24084	2.51164
H	-2.76088	-1.48093	2.77341
H	-1.29642	-2.31894	2.20855
C	-3.55893	0.81781	0.45539
H	-3.21078	1.71547	-0.09041
C	-4.97210	0.45331	-0.03592
H	-5.66178	1.29025	0.17150
H	-5.37565	-0.42993	0.48756
H	-5.01222	0.25819	-1.12016
C	-3.54138	1.13729	1.96247
H	-4.14736	2.03838	2.16244
H	-2.51990	1.31188	2.34130
H	-3.98223	0.31827	2.55478
C	3.40842	1.51665	0.36780

```
H   2.88181  2.27068 -0.25188
C   3.26981  1.93386  1.84376
H   3.65572  2.95945  1.97578
H   3.85846  1.27905  2.50727
H   2.22207  1.91532  2.18576
C   4.88182  1.45145 -0.07547
H   5.35494  2.43860  0.06674
H   4.99971  1.18172 -1.13808
H   5.45676  0.72966  0.52911
C   3.35865 -1.53254  0.63872
H   4.42716 -1.29058  0.48278
C   3.03139 -2.83043 -0.12102
H   3.59397 -3.66736  0.32762
H   3.31091 -2.78638 -1.18674
H   1.95781 -3.07667 -0.04564
C   3.08031 -1.69339  2.14546
H   3.65107 -2.55343  2.53660
H   2.00861 -1.88075  2.32596
H   3.37083 -0.80739  2.73064
H   0.23019  1.68859  0.65209
H  -1.21085  5.43627 -0.07892
H  -1.44959  3.27427  1.09784
B  -0.31482  2.30369 -0.37811
C  -1.58634  4.52582 -0.57708
N  -1.13854  3.31856  0.12495
H   0.14823 -1.58306  0.19587
H  -2.68865  4.58329 -0.61142
H   0.07069 -0.04611  1.76064
H  -1.20192  4.50847 -1.60744
H   0.10564  2.45526 -1.50493
```

D

```
BP86 energy = -805.504873029
Enthalpy 0K = -804.988205
Enthalpy 298K = -804.957453
Free energy 298K = -805.048023
Low freq. = 24.2876 cm-1
Second freq. = 27.0770 cm-1

Ir  0.00003  0.20112  0.02061
H  -0.00024 -0.81696  1.17754
H   0.00008  1.25199  1.21644
P  -2.32652  0.00735 -0.12339
P   2.32651  0.00737 -0.12334
N   0.00004 -1.30588 -1.67413
H   0.00008 -0.79581 -2.57122
C  -2.50680 -1.26741 -1.52644
H  -3.39517 -1.90581 -1.38303
H  -2.66872 -0.70092 -2.46222
C  -1.24839 -2.13981 -1.63727
H  -1.15661 -2.79657 -0.75670
H  -1.30205 -2.78598 -2.53558
C   1.24846 -2.13983 -1.63716
H   1.30215 -2.78605 -2.53544
H   1.15661 -2.79655 -0.75656
C   2.50692 -1.26749 -1.52629
H   2.66902 -0.70113 -2.46211
H   3.39520 -1.90597 -1.38266
C  -3.31578 -0.70840  1.32419
H  -4.35709 -0.77477  0.95533
C  -2.82874 -2.12412  1.68427
H  -3.43621 -2.51971  2.51647
H  -1.77676 -2.11456  2.01914
H  -2.92361 -2.83621  0.84750
C  -3.26026  0.22903  2.54441
H  -3.81390 -0.22525  3.38435
H  -3.71523  1.21150  2.34352
H  -2.22005  0.39507  2.87200
C  -3.28560  1.50066 -0.81002
H  -2.86128  1.58179 -1.83153
C  -4.80076  1.25484 -0.92898
H  -5.27094  2.09269 -1.47234
H  -5.28054  1.20142  0.06277
H  -5.04230  0.32968 -1.47922
C  -2.95587  2.80589 -0.06053
H  -3.37621  3.66466 -0.61184
H  -1.86879  2.95848  0.04194
H  -3.39255  2.82126  0.95123
C   3.28594  1.50047 -0.80998
H   2.86178  1.58156 -1.83156
C   2.95642  2.80585 -0.06071
H   3.37706  3.66444 -0.61205
H   3.39293  2.82124  0.95113
H   1.86937  2.95875  0.04158
C   4.80107  1.25430 -0.92869
H   5.27150  2.09192 -1.47218
H   5.04251  0.32896 -1.47867
H   5.28068  1.20101  0.06315
C   3.31539 -0.70834  1.32452
H   4.35684 -0.77469  0.95604
C   2.82832 -2.12409  1.68446
H   3.43547 -2.51960  2.51692
H   2.92362 -2.83620  0.84775
H   1.77620 -2.11464  2.01889
C   3.25943  0.22910  2.54472
H   3.81285 -0.22514  3.38483
H   2.21912  0.39510  2.87197
H   3.71444  1.21158  2.34394
```

Geometries with an outer-sphere $\mathbf{H_3B{\cdot}NMeH_2}$:

TS(1-H$_4$-1-H$_3$)

```
BP86 energy = -1025.10834586
Enthalpy 0K = -1024.414562
Enthalpy 298K = -1024.372535
Free energy 298K = -1024.492234
Low freq. = -581.3583 cm-1
Second freq. = 14.6546 cm-1

Ir  0.19880 -0.56525  0.03660
H   0.40815 -1.33628 -1.38391
H   0.63827 -1.95790  0.69859
```

```
H   0.17322 -0.44141  2.22705
P  -2.05064 -1.12247 -0.20040
P   2.36356  0.23398 -0.28576
N  -0.39695  1.30356 -1.09371
H  -0.60208  2.03697 -0.39524
C  -2.73370  0.42938 -1.04497
H  -3.62332  0.21126 -1.65971
H  -3.04764  1.11604 -0.23757
C  -1.64377  1.09560 -1.89140
H  -1.37377  0.45655 -2.74847
H  -2.00197  2.06871 -2.27923
C   0.72037  1.81341 -1.94540
H   0.45720  2.81272 -2.34304
H   0.82203  1.11635 -2.79368
C   2.02760  1.88666 -1.14936
H   1.93512  2.64583 -0.35124
H   2.86277  2.19180 -1.80218
C  -2.52263 -2.58967 -1.31515
H  -3.60404 -2.75529 -1.14559
C  -2.29904 -2.27775 -2.80585
H  -2.56490 -3.16249 -3.41051
H  -1.23779 -2.04724 -3.00398
H  -2.91668 -1.43870 -3.16640
C  -1.73679 -3.85136 -0.90878
H  -2.02594 -4.69519 -1.55979
H  -1.92218 -4.15592  0.13298
H  -0.65336 -3.68000 -1.02178
C  -3.18716 -1.25626  1.32321
H  -2.87947 -0.35805  1.89513
C  -4.69194 -1.15467  1.01205
H  -5.27229 -1.16110  1.95195
H  -5.04260 -2.01228  0.41237
H  -4.95256 -0.23078  0.47145
C  -2.88199 -2.50671  2.16989
H  -3.33078 -2.41063  3.17483
H  -1.79734 -2.68436  2.27689
H  -3.31317 -3.41371  1.71374
C   3.42577  0.79333  1.19352
H   2.68598  1.38347  1.77088
C   3.88913 -0.39188  2.06064
H   4.25818 -0.03245  3.03761
H   4.72162 -0.93619  1.58402
H   3.07747 -1.11863  2.23566
C   4.61049  1.70500  0.82338
H   5.12006  2.05034  1.74060
H   4.29967  2.60150  0.26327
H   5.36388  1.16938  0.22054
C   3.53997 -0.73324 -1.42478
H   4.53257 -0.25977 -1.29827
C   3.12760 -0.62773 -2.90419
H   3.82231 -1.22254 -3.52277
H   3.15554  0.40647 -3.28528
H   2.11334 -1.03505 -3.05939
C   3.61355 -2.21089 -0.99389
H   4.30602 -2.75792 -1.65776
H   2.61833 -2.68023 -1.06625
H   3.97142 -2.33936  0.03948
H  -0.03958  0.34230  1.49734
H  -0.50023 -1.57779  3.60666
H  -0.39187  0.69629  4.50991
H   0.58890 -0.42298  5.51483
C   0.48502  0.03154  4.51659
N   0.32187 -0.98222  3.44588
H   1.13674 -1.60422  3.37526
H   1.37948  0.63367  4.29897
H  -1.22060  4.33443 -1.24394
H  -2.88574  6.31990 -1.25785
H  -0.95405  6.43386  0.19759
B  -1.34466  4.29226 -0.02840
C  -2.99561  6.33663 -0.16484
N  -1.73300  5.80563  0.43686
H  -0.29491  4.02133  0.54533
H  -3.20549  7.36090  0.18062
H  -1.80825  5.83236  1.46256
H  -3.81764  5.66485  0.11816
H  -2.27090  3.57468  0.32828
```

[1-H$_3$·NMeH$_3$]$^+$

```
BP86 energy = -1025.10399757
Enthalpy 0K = -1024.407012
Enthalpy 298K = -1024.365375
Free energy 298K = -1024.481219
Low freq. = 13.9210 cm-1
Second freq. = 26.7303 cm-1

Ir -0.07085 -0.28180 -0.17974
H  -0.23425 -1.89889  0.00892
H  -0.11517 -0.66001 -1.74163
H   0.03788  1.33205 -1.85627
P   2.21151 -0.69294  0.08020
P  -2.38708 -0.22659  0.10661
N  -0.03854 -0.11129  2.07164
H   0.07939  0.88999  2.31728
C   2.42479 -0.49713  1.94975
H   3.25751 -1.10764  2.33748
H   2.67558  0.56376  2.13052
C   1.11904 -0.84535  2.66846
H   0.90229 -1.92287  2.57895
H   1.20054 -0.59934  3.74552
C  -1.32521 -0.55758  2.68861
H  -1.33935 -0.27924  3.76067
H  -1.35538 -1.65769  2.62106
C  -2.52763  0.06016  1.97049
H  -2.52717  1.15439  2.12372
H  -3.47244 -0.33431  2.38062
C   2.87509 -2.42287 -0.35348
H   3.97740 -2.33829 -0.30028
C   2.41394 -3.48999  0.65545
H   2.80564 -4.47659  0.35197
H   1.31262 -3.56150  0.67678
H   2.77522 -3.29684  1.67887
C   2.45775 -2.82100 -1.78229
H   2.85918 -3.82106 -2.02301
```

```
H   2.82916 -2.12149 -2.54814
H   1.35848 -2.85967 -1.86237
C   3.53661  0.52343 -0.55748
H   3.11402  1.49035 -0.21859
C   4.92839  0.33852  0.07627
H   5.60957  1.13508 -0.27252
H   5.38452 -0.62311 -0.21518
H   4.90517  0.38550  1.17644
C   3.63360  0.54738 -2.09295
H   4.21881  1.42192 -2.42898
H   2.63816  0.58940 -2.56795
H   4.14623 -0.34931 -2.47931
C  -3.45759  1.20189 -0.57339
H  -2.81123  2.07688 -0.36202
C  -3.67506  1.10360 -2.09501
H  -4.03806  2.06501 -2.49930
H  -4.43414  0.34288 -2.34325
H  -2.75485  0.81540 -2.63581
C  -4.80446  1.39107  0.15039
H  -5.33754  2.26369 -0.26744
H  -4.68501  1.56649  1.23080
H  -5.46516  0.51714  0.01670
C  -3.39167 -1.80042 -0.27012
H  -4.45292 -1.48604 -0.24018
C  -3.17335 -2.89767  0.78709
H  -3.76322 -3.79085  0.51658
H  -3.49139 -2.59084  1.79682
H  -2.11261 -3.20076  0.82608
C  -3.05349 -2.33927 -1.67354
H  -3.65020 -3.24563 -1.87784
H  -1.98515 -2.60608 -1.73254
H  -3.26630 -1.61495 -2.47542
H   0.10605  1.44085 -0.38084
H   0.46459  0.93189 -3.47005
H   1.61919  3.01432 -2.90652
H   0.47284  3.28875 -4.26442
C   0.56092  3.02549 -3.20082
N  -0.01664  1.67386 -2.94132
H  -1.01562  1.62356 -3.19198
H   0.01748  3.76103 -2.59189
H   0.39650  4.06730  3.62504
H   1.68123  5.34298  1.79033
H  -0.42879  4.32146  1.19073
B   0.31988  3.12806  2.85858
C   1.62957  4.51793  1.06605
N   0.39643  3.72675  1.34194
H  -0.76601  2.56031  2.93244
H   1.63893  4.91815  0.03896
H   0.31726  2.93105  0.67267
H   2.49923  3.86438  1.22412
H   1.27496  2.36341  2.96156
```

1-H$_3$

```
BP86 energy = -928.826787886
Enthalpy 0K = -928.206585
Enthalpy 298K = -928.169914
Free energy 298K = -928.272899
Low freq. = 29.6061 cm-1
Second freq. = 32.6236 cm-1

Ir  0.03131 -0.27809 -0.25114
P  -2.24963 -0.39364  0.01673
P   2.31626 -0.21897  0.00154
N   0.01893  0.30141  1.94685
C  -2.46989  0.12802  1.83197
H  -2.62922  1.22158  1.83248
H  -3.35444 -0.34028  2.29822
C  -1.20263 -0.20814  2.63182
H  -1.27427  0.21665  3.65504
H  -1.08536 -1.30161  2.72243
C   3.33117  1.14992 -0.85909
H   2.88024  2.04881 -0.39253
C  -3.37507  0.86665 -0.86767
H  -2.97290  1.81101 -0.44989
C  -3.15958 -2.06230 -0.11340
H  -4.23413 -1.84084  0.03355
C   2.51379  0.27404  1.82790
H   3.42323 -0.15853  2.28045
H   2.61634  1.37384  1.85494
C   3.04466  1.21597 -2.36965
H   3.52579  0.38383 -2.91178
H   3.44550  2.15477 -2.79463
H   1.96041  1.16978 -2.56371
C   3.35770 -1.80427 -0.17317
H   4.40982 -1.50292 -0.00715
C   1.26912 -0.14677  2.62342
H   1.21478 -1.24645  2.69712
H   1.31844  0.26690  3.65260
C  -3.13050  0.89155 -2.38668
H  -2.05097  0.91517 -2.60812
H  -3.60650  1.78282 -2.83532
H  -3.56492  0.00715 -2.88383
C   4.83768  1.14040 -0.54778
H   5.04588  1.09863  0.53520
H   5.31606  2.05584 -0.94155
H   5.34293  0.28280 -1.02516
C   3.19996 -2.38481 -1.59051
H   2.13541 -2.58131 -1.80422
H   3.75600 -3.33627 -1.67695
H   3.58048 -1.70420 -2.36947
C  -2.70042 -3.03945  0.98252
H  -1.60988 -3.20038  0.92235
H  -3.20067 -4.01568  0.84755
H  -2.94547 -2.68218  1.99729
C  -2.94017 -2.67463 -1.50911
H  -3.35931 -2.05034 -2.31480
H  -3.42254 -3.66745 -1.56937
H  -1.86104 -2.79732 -1.70382
C  -4.86944  0.76721 -0.51609
H  -5.32656 -0.14149 -0.94519
H  -5.41864  1.63118 -0.93302
H  -5.04849  0.75647  0.57282
C   2.96791 -2.84890  0.88665
```

```
H   3.17124 -2.50386  1.91488
H   3.54818 -3.77633  0.72967
H   1.89476 -3.09518  0.80795
H   0.04248 -0.71579 -1.78752
H   0.09580 -1.89652  0.12363
H  -0.04612  1.30400 -0.86947
H  -0.00522  1.33649  1.98755
H  -1.08484  3.16713  2.05339
H  -2.00869  4.08237 -0.22310
H   0.00575  2.71119 -0.21887
B  -0.03328  3.72640  1.74880
C  -1.04015  4.49577 -0.54006
N   0.03510  3.71523  0.12787
H   0.95862  3.11242  2.13771
H  -0.95656  4.44018 -1.63770
H   0.94746  4.08394 -0.17015
H  -0.98326  5.54207 -0.20515
H  -0.01237  4.89011  2.11599
```

[1-$H_3B{\cdot}NMeH_2$]$^+$

```
BP86 energy = -1050.60319513
Enthalpy 0K = -1049.889404
Enthalpy 298K = -1049.846668
Free energy 298K = -1049.964724
Low freq. = 19.3663 cm-1
Second freq. = 27.0449 cm-1
```

```
Ir  0.06489 -0.43320  0.16349
H   0.23811 -1.96968 -0.17931
H   0.15286 -0.91791  1.69719
P  -2.23038 -0.82202 -0.07949
P   2.38191 -0.33581 -0.19251
N  -0.04602  0.12824 -2.01089
H  -0.13397  1.16379 -2.02930
C  -2.51756 -0.22579 -1.85131
H  -3.38343 -0.72087 -2.32240
H  -2.74499  0.85354 -1.78221
C  -1.25429 -0.43027 -2.69442
H  -1.06744 -1.50421 -2.86137
H  -1.37763  0.05085 -3.68422
C   1.20416 -0.21858 -2.75624
H   1.18109  0.24926 -3.75995
H   1.20904 -1.31293 -2.89229
C   2.45230  0.23761 -1.99423
H   2.48672  1.34175 -1.96518
H   3.36565 -0.11236 -2.50406
C  -2.85583 -2.61439  0.00087
H  -3.96012 -2.53739  0.01137
C  -2.43284 -3.43052 -1.23433
H  -2.80800 -4.46415 -1.13676
H  -1.33322 -3.48686 -1.31690
H  -2.83702 -3.02485 -2.17611
C  -2.37651 -3.30457  1.29272
H  -2.76481 -4.33759  1.32654
H  -2.71632 -2.79171  2.20560
H  -1.27504 -3.34845  1.32263
C  -3.49631  0.21866  0.88877
H  -3.07331  1.23617  0.77148
C  -4.91692  0.20139  0.29358
H  -5.57035  0.88385  0.86548
H  -5.37176 -0.80247  0.35140
H  -4.94169  0.52868 -0.75789
C  -3.52244 -0.13217  2.38909
H  -4.02773  0.66634  2.96065
H  -2.51055 -0.28397  2.80676
H  -4.08495 -1.06266  2.57451
C   3.50672  0.94461  0.65355
H   2.92027  1.87271  0.51354
C   3.65859  0.69560  2.16503
H   4.09696  1.58420  2.65221
H   4.33418 -0.15150  2.37089
H   2.69361  0.47593  2.65331
C   4.88062  1.11534 -0.02191
H   5.43469  1.93588  0.46754
H   4.80242  1.36686 -1.09162
H   5.50174  0.20807  0.07290
C   3.33210 -1.98127 -0.11103
H   4.40100 -1.70033 -0.16901
C   3.00565 -2.90581 -1.29780
H   3.57534 -3.84621 -1.19807
H   3.27336 -2.46463 -2.27184
H   1.93394 -3.17105 -1.31344
C   3.06503 -2.70216  1.22455
H   3.65031 -3.63762  1.26467
H   1.99679 -2.95862  1.31999
H   3.34371 -2.09746  2.10144
H   1.02678  2.72911  1.18743
H   1.22371  2.90550  3.78393
H   0.43245  0.78810  2.83004
B  -0.07356  2.24869  1.34697
C   0.18684  2.57372  3.93026
N  -0.16827  1.63005  2.82564
H  -0.33891  1.34189  0.49194
H   0.08271  2.08600  4.91106
H  -1.11857  1.26080  2.97197
H  -0.47939  3.44631  3.87384
H  -0.99283  3.04522  1.21334
H  -0.40568  4.17325 -0.32408
H   0.42395  5.21162 -1.30044
N  -0.42918  4.63966 -1.24633
H   0.69486  2.91110 -2.23525
H  -1.61266  6.28827 -0.51962
B  -0.36565  3.50649 -2.41247
C  -1.62333  5.53420 -1.32228
H  -1.35065  2.79128 -2.24311
H  -0.39153  4.08181 -3.48276
H  -2.52440  4.91038 -1.24125
H  -1.62418  6.02591 -2.30495
```

TS1

```
BP86 energy = -1050.55966900
Enthalpy 0K = -1049.853099
```

Enthalpy 298K = -1049.811456
Free energy 298K = -1049.925232
Low freq. = -293.4419 cm-1
Second freq. = 23.3708 cm-1

```
Ir  0.08105 -0.38658  0.12394
H   0.27162 -1.95999 -0.23053
H   0.11921 -1.01843  1.65725
P  -2.23892 -0.78434 -0.15433
P   2.43568 -0.24148 -0.13450
N   0.03966  0.03980 -1.99586
H  -0.09012  1.07639 -2.03287
C  -2.43250 -0.31067 -1.96777
H  -3.27214 -0.84017 -2.44677
H  -2.65927  0.77022 -1.98204
C  -1.12668 -0.56973 -2.72038
H  -0.92574 -1.64947 -2.81323
H  -1.17571 -0.13728 -3.73742
C   1.32591 -0.26108 -2.71068
H   1.28005  0.18504 -3.72190
H   1.39179 -1.35593 -2.81853
C   2.52439  0.29345 -1.93892
H   2.48462  1.39739 -1.93727
H   3.47057 -0.01111 -2.41530
C  -2.83841 -2.57819  0.03203
H  -3.94193 -2.49841  0.07207
C  -2.44921 -3.46188 -1.16663
H  -2.82736 -4.48583 -1.00282
H  -1.35198 -3.53184 -1.26975
H  -2.87357 -3.10775 -2.12015
C  -2.32513 -3.19836  1.34651
H  -2.75541 -4.20732  1.47039
H  -2.59449 -2.60975  2.23683
H  -1.22700 -3.29980  1.32266
C  -3.53179  0.29524  0.71280
H  -3.07559  1.30088  0.63658
C  -4.89919  0.29553  0.00244
H  -5.58418  0.98112  0.53125
H  -5.37094 -0.70240  0.01643
H  -4.84136  0.63408 -1.04418
C  -3.67819 -0.06236  2.20310
H  -4.27861  0.71239  2.71047
H  -2.70659 -0.12530  2.71924
H  -4.20784 -1.02052  2.33880
C   3.45495  1.08381  0.75988
H   2.78305  1.96210  0.70700
C   3.69153  0.73377  2.24187
H   4.08587  1.61761  2.77259
H   4.44055 -0.06852  2.35241
H   2.77092  0.41064  2.75590
C   4.78295  1.41502  0.05188
H   5.29868  2.22151  0.60198
H   4.64302  1.76372 -0.98350
H   5.46804  0.54954  0.03462
C   3.42765 -1.85544  0.02891
H   4.48353 -1.52668  0.08298
C   3.26091 -2.78296 -1.18797
H   3.86394 -3.69494 -1.03637
H   3.59896 -2.32343 -2.13096
H   2.21028 -3.10235 -1.30393
C   3.05984 -2.60074  1.32707
H   3.70752 -3.48760  1.43882
H   2.01370 -2.94838  1.29017
H   3.18237 -1.98354  2.23022
H   0.76971  2.96188  1.03481
H  -0.68690  2.93589  4.29535
H   1.08695  1.68177  3.11050
B  -0.13141  2.25487  1.47595
C  -0.78022  1.89224  3.93169
N   0.11590  1.58246  2.80383
H  -0.16754  1.33793  0.37174
H  -0.58439  1.21886  4.78457
H   0.08200 -0.02973  1.82376
H  -1.82620  1.74854  3.61908
H  -1.25780  2.69667  1.32158
H  -0.53063  3.96558 -3.42243
H  -1.80406  5.77035 -2.04113
H   0.39339  5.07620 -1.28456
B  -0.38652  3.36250 -2.37758
C  -1.65943  5.25203 -1.08292
N  -0.40736  4.44108 -1.16717
H   0.71138  2.80891 -2.32080
H  -1.60692  5.98109 -0.25954
H  -0.25138  3.95121 -0.26642
H  -2.50204  4.56590 -0.92029
H  -1.31815  2.58834 -2.16300
```

Int[1-H$_4$]$^+$

BP86 energy = -929.250531912
Enthalpy 0K = -928.619519
Enthalpy 298K = -928.581833
Free energy 298K = -928.690559
Low freq. = 13.1955 cm-1
Second freq. = 20.8756 cm-1

```
Ir -0.10297 -0.64616 -0.17702
H  -0.19550 -1.38190  1.30867
H  -0.30763 -2.22557 -0.29506
P   2.24303 -0.76423 -0.03356
P  -2.40101 -0.16037 -0.02718
N   0.13212  1.14888  1.09409
H   0.22986  1.95218  0.44505
C   2.59624  0.75426  1.04449
H   3.49162  0.60636  1.67109
H   2.80570  1.59406  0.35795
C   1.38352  1.09370  1.91814
H   1.22552  0.32466  2.69212
H   1.52995  2.07253  2.41198
C  -1.08833  1.40989  1.92465
H  -0.97767  2.39055  2.42443
H  -1.12991  0.62145  2.69405
C  -2.35018  1.39637  1.05434
H  -2.34013  2.26138  0.36741
```

```
H  -3.25198  1.48270  1.68321
C   3.06624 -2.22573  0.84828
H   4.15247 -2.03165  0.76192
C   2.68976 -2.27804  2.33984
H   3.18772 -3.14272  2.81180
H   1.60181 -2.40645  2.47250
H   3.00753 -1.37883  2.89335
C   2.72657 -3.55551  0.14925
H   3.23228 -4.38711  0.66992
H   3.04700 -3.57972 -0.90383
H   1.64096 -3.74980  0.18038
C   3.24749 -0.38798 -1.59794
H   2.85035  0.61426 -1.85435
C   4.76225 -0.27927 -1.34176
H   5.26454  0.10530 -2.24642
H   5.20851 -1.26365 -1.12047
H   5.00945  0.40477 -0.51258
C   2.92615 -1.33305 -2.76950
H   3.37566 -0.93618 -3.69623
H   1.84101 -1.42966 -2.93349
H   3.34519 -2.34063 -2.61215
C  -3.27994  0.46663 -1.58692
H  -2.63760  1.33238 -1.84365
C  -3.21853 -0.52645 -2.76119
H  -3.55364 -0.02452 -3.68540
H  -3.88322 -1.39183 -2.60352
H  -2.19592 -0.89991 -2.93005
C  -4.71382  0.96316 -1.32373
H  -5.10321  1.46736 -2.22526
H  -4.77200  1.68525 -0.49193
H  -5.39885  0.12733 -1.10193
C  -3.57081 -1.36128  0.85668
H  -4.57028 -0.89270  0.77576
C  -3.21362 -1.51207  2.34627
H  -3.91450 -2.22112  2.81994
H  -3.28742 -0.56261  2.90218
H  -2.19429 -1.91530  2.47299
C  -3.58953 -2.73180  0.15432
H  -4.29140 -3.40543  0.67584
H  -2.59125 -3.20078  0.18033
H  -3.90934 -2.66910 -0.89736
H   0.03747  0.44780 -1.41650
H  -0.18818 -1.28941 -1.63742
H  -0.07816  4.36362  1.13438
H   0.74107  6.83216  1.20689
H  -0.20191  5.98191 -0.85705
B   0.62237  4.21722  0.14392
C   1.35028  6.73572  0.29782
N   0.73623  5.67663 -0.56453
H   0.10737  3.47703 -0.69299
H   1.40101  7.70030 -0.23036
H   1.28282  5.58742 -1.43183
H   2.35882  6.40436  0.58052
H   1.76386  3.87706  0.42187
```

$[1\text{-}H_4]^+$

```
BP86 energy = -929.251289400
Enthalpy 0K = -928.619947
Enthalpy 298K = -928.582077
Free energy 298K = -928.691261
Low freq. = 13.0194 cm-1
Second freq. = 17.3039 cm-1

Ir  0.05904 -0.52085 -0.26571
H   0.14388 -1.60060  0.92447
H   0.15022 -1.82303 -1.19024
H   0.00606  0.06502 -1.89533
P   2.37907 -0.27670 -0.01444
P  -2.27017 -0.62347  0.00828
N  -0.05200  1.03695  1.36202
H  -0.13309  1.96199  0.90416
C   2.45219  1.08777  1.30054
H   3.36061  1.01189  1.92187
H   2.50502  2.04646  0.75338
C   1.19852  1.05390  2.18440
H   1.18824  0.14217  2.80436
H   1.18501  1.92764  2.86525
C  -1.27457  0.85096  2.20700
H  -1.38706  1.71551  2.88989
H  -1.10837 -0.05084  2.81956
C  -2.53170  0.69690  1.34231
H  -2.73569  1.64396  0.81140
H  -3.40666  0.47164  1.97520
C   3.39974 -1.72068  0.66703
H   4.44964 -1.37211  0.63548
C   3.03180 -2.04352  2.12661
H   3.64047 -2.89520  2.47676
H   1.97124 -2.33643  2.21524
H   3.22449 -1.20276  2.81357
C   3.24218 -2.96636 -0.22557
H   3.85131 -3.79222  0.18130
H   3.56812 -2.79280 -1.26274
H   2.19033 -3.29690 -0.25210
C   3.36366  0.49883 -1.44156
H   2.79785  1.44028 -1.59342
C   4.81794  0.84863 -1.07505
H   5.27883  1.42816 -1.89393
H   5.43124 -0.05820 -0.93873
H   4.89480  1.45649 -0.15810
C   3.28015 -0.32030 -2.74312
H   3.65526  0.28432 -3.58698
H   2.24796 -0.62944 -2.97552
H   3.90439 -1.22768 -2.69263
C  -3.36626  0.01967 -1.40202
H  -2.95030  1.03892 -1.53417
C  -3.16912 -0.75252 -2.71974
H  -3.63908 -0.19638 -3.54942
H  -3.64642 -1.74587 -2.68488
H  -2.10387 -0.89540 -2.96435
C  -4.85407  0.13687 -1.02195
H  -5.40355  0.65556 -1.82689
H  -5.01428  0.70873 -0.09281
H  -5.32184 -0.85503 -0.90079
```

```
C  -3.05539 -2.21380  0.67413
H  -4.14550 -2.02331  0.66060
C  -2.62221 -2.49904  2.12336
H  -3.09278 -3.43611  2.46816
H  -2.92598 -1.70497  2.82544
H  -1.52869 -2.63226  2.19307
C  -2.72945 -3.40906 -0.24132
H  -3.20383 -4.32196  0.15901
H  -1.64085 -3.58034 -0.28700
H  -3.09343 -3.26987 -1.27107
H  -0.06007  0.82752 -1.41152
H  -1.22326  3.89139  1.16305
H  -0.39176  6.34050  1.47743
H  -1.91286  5.84572 -0.34215
B  -0.85825  3.96587 -0.00319
C  -0.08211  6.44853  0.42888
N  -0.93191  5.54058 -0.40382
H  -1.60535  3.37710 -0.77090
H  -0.18488  7.49698  0.10920
H  -0.67148  5.64478 -1.39398
H   0.96328  6.12485  0.33122
H   0.31382  3.62740 -0.15250
```

TS(1-H_4-D)/TS2

```
BP86 energy = -929.219984753
Enthalpy 0K = -928.594287
Enthalpy 298K = -928.554957
Free energy 298K = -928.667360
Low freq. = -89.7881 cm-1
Second freq. = -8.0461 cm-1
```

```
Ir -0.12452 -0.54739  0.21194
H  -0.33573 -1.85135 -0.58212
H  -0.29954 -1.55317  1.43864
H   0.32898  1.92897  3.21538
P  -2.39620 -0.05501 -0.02162
P   2.18513 -0.82222 -0.00139
N   0.12653  0.90883 -1.48839
H   0.27971  1.85145 -1.08356
C  -2.36200  1.19918 -1.44927
H  -3.27449  1.14078 -2.06697
H  -2.33747  2.20333 -0.98913
C  -1.11549  0.99410 -2.32199
H  -1.18673  0.04971 -2.88707
H  -1.02099  1.82119 -3.05328
C   1.33722  0.58682 -2.31023
H   1.52356  1.40022 -3.03921
H   1.10336 -0.32887 -2.87866
C   2.57454  0.37444 -1.42639
H   2.87477  1.33151 -0.96315
H   3.42365  0.02162 -2.03639
C  -3.60388 -1.40594 -0.57609
H  -4.58020 -0.89559 -0.68282
C  -3.19943 -1.98433 -1.94474
H  -3.92468 -2.76058 -2.24463
H  -2.20413 -2.46006 -1.90118
H  -3.18779 -1.22422 -2.74346
C  -3.71666 -2.51577  0.48528
H  -4.39432 -3.30826  0.12251
H  -4.12175 -2.14712  1.44051
H  -2.73329 -2.97300  0.68733
C  -3.23323  0.94219  1.36473
H  -2.62520  1.86916  1.35430
C  -4.69705  1.30953  1.06065
H  -5.07143  2.01283  1.82499
H  -5.35150  0.42188  1.09010
H  -4.82023  1.79520  0.07780
C  -3.06681  0.28688  2.74871
H  -3.37513  0.99801  3.53485
H  -2.02224 -0.00891  2.93807
H  -3.69665 -0.61125  2.85690
C   3.28917 -0.15165  1.39488
H   3.01257  0.92184  1.38485
C   2.91070 -0.72240  2.77458
H   3.42223 -0.14777  3.56635
H   3.22197 -1.77425  2.88342
H   1.82466 -0.67174  2.95481
C   4.79548 -0.27571  1.10173
H   5.37117  0.26598  1.87258
H   5.07644  0.14777  0.12260
H   5.12782 -1.32735  1.12969
C   2.88736 -2.49606 -0.54589
H   3.97974 -2.33908 -0.62871
C   2.34247 -2.90497 -1.92685
H   2.77616 -3.87685 -2.21983
H   2.59902 -2.18229 -2.71915
H   1.24518 -3.02533 -1.90599
C   2.60167 -3.58228  0.50757
H   2.98763 -4.55376  0.15243
H   1.51779 -3.68832  0.68342
H   3.08243 -3.36993  1.47512
H   0.35315  2.32430  2.57570
H   1.59428  3.31438  0.22681
H   2.93047  5.54379 -0.07877
H   0.86632  5.58076  1.19093
B   0.61468  3.91258 -0.19863
C   2.05359  6.10421 -0.43082
N   0.83063  5.48095  0.16720
H  -0.41939  3.56305  0.35929
H   2.14118  7.16381 -0.14542
H   0.00061  6.00952 -0.13421
H   1.98630  6.01060 -1.52337
H   0.52654  3.85374 -1.42011
```

TS(A-B)

```
BP86 energy = -1050.57186824
Enthalpy 0K = -1049.861349
Enthalpy 298K = -1049.819591
Free energy 298K = -1049.933481
Low freq. = -607.0644 cm-1
Second freq. = 23.1766 cm-1
```

Ir	0.11465	-0.56816	0.19025
H	0.29995	-1.99214	-0.69753
H	0.33414	-2.14220	0.55580
P	-2.21041	-0.85513	-0.04881
P	2.42723	-0.22550	-0.09390
N	-0.03311	0.37406	-1.82121
H	-0.17080	1.39398	-1.68188
C	-2.49278	-0.01318	-1.71845
H	-3.35904	-0.43883	-2.25176
H	-2.71887	1.04645	-1.50263
C	-1.23112	-0.09599	-2.58596
H	-1.03268	-1.13251	-2.90482
H	-1.35308	0.53430	-3.48740
C	1.23278	0.24111	-2.60945
H	1.16466	0.88007	-3.51045
H	1.31278	-0.81095	-2.92893
C	2.44249	0.65941	-1.76500
H	2.38154	1.74109	-1.54879
H	3.38092	0.48042	-2.31575
C	-2.88506	-2.62547	-0.23777
H	-3.98517	-2.50472	-0.23418
C	-2.47239	-3.27597	-1.57069
H	-2.89150	-4.29578	-1.62474
H	-1.37508	-3.36794	-1.65077
H	-2.84353	-2.72493	-2.45016
C	-2.47084	-3.51619	0.94931
H	-2.92491	-4.51620	0.83759
H	-2.78852	-3.11346	1.92320
H	-1.37585	-3.64718	0.98066
C	-3.44674	0.04410	1.07895
H	-3.05326	1.07763	1.03193
C	-4.89464	0.04415	0.55434
H	-5.52309	0.68690	1.19595
H	-5.34118	-0.96464	0.57713
H	-4.97394	0.43112	-0.47445
C	-3.37650	-0.44004	2.54016
H	-3.87270	0.28708	3.20707
H	-2.33753	-0.58549	2.88799
H	-3.89877	-1.40205	2.67419
C	3.40020	0.97074	1.01523
H	2.74257	1.86053	0.98359
C	3.49295	0.48368	2.47415
H	3.79080	1.31651	3.13545
H	4.25610	-0.30375	2.59133
H	2.53795	0.06642	2.84177
C	4.78371	1.35999	0.46204
H	5.23099	2.14470	1.09756
H	4.73474	1.75875	-0.56413
H	5.48411	0.50734	0.46630
C	3.54417	-1.75356	-0.30260
H	4.57267	-1.34510	-0.31841
C	3.29384	-2.49104	-1.63044
H	3.96597	-3.36429	-1.69585
H	3.49069	-1.86233	-2.51404
H	2.25835	-2.86920	-1.69140
C	3.40329	-2.72105	0.88829
H	4.10662	-3.56312	0.76606
H	2.38407	-3.14052	0.93704
H	3.61733	-2.24648	1.85810
H	0.74241	2.32925	0.51687
H	0.45510	3.72122	2.70596
H	0.69605	1.30572	2.85900
B	-0.17049	1.58467	0.87901
C	-0.36287	3.08619	3.07475
N	-0.18253	1.71311	2.51006
H	0.15729	-0.76097	1.78736
H	-0.35759	3.07335	4.17625
H	-0.92144	1.09068	2.86535
H	-1.32039	3.48556	2.71155
H	-1.24881	2.06426	0.52111
H	-0.58650	4.46852	-3.39923
H	-1.93392	5.98762	-1.77724
H	0.21387	5.13960	-1.03179
B	-0.49360	3.67235	-2.48589
C	-1.85107	5.28982	-0.93229
N	-0.59271	4.50465	-1.09168
H	0.60601	3.12530	-2.46419
H	-1.86481	5.84543	0.01913
H	-0.49423	3.82625	-0.31316
H	-2.69793	4.59046	-0.97266
H	-1.43062	2.87813	-2.46666

B

BP86 energy = -1050.57515436
Enthalpy 0K = -1049.863344
Enthalpy 298K = -1049.821171
Free energy 298K = -1049.936074
Low freq. = 21.6826 cm-1
Second freq. = 31.8123 cm-1

Ir	0.10847	-0.54995	0.19648
H	0.30345	-2.17472	-0.62755
H	0.33069	-2.31310	0.24085
P	-2.22143	-0.80428	-0.04960
P	2.42252	-0.22019	-0.09747
N	-0.02307	0.31988	-1.86022
H	-0.15363	1.34352	-1.74243
C	-2.48454	-0.01567	-1.74904
H	-3.36235	-0.43567	-2.26749
H	-2.68261	1.05635	-1.56907
C	-1.22442	-0.15468	-2.61296
H	-1.04685	-1.20568	-2.89717
H	-1.33756	0.44239	-3.53843
C	1.24497	0.16448	-2.63695
H	1.18662	0.77263	-3.56026
H	1.33150	-0.89672	-2.92532
C	2.44648	0.61389	-1.79511
H	2.37298	1.70070	-1.60966
H	3.39138	0.42949	-2.33275
C	-2.92183	-2.57378	-0.18637
H	-4.01962	-2.43583	-0.15712
C	-2.56080	-3.26321	-1.51515
H	-3.00962	-4.27156	-1.53736

H	-1.46970	-3.39337	-1.62406
H	-2.93708	-2.72032	-2.39718
C	-2.49552	-3.44688	1.00999
H	-2.98169	-4.43554	0.93820
H	-2.76936	-3.00999	1.98205
H	-1.40482	-3.61115	1.01506
C	-3.46946	0.13157	1.03706
H	-3.03878	1.15093	1.02439
C	-4.89280	0.19133	0.45193
H	-5.52935	0.83007	1.08958
H	-5.36907	-0.80377	0.42050
H	-4.91757	0.61539	-0.56463
C	-3.48357	-0.37759	2.49140
H	-3.98738	0.35528	3.14591
H	-2.46874	-0.56091	2.88842
H	-4.04242	-1.32402	2.58329
C	3.41807	0.99242	0.97540
H	2.74959	1.87430	0.97224
C	3.57495	0.50123	2.42761
H	3.88607	1.33599	3.08008
H	4.35386	-0.27524	2.51048
H	2.64218	0.06882	2.83287
C	4.77461	1.40091	0.37181
H	5.24237	2.17745	1.00265
H	4.68018	1.81769	-0.64372
H	5.48121	0.55411	0.33144
C	3.53707	-1.76042	-0.26185
H	4.56639	-1.35356	-0.25691
C	3.32681	-2.51606	-1.58687
H	4.01163	-3.38100	-1.62689
H	3.53497	-1.89517	-2.47313
H	2.30002	-2.91335	-1.67152
C	3.37049	-2.71331	0.93789
H	4.08442	-3.55055	0.84678
H	2.35507	-3.14302	0.96837
H	3.55263	-2.22348	1.90622
H	0.76818	2.29287	0.54788
H	0.51271	3.58257	2.81088
H	0.73859	1.15622	2.83745
B	-0.14627	1.53734	0.88668
C	-0.30522	2.93430	3.15596
N	-0.14017	1.59059	2.52291
H	0.17623	-0.93795	1.76181
H	-0.28866	2.86684	4.25518
H	-0.87768	0.95132	2.85028
H	-1.26344	3.35815	2.82366
H	-1.22389	2.04603	0.56388
H	-0.55757	4.45118	-3.37079
H	-1.88476	5.96206	-1.72575
H	0.25621	5.08289	-0.99735
B	-0.46991	3.64195	-2.46841
C	-1.80647	5.25286	-0.88989
N	-0.55679	4.45674	-1.06288
H	0.62435	3.08362	-2.45824
H	-1.81153	5.79632	0.06857
H	-0.46314	3.76909	-0.29218
H	-2.66066	4.56284	-0.93607
H	-1.41414	2.85621	-2.45786

TS(B-C)

BP86 energy = -1050.55948768
Enthalpy 0K = -1049.850826
Enthalpy 298K = -1049.807329
Free energy 298K = -1049.925790
Low freq. = -124.7296 cm-1
Second freq. = 18.4709 cm-1

Ir	0.10664	-0.49039	0.20001
H	0.56174	-3.85452	0.52487
H	0.46503	-3.12807	0.72021
P	-2.20410	-0.82503	-0.08643
P	2.42098	-0.20863	-0.12598
N	-0.02201	0.34355	-1.88452
H	-0.15818	1.36808	-1.78146
C	-2.48198	-0.04932	-1.79255
H	-3.33658	-0.51113	-2.31564
H	-2.72734	1.01489	-1.62436
C	-1.21172	-0.14780	-2.64554
H	-1.00923	-1.19141	-2.93958
H	-1.33214	0.44961	-3.57010
C	1.24318	0.18276	-2.66563
H	1.18678	0.78973	-3.59010
H	1.31814	-0.87766	-2.95979
C	2.45672	0.61278	-1.83278
H	2.41726	1.70403	-1.66334
H	3.39364	0.39150	-2.37178
C	-2.88194	-2.59617	-0.28563
H	-3.95015	-2.44592	-0.53498
C	-2.21479	-3.33486	-1.46212
H	-2.62582	-4.35717	-1.53297
H	-1.12493	-3.42880	-1.32070
H	-2.40179	-2.84440	-2.43101
C	-2.78157	-3.41501	1.01463
H	-3.16334	-4.43567	0.83613
H	-3.37510	-2.98307	1.83401
H	-1.73895	-3.50293	1.35887
C	-3.49330	0.09688	0.97283
H	-3.12587	1.13640	0.87545
C	-4.92881	0.03068	0.42010
H	-5.58850	0.68707	1.01507
H	-5.34609	-0.98858	0.48628
H	-4.99681	0.36284	-0.62843
C	-3.44848	-0.29208	2.46484
H	-3.87509	0.51583	3.08501
H	-2.42445	-0.50967	2.81918
H	-4.04644	-1.19586	2.66618
C	3.44450	1.01591	0.91570
H	2.83413	1.93238	0.80523
C	3.49289	0.65032	2.41309
H	3.71544	1.54615	3.01894
H	4.28645	-0.08406	2.62699
H	2.54758	0.20489	2.77176
C	4.85224	1.29301	0.35773

```
H   5.32808  2.10677  0.93330
H   4.83661  1.60608 -0.69883
H   5.50867  0.41063  0.44622
C   3.53215 -1.74272 -0.34597
H   4.51730 -1.32027 -0.62308
C   3.05231 -2.63622 -1.50601
H   3.71553 -3.51498 -1.59003
H   3.07977 -2.11742 -2.47777
H   2.02925 -3.01236 -1.33776
C   3.68295 -2.54917  0.95701
H   4.30732 -3.44013  0.76810
H   2.70825 -2.89681  1.33448
H   4.17119 -1.97183  1.75633
H   0.78582  2.25383  0.63779
H   0.48714  3.43920  2.97012
H   0.68629  1.00653  2.86661
B  -0.15383  1.51961  0.93308
C  -0.34513  2.78308  3.26134
N  -0.18226  1.47003  2.56230
H   0.20044 -1.08549  1.69783
H  -0.35090  2.65242  4.35431
H  -0.93462  0.82184  2.83754
H  -1.29252  3.23614  2.93689
H  -1.23284  2.00795  0.59630
H  -0.55405  4.47993 -3.33444
H  -1.91250  6.00498 -1.73425
H   0.20611  5.12980 -0.94686
B  -0.48294  3.67615 -2.42613
C  -1.85944  5.30039 -0.89267
N  -0.60505  4.50291 -1.02780
H   0.61375  3.12473 -2.38484
H  -1.89066  5.84865  0.06245
H  -0.53063  3.82379 -0.25077
H  -2.71238  4.61069 -0.96068
H  -1.42384  2.88685 -2.42827
```

C

```
BP86 energy = -1049.38808178
Enthalpy 0K = -1048.692632
Enthalpy 298K = -1048.650655
Free energy 298K = -1048.767747
Low freq. = 15.3007 cm-1
Second freq. = 22.4850 cm-1

Ir -0.10594 -0.43543 -0.20693
P   2.17344 -0.86662  0.07826
P  -2.40564 -0.18080  0.12483
N   0.03535  0.36252  1.88911
H   0.19541  1.38589  1.80802
C   2.48537 -0.12726  1.79280
H   3.32151 -0.62105  2.31603
H   2.77139  0.92740  1.62678
C   1.20990 -0.17872  2.64357
H   0.97011 -1.21619  2.93143
H   1.35418  0.40614  3.57264
C  -1.23647  0.20850  2.66455
H  -1.18151  0.81576  3.58877
H  -1.31854 -0.85072  2.96094
C  -2.45021  0.63788  1.83026
H  -2.40505  1.72738  1.64947
H  -3.38755  0.42838  2.37263
C   2.63951 -2.71179  0.19423
H   3.74601 -2.73916  0.19623
C   2.13411 -3.35867  1.49768
H   2.42015 -4.42491  1.51460
H   1.03219 -3.31712  1.56778
H   2.55973 -2.89310  2.40124
C   2.11082 -3.48422 -1.03074
H   2.41805 -4.54278 -0.96367
H   2.48880 -3.08696 -1.98491
H   1.00891 -3.44658 -1.07165
C   3.54442 -0.05356 -0.96224
H   3.21998  1.00531 -0.95663
C   4.94601 -0.14480 -0.33058
H   5.67036  0.41396 -0.94944
H   5.30564 -1.18693 -0.27821
H   4.98411  0.28221  0.68415
C   3.55559 -0.55519 -2.41857
H   4.15818  0.12141 -3.04946
H   2.54213 -0.62534 -2.85282
H   4.01068 -1.55690 -2.49602
C  -3.50026  0.98117 -0.91186
H  -2.87993  1.89813 -0.93922
C  -3.69084  0.47342 -2.35361
H  -4.09049  1.28085 -2.99177
H  -4.41484 -0.35768 -2.39578
H  -2.75049  0.10724 -2.80345
C  -4.85156  1.31896 -0.25523
H  -5.39411  2.05310 -0.87693
H  -4.73816  1.75962  0.74796
H  -5.50023  0.43018 -0.16882
C  -3.38997 -1.80759  0.27542
H  -4.45593 -1.50977  0.26470
C  -3.10513 -2.54659  1.59631
H  -3.69588 -3.47868  1.63106
H  -3.37645 -1.95592  2.48616
H  -2.04132 -2.83373  1.67694
C  -3.10414 -2.72639 -0.92914
H  -3.71399 -3.64365 -0.85062
H  -2.04087 -3.02005 -0.95346
H  -3.33547 -2.25258 -1.89517
H  -0.74313  2.30729 -0.67336
H  -0.47592  3.43044 -3.05711
H  -0.74863  1.00633 -2.86860
B   0.17518  1.54920 -0.98087
C   0.32335  2.73803 -3.35670
N   0.14319  1.45317 -2.61061
H  -0.21357 -1.05654 -1.69226
H   0.28517  2.57399 -4.44449
H   0.86225  0.76922 -2.88761
H   1.29599  3.16860 -3.08002
H   1.28022  2.01323 -0.69715
H   0.68065  4.48589  3.27799
```

```
H   2.06356  5.96212  1.65137
H  -0.08775  5.13911  0.89480
B   0.57932  3.67833  2.37592
C   1.98071  5.25401  0.81514
N   0.70727  4.49127  0.97071
H  -0.53155  3.15424  2.35129
H   2.01429  5.79521 -0.14389
H   0.60562  3.81006  0.19828
H   2.81567  4.54203  0.87622
H   1.50019  2.86533  2.37497
```

TS(C-D)

```
BP86 energy = -1049.36453429
Enthalpy 0K = -1048.675994
Enthalpy 298K = -1048.634488
Free energy 298K = -1048.748512
Low freq. = -879.4645 cm-1
Second freq. = 21.3212 cm-1
```

```
Ir -0.07713 -0.41374  0.19001
P  -2.38901 -0.20530 -0.17498
P   2.22139 -0.81489 -0.09572
N   0.09415  0.61381 -1.76553
H   0.23801  1.62576 -1.57198
C  -2.40199  0.82940 -1.75461
H  -3.31639  0.67437 -2.35119
H  -2.38743  1.88627 -1.43229
C  -1.15568  0.53200 -2.59352
H  -1.20284 -0.48318 -3.02072
H  -1.07899  1.25218 -3.42997
C   1.28859  0.17242 -2.56073
H   1.43044  0.86630 -3.41094
H   1.06144 -0.82595 -2.96999
C   2.55278  0.13299 -1.69664
H   2.83080  1.16049 -1.40025
H   3.39707 -0.29076 -2.26579
C  -3.30616 -1.82927 -0.57234
H  -4.38103 -1.57078 -0.51549
C  -3.00439 -2.35376 -1.98810
H  -3.56768 -3.28743 -2.16061
H  -1.93288 -2.59411 -2.10671
H  -3.29661 -1.64669 -2.78132
C  -2.98714 -2.90980  0.47976
H  -3.58745 -3.81379  0.27580
H  -3.20589 -2.58755  1.50905
H  -1.92087 -3.18957  0.43843
C  -3.56582  0.75840  0.96642
H  -2.97041  1.66318  1.19626
C  -4.87469  1.19055  0.27833
H  -5.48125  1.78589  0.98339
H  -5.48928  0.32355 -0.01996
H  -4.70388  1.81370 -0.61378
C  -3.84782  0.00124  2.27777
H  -4.34759  0.67296  2.99758
H  -2.92923 -0.38336  2.75114
H  -4.52759 -0.85131  2.11051
C   3.54458 -0.10238  1.07221
H   3.20577  0.94851  1.16641
C   3.52206 -0.75758  2.46679
H   4.10730 -0.14880  3.17829
H   3.98532 -1.75814  2.44724
H   2.50099 -0.87374  2.86915
C   4.96542 -0.10638  0.47715
H   5.65920  0.39936  1.17166
H   5.02608  0.42070 -0.48831
H   5.34609 -1.13221  0.33381
C   2.72684 -2.62109 -0.42031
H   3.83357 -2.62138 -0.41023
C   2.24844 -3.11728 -1.79766
H   2.55307 -4.16945 -1.93486
H   2.67818 -2.54294 -2.63440
H   1.14704 -3.08329 -1.87528
C   2.20477 -3.54604  0.69652
H   2.54970 -4.57866  0.51297
H   1.10177 -3.55135  0.71482
H   2.55326 -3.25168  1.69809
H   1.24632  2.08322  0.68693
H  -0.36797  2.55087  4.06853
H   1.26771  1.03058  2.94201
B   0.20335  1.66796  1.16526
C  -0.61324  1.54789  3.67449
N   0.31148  1.15616  2.59578
H  -0.25691 -1.47201  1.40044
H  -0.57640  0.82534  4.50596
H   0.01291 -0.07597  1.90135
H  -1.63743  1.57871  3.27619
H  -0.80581  2.31549  0.95614
H  -0.17316  5.37580 -0.49501
H   0.56421  4.06118  0.17825
N   0.64186  4.75479 -0.58395
H  -0.55796  3.39856 -1.97766
H   1.90728  6.07881  0.55333
B   0.53691  3.95616 -1.99759
C   1.89133  5.55570 -0.41576
H   0.61298  4.77504 -2.89150
H   1.48111  3.16933 -2.00778
H   1.94914  6.28104 -1.23912
H   2.74859  4.87212 -0.48999
```

[D·H_2B=NMeH]$^+$

```
BP86 energy = -1049.41517860
Enthalpy 0K = -1048.722581
Enthalpy 298K = -1048.680382
Free energy 298K = -1048.798464
Low freq. = 16.8711 cm-1
Second freq. = 18.8747 cm-1
```

```
Ir -0.44737 -0.56378 -0.16380
P   1.55164 -1.71806  0.28530
P  -2.39167  0.73100  0.03539
N   0.49839  1.09336  1.06754
H   0.94383  1.77578  0.42993
```

```
C   2.52177 -0.38716  1.22056
H   3.25003 -0.82615  1.92366
H   3.09544  0.17275  0.45932
C   1.57302  0.56001  1.96583
H   1.06697  0.02496  2.78596
H   2.13939  1.40583  2.39814
C  -0.53654  1.84433  1.84971
H  -0.07395  2.74545  2.29387
H  -0.86483  1.18118  2.66693
C  -1.72710  2.23805  0.96828
H  -1.40351  2.96493  0.20096
H  -2.50996  2.72456  1.57477
C   1.44493 -3.22474  1.43848
H   2.45273 -3.68013  1.40429
C   1.13707 -2.83647  2.89556
H   1.08407 -3.75008  3.51306
H   0.16020 -2.32853  2.97576
H   1.91011 -2.18778  3.33951
C   0.40759 -4.23689  0.91534
H   0.38790 -5.12147  1.57577
H   0.62687 -4.58745 -0.10506
H  -0.60020 -3.78971  0.90675
C   2.76830 -2.20335 -1.09002
H   2.89726 -1.23300 -1.60676
C   4.14117 -2.67802 -0.57925
H   4.82955 -2.80917 -1.43278
H   4.07536 -3.65397 -0.06898
H   4.61038 -1.95978  0.11307
C   2.15361 -3.20180 -2.08934
H   2.77866 -3.26105 -2.99802
H   1.12869 -2.91945 -2.38585
H   2.10788 -4.21970 -1.66745
C  -3.11715  1.51865 -1.53346
H  -2.22294  2.03844 -1.93281
C  -3.56799  0.47889 -2.57613
H  -3.74757  0.97790 -3.54433
H  -4.51338 -0.00681 -2.28246
H  -2.81264 -0.30864 -2.73153
C  -4.21352  2.56315 -1.25351
H  -4.48002  3.08250 -2.19070
H  -3.89716  3.33254 -0.52979
H  -5.13629  2.09232 -0.87377
C  -3.84338  0.06341  1.05516
H  -4.65223  0.80744  0.92496
C  -3.49975 -0.03276  2.55235
H  -4.36803 -0.43668  3.10150
H  -3.25703  0.94505  3.00018
H  -2.65206 -0.71921  2.72250
C  -4.30650 -1.30195  0.51222
H  -5.15907 -1.66851  1.11025
H  -3.49326 -2.04374  0.58086
H  -4.63215 -1.25575 -0.53841
H  -0.44766 -0.27148 -1.87529
H   1.85892  1.19301 -4.90313
H   1.25077 -0.71070 -3.43844
B   0.52746  0.61428 -1.97308
C   2.19816  1.11019 -3.85561
N   1.30916  0.24379 -3.07743
H  -0.83702 -1.16649  1.28081
H   3.23307  0.72364 -3.86037
H  -1.17907 -1.84307 -0.78313
H   2.20044  2.11476 -3.40788
H   0.55213  1.77695 -1.65028
H   2.11707  3.83764  1.95057
H   4.25240  5.28183  2.23709
H   2.45152  6.12589  0.85652
B   2.25297  3.95031  0.74084
C   4.38617  5.43700  1.15770
N   3.04129  5.35851  0.50711
H   1.18263  4.06275  0.15404
H   4.86691  6.40975  0.97036
H   3.14266  5.52364 -0.50331
H   5.00742  4.62383  0.75775
H   2.96296  3.07399  0.26131
```

[1-NMeH$_2$]$^+$

```
BP86 energy = -1023.94649551
Enthalpy 0K = -1023.264221
Enthalpy 298K = -1023.223521
Free energy 298K = -1023.337412
Low freq. = 14.2403 cm-1
Second freq. = 17.7486 cm-1

Ir  0.09217 -0.49355  0.22992
H   0.21207 -1.74113 -0.75665
H   0.19978 -1.54984  1.42035
P  -2.20153 -0.77148 -0.10938
P   2.38700 -0.28808 -0.10650
N  -0.05319  0.93475 -1.53970
H  -0.14897  1.89830 -1.17634
C  -2.51852  0.49375 -1.48645
H  -3.39023  0.22501 -2.10705
H  -2.75360  1.44902 -0.98175
C  -1.27336  0.66238 -2.36300
H  -1.07584 -0.26145 -2.93062
H  -1.42757  1.48214 -3.09320
C   1.18553  0.88891 -2.37834
H   1.16968  1.70619 -3.12745
H   1.16048 -0.06749 -2.92606
C   2.45657  0.97339 -1.52510
H   2.53836  1.97221 -1.05729
H   3.34997  0.83668 -2.15821
C  -2.79494 -2.45480 -0.77083
H  -3.89800 -2.43017 -0.68149
C  -2.42916 -2.67060 -2.25036
H  -2.78894 -3.66225 -2.57626
H  -1.33460 -2.65518 -2.39414
H  -2.88498 -1.92284 -2.92025
C  -2.23718 -3.60308  0.09276
H  -2.61948 -4.56839 -0.28355
H  -2.52110 -3.52043  1.15291
H  -1.13601 -3.62151  0.04356
C  -3.52210 -0.26871  1.17228
```

H	-3.13737	0.70704	1.53178
C	-4.92634	-0.04485	0.57917
H	-5.61479	0.29602	1.37277
H	-5.34928	-0.97744	0.16747
H	-4.93913	0.71671	-0.21684
C	-3.57334	-1.24643	2.36221
H	-4.16369	-0.80873	3.18640
H	-2.57133	-1.49458	2.74986
H	-4.06845	-2.19037	2.07844
C	3.49993	0.56716	1.18716
H	2.98588	1.54435	1.31066
C	3.48951	-0.15721	2.54645
H	3.91596	0.49499	3.32874
H	4.10583	-1.07090	2.51729
H	2.47189	-0.45162	2.85229
C	4.93351	0.85533	0.70373
H	5.46417	1.47376	1.44908
H	4.96016	1.39894	-0.25508
H	5.51428	-0.07513	0.58635
C	3.35872	-1.80371	-0.71003
H	4.41899	-1.48780	-0.72479
C	2.95242	-2.21692	-2.13623
H	3.52824	-3.10968	-2.43663
H	3.15631	-1.43209	-2.88362
H	1.88173	-2.48069	-2.18357
C	3.18318	-2.98019	0.26904
H	3.74917	-3.85507	-0.09620
H	2.12026	-3.26276	0.34833
H	3.54574	-2.74952	1.28286
H	-0.42022	1.71524	3.82974
C	-0.46098	0.84625	3.14805
H	-0.59988	1.97156	1.41620
H	0.92198	1.59495	1.83578
N	-0.02024	1.19449	1.76931
H	-1.49010	0.46561	3.11209
H	0.18119	0.04375	3.53709
H	0.30837	3.88781	0.18522
H	0.72171	6.30089	-0.74710
H	-1.00010	5.97485	0.92809
B	-0.87261	4.04512	-0.09806
C	-0.33925	6.46833	-0.97798
N	-1.15899	5.64431	-0.03358
H	-1.62870	3.53014	0.71952
H	-0.58260	7.53797	-0.88713
H	-2.15747	5.80938	-0.22130
H	-0.54101	6.12015	-2.00025
H	-1.13315	3.69612	-1.24228

1-H_3-TS(A-B)

BP86 energy = -928.787700060
Enthalpy 0K = -928.172098
Enthalpy 298K = -928.136340
Free energy 298K = -928.236481
Low freq. = -105.3412 cm-1
Second freq. = 23.5023 cm-1

Ir	0.00792	-0.32317	-0.26249
P	-2.30310	-0.29089	0.00794
P	2.32839	-0.26334	0.01445
N	0.01609	0.95545	1.56928
C	-2.49349	0.87132	1.49417
H	-2.63007	1.88354	1.07543
H	-3.38853	0.63421	2.09523
C	-1.23358	0.83434	2.37248
H	-1.27372	1.64969	3.12264
H	-1.17908	-0.12294	2.92000
C	3.35724	0.72228	-1.23800
H	2.90939	1.72867	-1.10529
C	-3.36519	0.58645	-1.30275
H	-2.90670	1.59526	-1.28651
C	-3.21939	-1.88388	0.48248
H	-4.29173	-1.61232	0.52333
C	2.50938	0.78988	1.57240
H	3.38315	0.48755	2.17484
H	2.65887	1.82870	1.22077
C	3.13517	0.27428	-2.69328
H	3.63746	-0.68419	-2.91114
H	3.55653	1.02732	-3.38302
H	2.06400	0.15546	-2.92468
C	3.25413	-1.88062	0.36710
H	4.31928	-1.59179	0.45424
C	1.22923	0.74720	2.41494
H	1.11261	-0.22897	2.91894
H	1.27597	1.54218	3.18479
C	-3.16894	0.00485	-2.71463
H	-2.10173	-0.14005	-2.94830
H	-3.60014	0.69042	-3.46581
H	-3.67899	-0.96675	-2.83032
C	4.85119	0.80796	-0.87808
H	5.01475	1.15416	0.15608
H	5.35432	1.52883	-1.54676
H	5.36044	-0.16391	-1.00459
C	3.08350	-2.87694	-0.79407
H	2.01537	-3.10001	-0.95711
H	3.60398	-3.82324	-0.55990
H	3.49616	-2.49449	-1.74123
C	-2.78525	-2.38947	1.86988
H	-1.70175	-2.59891	1.89035
H	-3.31534	-3.32975	2.10507
H	-3.01645	-1.67157	2.67446
C	-3.00054	-2.97030	-0.58683
H	-3.39319	-2.67765	-1.57349
H	-3.51123	-3.90283	-0.28606
H	-1.92477	-3.18497	-0.70297
C	-4.85274	0.71523	-0.92956
H	-5.36420	-0.26266	-0.95846
H	-5.36776	1.37072	-1.65443
H	-5.00253	1.15107	0.07263
C	2.80302	-2.50625	1.69852
H	2.98577	-1.84319	2.56047
H	3.35990	-3.44355	1.87753
H	1.72721	-2.75135	1.67237
H	0.02035	-1.33465	-1.51110

```
H   0.02843 -1.66689  0.62848
H   0.01590  0.82485 -1.58563
H  -0.04292  1.41268 -0.89270
H   1.26904  5.17995  0.46273
H  -1.50856  4.00384  1.15166
H   0.06286  1.91707  1.13465
B   1.20644  3.95158  0.57306
C  -1.27634  4.09335  0.07312
N  -0.03951  3.35359 -0.21433
H   1.08998  3.64612  1.77659
H  -2.14887  3.70852 -0.49744
H   0.15849  3.48758 -1.21461
H  -1.19055  5.18286 -0.13136
H   2.23681  3.44377  0.09805
```

1-H_3-B

```
BP86 energy = -928.798986045
Enthalpy 0K = -928.184221
Enthalpy 298K = -928.147502
Free energy 298K = -928.250591
Low freq. = 25.4477 cm-1
Second freq. = 30.0714 cm-1

Ir  0.06213 -0.40810  0.24328
P   2.36251 -0.14958  0.01452
P  -2.25690 -0.43463 -0.04052
N  -0.02547  1.21613 -1.28706
C   2.46770  1.23257 -1.27154
H   2.53902  2.17722 -0.70088
H   3.37316  1.15457 -1.89835
C   1.18530  1.21681 -2.11980
H   1.19167  2.10786 -2.79116
H   1.21292  0.32804 -2.79514
C  -3.32707  0.19914  1.39132
H  -2.88600  1.20599  1.53378
C   3.34257  0.58863  1.47097
H   2.78677  1.53554  1.62496
C   3.41597 -1.57301 -0.67418
H   4.46130 -1.20876 -0.65343
C  -2.49358  0.90552 -1.34596
H  -3.36404  0.70301 -1.99388
H  -2.68672  1.83730 -0.78221
C  -3.14117 -0.59352  2.69756
H  -3.65582 -1.56950  2.66421
H  -3.57272 -0.02549  3.54094
H  -2.07715 -0.77759  2.91860
C  -3.09313 -1.99578 -0.72454
H  -4.17609 -1.76497 -0.73295
C  -1.19836  1.04478 -2.16001
H  -1.07877  0.15498 -2.82384
H  -1.29911  1.92301 -2.83985
C   3.22228 -0.23622  2.76519
H   2.18069 -0.53862  2.96088
H   3.57850  0.35793  3.62570
H   3.83930 -1.14986  2.72282
C  -4.81314  0.36046  1.02403
H  -4.95721  0.95377  0.10583
H  -5.34381  0.88437  1.83898
H  -5.31058 -0.61626  0.88670
C  -2.83207 -3.20769  0.18861
H  -1.74871 -3.39507  0.27970
H  -3.30618 -4.11024 -0.23823
H  -3.23446 -3.06659  1.20410
C   3.03669 -1.90005 -2.12963
H   1.97817 -2.20345 -2.20067
H   3.65611 -2.73956 -2.49364
H   3.19543 -1.04903 -2.81221
C   3.28571 -2.82519  0.21269
H   3.64976 -2.65853  1.23879
H   3.87250 -3.65543 -0.22041
H   2.23102 -3.14188  0.27788
C   4.80680  0.92747  1.13821
H   5.41212  0.01401  1.00503
H   5.26243  1.49858  1.96713
H   4.90305  1.53653  0.22349
C  -2.64080 -2.29315 -2.16493
H  -2.88544 -1.47613 -2.86345
H  -3.14224 -3.20741 -2.53093
H  -1.55131 -2.46416 -2.20663
H   0.12881 -1.77753  1.11211
H   0.13833 -1.42452 -1.01147
H  -0.00055  0.11245  1.87759
H  -0.10311  0.90581  1.39413
H  -2.49783  3.52656  0.50552
H  -1.26953  4.59532 -1.53856
H  -0.18039  2.67767 -0.45841
B  -1.45338  3.72270  1.12470
C  -0.32525  4.72314 -0.98806
N  -0.24578  3.65875  0.04468
H  -1.23138  2.84297  1.95520
H   0.51992  4.67222 -1.69664
H   0.63642  3.77129  0.56043
H  -0.34602  5.71053 -0.50082
H  -1.43523  4.83877  1.63008
```

1-H_3-TS(B-C)

```
BP86 energy = -928.779253178
Enthalpy 0K = -928.167168
Enthalpy 298K = -928.129483
Free energy 298K = -928.233880
Low freq. = -350.8243 cm-1
Second freq. = 31.7273 cm-1

Ir  0.04616 -0.40917  0.24420
P   2.34114 -0.19779  0.02117
P  -2.26266 -0.41481 -0.04814
N  -0.01495  1.17976 -1.19534
C   2.48145  1.25785 -1.18490
H   2.59359  2.16278 -0.55866
H   3.37810  1.18547 -1.82515
C   1.19403  1.34268 -2.02150
H   1.17559  2.32488 -2.55013
```

```
H   1.22930  0.57161 -2.82667
C  -3.38131  0.11413  1.39435
H  -3.02252  1.15113  1.55161
C   3.38807  0.41175  1.49504
H   2.92969  1.40597  1.67082
C   3.32686 -1.60239 -0.79268
H   4.36832 -1.23479 -0.87016
C  -2.50480  0.99553 -1.27950
H  -3.35854  0.80849 -1.95418
H  -2.73126  1.89550 -0.67923
C  -3.12157 -0.67803  2.68783
H  -3.53556 -1.69978  2.63713
H  -3.60830 -0.16998  3.53970
H  -2.04335 -0.75988  2.90010
C  -3.06592 -1.93339 -0.85367
H  -4.13345 -1.66912 -0.98218
C  -1.20530  1.21646 -2.06857
H  -1.11743  0.45442 -2.87833
H  -1.27302  2.20247 -2.58451
C   3.17222 -0.42694  2.76759
H   2.10123 -0.61609  2.94556
H   3.58794  0.10233  3.64385
H   3.68216 -1.40319  2.70433
C  -4.87537  0.16410  1.02838
H  -5.06477  0.75293  0.11510
H  -5.44679  0.63641  1.84747
H  -5.29353 -0.84745  0.88070
C  -2.95259 -3.17608  0.04764
H  -1.89698 -3.39253  0.28551
H  -3.37655 -4.05687 -0.46828
H  -3.49344 -3.05547  0.99967
C   2.79597 -1.88762 -2.20962
H   1.72841 -2.16552 -2.18080
H   3.35688 -2.72804 -2.65722
H   2.90271 -1.02046 -2.88162
C   3.29599 -2.87573  0.07221
H   3.78291 -2.73336  1.05007
H   3.82334 -3.69664 -0.44671
H   2.25706 -3.19747  0.25786
C   4.87927  0.61070  1.17113
H   5.39000 -0.35575  1.01694
H   5.38852  1.11494  2.01229
H   5.03776  1.22746  0.26998
C  -2.45215 -2.20051 -2.24004
H  -2.59453 -1.35485 -2.93261
H  -2.92422 -3.09011 -2.69533
H  -1.36841 -2.39305 -2.15760
H   0.06090 -1.36732  1.56044
H   0.12888 -1.87973 -0.26358
H  -0.02537  0.81675  1.97102
H  -0.24617  1.55659  2.08751
H  -2.43641  4.09036 -0.30854
H  -0.33123  4.79207 -1.63950
H  -0.16420  2.76382 -0.28007
B  -1.72698  4.06777  0.69161
C   0.32295  4.75744 -0.75598
N  -0.21780  3.73588  0.17843
H  -2.03370  3.16601  1.46818
H   1.35531  4.52395 -1.06924
H   0.39162  3.68938  1.00493
H   0.29285  5.74356 -0.26772
H  -1.67789  5.15472  1.25221
```

1-H$_3$-C

```
BP86 energy = -927.613223386
Enthalpy 0K = -927.013411
Enthalpy 298K = -926.976674
Free energy 298K = -927.081089
Low freq. = 14.9846 cm-1
Second freq. = 28.7881 cm-1
```

```
Ir -0.03225 -0.14843 -0.20843
P  -2.32683 -0.19173  0.06377
P   2.26973 -0.32869  0.01281
N  -0.00415 -0.05357  1.80274
C  -2.48988  0.25845  1.89260
H  -2.61244  1.35616  1.91038
H  -3.38648 -0.18801  2.35745
C  -1.20686 -0.13152  2.64854
H  -1.08761  0.54139  3.52623
H  -1.31360 -1.15743  3.06785
C   3.46389  0.84824 -0.88390
H   3.18471  1.81593 -0.42100
C  -3.48076  1.08122 -0.76368
H  -3.13423  2.01748 -0.28370
C  -3.20179 -1.87173 -0.10138
H  -4.24991 -1.70500  0.21360
C   2.49347  0.15770  1.82005
H   3.38475 -0.31070  2.27325
H   2.64585  1.25170  1.80063
C   3.18820  0.94368 -2.39526
H   3.52963  0.04429 -2.93598
H   3.73122  1.80631 -2.82250
H   2.11182  1.06810 -2.59989
C   3.01803 -2.06940 -0.14126
H   4.08664 -1.96187  0.12747
C   1.21867 -0.16460  2.61848
H   1.28799 -1.18471  3.05943
H   1.15122  0.53651  3.47872
C  -3.22819  1.21204 -2.27628
H  -2.15097  1.29874 -2.49376
H  -3.74388  2.10623 -2.67139
H  -3.61405  0.34132 -2.83293
C   4.94844  0.58098 -0.57840
H   5.15163  0.53001  0.50470
H   5.56957  1.39658 -0.99043
H   5.29818 -0.35953 -1.03932
C   2.90371 -2.60334 -1.58010
H   1.84915 -2.61811 -1.90568
H   3.29505 -3.63565 -1.63317
H   3.47220 -1.99499 -2.30194
C  -2.55297 -2.90029  0.84304
H  -1.47785 -3.00840  0.61837
```

```
H  -3.03504 -3.88651  0.71463
H  -2.65251 -2.61673  1.90376
C  -3.17613 -2.37235 -1.55662
H  -3.73909 -1.71535 -2.23928
H  -3.62644 -3.37972 -1.61818
H  -2.13817 -2.43661 -1.92641
C  -4.97179  0.88937 -0.43411
H  -5.37867 -0.01699 -0.91504
H  -5.55833  1.74703 -0.81053
H  -5.15771  0.81247  0.65086
C   2.34728 -3.03114  0.85628
H   2.51173 -2.72889  1.90356
H   2.75920 -4.04953  0.73397
H   1.25850 -3.07541  0.68045
H  -0.02665  0.63847 -1.61751
H  -0.06427 -1.10627 -1.49106
H   1.08800  3.63075  2.17486
H  -1.25042  3.88474  1.01914
H   0.35112  2.34746  0.03481
B   1.55920  3.92311  1.08417
C  -0.81857  4.06986  0.02476
N   0.50092  3.38711 -0.03603
H   2.62282  3.35377  0.85876
H  -1.50129  3.70185 -0.75887
H   0.90363  3.52847 -0.97161
H  -0.65794  5.15222 -0.08678
H   1.65035  5.13074  0.91016
```

1-H$_3$-TS(C-D)

```
BP86 energy = -927.606482039
Enthalpy 0K = -927.008057
Enthalpy 298K = -926.972220
Free energy 298K = -927.073262
Low freq. = -150.9419 cm-1
Second freq. = 29.9202 cm-1

Ir  0.03122 -0.30660  0.23478
P   2.31953 -0.19955 -0.01928
P  -2.27236 -0.31046 -0.01389
N   0.00440  0.94680 -1.45603
C   2.50374  0.96823 -1.50610
H   2.70006  1.97207 -1.08579
H   3.37128  0.70399 -2.13573
C   1.19867  0.97260 -2.32244
H   1.18517  1.87876 -2.97403
H   1.19365  0.10323 -3.01808
C  -3.38701  0.40447  1.34902
H  -3.01945  1.44837  1.37359
C   3.39857  0.66900  1.29326
H   3.00660  1.70391  1.23114
C   3.26430 -1.76422 -0.53772
H   4.30164 -1.43994 -0.74967
C  -2.49389  0.94652 -1.41338
H  -3.38920  0.73942 -2.02549
H  -2.64237  1.91557 -0.90351
C  -3.10758 -0.20976  2.73195
H  -3.49221 -1.24095  2.81728
H  -3.60668  0.39229  3.51256
H  -2.02679 -0.22556  2.94446
C  -3.11687 -1.89309 -0.63950
H  -4.17780 -1.62292 -0.80525
C  -1.22225  0.99162 -2.27809
H  -1.23096  0.14921 -3.00616
H  -1.23813  1.92152 -2.89463
C   3.11224  0.16200  2.71773
H   2.02863  0.13651  2.91501
H   3.58981  0.82937  3.45767
H   3.51426 -0.85225  2.88219
C  -4.88448  0.40098  0.99527
H  -5.08816  0.85911  0.01196
H  -5.45076  0.97677  1.74960
H  -5.30154 -0.62180  0.98831
C  -3.03678 -3.01853  0.40765
H  -1.98649 -3.23660  0.66714
H  -3.48921 -3.94300  0.00450
H  -3.56826 -2.76576  1.33890
C   2.65035 -2.35075 -1.82219
H   1.58689 -2.59959 -1.66391
H   3.18555 -3.27450 -2.10861
H   2.71181 -1.65252 -2.67357
C   3.27620 -2.80926  0.59222
H   3.81683 -2.45749  1.48567
H   3.77104 -3.73490  0.24592
H   2.24743 -3.06537  0.89844
C   4.90141  0.69131  0.96358
H   5.34681 -0.31558  1.04364
H   5.43913  1.34065  1.67813
H   5.10711  1.07398 -0.05123
C  -2.50107 -2.34383 -1.97645
H  -2.64787 -1.60023 -2.77710
H  -2.96913 -3.28859 -2.30879
H  -1.41631 -2.51546 -1.86529
H   0.03564 -0.68741  1.80086
H   0.07421 -1.85937  0.45630
H  -1.90496  3.31170  0.98761
H  -1.40498  4.49145 -1.18300
H   0.04010  2.55340 -0.68051
B  -0.71112  3.20980  1.26033
C  -0.35067  4.64412 -0.90981
N   0.09698  3.47257 -0.11683
H  -0.41340  2.05225  1.58756
H   0.25357  4.77600 -1.82414
H   1.09060  3.58819  0.11976
H  -0.28703  5.55141 -0.28904
H  -0.34102  4.03571  2.08293
```

1-H$_3$-D

```
BP86 energy = -927.633695776
Enthalpy 0K = -927.034642
Enthalpy 298K = -926.999094
Free energy 298K = -927.098826
Low freq. = 25.9780 cm-1
```

Second freq. = 35.9382 cm-1

```
Ir -0.03956 -0.23886 -0.24683
P  -2.32912 -0.12603  0.02152
P   2.25323 -0.32504 -0.00635
N   0.01831  0.79522  1.72990
C  -2.48115  0.81117  1.67037
H  -2.58248  1.87954  1.40783
H  -3.38558  0.52357  2.23457
C  -1.22145  0.59617  2.52641
H  -1.23617  1.28857  3.39634
H  -1.20378 -0.43404  2.92436
C   3.34991  0.69340 -1.17698
H   2.94146  1.70719 -0.99772
C  -3.35795  0.97025 -1.14081
H  -2.86966  1.95034 -0.97399
C  -3.33518 -1.71815  0.28512
H  -4.38885 -1.39821  0.39932
C   2.50857  0.57950  1.64782
H   3.38562  0.20004  2.20069
H   2.70997  1.63559  1.39418
C   3.08352  0.36399 -2.65645
H   3.50946 -0.61234 -2.94577
H   3.55228  1.13047 -3.29950
H   2.00230  0.34830 -2.86675
C   3.12169 -2.00074  0.22537
H   4.20130 -1.77495  0.32165
C   1.24172  0.47962  2.51373
H   1.13094 -0.54631  2.90819
H   1.32871  1.16357  3.38582
C  -3.13578  0.61352 -2.62122
H  -2.06134  0.51552 -2.84374
H  -3.55326  1.40963 -3.26361
H  -3.63716 -0.32972 -2.89919
C   4.84831  0.67814 -0.82967
H   5.04235  0.92757  0.22797
H   5.38772  1.41883 -1.44754
H   5.30404 -0.30640 -1.03569
C   2.88776 -2.89837 -1.00379
H   1.80693 -3.04156 -1.17302
H   3.35112 -3.88907 -0.84353
H   3.31847 -2.47255 -1.92398
C  -2.89853 -2.45096  1.56591
H  -1.82405 -2.70069  1.52307
H  -3.46457 -3.39446  1.66935
H  -3.08118 -1.85633  2.47708
C  -3.20397 -2.64101 -0.94055
H  -3.61733 -2.18679 -1.85517
H  -3.74540 -3.58804 -0.76208
H  -2.14292 -2.87589 -1.13119
C  -4.84845  1.07466 -0.77529
H  -5.38263  0.12779 -0.96891
H  -5.33587  1.85182 -1.39164
H  -5.00928  1.34467  0.28294
C   2.64584 -2.70421  1.50870
H   2.89107 -2.13372  2.42060
H   3.13286 -3.69229  1.59710
H   1.55381 -2.86360  1.48138
H  -0.07720 -0.91499 -1.70122
H  -0.10149 -1.75535  0.30769
H   0.04003  1.35393 -1.05053
H   2.09783  3.71399  0.67189
H   0.05614  1.82168  1.39573
B   0.05193  2.73563 -0.98012
C   1.12504  4.21606  0.81369
N   0.04070  3.29262  0.44605
H  -0.95998  2.97654 -1.62048
H   1.05478  4.51985  1.87594
H  -0.85399  3.74973  0.64473
H   1.15392  5.13940  0.19768
H   1.09378  2.95648 -1.57746
```

1-H_3-TS(D-1-H_3)

BP86 energy = -927.624525237
Enthalpy 0K = -927.026503
Enthalpy 298K = -926.990954
Free energy 298K = -927.091274
Low freq. = -33.6971 cm-1
Second freq. = 27.1802 cm-1

```
Ir -0.05747 -0.36349 -0.22066
P  -2.32654 -0.12908  0.01158
P   2.22579 -0.40562 -0.02601
N   0.04004  0.95315  1.61015
C  -2.45428  1.01455  1.53415
H  -2.52290  2.04765  1.14566
H  -3.36693  0.82602  2.12615
C  -1.20640  0.86183  2.41822
H  -1.20748  1.62852  3.22419
H  -1.19969 -0.13172  2.89751
C   3.29125  0.49935 -1.32074
H   2.89099  1.52725 -1.21672
C  -3.31046  0.87043 -1.27755
H  -2.79886  1.84958 -1.19765
C  -3.43232 -1.61456  0.46233
H  -4.46746 -1.22691  0.52433
C   2.52023  0.68525  1.51175
H   3.40205  0.36202  2.09198
H   2.72730  1.70593  1.14192
C   2.96922  0.03424 -2.75159
H   3.38103 -0.96849 -2.96012
H   3.41489  0.73061 -3.48520
H   1.87934  0.00346 -2.91117
C   3.15448 -2.02066  0.37342
H   4.23015 -1.76212  0.41586
C   1.27025  0.68927  2.40577
H   1.13565 -0.30032  2.87468
H   1.37937  1.44032  3.21895
C  -3.06898  0.35352 -2.70664
H  -1.99243  0.20068 -2.88518
H  -3.44920  1.08541 -3.44251
H  -3.59230 -0.60041 -2.89316
C   4.80203  0.51187 -1.03198
```

H	5.03704	0.85013	-0.00751
H	5.31934	1.19340	-1.73196
H	5.24870	-0.48861	-1.16847
C	2.90898	-3.05297	-0.74252
H	1.82621	-3.22849	-0.86398
H	3.39334	-4.01331	-0.48695
H	3.30958	-2.72498	-1.71544
C	-3.03737	-2.20918	1.82484
H	-1.97202	-2.49975	1.81948
H	-3.64211	-3.11096	2.03167
H	-3.20378	-1.50456	2.65820
C	-3.34125	-2.68478	-0.64092
H	-3.72130	-2.32338	-1.61023
H	-3.93351	-3.57472	-0.35913
H	-2.29144	-2.99392	-0.78287
C	-4.80487	1.05357	-0.96252
H	-5.36258	0.10747	-1.07676
H	-5.25622	1.78011	-1.66284
H	-4.98132	1.42857	0.06093
C	2.72031	-2.58694	1.73627
H	2.98471	-1.92027	2.57541
H	3.21932	-3.55642	1.91725
H	1.62842	-2.75087	1.75085
H	-0.12241	-1.24731	-1.54896
H	-0.13783	-1.80442	0.63213
H	0.02263	0.99867	-1.22371
H	2.25569	4.17434	0.24062
H	0.10154	1.90234	1.19996
B	0.06789	3.42069	-1.08712
C	1.39224	4.44782	0.86625
N	0.17456	3.94757	0.22759
H	-1.03182	3.29061	-1.55712
H	1.54538	4.02279	1.87669
H	-0.67930	4.21192	0.72087
H	1.38117	5.54987	0.96626
H	1.07568	3.29947	-1.73611

6. References

(S1) Martínez-Martínez, A. J.; Weller, A. S. Solvent-Free Anhydrous Li^+, Na^+ and K^+ Salts of $[B(3,5-(CF_3)_2C_6H_3)_4]^-$, $[BAr^F_4]^-$. Improved Synthesis and Solid-State Structures. *Dalton. Trans.* **2019**, *48*, 3551–3554.

(S2) Marigo, M.; Marsich, N.; Farnetti, E. Polymerization of Phenylacetylene Catalyzed by Organoiridium Compounds. *J. Mol. Catal. A Chem.* **2002**, *187*, 169–177.

(S3) Crisenza, G. E. M.; McCreanor, N. G.; Bower, J. F. Branch-Selective, Iridium-Catalyzed Hydroarylation of Monosubstituted Alkenes via a Cooperative Destabilization Strategy. *J. Am. Chem. Soc.* **2014**, *136*, 10258–10261.

(S4) Choi, Y. K.; Heo, J. H.; Hong, K. H.; Im, S. H. Dual-Site Mixed Layer-Structured $FAxCs_3$- $XSb_2I_6Cl_3$Pb-Free Metal Halide Perovskite Solar Cells. *RSC Adv.* **2020**, *10*, 17724–17730.

(S5) Jaska, C. A.; Temple, K.; Lough, A. J.; Manners, I. Transition Metal-Catalyzed Formation of Boron-Nitrogen Bonds: Catalytic Dehydrocoupling of Amine-Borane Adducts to Form Aminoboranes and Borazines. *J. Am. Chem. Soc.* **2003**, *125*, 9424–9434.

(S6) Werkmeister, S.; Junge, K.; Wendt, B.; Alberico, E.; Jiao, H.; Baumann, W.; Junge, H.; Gallou, F.; Beller, M. Hydrogenation of Esters to Alcohols with a Well-Defined Iron Complex.Pdf. *Angew. Chemie - Int. Ed.* **2014**, *53*, 8722–8726.

(S7) Colebatch, A. and Weller, A. S., Amine-Borane Dehydropolymerisation: Challenges and Opportunities. *Chem. Eur. J.*, **2019**, *25*, 1379-1390.

(S8) Staubitz, A., Sloan, M. E., Robertson, A. P. M., Friedrich, A., Schneider, S., Gates, P. J., Schmedt auf der Guenne, J. and Manners, I., *J. Am. Chem. Soc.* 2010, 132, 13332– 13345.

(S9) Anke, F.; Boye, S.; Spannenberg, A.; Lederer, A.; Heller, D. and Beweries, T., Chem. Eur. J., 2020, 26, 7889-7899

(S10) Clarke, Z. E.; Maragh, P. T.; Dasgupta, T. P.; Gusev, D. G.; Lough, A. J.; Abdur-Rashid, K. A Family of Active Iridium Catalysts for Trasfer Hydrogenation of Ketones. *Organometallics*, **2006**, *25*, 4113-4117.

(S11) Brodie, C. N.; Boyd, T. M.; Sotorrios, L.; Ryan, D. E.; Magee, E.; Huband, S.; Town, J. S.; Lloyd-Jones, G. C.; Haddleton, D. M.; Macgregor, S. A.; Weller, A. S. Controlled Synthesis of Well-Defined Polyaminoboranes on Scale Using a Robust and Efficient Catalyst. *J. Am. Chem. Soc.*, **2021**, *143*, 21010-21023.

(S12) Roselló-Merino, M.; López-Serrano, J.; Conejero, S. Dehydrocoupling Reactions of Dimethylamine-Borane by Pt(II) Complexes: A New Mechanism Involving DEprotonation of Boronium Cations. J. Am. Chem. Soc., 2013, 135, 10910-10913.

(S13) Burés, J. Variable Time Normalization Analysis: General Graphical Eludication of Reaction Orders from Concentration Profiles. *Angew. Chemie - Ind. Ed.* **2016**, *55*, 16084-16087.

(S14) Cope, A. C.; Overberger, G. C. Cyclic Polyolefins. I. Synthesis of Cyclooctatetraene from Psuedopelletierine, *J. Am. Chem. Soc.*, **1948**, *70*, 1433-1437.

(S15) Cosier, B. J.; Glazer, A. M. A Nitrogen-Gas-Stream Cryostat for General X-Ray Diffraction Studies. *J.*

Appl. Crystallogr. **1986**, *19*, 105–107.

(S16) Oxford Diffraction Ltd. **2011**.

(S17) Sheldrick, G. M. A Short History of SHELX. *Acta Crystallogr. Sect. A Found. Crystallogr.* **2008**, *64*, 112–122.

(S18) Sheldrick, G. M. SHELXT - Integrated Space-Group and Crystal-Structure Determination. *Acta Crystallogr. Sect. A Found. Crystallogr.* **2015**, *71*, 3–8.

(S19) Dolomanov, O. V.; Bourhis, L. J.; Gildea, R. J.; Howard, J. A. K.; Puschmann, H. OLEX2: A Complete Structure Solution, Refinement and Analysis Program. *J. Appl. Crystallogr.* **2009**, *42*, 339–341.

(S20) Bondi, A. Van Der Waals Volumes and Radii. *J. Phys. Chem.* **1964**, *68*, 441–451.

(S21) Frisch, M. J.; Trucks, G. W.; Schlegel, H. B.; Scuseria, G. E.; Robb, M. A.; Cheeseman, J. R.; Scalmani, G.; Barone, V.; Petersson, G. A.; Nakatsuji, H.; Li, X.; Caricato, M.; Marenich, A. V.; Bloino, J.; Janesko, B. G.; Gomperts, R.; Mennucci, B.; Hratchian, H. P.; Ortiz, J. V.; Izmaylov, A. F.; Sonnenberg, J. L.; Williams-Young, D.; F. Ding, F. L.; F. Egidi, J. G.; B. Peng, A. P.; Henderson, T.; Ranasinghe, D.; Zakrzewski, V. G.; Gao, J.; Rega, N.; Zheng, G.; Liang, W.; Hada, M.; Ehara, M.; Toyota, K.; Fukuda, R.; Hasegawa, J.; Ishida, M.; Nakajima, T.; Honda, Y.; Kitao, O.; Nakai, H.; Vreven, T.; Throssell, K.; J. A. Montgomery, J.; Peralta, J. E.; Ogliaro, F.; Bearpark, M. J.; Heyd, J. J.; Brothers, E. N.; Kudin, K. N.; Staroverov, V. N.; Keith, T. A.; Kobayashi, R.; Normand, J.; Raghavachari, K.; Rendell, A. P.; Burant, J. C.; Iyengar, S. S.; Tomasi, J.; Cossi, M.; Millam, J. M.; Klene, M.; Adamo, C.; Cammi, R.; Ochterski, J. W.; Martin, R. L.; Morokuma, K.; Farkas, O.; Foresman, J. B.; Fox, D. J. *Gaussian 16, Revision A.03* Gaussian Inc.: Wallingford CT, 2016.

(S22) Becke, A. D. Density-functional exchange-energy approximation with correct asymptotic behavior. *Phys. Rev. A* **1988**, *38*, 3098-3100.

(S23) Perdew, J. P. Density-functional approximation for the correlation energy of the inhomogeneous electron gas. *Phys. Rev. B* **1986**, *33*, 8822-8824.

(S24) Andrae, D.; Häußermann, U.; Dolg, M.; Stoll, H.; Preuß, H. Energy-adjustedab initio pseudopotentials for the second and third row transition elements. *Theor. Chim. Acta* **1990**, *77*, 123-141.

(S25) Hehre, W. J.; Ditchfield, R.; Pople, J. A. Self-Consistent Molecular Orbital Methods. XII. Further Extensions of Gaussian-Type Basis Sets for Use in Molecular Orbital Studies of Organic Molecules. *J. Chem. Phys,* **1972**, *56*, 2257-2261.

(S26) Hariharan, P. C.; Pople, J. A. The influence of polarization functions on molecular orbital hydrogenation energies. *Theor. Chim. Acta* **1973**, *28*, 213-222.

(S27) Höllwarth, A.; Böhme, M.; Dapprich, S.; Ehlers, A. W.; Gobbi, A.; Jonas, V.; Köhler, K. F.; Stegmann, R.; Veldkamp, A.; Frenking, G. A set of d-polarization functions for pseudo-potential basis sets of the main group elements Al-Bi and f-type polarization functions for Zn, Cd, Hg. *Chem. Phys. Lett.* **1993**, *208*, 237-240.

(S28) Weigend, F.; Ahlrichs, R. Balanced basis sets of split valence, triple zeta valence and quadruple zeta valence quality for H to Rn: Design and assessment of accuracy. *Phys. Chem. Chem. Phys.* **2005**, *7*, 3297-3305.

(S29) Weigend, F.; Köhn, A.; Hättig, C. Efficient use of the correlation consistent basis sets in resolution of the identity MP2 calculations. *J. Chem. Phys,* **2002**, *116*, 3175-3183.

(S30) Grimme, S.; Ehrlich, S.; Goerigk, L. Effect of the damping function in dispersion corrected density functional theory. *J. Comput. Chem.* **2011**, *32*, 1456-1465.

(S31) Tomasi, J.; Mennucci, B.; Cammi, R. Quantum mechanical continuum solvation models. *Chem. Rev.* **2005**, *105*, 2999-3093.

(S32) Laplaza, R.; Peccati, F.; A. Boto, R.; Quan, C.; Carbone, A.; Piquemal, J. P.; Maday, Y.; Contreras-García, J. NCIPLOT and the analysis of noncovalent interactions using the reduced density gradient. *WIREs Computational Molecular Science* **2020**.

(S33) Humphrey, W.; Dalke, A.; Schulten, K. VMD: Visual molecular dynamics. http://www.ks.uiuc.edu/Research/vmd/ **1996**, *14*, 33-38.
